# Antifungal Agent 4-AN Changes the Genome-Wide Expression Profile, Downregulates Virulence-Associated Genes and Induces Necrosis in *Candida albicans* Cells

**DOI:** 10.3390/molecules25122928

**Published:** 2020-06-25

**Authors:** Aleksandra Martyna, Maciej Masłyk, Monika Janeczko, Elżbieta Kochanowicz, Bartłomiej Gielniewski, Aleksandra Świercz, Oleg M. Demchuk, Konrad Kubiński

**Affiliations:** 1 Department of Molecular Biology, Institute of Biological Sciences, The John Paul II Catholic University of Lublin, ul. Konstantynów 1i, 20-708 Lublin, Poland; aleksandra.martyna@kul.pl (A.M.); maciekm@kul.pl (M.M.); mjanec@kul.pl (M.J.); mazure@kul.pl (E.K.); 2 Laboratory of Molecular Neurobiology, Neurobiology Center, Nencki Institute of Experimental Biology, Polish Academy of Sciences, 3 Pasteur Street, 02-093 Warsaw, Poland; b.gielniewski@nencki.gov.pl; 3 Institute of Computing Science, Poznan University of Technology, Piotrowo 2, 60-965 Poznań, Poland; aleksandra.swiercz@cs.put.poznan.pl; 4 Institute of Bioorganic Chemistry, Polish Academy of Sciences, Noskowskiego 12/14, 61-704 Poznań, Poland; 5 Pharmaceutical Research Institute, Rydygiera Street 8, 01-793 Warsaw, Poland; O.Demchuk@IFarm.eu

**Keywords:** 4-AN, *C. albicans*, gene expression, synergy, antifungals, necrosis

## Abstract

In the light of the increasing occurrence of antifungal resistance, there is an urgent need to search for new therapeutic strategies to overcome this phenomenon. One of the applied approaches is the synthesis of small-molecule compounds showing antifungal properties. Here we present a continuation of the research on the recently discovered anti-*Candida albicans* agent 4-AN. Using next generation sequencing and transcriptional analysis, we revealed that the treatment of *C. albicans* with 4-AN can change the expression profile of a large number of genes. The highest upregulation was observed in the case of genes involved in cell stress, while the highest downregulation was shown for genes coding sugar transporters. Real-time PCR analysis revealed 4-AN mediated reduction of the relative expression of genes engaged in fungal virulence (ALS1, ALS3, BCR1, CPH1, ECE1, EFG1, HWP1, HYR1 and SAP1). The determination of the fractional inhibitory concentration index (FICI) showed that the combination of 4-AN with amphotericin B is synergistic. Finally, flow cytometry analysis revealed that the compound induces mainly necrosis in *C. albicans* cells.

## 1. Introduction

Together with *Candida glabrata*, *Candida topicalis*, *Candida parapsilosis* and *Candida krusei*, *Candida albicans* cause over 95% of invasive candidiasis [1]. In comparison with other fungal pathogens, *C. albicans* threatens human health to the greatest extent [2]. The available systemic drugs against *C. albicans* mainly belong to three groups of chemicals: azoles, polyenes and echinocandins. The mechanisms of their action in fungal cells and the mechanisms of antifungal resistance are well recognized [3]. On one hand, antifungal drugs are effective in most cases; on the other hand, in some circumstances, fungi can develop different strategies of antifungal resistance, which poses a crucial problem for the fight against candidiasis [4].

**Figure 1:**
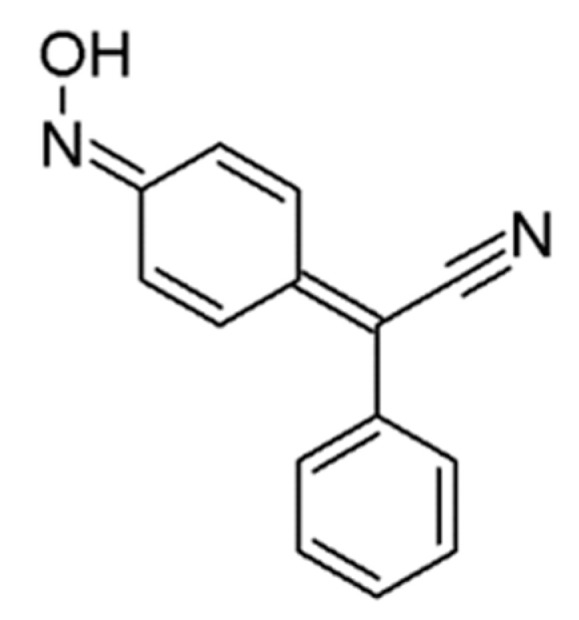
Chemical structure of 4-AN.

One of approaches to solving this issue is to discover new agents that overcome cellular and molecular antifungal resistance. Since a large number of chemicals derived from quinones show antifungal properties, we focused our research on this class of chemicals [5,6,7]. The 4-AN is a recently revealed antifungal agent containing a quinone methide moiety substituted with an oxime group (Figure 1). It is known that quinone methides can react with biological nucleophiles to give covalent bonds with proteins. Those protein alkylation reactions induce cellular biological pathway events that, in some cases, lead to antiproliferative cellular effects [8]. The 4-AN shows high anti-*C. albicans* activity with minimal inhibitory concentration/minimal fungicidal concentration (MIC/MFC) values of 4/4 µg/mL and effectively disturbs hyphal growth at the 4-AN concentration of MIC/16. Concurrently, at the effective anti-*C. albicans* doses, the compound does not exert any toxic effect on either human erythrocytes or Zebrafish embryos [9]. Since the structure of 4-AN resembles known protein kinase inhibitors on the one hand and the phosphorylation status of some Candida proteins correlates with fungal pathogenicity on the other hand, we examined the ability of 4-AN to inhibit protein kinases. The compound reduced the level of phosphorylation of cellular proteins and inhibited the activity of several protein kinases, e.g., CK2, DYRK1A, CLK1, CDK9/CyclinT and Pim1 in vitro. Based on these data, we assume that protein kinases may be cellular targets of 4-AN and the compound disturbs the growth of *C. albicans* through the modulation of cellular protein phosphorylation. We also observed that 4-AN shows fungistatic and fungicidal effects on clinical *C. albicans* isolates with MIC/MFC values of 4–256 µg/mL. It is noteworthy that the chemical can overcome the antifungal resistance towards the systemic drugs ketoconazole and caspofungin [10].

This study is a continuation of studies on the anti-*C. albicans* properties of 4-AN. Here we report changes in the genome-wide expression profile of *C. albicans* in response to 4-AN. Using real-time qPCR, we also examined the expression of genes important for biofilm formation and hyphal growth. Moreover, the synergism of the 4-AN action with some systemic antifungals was studied. Finally, using flow cytometry, we determined the type of cell death induced by 4-AN in *C. albicans* cells.

## 2. Results

### 2.1. Gene-Expression Response to 4-AN Exposure

**Figure 2:**
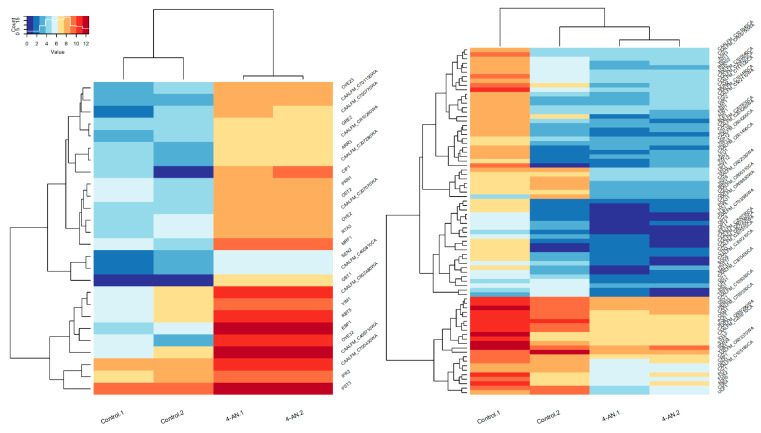
Heat maps of gene-expression log values in the response to 4-AN treatment of 104 differentially expressed genes. Left heat map presents 26 upregulated genes in 4-AN treat cells, while right heat map depicts 78 downregulated genes.

In order to explore the genome-wide expression profile of *C. albicans* in response to 4-AN, total RNA was isolated from *C. albicans* treated with the compound and from untreated fungi (control). RNA was sequenced, which was followed by bioinformatics analysis of the next generation sequencing (NGS) results (the complete analysis is available at https://www.ncbi.nlm.nih.gov/geo/query/acc.cgi?acc=GSE150837). A statistical difference in the expression (*p* ≤ 0.05) compared with the control cells) of 104 genes was revealed: 26 showed an increase and 78 showed a decrease in expression. The other genes that were affected but did not meet the criteria of the statistical significance (*p* > 0.05) are listed in the Supplementary Materials (Table S1). The pattern of the expression values for the differentially expressed genes is presented with heat maps (Figure 2). Expression values of each sample were log_2_ transformed for the clear view of low expressed genes. Left panel of Figure 2 presents upregulated genes, and the right one, downregulated. On each heat map hierarchical clustering (HC) indicates two groups of genes. The smaller groups include genes with significantly higher expression (marked in dark red) in 4-AN samples (for upregulated genes) or in control samples (for downregulated genes). It is worth noting that genes with high absolute values of log_2_-fold change > 6 (which is equivalent to expression ratio > 64) are equally distributed among the HC groups. The highest absolute log_2_-fold change was observed for 5 upregulated (GST1, EBP1, CAALFM_C406710WA, OYE32, CIP1) and 8 downregulated genes (OPT2, IFF11, HXT5, PLB1, CAALFM_C703560WA, CAALFM_CR03580CA, IHD1, HGT12).

**Figure 3:**
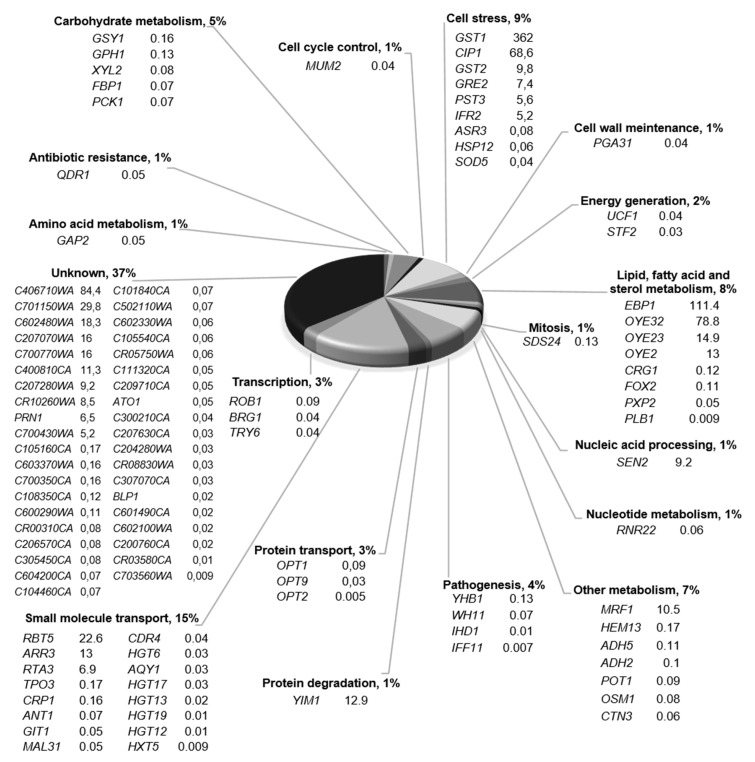
Distribution of 4-AN-responsive genes. The genes were assigned to functional categories according to the candida genome database (http://www.candidagenome.org). The average gene-expression ratio in cells treated with 4-AN vs control cells is presented.

The differentially expressed genes were grouped based on their function depicted in the candida genome database (http://www.candidagenome.org) (Figure 3). The largest group comprised genes with unknown function (36%), followed by small-molecule transport (15%), cell stress (8%), lipid, fatty acid and sterol metabolism (7%), other metabolism (6%), pathogenesis (6%) and carbohydrate metabolism (5%).

### 2.2. Response of Biofilm Formation—And Hyphal Growth-Associated Genes to 4-AN

**Figure 4:**
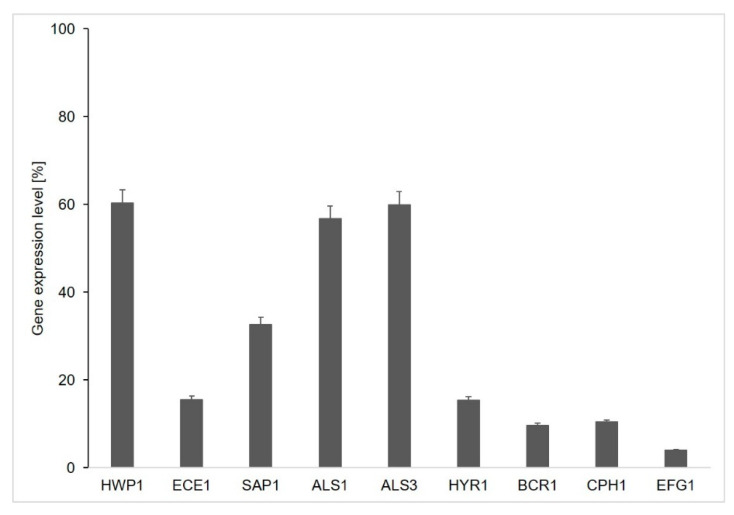
Relative expression of the selected genes in response to the 4-AN action detected by real-time PCR.

To explore the mechanism of the interference of 4-AN in C. albicans hyphal growth and biofilm formation, the effect of the compound on the expression of the selected genes involved in these processes was examined. *C. albicans* were cultured in the presence of the compound or without 4-AN (control). This was followed by RNA isolation and a real-time PCR assay of the selected genes, i.e., ALS1, ALS3, BCR1, CPH1, ECE1, EFG1, HWP1, HYR1 and SAP1 [11,12,13,14,15]. The 4-AN action resulted in suppression of all examined genes. The three genes responsible for C. albicans adhesion (ALS1, ALS3 and HWP1) were downregulated to a lesser extent (to about 60%). The expression of gene SAP1 encoding aspartyl proteinase important for virulence was reduced to 33%. The highest decrease (from 16% to 4%) was observed in the case of the expression of genes engaged in hyphal growth (ECE1, HYR1, CPH1, EFG1, HWP1). The 4-AN compound was also found to downregulate significantly (to 10%) the positive regulator of adherence BCR1 (Figure 4).

### 2.3. Combination of 4-AN with Antifungal Drugs

In order to verify whether 4-AN could be applied in combination with systemic antifungal drugs, the fractional inhibitory concentration index (FICI) was calculated with the use of the checkerboard method, which is the most commonly used approach to characterize the activity of antimicrobial combinations [16,17,18]. The 4-AN was examined in combination with the representatives of three groups of antifungals: azoles (ketoconazole), polyenes (amphotericin B) and echinocandins (caspofungin). The results revealed that 4-AN showed no interaction with caspofungin and ketoconazole, while the combination of 4-AN with amphotericin B was slightly synergistic (Table 1).

### 2.4. Effect of 4-AN on Cell Death in C. albicans Cells

**Figure 5:**
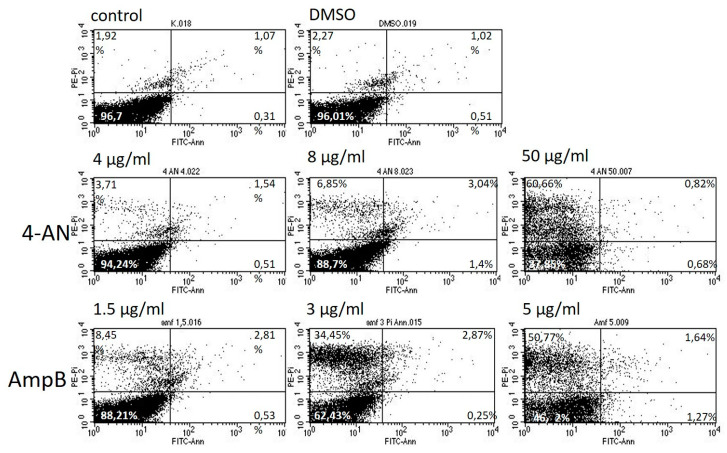
Flow cytometry analysis of *C. albicans* cells treated with 4-AN and amphotericin B (AmpB) (control).

To determine whether 4-AN kills cells through induction of apoptosis and/or necrosis, flow cytometry analyses were performed followed by annexin V- and PI-staining. The systemic drug amphotericin B was used as a positive control. The analysis showed that 4-AN and amphotericin B induced dose-dependent necrosis of *C. albicans* cells (Figure 5). The 4-AN at 4 (MIC), 8 and 50 µg/mL induced necrosis in 3.71, 6.85 and 60.66% of cells, respectively. Necrosis was significantly greater in response to 1.5 (MIC), 3 and 5 µg/mL, i.e., it was detected in 8.45, 34.45% and 50.77% of cells. The percentages of early and late apoptotic cells were low, from 0.51% to 3.04% and were not correlated with the doses of the antifungal agents.

## 3. Discussion

Gene-expression profiling experiments can reveal three main groups of responses: (i) responses consistent with the mechanisms of action of a compound, (ii) responses indicative of other pathways that may be affected by an agent and (iii) responses that reflect mechanisms of resistance to a drug [19]. In the case of 4-AN, the suggested mechanism of action in *C. albicans* cells is downregulation of cellular phosphorylation through inhibition of the activity of some protein kinases [9,10]. Since protein kinases can act as transcriptional factors or they regulate the activity of many transcriptional factors via phosphorylation, a decrease in the cellular activity of these enzymes can significantly disturb the expression of genes [20,21,22]. For example, protein kinase CK2—which is inhibited by 4-AN—upregulates the specific DNA-binding activity of the TATA-binding protein (TBP) by direct phosphorylation of this protein in yeast, which in turn activates RNA polymerase II [9,23]. Another kinase that is targeted by 4-AN is DYRK1A, and the Candida equivalent of the kinase is YAK1 [9]. Genome-wide analysis of DYRK1A-associated loci reveals that the kinase is recruited preferentially to promoters of genes actively transcribed by RNA polymerase II. The kinase phosphorylates the *C*-terminal domain of the polymerase at Ser2 and Ser5 and activates its association at the target promoters [24]. Taking the above into consideration, it can be assumed that many gene responses are a result of 4-AN-mediated inhibition of kinase activity in *C. albicans* cells (Figure 3).

A study on changes in the genome-wide expression profile of *C. albicans* in response to representatives of polyenes (amphotericin B), pyrimidines (Flucytosine), azoles (ketoconazole), and echinocandins (caspofungin) was conducted earlier [19]. In comparison with this study, the expression profile of *C. albicans* in response to 4-AN shows no similarity with any of the antifungals tested, which may be a result of different mechanisms of action. The highest upregulation mediated by 4-AN was observed in the case of cell stress genes (GST1 and CIP1) and genes regulating lipid, fatty acid and sterol metabolism (EBP1 and OYE32). These two groups of the analyzed genes contain the largest number of upregulated genes. The increase in the expression of GST1 coding glutathione S-transferase and genes CIP1, EBP1, and OYE32 coding oxidoreductases is a response of the *C. albicans* cell to the xenobiotic 4-AN, which induces oxidative stress. It is also worth mentioning that only 3 of the 16 responsive small molecule transport genes were upregulated (RBT5, ARR3 and RTA3). A common function of these genes is engagement in biofilm formation. Increased expression of three more genes (YIM1, MRF1 and SEN2) representing three distinct groups was observed. YIM1 and MRF1 encode mitochondrial proteins and are induced by the fungicide benomyl, while SEN2 encodes endonuclease.

The largest number of downregulated genes belongs to three functional groups, namely small molecule transport, other metabolism and carbohydrate metabolism genes. The lowest expression was observed in the case of genes coding sugar transporters (HXT5, HGT12, HGT19, HGT13, HGT17 and HGT6)

Since 4-AN can disturb both *C. albicans* hyphal growth and biofilm formation, we used real time PCR to examine whether the compound can affect the expression of genes engaged in hyphal growth, biofilm formation and virulence. It should be noted that the genes selected for this assay were also detected in the above-described genome-wide expression profiling. Nevertheless, none of these genes met the statistical criteria that were accepted during the bioinformatic analysis (Table S1). In contrast, it must be noted that most the genes selected for real-time PCR were downregulated in the genome-wide expression profiling experiment described above. The real-time PCR analysis revealed that the genes responsible for cell adhesion (HWP1, ALS1 and ALS2) were downregulated to a lesser extent. This corresponds to our recent data showing that 4-AN has no significant influence on the adhesion phase at the concentration of up to MIC/2, but it can destroy mature biofilm to 50% at the same concentration [10]. The largest decrease in expression was observed in the case of four genes having key roles in the morphogenetic regulation in *C. albicans* and in controlling the growth of hyphae (EFG1, CPH1, ECE1 and HYR1). The expression of the same genes in response to systemic drugs were examined by An and collaborators [14]. They showed that the genes were downregulated in response to Fluconazole, amphotericin B and fungichromin.

Synergistic drug combination is one of the strategies for searching drugs with a novel mode of action. There are several mechanisms proposed for antifungal synergy: (i) inhibition of different stages of the same biochemical pathway, (ii) increased penetration of an antifungal agent as a result of the cell wall activity of another agent, (iii) a transport interaction and (iv) simultaneous inhibition of different cell targets [18,19]. Based on the determined fractional inhibitory concentration index (FICI), 4-AN shows antifungal slight synergy with amphotericin B. Since the genome-wide expression profile of *C. albicans* in response to 4-AN is different than the profile in response to amphotericin B, the agents probably act in diverse biochemical pathways. Moreover, the compounds differ in their cellular targets: AmpB disturbs cell wall integrity, while 4-AN acts as a protein kinase inhibitor. Taking the above into consideration, it can be concluded that the antifungal combination of 4-AN and AmpB is a reasonable pathway in the development of this new antifungal agent.

Based on our recent studies, we suggest protein kinase inhibition as a possible cellular target of 4-AN. Many small-molecule inhibitors of protein kinases show the ability to induce apoptosis; hence, the compounds are considered as promising anti-cancer agents [25]. One of such molecules is staurosporine, which inhibits many protein kinases and thus induces apoptosis in a variety of cancer cells [26,27]. On one hand, 4-AN can act as a multikinase inhibitor. On the other hand, it induces mainly necrosis and a low level of apoptosis in *C. albicans*, as revealed by the flow cytometry analysis. A similar effect against *C. albicans* is also exerted by systemic antifungal drugs, amphotericin B and caspofungin, which can both induce necrosis and apoptosis with prevalence of necrotic cells [28,29]. Such an effect of 4-AN on fungal cells may be a result of some other cellular activity of the agent, beside inhibition of protein kinases.

## 4. Materials and Methods

### 4.1. C. albicans Cultures and RNA Isolation

*C. albicans* (ATCC 10,231) was cultured in duplicate in 100 mL flasks at 30 °C with vigorous shaking in the presence of 4-AN at the concentration of MIC/4 (1 µg/mL) or 0.1% DMSO (control cells in duplicate). The cultures were terminated by centrifugation when OD_600_ reached a value of 1. Cells were frozen at −70 °C or used immediately. Total RNA was isolated from four cell samples using a YeaStarTM RNA kit (Zymo Research, Irvine, CA, USA) according to the manufacturer’s protocol.

### 4.2. RNA Sequencing

The quality and integrity of total RNA was assessed with an Agilent 2100 Bioanalyzer using an RNA 6000 Nano Kit (Agilent Technologies, Ltd., Santa Clara, CA, USA). Strand-specific polyA enriched RNA libraries were prepared using the KAPA Stranded mRNA Sample Preparation Kit according to the manufacturer’s protocol (Kapa Biosystems, Wilmington, MA, USA). Briefly, mRNA molecules were enriched with 500 ng of total RNA using poly T oligo-attached magnetic beads (Kapa Biosystems). The mRNA was fragmented, and the first-strand cDNA was synthesized using reverse transcriptase. The second cDNA synthesis was performed to generate double-stranded cDNA (dsDNA). Adenosines were added to the 3′ ends of dsDNA and adapters were ligated (adapters from NEB, Ipswich, MA, USA). Following the adapter ligation, uracil in a loop structure of the adapter was digested by USER enzyme from NEB. Adapters containing DNA fragments were amplified by PCR using NEB starters. Library evaluation was done with an Agilent 2100 Bioanalyzer using the Agilent DNA High Sensitivity chip (Agilent Technologies, Ltd.) The mean library size was 300 bp. The libraries were quantified using a Quantus fluorometer and QuantiFluor double stranded DNA System (Promega, Madison, WI, USA). The libraries were run in the rapid run flow cell and were paired-end sequenced (2 × 76 bp) on HiSeq 1500 (Illumina, San Diego, CA, USA). The experiment was done in duplicate.

### 4.3. Sequencing Data Analysis

Raw paired-end reads were filtered according to the quality with the Trimmomatic tool with parameters SLIDINGWINDOW:6:20 MINLEN:35 [30]. On average 6.5% of low-quality reads were discarded from sequenced libraries. The remaining paired-end reads were mapped to the genome of *C. albicans* SC531 (assembly ASM18296v3) with STAR [31]. Depending on the library, 92%–95% of reads were uniquely mapped to the reference genome. The expression of all 6263 genes was calculated with the htseq-count tool [32]. The genes with very low expression across the examined samples were discarded from further analysis. Thus, we obtained 5775 genes having on average at least 2 mapped reads.

The differential expression analysis of the control and 4-AN treated sample was performed with the negative binomial (Gamma–Poisson) distribution implemented in the DESeq2 R package [33]. First, size factors for each sequencing library are estimated using the median ratio method. Next, coefficients of dispersion are calculated for each gene. Finally, counts for each gene are modeled using negative binomial distribution, normalized by the library size factors and gene specific coefficients of dispersion and the Wald test is used to test the significance of differential expression. The indicator that tells if differential expression of a gene is statistically significant is the *p*-value, which was later adjusted for multiple test correction by the Benjamini–Hochberg procedure to reduce false discovery rates (FDR). Hereafter, *p* stands for FDR adjusted *p*-value.

### 4.4. Real-Time PCR

The relative expression of genes associated with adhesion and hyphal growth of, i.e., ALS1, ALS3, BCR1, CPH1, ECE1, EFG1, HWP1, HYR1, and SAP1 was evaluated using two-step real-time PCR after treatment with 4-AN (1 µg/mL). Control cells were treated with DMSO (0.1%). Total RNA was converted to cDNA using smart a First Strand cDNA Synthesis Kit (EurX, Gdańsk, Poland). For PCR detection of transcripts, we used TaqMan Gene Expression Assays (Lot: 170255, designed by manufacturer, Thermo Fisher Scientific, Waltham, MA, USA) and Probe qPCR Master Mix (EurX). The cDNA samples were pre-treated at 50 °C for 2 min with UNG to degrade any dUMP-containing PCR products and then subjected to initial denaturation at 95 °C for 10 min., followed by 40 amplification cycles with denaturation at 94 °C for 15 s, annealing at 60 °C for 30 s and extension at 72 °C for 30 s using RotorGene-6000 (Corbett Life Science, Mortlake, Australia). The relative gene-expression level of the tested genes was calculated with the 2^−(ΔΔCt)^ method using ACT as a reference gene. The experiment was done in triplicate.

### 4.5. Determination of the Fractional Inhibitory Concentration Index

The effectiveness of the antifungal combination was determined using the checkerboard microdilution method [16]. The combinations of caspofungin/4AN, amphotericin B/4AN and ketoconazole/4AN were used at 1/16, 1/8, 1/4, 1/2, MIC, 2 × MIC and 4 × MIC. Minimal inhibitory concentrations (MIC) were determined as previously described [10]. The fractional inhibitory concentration index (FICI) values were calculated for each well with the equation FICI = FICA + FICB = (MICA + B / MICA) + (MICB + A / MICB), where MICA and MICB are the MICs of drugs A and B alone, respectively and MICA + B and MICB + A are the concentrations of the drugs applied in combination, respectively, in all the wells corresponding to the MIC. Drug interactions were classified as synergistic, indifferent, of antagonistic referring to the fractional inhibitory concentration index (FICI), which was defined as FICI ≤ 0.5, synergy; 0.5 < FICI ≤ 4, no interaction; FICI > 4, antagonism [1].

### 4.6. Flow Cytometry

Overnight cultures were refreshed in Sabouraud dextrose broth medium to the mid-exponential phase, then treated with 4, 8 and 50 µg/mL of 4-AN and with 1.5, 3 and 5-µg/mL of amphotericin B for 2 h (control). Then, the cells were harvested. To prepare protoplasts, *C. albicans* cells were incubated at 30 °C for 20 min. in 0.02 mg/mL zymolyase 20T in 0.1-M PPB buffer (50-mM K2HPO4, 5-mM EDTA, 50-mM DTT, 50-mM KH2PO4 and 40-mM β-mercaptoethanol) with 1-M sorbitol at pH 6. After that, the cell protoplasts were washed in PPB buffer with 1-M sorbitol and resuspended in 1× binding buffer (BD Pharmingen, San Diego, CA, USA) with 1-M sorbitol. Next, the FITC Annexin V Apoptosis Detection Kit I (BD Pharmingen) was used according to the manufacturer’s instructions. The stained cells were analyzed by flow cytometry (BD FACSCalibur).

## 5. Conclusions

Summing up, further data on anti-*C. albicans* agent 4-AN were revealed: (i) 4-AN can change the expression profile of a large number of genes. The highest upregulation was observed in the case of genes involved in cell stress, while the highest downregulation was shown for genes coding sugar transporters; (ii) 4-AN reduces the expression of genes engaged in fungal virulence; (iii) 4-AN shows slight synergism with amphotericin; (iv) 4-AN kills *C. albicans* cells mainly via necrosis.

## Figures and Tables

**Table 1:** Fractional inhibitory concentration index (FICI) values for 4-AN in combination with systemic antibiotics. FICI ≤ 0.5, synergism; 0.5 < FICI ≤ 4, no interaction; FICI > 4, antagonism.

	4-AN
Caspofungin	**1.5**
Amphotericin B	**0.45**
Ketoconazole	**1**

## Supplementary Materials

The following are available online at https://www.mdpi.com/1420-3049/25/12/2928/s1. Table S1: Transcriptome profile of *C. albicans* genes after 4-AN treatment.
