# Supplementary material for: Antifungal Agent 4-AN Changes the Genome-Wide Expression Profile, Downregulates Virulence-Associated Genes and Induces Necrosis in Candida albicans Cells
Source: Molecules. 2020 Jun 25;25(12):2928. doi: 10.3390/molecules25122928 (PMC7356344; doi:10.3390/molecules25122928)
Supplement: Supplementary file 1 [file molecules-25-02928-s001.pdf]

Supplementary material

The antifungal agent 4-AN changes the genome-wide expression profile, down-regulates virulence-associated genes and induces necrosis in *Candida albicans* cells

Aleksandra Martyna, Maciej Masłyk, Monika Janeczko, Elżbieta Kochanowicz, Bartłomiej Gielniewski, Aleksandra Świercz, Oleg M. Demchuk, and Konrad Kubiński*

Table S1. Transcriptome profile of *C. albicans* genes after 4-AN treatment

Gene	baseMean	log2FoldChange	lfcSE	stat	pvalue	padj
EBP1	1497,806686	6,794108741	0,7249343	9,372033493	7,11E-21	3,95E-17
_C406710WA	3020,310624	6,419655938	0,7095013	9,048123964	1,45E-19	4,04E-16
OYE32	805,2759316	6,273496533	0,7923963	7,917120083	2,43E-15	4,50E-12
CDR4	508,3831811	-4,544148955	0,7023087	-6,47030175	9,78E-11	1,36E-07
UCF1	416,2360412	-4,512925227	0,7209592	-6,25961292	3,86E-10	4,28E-07
HGT6	133,906572	-4,762076205	0,7662979	-6,21439287	5,15E-10	4,77E-07
RBT5	907,0208565	4,5208984	0,735818	6,144044087	8,04E-10	6,38E-07
_C701150WA	134,654893	4,944623594	0,8218976	6,016106975	1,79E-09	1,24E-06
CIP1	222,7617918	6,083635958	1,0153731	5,991527489	2,08E-09	1,28E-06
BRG1	124,033519	-4,581223782	0,7722079	-5,93262974	2,98E-09	1,65E-06
_C602480WA	584,6113993	4,195382938	0,7353374	5,705385155	1,16E-08	5,86E-06
AQY1	131,0466608	-5,128398457	0,9105998	-5,63189075	1,78E-08	7,61E-06
PCK1	1125,62616	-3,82333892	0,6786534	-5,63371366	1,76E-08	7,61E-06
_C207070WA	179,4326967	4,038811242	0,7693978	5,249314936	1,53E-07	6,05E-05
BLP1	151,8083043	-5,625917289	1,0783198	-5,21729952	1,82E-07	6,72E-05
GST1	45,93784342	8,546710643	1,6633641	5,138208002	2,77E-07	9,62E-05
ROB1	987,6902513	-3,511380321	0,6880328	-5,10350751	3,33E-07	0,000109
_C111320CA	244,253553	-4,278055165	0,8542935	-5,00771128	5,51E-07	0,000155
OYE2	216,4620416	3,66985391	0,7326483	5,009025522	5,47E-07	0,000155
YIM1	425,2559627	3,739711213	0,7472203	5,004830621	5,59E-07	0,000155
OYE23	148,9300365	3,893499462	0,8149296	4,777712663	1,77E-06	0,000469
HGT19	109,5312826	-5,839554135	1,2250328	-4,76685545	1,87E-06	0,000472
_C602330WA	206,3314178	-3,93154437	0,8356141	-4,70497633	2,54E-06	0,000564
OPT1	157,3449282	-3,384009761	0,7182071	-4,71174652	2,46E-06	0,000564
STF2	3073,717532	-4,838191994	1,0253329	-4,71865476	2,37E-06	0,000564
IHD1	33,34241698	-6,476889632	1,3813342	-4,688865	2,75E-06	0,000587
MRF1	275,8032924	3,408116585	0,7464092	4,566016567	4,97E-06	0,001022
_C700770WA	87,09978758	4,042478278	0,8907041	4,5385201	5,67E-06	0,001123
_CR00310CA	75,4273895	-3,602451522	0,7960962	-4,52514606	6,04E-06	0,001155
_C600290WA	744,035418	-3,125489716	0,7030794	-4,4454294	8,77E-06	0,001571

HXT5	62,39906685	-6,831518951	1,5362523	-4,44687291	8,71E-06	0,001571
MUM2	496,9714445	-4,670208509	1,0561852	-4,42177026	9,79E-06	0,001698
OSM1	1443,71257	-3,607167107	0,8183459	-4,40787596	1,04E-05	0,001756
HGT12	293,3398665	-6,125788015	1,4068852	-4,35414911	1,34E-05	0,002181
_C105540CA	271,9553879	-4,001239767	0,9345569	-4,28142996	1,86E-05	0,002863
_C207630CA	102,6053915	-4,893020568	1,141947	-4,28480543	1,83E-05	0,002863
GPH1	355,3632558	-2,863036181	0,6755389	-4,23815174	2,25E-05	0,003381
GST2	120,8214817	3,274609499	0,7793814	4,201549527	2,65E-05	0,003872
HGT13	107,6007914	-5,320940398	1,2719925	-4,18315398	2,87E-05	0,00399
RNR22	110,3607361	-3,875605685	0,9256726	-4,18679941	2,83E-05	0,00399
QDR1	148,2875182	-4,336175263	1,0381726	-4,17673838	2,96E-05	0,004004
ARR3	89,29904976	3,65637353	0,885203	4,130547958	3,62E-05	0,004632
_C204280WA	90,134	-4,986746137	1,207513	-4,12976599	3,63E-05	0,004632
_C206570CA	169,3173978	-3,640831053	0,8821455	-4,12724543	3,67E-05	0,004632
_C108350CA	1591,232348	-3,015802845	0,7438924	-4,0540846	5,03E-05	0,006165
OPT2	13,87769137	-7,620561222	1,8813405	-4,05060171	5,11E-05	0,006165
_CR03580CA	38,40799853	-6,624509999	1,6397763	-4,03988641	5,35E-05	0,006316
SOD5	25,42158517	-4,680082587	1,1768559	-3,97676775	6,99E-05	0,008079
_C601490CA	94,6957516	-5,161401788	1,2999566	-3,9704415	7,17E-05	0,008127
GIT1	21,99264637	-4,259476657	1,0925872	-3,89852325	9,68E-05	0,010745
_C209710CA	2069,405983	-4,15585679	1,0751679	-3,86530965	0,000111	0,01162
_C603370WA	470,1572498	-2,625177531	0,6786564	-3,86819841	0,00011	0,01162
_CR08830WA	41,41954731	-5,176923622	1,3373904	-3,87091422	0,000108	0,01162
WH11	139,9425408	-3,729905189	0,9662602	-3,86014564	0,000113	0,011649
RTA3	145,7792233	2,84781958	0,7426293	3,834779661	0,000126	0,012684
HGT17	51,15448728	-4,923823667	1,2864993	-3,82730376	0,00013	0,012842
PST3	2938,300872	2,507249065	0,6580091	3,810356181	0,000139	0,013514
_CR10260WA	73,23424144	3,090659767	0,8165059	3,7852267	0,000154	0,014698
YHB1	1731,923077	-2,850007347	0,7622297	-3,73903999	0,000185	0,01738
ADH2	117,5394934	-3,168498676	0,8612433	-3,67898193	0,000234	0,021559
_C307070CA	68,50465519	-5,143822606	1,3992936	-3,6760138	0,000237	0,021559
GAP2	161,0625896	-4,445829956	1,2108326	-3,67171318	0,000241	0,021571
_C305450CA	26,47833772	-3,589029091	0,9827731	-3,65194078	0,00026	0,022932
_C604200CA	63,44115969	-3,779152165	1,0481045	-3,60570184	0,000311	0,025745
_C700430WA	827,5311357	2,403548061	0,6679243	3,598533769	0,00032	0,025745
_CR05750WA	193,9781054	-3,913202868	1,083752	-3,61079177	0,000305	0,025745
GSY1	1291,668127	-2,554435604	0,7094962	-3,60035147	0,000318	0,025745
HEM13	1604,909664	-2,901428959	0,8031978	-3,61234663	0,000303	0,025745
PLB1	21,42089111	-6,812481043	1,8914552	-3,60171428	0,000316	0,025745
GRE2	69,66366349	2,897695604	0,8070279	3,590576756	0,00033	0,026165
CRP1	387,7839497	-2,591151003	0,7235873	-3,58097927	0,000342	0,026763
XYL2	1209,027375	-3,516568928	0,9841402	-3,57323975	0,000353	0,027184
_C300210CA	31,80677946	-4,498826744	1,2653651	-3,5553587	0,000377	0,028703
PRN1	110,6221018	2,670321408	0,7530278	3,546112686	0,000391	0,029327
_C602100WA	31,68460735	-5,736302016	1,6204068	-3,54003818	0,0004	0,02961
IFF11	9,082375476	-6,995521874	1,9845944	-3,52491272	0,000424	0,030941
ASR3	185,2257034	-3,533198436	1,003428	-3,52112792	0,00043	0,030979
_C703560WA	19,93539833	-6,706448617	1,9102861	-3,51070384	0,000447	0,031403
OPT9	19,98513705	-5,12912638	1,4607068	-3,51140044	0,000446	0,031403
_C207280WA	79,26821152	3,223320116	0,9223284	3,494764007	0,000474	0,03231

CTN3	33,36840862	-4,04212693	1,157146	-3,49318663	0,000477	0,03231
TRY6	20,52670961	-4,730732215	1,3526192	-3,49746061	0,00047	0,03231
MAL31	27,30491178	-4,332808177	1,2441356	-3,4825852	0,000497	0,033212
ADH5	622,7876702	-3,133270642	0,9026123	-3,47133598	0,000518	0,03382
IFR2	475,7063593	2,448630301	0,7053842	3,471342602	0,000518	0,03382
_C105160CA	265,7544215	-2,454321698	0,7135856	-3,43942139	0,000583	0,036829
FBP1	97,81231478	-3,710815535	1,0790378	-3,43900435	0,000584	0,036829
SDS24	2790,113991	-2,858800141	0,8310193	-3,44011279	0,000581	0,036829
TPO3	107,6491182	-2,536352551	0,7394554	-3,43002788	0,000604	0,037642
ANT1	141,6664125	-3,673375588	1,0753238	-3,41606463	0,000635	0,038769
_C200760CA	16,07401647	-5,322754765	1,5595583	-3,41298862	0,000643	0,038769
HSP12	68,75769245	-3,863531322	1,1316328	-3,41412105	0,00064	0,038769
_C104460CA	113,6986493	-3,748800728	1,1012162	-3,40423687	0,000663	0,039603
ATO1	99,53846641	-4,203715394	1,2391989	-3,39228475	0,000693	0,040049
_C101840CA	187,1822691	-3,66419172	1,0796793	-3,39377794	0,000689	0,040049
_C700350CA	1141,053486	-2,647384011	0,7798467	-3,39474939	0,000687	0,040049
POT1	587,8179702	-3,369089649	0,9939362	-3,38964385	0,0007	0,040049
_C502110WA	118,2678069	-3,783905857	1,1186042	-3,38270315	0,000718	0,040656
_C400810CA	29,0257234	3,46346343	1,0250793	3,378727302	0,000728	0,040832
PGA31	28,72625905	-4,643661072	1,3810186	-3,36248992	0,000772	0,042878
CRG1	36,90849228	-3,012107746	0,9001719	-3,3461473	0,000819	0,045036
FOX2	357,3909134	-3,053306652	0,9166398	-3,33097751	0,000865	0,047097
SEN2	35,46693812	3,235835727	0,973779	3,32296739	0,000891	0,048
PXP2	44,16992322	-4,120518314	1,245132	-3,30930227	0,000935	0,049921
RDN25	509101,5822	-3,590094134	1,0861928	-3,30520877	0,000949	0,050173
CUP1	307,4271953	-2,987782834	0,9048515	-3,30195948	0,00096	0,050279
PGA10	21,18088461	-4,718083452	1,4389062	-3,2789375	0,001042	0,054057
IFE2	684,6603072	-2,169403853	0,6626657	-3,27375307	0,001061	0,054549
RHR2	1204,401537	-2,33838221	0,7152479	-3,2693313	0,001078	0,0549
_C306860CA	70,49982565	-2,479922327	0,7658013	-3,23833637	0,001202	0,059863
_C702610CA	263,5567633	-3,01059217	0,9300491	-3,23702492	0,001208	0,059863
OPT3	44,01248938	-3,270796365	1,009915	-3,2386847	0,001201	0,059863
_C101220CA	41,90301039	-3,917165987	1,2177052	-3,21684273	0,001296	0,063669
_CR04220CA	34,89816761	-3,521555389	1,0969779	-3,21023375	0,001326	0,06458
DAK2	892,6076135	-2,183940616	0,6817264	-3,20354436	0,001357	0,065525
PRN4	111,510746	2,366227118	0,7400378	3,197440949	0,001387	0,06635
GLK1	693,484894	-3,012780704	0,9443487	-3,19032636	0,001421	0,067424
_C205130WA	47,27080424	-3,267377561	1,0298008	-3,17282485	0,00151	0,071017
_CR07140CA	19,45353967	-5,652588941	1,7829433	-3,17036941	0,001522	0,071018
_C102270CA	91,33270922	-3,310407101	1,0451556	-3,16738209	0,001538	0,071026
RDN58	1140,524041	-3,293100466	1,0403121	-3,16549268	0,001548	0,071026
ARF1	225,3199005	-3,213050329	1,0162682	-3,16161655	0,001569	0,071388
OPT8	186,5603789	2,368549359	0,7523119	3,148360743	0,001642	0,074099
CIRT4B	57,17330564	-3,152496459	1,0021334	-3,14578517	0,001656	0,074151
MAK16	267,4024408	2,439195027	0,78013	3,126652091	0,001768	0,078517
_C107160CA	53,8668563	-2,600357253	0,8328513	-3,12223456	0,001795	0,079073
ARA1	161,4588587	-3,043844657	0,9775247	-3,11382892	0,001847	0,08072
PGA37	25,12210584	-4,208542436	1,3526303	-3,11137678	0,001862	0,080757
ASR1	91,49263512	-3,434176134	1,110577	-3,092245	0,001986	0,084693
_C701070CA	364,0327236	-2,567093719	0,8298481	-3,09345032	0,001978	0,084693

CDR2	50,67738472	2,631984867	0,8516577	3,090425879	0,001999	0,084693
_C402930WA	16,82509463	-4,875056444	1,5876733	-3,07056654	0,002137	0,089848
FDH1	26,73991001	-3,110865307	1,0187185	-3,05370466	0,00226	0,09434
FCY2	78,24656532	-2,525926521	0,8278776	-3,05108708	0,00228	0,094456
LTV1	375,4859109	2,298659629	0,7576946	3,033754793	0,002415	0,099314
NBP2	438,1433633	-3,052168432	1,0097889	-3,02258056	0,002506	0,102297
AOX2	44,30117677	-2,71546105	0,8991475	-3,0200395	0,002527	0,102407
MAE1	325,626647	-2,20513499	0,7325742	-3,01011844	0,002611	0,105045
_C206630CA	16,50742346	-4,813262247	1,6092018	-2,99108679	0,00278	0,110222
PEX4	148,6498406	-3,05821388	1,0221141	-2,99204751	0,002771	0,110222
SOU2	7,896444078	-6,797111018	2,2749651	-2,98778698	0,00281	0,110628
_C301180CA	158,5390273	-2,223379321	0,7461027	-2,97999111	0,002883	0,112684
SLR1	3030,968938	-2,920316188	0,9828569	-2,97125266	0,002966	0,11513
GIT3	87,89561703	-2,219424491	0,7477738	-2,9680426	0,002997	0,115531
CDR11	126,4729396	-2,3760022	0,8037122	-2,95628474	0,003114	0,119201
ADR1	95,41354832	-2,714124143	0,9205298	-2,94843709	0,003194	0,121432
_C401300WA	1006,009775	-2,769319905	0,9426034	-2,93794817	0,003304	0,124762
_CR01710WA	109,2765805	2,255674658	0,7687598	2,934173389	0,003344	0,125437
NMD3	621,6173053	2,074451113	0,7076391	2,931509877	0,003373	0,125668
GPM2	229,1356035	-2,468135329	0,8459501	-2,91758967	0,003527	0,13054
CBF1	307,1291368	-2,021631348	0,6936607	-2,91443836	0,003563	0,130992
HPD1	17,6540787	-4,922385439	1,6921118	-2,90901895	0,003626	0,132408
MNN22	66,60263516	-2,301024691	0,7928201	-2,90232904	0,003704	0,134385
_C103270WA	128,4230926	-2,99183188	1,0325654	-2,89747434	0,003762	0,135596
HSP70	258,4716243	-2,107264848	0,7294374	-2,88889062	0,003866	0,138454
PFK2	4633,038374	-2,095895782	0,7265422	-2,8847543	0,003917	0,139387
SOD6	108,9728448	-2,682580204	0,9308293	-2,88192499	0,003953	0,139749
TRY4	6,091751829	-6,403080452	2,228898	-2,87275616	0,004069	0,142959
_C305750CA	28,98380506	-3,723757304	1,2972115	-2,87058613	0,004097	0,143038
RDN18	177829,8562	-3,102177707	1,0829761	-2,86449335	0,004177	0,144908
HMO1	1597,888274	-2,457974788	0,8618025	-2,85213232	0,004343	0,149729
_C209240CA	33,72478566	-3,135458561	1,1015346	-2,8464458	0,004421	0,151488
JEN1	10,38404269	-5,730928369	2,0176315	-2,84042368	0,004505	0,153431
_C403000CA	19,97104762	-3,412210659	1,2032699	-2,83578152	0,004571	0,15473
DOS2	475,6939155	-2,765317299	0,9761942	-2,83275314	0,004615	0,155256
_C100020CA	1442,073182	-2,916967885	1,0325754	-2,8249443	0,004729	0,158133
PHO89	63,12894869	-2,613370894	0,9282109	-2,81549255	0,00487	0,161885
PTC4	144,8805887	-2,532685431	0,9001812	-2,81352835	0,0049	0,161908
HGT10	79,91845186	-2,219305099	0,789443	-2,81122902	0,004935	0,162104
_C703200CA	174,4609652	-1,973717268	0,7027643	-2,80850514	0,004977	0,162521
_CR00090CA	148,5925912	2,436851563	0,8706262	2,798964153	0,005127	0,166422
TRY5	23,50831609	-3,212207903	1,1487246	-2,79632546	0,005169	0,166812
RSN1	22,29075575	-3,415971897	1,2242752	-2,79019935	0,005268	0,169019
GAL10	154,2254894	-2,387910164	0,8595159	-2,77820363	0,005466	0,174379
LEU42	687,4719205	-2,508708209	0,9052326	-2,77134097	0,005583	0,17708
ECI1	178,3599189	-2,880727202	1,0416995	-2,76541083	0,005685	0,179307
FRP1	64,66621302	2,179303766	0,7890906	2,76179158	0,005749	0,179965
KTI12	90,4281665	2,502283636	0,9064511	2,760527935	0,005771	0,179965
ALD5	3399,40906	-2,272441035	0,8240475	-2,75765766	0,005822	0,180277
FMP45	12,74978656	-4,377671351	1,5888819	-2,75518996	0,005866	0,180277

RAS2	10,23917	-3,711347151	1,3473779	-2,75449609	0,005878	0,180277
_C407150WA	37,82792148	2,636826845	0,9601094	2,746381662	0,006026	0,18189
ECM38	41,76376246	-2,581807333	0,940141	-2,74619176	0,006029	0,18189
GCA2	19,13988885	-3,506089322	1,2752915	-2,74924549	0,005973	0,18189
_C104200CA	493,2801445	-2,720199234	0,9927023	-2,74019632	0,00614	0,184241
_C502690WA	51,09507296	-2,860767127	1,0448835	-2,73788151	0,006184	0,184545
ATO9	17,55709332	-4,107925271	1,5027724	-2,73356454	0,006265	0,185982
_CR07840CA	13,35011434	-3,653678812	1,3440644	-2,71838083	0,00656	0,193701
_C108530WA	29,10750546	-3,220578368	1,1886911	-2,70934848	0,006742	0,19696
_C401760WA	126,9402323	-2,300913923	0,8487399	-2,71097661	0,006709	0,19696
_C301060WA	12,07058319	-4,325417238	1,5975561	-2,70752132	0,006779	0,19701
MRV8	5,922333058	-6,371256644	2,3681188	-2,69042944	0,007136	0,206313
APS3	125,8511342	-2,692273531	1,0037891	-2,68211078	0,007316	0,208306
ASR2	132,5803914	-2,600493058	0,9695961	-2,68203756	0,007318	0,208306
_C101610CA	13,75559539	-3,765554812	1,4032074	-2,68353402	0,007285	0,208306
_C200770WA	33,94064534	-2,721643924	1,0160765	-2,67858175	0,007393	0,208331
_C304150WA	2448,651539	-2,672672384	0,9977687	-2,67864937	0,007392	0,208331
_C504260WA	12,11661892	-3,871326438	1,4495497	-2,67070961	0,007569	0,212203
OSM2	132,0787293	-2,310581931	0,8664713	-2,66665733	0,007661	0,213699
_C101930WA	129,2617218	-2,602791181	0,9831137	-2,64749769	0,008109	0,21851
_C110130WA	25,79092277	-3,139232197	1,1846054	-2,65002365	0,008049	0,21851
_C600810CA	7,837206556	-4,286052767	1,6188204	-2,64763954	0,008106	0,21851
_C601560WA	414,9417417	-2,609058837	0,9818013	-2,65742041	0,007874	0,21851
_CR08670CA	25,91060282	-3,38617291	1,2761928	-2,65333964	0,00797	0,21851
HBR3	324,4851166	2,00469148	0,7552934	2,654189191	0,00795	0,21851
POP3	34,14603917	2,468112243	0,9305486	2,652319616	0,007994	0,21851
SLK19	5526,909357	-2,423122255	0,9210169	-2,63092043	0,008515	0,228353
_C107080WA	34,74258535	-2,875625397	1,0978742	-2,61926684	0,008812	0,234133
PFK1	1965,276171	-1,849872414	0,706731	-2,61750562	0,008858	0,234133
RRP15	211,21305	2,128041138	0,812722	2,618412285	0,008834	0,234133
_C602450WA	9,62749201	-3,972015388	1,520173	-2,61287065	0,008979	0,236208
IFA14	7,667787785	-4,248673686	1,6296635	-2,60708654	0,009132	0,239102
_C603290WA	498,717608	-2,486346184	0,9563773	-2,59975458	0,009329	0,240863
FRE7	216,4814179	1,822166946	0,7002329	2,60223	0,009262	0,240863
SOU1	115,1777396	-2,214868047	0,8519041	-2,59990305	0,009325	0,240863
_C306370CA	214,3818077	2,067865013	0,7962105	2,597133428	0,009401	0,241585
BBC1	1034,336671	-2,401672381	0,9285333	-2,58652256	0,009695	0,244262
DAP1	117,6289606	-2,487052247	0,9608145	-2,58848336	0,00964	0,244262
NCS2	92,43440062	2,036899819	0,7868495	2,588677904	0,009635	0,244262
RPR1	469,7405065	-2,757550411	1,0657423	-2,58744573	0,009669	0,244262
TNA1	16,26218567	-3,964511754	1,5333831	-2,58546724	0,009725	0,244262
GDH2	944,519781	-1,799880816	0,6977677	-2,57948422	0,009895	0,247415
_C106410WA	75,35420243	-2,168388276	0,8420563	-2,57511087	0,010021	0,249441
CYS3	1671,971235	1,6895335	0,656854	2,572159821	0,010107	0,250455
ADH1	8256,841344	-1,700549343	0,6628332	-2,56557672	0,0103	0,253266
_CR07700WA	44,38238557	-2,66184263	1,0376702	-2,56521053	0,010311	0,253266
_C111200WA	522,3175533	-1,997127925	0,779184	-2,56310184	0,010374	0,253687
_C703780CA	10,13695333	-3,32081261	1,2981039	-2,55820255	0,010521	0,256161
MRV5	8,261679273	-5,354119115	2,096976	-2,55325727	0,010672	0,258693
_C100310WA	59,92841851	-2,110765427	0,8290967	-2,54586164	0,010901	0,262913

MAF1	160,1780447	-1,788796889	0,702983	-2,54458072	0,010941	0,262913
_C113130CA	147,9373177	1,947930339	0,7659808	2,543053809	0,010989	0,262927
_C101360CA	499,9283361	-1,810249305	0,7162941	-2,52724301	0,011496	0,272832
_C114190CA	29,86807057	-2,348971784	0,9295156	-2,52709229	0,011501	0,272832
_C402260CA	82,99918715	2,603331311	1,0310679	2,524888256	0,011574	0,273381
_C200510WA	50,77192927	-2,713707397	1,0760734	-2,52186075	0,011674	0,274577
_C301130CA	15,68245974	-3,036567382	1,2054896	-2,51894948	0,011771	0,275689
_C407210WA	161,5055046	-2,315592369	0,9238794	-2,50637955	0,012197	0,282117
GAT1	27,98877023	-2,431527468	0,9691545	-2,50891633	0,01211	0,282117
PDC11	8761,985009	-1,731362222	0,6906712	-2,50678225	0,012184	0,282117
GAC1	70,05296478	-2,370603683	0,9480613	-2,50047517	0,012403	0,285673
RHD3	898,2453856	-1,960276625	0,7850001	-2,49716745	0,012519	0,287161
_C208630CA	383,0104793	-2,182355902	0,8757429	-2,49200536	0,012702	0,290169
_C401800WA	6,774658198	-5,085469935	2,0457734	-2,48584222	0,012925	0,291642
_C600930CA	39,92655363	-2,447312988	0,9839174	-2,48731551	0,012871	0,291642
GDH3	1213,078534	1,638218552	0,6586849	2,48710507	0,012879	0,291642
ICL1	106,9733879	-2,669487491	1,0762078	-2,48045727	0,013121	0,291701
REI1	273,4861165	1,938820479	0,7810628	2,482284917	0,013054	0,291701
SUC1	88,28064864	-2,075933742	0,8370618	-2,48002442	0,013137	0,291701
YVH1	57,71112182	2,169783511	0,8739427	2,482752512	0,013037	0,291701
_C202220CA	7,603642267	-5,226884146	2,1142228	-2,47224856	0,013427	0,292279
_C301540WA	7,712295225	-3,692467448	1,4922111	-2,4744941	0,013343	0,292279
_C406420WA	211,5420143	-2,087420228	0,8435149	-2,47466917	0,013336	0,292279
DOT5	186,0552671	-2,220848948	0,8982751	-2,47234843	0,013423	0,292279
IFR1	13,59982347	3,1564282	1,2738668	2,477832191	0,013218	0,292279
_CR02030CA	94,9378465	1,879130014	0,7605773	2,470662799	0,013486	0,292406
MRV2	14,10119251	-3,505921182	1,4198045	-2,46929858	0,013538	0,292406
_CR09150WA	13,72428381	-2,987274993	1,2141037	-2,46047756	0,013875	0,298532
_C100030CA	83,29532975	-2,474645887	1,0069532	-2,45755789	0,013989	0,298655
PEX13	386,0408853	-2,3733064	0,9654034	-2,45835728	0,013957	0,298655
_C400080CA	102,6116441	-2,870257898	1,1703089	-2,4525644	0,014184	0,300955
GCN5	343,3768167	-2,29652808	0,9371005	-2,45067419	0,014259	0,300955
RTA2	193,5907127	1,816320582	0,7410596	2,450977866	0,014247	0,300955
_C210870WA	240,8429498	-2,418109153	0,9918281	-2,4380324	0,014767	0,310508
_C600230WA	78,69236852	-2,465760449	1,0127661	-2,434679	0,014905	0,312218
RDN5	227,3409795	-2,463496749	1,0204204	-2,41419791	0,01577	0,328254
ROA1	202,8604983	1,871919253	0,7755199	2,413760399	0,015789	0,328254
_C302040CA	198,1834231	1,8455219	0,7652979	2,411507719	0,015887	0,329057
HXK2	6438,002361	-1,722506935	0,7159711	-2,40583294	0,016136	0,33297
GDB1	157,6195293	-1,694379711	0,7059731	-2,40006285	0,016392	0,337013
OFR1	213,6769377	-1,992380135	0,8358911	-2,38354023	0,017147	0,351229
_C111000CA	136,4567848	1,703179579	0,7153505	2,380902298	0,01727	0,351902
SOD3	547,6482647	-2,242559383	0,9422014	-2,38012745	0,017307	0,351902
BUD21	161,0028069	1,691750908	0,7118282	2,376628127	0,017472	0,353961
ALS4	152,023961	-1,704758247	0,7177235	-2,37522967	0,017538	0,354013
AXL1	33,64968918	-2,306174949	0,9730222	-2,37011542	0,017783	0,356516
_C103120WA	137,4044785	-1,850496942	0,7816676	-2,36737062	0,017915	0,356516
RRN3	265,244505	1,928907907	0,8148171	2,367289405	0,017919	0,356516
YTM1	368,7170035	1,783278758	0,7526984	2,369181033	0,017828	0,356516
_C402110WA	86,96763714	-2,189142058	0,9261347	-2,36374056	0,018091	0,357387

PEX22	62,99107052	-2,402201504	1,0158957	-2,36461429	0,018049	0,357387
DAL9	37,00064003	-2,581604541	1,0948751	-2,35789866	0,018379	0,360698
MLS1	185,4668411	-1,972244951	0,8365156	-2,35769059	0,018389	0,360698
_C112060CA	27,4704661	2,362556058	1,0045602	2,35183116	0,018681	0,362488
_C114480WA	173,2323482	-2,52437798	1,0746961	-2,34892264	0,018828	0,362488
_C307660WA	53,42423904	2,065211137	0,8795438	2,348048018	0,018872	0,362488
_C401570CA	42,82630506	-2,44234208	1,0399927	-2,34842234	0,018853	0,362488
DUG3	80,30227831	-2,014245063	0,856503	-2,35170813	0,018687	0,362488
MNN1	13,02173936	-3,097977915	1,3175383	-2,35133799	0,018706	0,362488
_C107400CA	114,6400935	-1,945163403	0,8292989	-2,34555165	0,018999	0,36294
_C110970WA	607,7036506	1,560426284	0,6654282	2,344995549	0,019027	0,36294
_C303980CA	23,70165579	2,31749745	0,9909533	2,338654499	0,019353	0,36294
_CR07160CA	55,15387566	-2,595239022	1,1079483	-2,34238275	0,019161	0,36294
MTG1	49,10137792	1,913643333	0,8180299	2,339331813	0,019318	0,36294
MTG2	106,9935216	1,826824656	0,7802296	2,34139383	0,019212	0,36294
NSA1	197,4511622	1,719865639	0,7350122	2,339914498	0,019288	0,36294
_C207790CA	22,77160038	2,670514244	1,1425784	2,33726997	0,019425	0,363061
_C600980CA	27,90660274	-2,544034269	1,090396	-2,33312881	0,019641	0,363323
MET10	330,0104964	1,653711818	0,7095187	2,330751589	0,019766	0,363323
PEX12	129,9636607	-1,98029672	0,8494459	-2,33128048	0,019739	0,363323
PIF1	249,7344175	1,691079084	0,7250178	2,332465685	0,019676	0,363323
RSM22	155,9038916	1,985502316	0,8517067	2,331204387	0,019743	0,363323
_CR07250CA	34,32213891	-2,874846334	1,2341874	-2,32934336	0,019841	0,363488
_C210600WA	79,31885271	-2,305949105	0,9929009	-2,32243642	0,020209	0,366994
_C307800CA	82,43541969	2,259001188	0,9720861	2,323869399	0,020132	0,366994
_C502900WA	1118,943873	-2,121456324	0,9140991	-2,32081666	0,020297	0,366994
YTH1	183,8412065	-1,647076375	0,7094673	-2,32156785	0,020256	0,366994
SSY1	69,34534655	-1,923976926	0,8297373	-2,3187783	0,020407	0,367791
CAP1	255,438619	1,773365894	0,7655967	2,316318695	0,020541	0,368488
NAG3	21,65661248	-2,332307063	1,0072023	-2,31562915	0,020579	0,368488
ATC1	190,4496155	-2,212261287	0,957097	-2,31142852	0,020809	0,368557
BMT4	600,8745829	-2,413783972	1,0434504	-2,31327149	0,020708	0,368557
_C201920CA	190,5613952	-2,04448705	0,8852404	-2,30952738	0,020914	0,368557
FPG1	201,3846407	-2,169731153	0,9392422	-2,3100869	0,020883	0,368557
tV(UAC)1	10,78090683	-3,690158525	1,5961281	-2,31194379	0,020781	0,368557
_C107780WA	267,7684534	-1,818222762	0,7879123	-2,30764609	0,021019	0,369226
_C306760WA	145,64673	1,95318069	0,8469756	2,306064795	0,021107	0,369606
_C206740WA	121,2766209	-1,759646533	0,764002	-2,30319612	0,021268	0,37125
_C401860CA	21,53081944	-2,568185251	1,1197428	-2,2935493	0,021816	0,376523
_CR08500WA	289,8082446	1,723673952	0,7516726	2,293118013	0,021841	0,376523
FAA2-3	119,0981119	-2,225631369	0,9703161	-2,29371784	0,021807	0,376523
RPN1	1204,228509	-1,815224108	0,7910134	-2,29480832	0,021744	0,376523
_C501420WA	79,79241182	-2,14091522	0,9353446	-2,28890528	0,022085	0,379295
GPI19	43,87759102	-2,419208491	1,057897	-2,28680901	0,022207	0,379295
SPT5	1966,88434	-1,940927516	0,8486366	-2,28711276	0,022189	0,379295
_C111670WA	298,6338963	-1,787959425	0,7862344	-2,27407937	0,022961	0,386268
_C505340WA	634,5726316	1,524844627	0,6704491	2,274363057	0,022944	0,386268
CAT1	703,7225453	-1,984795698	0,8718967	-2,2764115	0,022821	0,386268
HGT1	148,2769028	-1,749303836	0,769247	-2,27404704	0,022963	0,386268
PHO100	6,372880141	-3,938445268	1,7287279	-2,27823314	0,022713	0,386268

ALS2	143,3585048	-1,660520109	0,731906	-2,26876137	0,023283	0,389286
_C403720CA	301,3482036	1,681035517	0,7408915	2,268936049	0,023272	0,389286
_CR10620CA	237,3526533	1,602298054	0,706697	2,267305541	0,023372	0,389313
PAN1	2131,459423	-1,804731403	0,796286	-2,26643618	0,023425	0,389313
TSA1	37,76974016	-2,53204997	1,1188188	-2,26314566	0,023627	0,391498
CNS1	124,2095055	1,833656065	0,8107563	2,261661261	0,023718	0,391847
_C306490WA	205,8429121	-2,470982292	1,0938599	-2,25895683	0,023886	0,393447
_C702920WA	15,47376482	-3,012199545	1,3352131	-2,25596919	0,024073	0,395346
_C205990CA	13,79092732	-3,046474786	1,3518541	-2,25355295	0,024224	0,396664
ACS1	245,7395273	-1,641080194	0,7305207	-2,24645274	0,024675	0,398621
_C102060WA	76,3370696	-2,009500207	0,8938253	-2,24820234	0,024563	0,398621
_C205050CA	70,08583248	1,842547191	0,8203618	2,246017655	0,024703	0,398621
NOG2	419,771825	1,683243415	0,7490237	2,247249954	0,024624	0,398621
PGA48	237,554362	-2,106660786	0,9378554	-2,24625339	0,024688	0,398621
_C202260WA	840,9408531	-2,111191067	0,9433235	-2,23803497	0,025219	0,402268
CDC34	510,1082612	-1,753284578	0,783278	-2,23839384	0,025195	0,402268
FRE30	64,92730384	1,924882704	0,8589756	2,240905079	0,025032	0,402268
IMP4	176,3325481	1,986194545	0,8870349	2,239139031	0,025147	0,402268
_C300440WA	14,49652835	-2,901992109	1,2994594	-2,23323034	0,025534	0,406122
TEC1	78,39133209	-1,896428245	0,8496066	-2,23212508	0,025607	0,406122
MNN4	806,2898444	-1,853391448	0,8337808	-2,22287607	0,026224	0,41473
_C209280CA	66,30781715	-2,22098024	1,0007806	-2,21924792	0,02647	0,416244
PEX6	135,5267885	-1,835778759	0,8270528	-2,21966329	0,026442	0,416244
ARG83	41,43384751	-2,033311935	0,9167029	-2,21807074	0,02655	0,416325
_C208180CA	353,8882825	1,850686133	0,8355423	2,214952116	0,026763	0,418488
TPI1	3045,156497	-1,714285813	0,7781663	-2,20298135	0,027596	0,430297
_C700030WA	423,5519013	-2,016927867	0,9163704	-2,20099626	0,027736	0,431272
_C305990CA	36,26404478	-2,154926272	0,979863	-2,1992117	0,027863	0,43203
_C303920WA	156,2458579	-2,041201946	0,9301884	-2,19439633	0,028207	0,436147
_C111270WA	255,0871611	-2,167576686	0,9894833	-2,19061473	0,02848	0,437924
GAL1	183,8214048	-1,702598692	0,7770606	-2,19107576	0,028446	0,437924
DES1	660,6859842	-1,922969785	0,8794912	-2,18645718	0,028782	0,441353
NOC4	176,1854059	2,255342309	1,0339201	2,181350594	0,029157	0,445877
MDH1-3	152,0883705	-1,855001326	0,8509155	-2,18000645	0,029257	0,446169
ASF1	278,1968201	-2,108387473	0,9680606	-2,17794993	0,02941	0,44727
RPA12	42,65558885	2,10876529	0,9695392	2,175018155	0,029629	0,449369
_C503510CA	33,16356301	-1,969835798	0,9067817	-2,17233744	0,02983	0,450392
RBR2	62,92885847	-2,292457818	1,0554774	-2,17196298	0,029858	0,450392
_CR07190WA	127,5088128	-1,67913615	0,7739144	-2,16966651	0,030032	0,451784
_C111890WA	48,52199142	-2,035158471	0,9396793	-2,16580118	0,030326	0,454978
_C210650WA	34,59562	-2,60107783	1,2020193	-2,16392356	0,03047	0,455903
_C402520CA	77,72589435	-1,887034831	0,8736603	-2,15991827	0,030779	0,459286
_C403600CA	52,4760793	-1,702841138	0,7889283	-2,15842327	0,030895	0,45978
_C105360CA	178,5443398	1,562013774	0,7245618	2,155804723	0,031099	0,460347
_C400680WA	107,5223421	-1,983280095	0,9199543	-2,15584625	0,031096	0,460347
FBA1	8334,443926	-1,561251454	0,7246954	-2,15435529	0,031212	0,46053
HCA4	695,200062	1,436130433	0,6672005	2,152472041	0,03136	0,46053
NCR1	135,3565581	-1,65050668	0,7667059	-2,15272471	0,03134	0,46053
_C209660WA	163,0463427	1,748356468	0,8133009	2,149704406	0,031579	0,462514
_C203110WA	13,77994604	2,513316244	1,1697909	2,148517583	0,031673	0,462671

SDA1	479,0841191	1,766417574	0,8232977	2,145539264	0,03191	0,464911
_C302880WA	9,443666445	-3,508948507	1,6369163	-2,1436334	0,032062	0,46591
HOL4	159,8057319	-1,783115153	0,8340465	-2,13790861	0,032524	0,47016
NAR1	202,1391076	1,497160479	0,7001902	2,138219725	0,032499	0,47016
ALD6	19,45240486	-2,428730829	1,1375418	-2,13506951	0,032755	0,472272
_C203780CA	440,2431388	-2,058494441	0,967768	-2,12705359	0,033416	0,480544
HMS1	228,094618	-1,459170482	0,6867532	-2,1247379	0,033608	0,481505
WAL1	505,7942981	-1,661671231	0,7822683	-2,12417057	0,033656	0,481505
BUD22	155,6754777	1,772120704	0,8347194	2,123013628	0,033753	0,481649
_C209820WA	20,34794446	-2,095590689	0,9891365	-2,11860615	0,034124	0,485257
FAL1	115,4501999	1,638909776	0,7738237	2,117936924	0,03418	0,485257
TAZ1	72,17971137	1,632653863	0,7714616	2,116312569	0,034318	0,485971
_C204620WA	843,2083607	-1,575689845	0,7452798	-2,11422583	0,034496	0,487245
PTP2	44,05834238	-1,803799656	0,8540688	-2,11200735	0,034686	0,488683
POX1-3	261,7707991	-1,667681459	0,7900875	-2,11075552	0,034793	0,488956
_C200410CA	566,7905065	1,418129063	0,6732624	2,10635423	0,035174	0,49181
MDG1	1322,761415	-1,518717334	0,7207913	-2,10701402	0,035116	0,49181
_C100580WA	71,86281644	-2,176409253	1,0351314	-2,10254397	0,035506	0,495206
HGT7	6412,222776	-1,398879258	0,6657449	-2,10122424	0,035621	0,495573
_C205810WA	38,45336907	-2,035128108	0,9725483	-2,0925727	0,036387	0,498731
_C400590CA	397,666379	-1,494248767	0,7130062	-2,09570221	0,036109	0,498731
_C403770WA	109,6968544	-1,998444027	0,9538125	-2,09521684	0,036152	0,498731
_C700310CA	80,78179716	-1,80839924	0,8631881	-2,09502341	0,036169	0,498731
_CR08920WA	120,6852335	-1,903890242	0,9097745	-2,09270567	0,036375	0,498731
EHD3	84,39480276	-1,9951983	0,9531766	-2,09320945	0,03633	0,498731
_C300850CA	1239,325319	-1,61936832	0,7754904	-2,08818618	0,036781	0,50165
FOX3	160,4964553	-1,79385451	0,8587025	-2,08902902	0,036705	0,50165
GCS1	187,9359418	1,556507583	0,7459067	2,086732274	0,036912	0,502207
_C405900CA	58,44411887	-1,655809525	0,7950187	-2,08273041	0,037276	0,504885
SEO1	96,57835703	1,719497969	0,8256643	2,082563157	0,037291	0,504885
_C601590WA	816,8043804	-1,869679027	0,8983975	-2,08112676	0,037422	0,505429
_C400170WA	277,0830221	-1,729104334	0,8316678	-2,07908049	0,03761	0,506514
SMT3	804,3140088	-1,86538762	0,8975704	-2,07826319	0,037685	0,506514
_CR04970CA	6,950836544	-4,070391676	1,9608206	-2,07586129	0,037907	0,508262
SFC1	32,3747429	-2,350494417	1,1334796	-2,07369811	0,038107	0,50972
_CR10470CA	226,1450849	1,577140369	0,7620755	2,069532978	0,038496	0,512822
MEF2	205,57756	1,59701871	0,7717916	2,069235712	0,038524	0,512822
_C201450CA	10,58235118	-2,584046072	1,2501037	-2,06706533	0,038728	0,513276
TPS2	885,5770902	-1,44725647	0,7002043	-2,06690594	0,038743	0,513276
SPS20	64,14705352	-1,978035486	0,9598323	-2,06081369	0,039321	0,51969
_C210740CA	346,432739	1,689450999	0,8205761	2,058859711	0,039508	0,520205
CCC1	274,3606466	1,593260792	0,774011	2,058447143	0,039547	0,520205
_C503670CA	33,25989606	-1,780311356	0,8653641	-2,05729735	0,039658	0,520424
_C502520WA	14,01957768	-2,261114969	1,1002719	-2,05505117	0,039874	0,521775
HNM3	19,77901031	-2,287254178	1,1134087	-2,05428089	0,039949	0,521775
HGT2	359,5819408	-1,964082099	0,9565528	-2,0532918	0,040044	0,521798
_C500100CA	131,6329897	-1,937866298	0,9467713	-2,0468156	0,040676	0,527579
GPX2	61,17985352	-1,928973846	0,9426221	-2,04639154	0,040718	0,527579
HNT2	23,14098232	-2,423486156	1,184598	-2,04583	0,040773	0,527579
RPF1	252,1765459	1,509613765	0,739085	2,042544127	0,041098	0,530541

_C505510CA	146,3629349	-2,065861449	1,0127895	-2,03977382	0,041373	0,532856
_C300170CA	179,5201785	-1,592443887	0,781673	-2,03722503	0,041627	0,533659
SBP1	2534,031052	-1,754676846	0,8610369	-2,03786477	0,041563	0,533659
_C302020WA	511,8074159	1,575085153	0,7738288	2,035443884	0,041806	0,534334
_CR07230WA	1750,636477	-1,972669144	0,9707586	-2,03209041	0,042145	0,534334
CAF16	202,9570016	-1,567043376	0,7715728	-2,03097291	0,042258	0,534334
CMK1	459,0165936	-1,524442506	0,7500671	-2,03240811	0,042112	0,534334
PGA62	3626,303845	-1,335167329	0,6573174	-2,03123678	0,042231	0,534334
TRM2	82,21046755	1,91265388	0,9412247	2,032090679	0,042144	0,534334
_C202500WA	580,2739499	-1,868119705	0,9233546	-2,02318767	0,043054	0,542645
_CR08940WA	161,3335615	1,440830282	0,7123521	2,022637839	0,043111	0,542645
_C110240CA	22,16013067	-2,506969855	1,2426202	-2,01748677	0,043645	0,547965
_C504030WA	41,9548034	1,732810952	0,8592464	2,01666369	0,043731	0,547965
_C103750WA	58,13015101	-1,819023637	0,9064882	-2,00667107	0,044785	0,557313
_C305360CA	95,58694511	-1,492376626	0,7440227	-2,00582124	0,044875	0,557313
_C402570CA	29,00785697	-1,990125074	0,9921881	-2,0057942	0,044878	0,557313
TLO7	48,54033667	-2,080522925	1,0362833	-2,0076777	0,044678	0,557313
SWC4	575,798661	-1,643724173	0,8207412	-2,00273143	0,045206	0,560132
_C208950WA	18,58840622	-2,133239262	1,0667481	-1,99975919	0,045526	0,561685
TBF1	187,0983864	1,469248334	0,7347384	1,999689119	0,045534	0,561685
_C107790CA	100,5092574	1,598342673	0,8044995	1,986754075	0,04695	0,567066
_C202300WA	65,4302936	-2,125026177	1,0680351	-1,9896594	0,046628	0,567066
_C205840WA	67,09440424	1,857518954	0,9339474	1,988890422	0,046713	0,567066
_C601890CA	220,7192691	1,751320987	0,878668	1,993154456	0,046245	0,567066
_C602900CA	35,61455104	1,799219959	0,9059382	1,986029418	0,04703	0,567066
_C702370WA	100,440946	-1,804247677	0,9086021	-1,98574009	0,047062	0,567066
_CR09570WA	33,67575778	-2,005414111	1,0088306	-1,98786018	0,046827	0,567066
CST5	22,02289943	-2,178984228	1,0941569	-1,99147335	0,046429	0,567066
PRN2	27,10162742	1,838707289	0,9258409	1,985986177	0,047035	0,567066
PWP2	348,4582294	1,498420497	0,7546985	1,985455876	0,047094	0,567066
RCL1	199,2499004	1,6150261	0,8123255	1,98815142	0,046795	0,567066
_CR03710CA	41,26104903	-2,14432192	1,0855254	-1,97537706	0,048225	0,579435
_C406790WA	123,0402996	1,496812803	0,7581988	1,974169435	0,048362	0,579827
_C300400CA	57,4928096	-1,859430192	0,9426587	-1,97253806	0,048548	0,5808
_C702010CA	14,18779145	-2,543565311	1,2910469	-1,97015711	0,04882	0,5828
_C200170CA	276,0814709	1,385002498	0,703742	1,968054284	0,049062	0,583834
_CR01700CA	163,5645497	1,41095843	0,7171065	1,967571768	0,049117	0,583834
_C103660WA	31,180841	-2,047302689	1,0443125	-1,96043116	0,049945	0,588635
_C205530CA	436,717547	-1,628774647	0,8301424	-1,96204255	0,049758	0,588635
_CR07030CA	47,59744153	1,770214306	0,9025087	1,961437482	0,049828	0,588635
ZCF20	66,82075921	1,63101969	0,8318715	1,960663059	0,049918	0,588635
GAL7	167,1615504	-1,687876973	0,8614572	-1,95932773	0,050074	0,588905
_C210730WA	9,669558966	2,72150204	1,3928943	1,953846766	0,050719	0,591495
_C401220CA	28,07722075	-2,219637873	1,1360422	-1,95383407	0,050721	0,591495
_C503880CA	15,45412674	-3,024373534	1,5466141	-1,95548034	0,050526	0,591495
_C702520WA	10,53937203	-2,741373865	1,4030517	-1,9538652	0,050717	0,591495
_C203000CA	81,53628931	1,539699259	0,7907569	1,947120768	0,05152	0,598303
_CR04110WA	256,3982504	1,327756303	0,6816099	1,947971024	0,051418	0,598303
FTR2	350,3160531	1,358617743	0,699199	1,943105922	0,052003	0,601397
LEU2	111,1031412	-1,5807877	0,8131966	-1,94391821	0,051905	0,601397

_C400800WA	16,49192175	2,30172938	1,1854612	1,941632034	0,052182	0,602205
_C100540CA	130,0412789	1,499768626	0,7730311	1,940114108	0,052366	0,603076
_C503480CA	218,5558379	1,351978971	0,6977639	1,937587983	0,052674	0,604357
_C600730WA	9,355273427	2,795114136	1,4427039	1,937413651	0,052695	0,604357
_CR05770WA	28,54619498	-1,825554641	0,9456503	-1,93047535	0,053548	0,608925
CDR1	2072,196294	1,862186053	0,9640954	1,931537163	0,053417	0,608925
FGR13	46,76309367	-1,804292007	0,9349906	-1,92974346	0,053639	0,608925
POS5	130,2574194	-1,651234709	0,8543503	-1,93273739	0,053269	0,608925
RAD9	89,77456985	-1,600980903	0,8296444	-1,92971952	0,053642	0,608925
PRP5	163,7735398	1,432129259	0,7428488	1,927888009	0,053869	0,610259
_CR07200WA	115,0726741	1,396991694	0,7257696	1,924841921	0,054249	0,613314
ADAEC	469,4207457	-1,320095793	0,6915221	-1,90897129	0,056266	0,61588
ALP1	164,5597919	1,335065499	0,7002526	1,906548428	0,056579	0,61588
ALS1	976,4645067	-1,360139838	0,7113461	-1,91206471	0,055868	0,61588
ATG15	215,1059124	-1,639909729	0,8583928	-1,91044197	0,056076	0,61588
BUL1	113,7284165	1,415325563	0,7423876	1,906451046	0,056592	0,61588
_C105440CA	9,412354867	-2,561487059	1,3382852	-1,91400691	0,055619	0,61588
_C113720WA	210,0598492	1,51266667	0,7888663	1,917519642	0,055172	0,61588
_C305250CA	38,10164709	-1,682525145	0,8830491	-1,90535856	0,056733	0,61588
_C306560WA	86,39407695	-1,505851003	0,7873072	-1,9126601	0,055792	0,61588
_C307380WA	213,5557988	-1,774281717	0,9270637	-1,91387255	0,055636	0,61588
_C307820WA	395,9005811	-1,896370464	0,9868971	-1,92154838	0,054663	0,61588
_C504540CA	24,57648922	-1,860898009	0,9743254	-1,90993474	0,056142	0,61588
_C603600CA	22,05874678	2,448304552	1,2876337	1,901398366	0,05725	0,61588
_C700260CA	140,8748672	-1,720919853	0,900388	-1,91130923	0,055965	0,61588
_CR00980CA	559,1542242	-1,545958045	0,8088973	-1,91119191	0,05598	0,61588
_CR03600CA	178,2902046	-1,546454242	0,812547	-1,90321826	0,057012	0,61588
_CR06040WA	387,2903517	1,438551159	0,7540441	1,907781214	0,056419	0,61588
_CR09340WA	340,1279314	-1,620988035	0,8497602	-1,90758293	0,056445	0,61588
_CR09800CA	507,6530835	1,615386265	0,8449739	1,91175875	0,055907	0,61588
PDR16	95,10225876	1,708501037	0,8984879	1,901529291	0,057233	0,61588
RPT2	2959,999257	-1,781034762	0,9354048	-1,90402559	0,056907	0,61588
SOD1	477,4847106	1,310190183	0,6884277	1,90316303	0,057019	0,61588
TES15	33,8112195	-2,005082174	1,0452127	-1,91834848	0,055067	0,61588
THG1	49,7551591	1,626912398	0,851303	1,911085031	0,055994	0,61588
TPS3	438,816991	-1,615401456	0,8491484	-1,90237825	0,057122	0,61588
_C110980WA	5,294396698	-3,68848146	1,9449105	-1,89647881	0,057897	0,619952
_C500390CA	134,6074832	-1,358600541	0,7170822	-1,89462305	0,058142	0,619952
KRR1	310,6428153	1,305000306	0,6889134	1,894287943	0,058187	0,619952
NCE103	107,4097662	1,833705098	0,9669272	1,896425281	0,057904	0,619952
URA7	835,1338273	1,435467306	0,7574661	1,895091246	0,05808	0,619952
FAD3	119,9039168	1,471196283	0,7785994	1,889541959	0,058819	0,62549
_C109710CA	227,5263455	1,510533855	0,8000749	1,887990564	0,059027	0,625582
_CR06690CA	103,3233921	-1,58868725	0,8419305	-1,88695761	0,059166	0,625582
DBP7	276,7391202	1,653473804	0,8759564	1,88762121	0,059077	0,625582
_C202920WA	99,5163335	-1,360649494	0,7219555	-1,88467215	0,059474	0,626453
_C503120WA	72,60052178	-1,664304511	0,882823	-1,88520741	0,059402	0,626453
_C403710CA	6,533123402	-2,887446624	1,5369522	-1,87868343	0,060288	0,632622
_C701740CA	29,81719704	-1,671842067	0,8895939	-1,8793318	0,060199	0,632622
BUB3	117,5529338	-1,33194015	0,7143103	-1,86465205	0,06223	0,64202

_C110250CA	50,01286445	1,584554372	0,8467616	1,871311041	0,061302	0,64202
_C304920CA	102,6240739	-1,512312843	0,8105902	-1,86569336	0,062084	0,64202
_C402620CA	362,1514598	-1,602780239	0,8603002	-1,86304765	0,062456	0,64202
_C703350CA	81,82551932	1,433351719	0,7685615	1,864979874	0,062184	0,64202
_CR00270CA	35,7681845	-1,831147658	0,9811814	-1,86626818	0,062004	0,64202
DAL7	8,004209392	-2,853838476	1,53023	-1,8649736	0,062185	0,64202
GLR1	305,1957945	1,39056216	0,7443043	1,868271123	0,061724	0,64202
HGT4	79,06418649	-1,910196098	1,0221055	-1,86888355	0,061639	0,64202
NPL3	2636,502062	-1,500107574	0,8040722	-1,86563792	0,062092	0,64202
PPT2	16,86629257	-2,068033145	1,1095819	-1,86379501	0,06235	0,64202
BUD23	93,47194482	1,477292096	0,7944041	1,859622882	0,062939	0,643434
_C201420CA	57,92537824	1,51985989	0,817281	1,859653976	0,062934	0,643434
UTP5	319,161229	1,567526889	0,8429335	1,85960925	0,062941	0,643434
_C103150CA	62,64273302	-1,71884669	0,9247624	-1,85869009	0,063071	0,643541
_C209590CA	40,80084381	-1,661617833	0,8955262	-1,85546538	0,06353	0,643541
_C505360CA	205,7220421	-1,584211058	0,8533179	-1,85653086	0,063378	0,643541
RFC4	100,4951914	1,424349809	0,7667233	1,857710425	0,06321	0,643541
RGD3	76,56804099	-1,419715003	0,7651563	-1,85545742	0,063531	0,643541
_C206610CA	96,79365432	-1,581674598	0,8533062	-1,85358376	0,063799	0,645076
_C504910WA	79,44985978	1,697084215	0,9175994	1,84948266	0,064388	0,646711
_CR03230WA	300,5040671	-1,546921456	0,8357789	-1,85087399	0,064188	0,646711
_CR07820WA	7,983043023	-2,880909115	1,5585871	-1,84841075	0,064543	0,646711
OPY2	77,91232947	1,429282921	0,7729821	1,849050511	0,064451	0,646711
UTP18	398,8887998	1,599387695	0,8642749	1,850554288	0,064234	0,646711
_CR07170WA	122,3221395	-1,893124423	1,0254676	-1,84610841	0,064876	0,648882
_C503920CA	73,84297045	1,585836653	0,8600166	1,843960505	0,065189	0,649665
DRE2	195,1907067	1,279829152	0,6939943	1,844149331	0,065161	0,649665
_C407040WA	19,48926508	-2,135328682	1,1594987	-1,84159646	0,065534	0,649775
_C600710WA	64,14068744	-1,660590994	0,9017692	-1,84148126	0,065551	0,649775
SNQ2	411,2067909	1,627543005	0,8831402	1,842904417	0,065343	0,649775
SPL1	757,7089405	1,25145916	0,6811758	1,837204377	0,06618	0,654837
_C601040CA	203,8708146	1,288861552	0,7030249	1,83330848	0,066757	0,659057
PGA27	11,36494592	2,26422614	1,2354432	1,832723745	0,066844	0,659057
TFS1	217,2447061	-1,39821391	0,763826	-1,8305399	0,067169	0,661093
_C704140CA	155,462438	1,289102006	0,7073107	1,822539966	0,068373	0,671751
_C112070CA	31,79164668	1,734094364	0,9528357	1,81993012	0,06877	0,674453
ARG11	18,55263171	1,924075284	1,0733955	1,792512986	0,073051	0,674686
BAS1	384,9618302	1,226402151	0,6782309	1,808236875	0,07057	0,674686
_C109650WA	240,5000357	-1,692823429	0,9330139	-1,81436026	0,069622	0,674686
_C110140CA	77,49530936	-1,663465795	0,9233469	-1,80156111	0,071614	0,674686
_C110460WA	83,20875407	-1,40438231	0,7816337	-1,79672699	0,072379	0,674686
_C111160CA	77,04577894	1,371258666	0,7604474	1,80322628	0,071353	0,674686
_C208060WA	20,41402361	-1,954265567	1,0813126	-1,80730861	0,070714	0,674686
_C208100WA	80,96688624	-1,347404326	0,7523122	-1,79101758	0,07329	0,674686
_C209500WA	91,13290544	1,510866756	0,8356011	1,808119722	0,070588	0,674686
_C303770CA	79,61001963	-1,549211297	0,8601785	-1,80103469	0,071697	0,674686
_C406850CA	325,6768059	1,23971916	0,6891471	1,798918003	0,072032	0,674686
_C500090CA	365,2126057	-1,64260761	0,9110466	-1,80298973	0,07139	0,674686
_C501110WA	170,4696679	1,256072353	0,7012214	1,791263531	0,073251	0,674686
_C503490CA	153,1921274	-1,656061807	0,9142342	-1,81141961	0,070076	0,674686

_C504870WA	55,00334772	-1,497344647	0,8358567	-1,79138918	0,073231	0,674686
_C600120WA	66,94104923	-1,790300663	0,9876362	-1,81271268	0,069876	0,674686
_C700060CA	181,6859265	-1,648335886	0,908307	-1,81473431	0,069565	0,674686
_C703850WA	319,0414729	1,222472098	0,6775617	1,804222589	0,071196	0,674686
_CR04560CA	142,5012535	1,300351793	0,7208111	1,8040118	0,071229	0,674686
DOG1	122,6155466	-1,412519986	0,7840804	-1,80149881	0,071624	0,674686
ENA2	702,5231319	-1,223788226	0,6779923	-1,80501795	0,071072	0,674686
ERG1	525,0426215	-1,253761312	0,6985332	-1,79484866	0,072678	0,674686
FAD1	88,07138227	-1,561294903	0,8635477	-1,80800077	0,070606	0,674686
FET31	895,4648285	1,406812964	0,7849129	1,792317299	0,073082	0,674686
GCA1	5,680798262	-3,083511218	1,7194536	-1,79330873	0,072924	0,674686
GLX3	275,7497996	1,296564493	0,7163108	1,810058463	0,070287	0,674686
JIP5	178,9092353	1,53448344	0,8547715	1,795197265	0,072622	0,674686
KSP1	151,0361721	-1,399395224	0,774436	-1,80698629	0,070764	0,674686
MVB12	92,04309303	-1,635767501	0,9095588	-1,79841862	0,072111	0,674686
PGA5	27,96800327	-1,969997807	1,0898543	-1,80757902	0,070672	0,674686
PHHB	43,57929907	-1,606010241	0,8867013	-1,81121894	0,070107	0,674686
RIB4	239,990792	-1,577647114	0,8731574	-1,80683021	0,070789	0,674686
SAP99	13,4735735	-2,137003282	1,1901683	-1,79554713	0,072567	0,674686
SRP40	306,256291	1,568825116	0,874659	1,793642046	0,07287	0,674686
TLG2	44,35001532	-1,613557503	0,8972294	-1,79837789	0,072117	0,674686
TPO5	106,3887059	-1,583006163	0,8717648	-1,81586377	0,069391	0,674686
TSR2	192,326738	1,254573697	0,6956178	1,803538684	0,071304	0,674686
FGR47	161,8801953	-1,56575607	0,8747959	-1,78985295	0,073478	0,675288
PRM1	5,978831357	-3,203686982	1,7928645	-1,78690971	0,073952	0,678526
GSG1	59,05289878	-1,584377861	0,8894043	-1,78139208	0,074848	0,684487
PST2	48,94652528	1,715105542	0,962518	1,781894462	0,074766	0,684487
_C110510WA	32,02922191	-1,894003527	1,063989	-1,78009699	0,07506	0,68489
TRM1	76,74367396	1,698934124	0,9546647	1,779613448	0,075139	0,68489
DAC1	60,68761367	-1,67272541	0,9409453	-1,77770747	0,075452	0,686612
IFM1	186,6139699	1,486643269	0,8374641	1,775172535	0,075869	0,689282
MSS116	356,2262101	1,542382081	0,869309	1,774262218	0,07602	0,689519
GUA1	1707,87349	1,3869651	0,782778	1,771849902	0,076419	0,690887
PHO4	226,9621112	-1,354868196	0,7646164	-1,771958	0,076402	0,690887
ATO6	18,49456524	-1,974996682	1,1193247	-1,76445375	0,077656	0,691599
_C100420WA	132,6141279	-1,270014941	0,7181173	-1,76853406	0,076972	0,691599
_C112440WA	28,59814137	1,612003182	0,9124216	1,766730546	0,077273	0,691599
ERG5	625,2062929	1,341358351	0,758263	1,768988348	0,076896	0,691599
GLK4	58,10858407	-1,480034602	0,836201	-1,76995068	0,076735	0,691599
MCM3	251,2421012	1,213658856	0,6871751	1,766156586	0,07737	0,691599
MIS12	1618,474819	1,388280516	0,7870393	1,763927727	0,077744	0,691599
RGT1	36,85374472	-1,496336607	0,8477244	-1,76512161	0,077543	0,691599
SGD1	397,3621535	1,246087404	0,7048652	1,767837777	0,077088	0,691599
TLO1	370,8181378	-1,634710386	0,9261206	-1,76511614	0,077544	0,691599
_C104710CA	45,91131791	1,740050405	0,9875056	1,762066363	0,078058	0,693222
HSP21	10,80673996	-2,119589806	1,2033778	-1,76136691	0,078176	0,693222
_C102040CA	342,1446634	-1,518054575	0,8625283	-1,76000554	0,078407	0,694018
_C210130WA	17,04150121	-1,871952843	1,0667471	-1,75482352	0,079289	0,694018
_C602790CA	33,1869802	-1,534051132	0,8744877	-1,75422843	0,079391	0,694018
_C701650WA	432,4359002	-1,382502165	0,7878419	-1,75479653	0,079294	0,694018

_C702160WA	24,01312598	1,961158807	1,117553	1,754868722	0,079282	0,694018
_C704280CA	140,4498944	-1,458335306	0,8313002	-1,75428238	0,079382	0,694018
CAR2	897,2674864	-1,291120924	0,7345357	-1,75773744	0,078792	0,694018
MLT1	532,7312192	1,390981982	0,7922357	1,75576795	0,079128	0,694018
WOR3	35,01830541	-1,486601755	0,8454313	-1,75839459	0,07868	0,694018
_C503030WA	297,2395094	-1,661143405	0,9482342	-1,75182827	0,079803	0,696523
_C209980WA	107,1649648	-1,41586408	0,8094113	-1,74925165	0,080248	0,6993
_C702190WA	5,76611015	-2,724247098	1,5588639	-1,74758493	0,080536	0,700713
_C305140CA	139,1002184	1,412030474	0,8087355	1,74597315	0,080816	0,702046
LEU1	368,2892285	-1,169260092	0,6702413	-1,74453597	0,081066	0,703118
_C112140WA	28,22731881	-1,906465286	1,0934952	-1,74346007	0,081253	0,703646
_C703030WA	33,64050198	-1,715797629	0,9896686	-1,7337092	0,08297	0,712949
_CR03730CA	43,36489616	-1,477299858	0,8508584	-1,73624646	0,08252	0,712949
_CR10410CA	161,7146929	1,393831389	0,8037763	1,73410358	0,0829	0,712949
SCR1	154,1714391	-1,561489007	0,8999632	-1,73505869	0,08273	0,712949
YAE1	36,19570787	-1,586887199	0,9150515	-1,73420528	0,082882	0,712949
INO2	45,47704089	1,515733429	0,8764849	1,729332052	0,08375	0,718539
NTH1	96,92804074	-1,264091898	0,7315886	-1,72787251	0,084011	0,719669
_C400910CA	42,70588945	-1,413557092	0,8191663	-1,72560456	0,084419	0,722045
ALS7	68,57703339	-1,34528537	0,7803307	-1,72399394	0,084709	0,723414
_C106400CA	323,0135389	-1,43433533	0,8341938	-1,7194269	0,085537	0,723763
_C400050WA	96,06269553	-1,389792642	0,8084255	-1,71913501	0,08559	0,723763
_C602880WA	156,7555596	1,23640476	0,7196682	1,718020567	0,085793	0,723763
_C603440WA	89,54020593	1,551782319	0,9015135	1,721307968	0,085195	0,723763
_C700160CA	260,4736193	1,172719279	0,6820648	1,719366317	0,085548	0,723763
FGR38	75,10501349	-1,562686864	0,9071841	-1,72256856	0,084967	0,723763
MSN4	110,115895	-1,260589398	0,7335008	-1,7185931	0,085688	0,723763
SSZ1	2055,176628	1,167560308	0,6795216	1,718209192	0,085758	0,723763
_C108240CA	21,98523346	-1,715228104	1,0006237	-1,71415897	0,0865	0,727799
_C302790WA	23,97713114	-2,045514408	1,1944273	-1,71254823	0,086796	0,727799
_C602290CA	82,87123902	1,53733863	0,8970002	1,7138666	0,086553	0,727799
ECM1	139,0522446	1,218653792	0,7113323	1,713198933	0,086676	0,727799
_C401500WA	39,24912226	1,477510175	0,8635796	1,71091378	0,087097	0,729224
GCD6	465,7424349	1,257675439	0,7358805	1,709075747	0,087437	0,730967
_CR06650CA	5,192180027	-2,943097425	1,7240301	-1,70710324	0,087803	0,732923
_C114500CA	1044,569064	-1,475884593	0,8655619	-1,70511733	0,088173	0,7336
_C200280CA	35,88763865	1,455989947	0,8548934	1,70312457	0,088545	0,7336
_C604120CA	117,7901241	-1,2759427	0,7489637	-1,70361076	0,088454	0,7336
_CR04750WA	39,12403529	1,565124884	0,9179497	1,705022525	0,08819	0,7336
NOP13	196,4256459	1,529544333	0,8973847	1,704446574	0,088298	0,7336
GPM1	3751,403832	-1,276961003	0,7513727	-1,69950402	0,089224	0,73703
UTP9	334,8034346	1,546826788	0,9099356	1,699929868	0,089144	0,73703
ABP2	6,983670485	-2,392826391	1,4087297	-1,69857027	0,0894	0,737267
_C704290WA	96,03478145	-1,612650951	0,9497677	-1,69794253	0,089519	0,737267
_C201900CA	90,0793449	-1,337265593	0,7879412	-1,69716417	0,089666	0,737384
PHO86	83,74462933	-1,251689008	0,7379805	-1,69610027	0,089867	0,737945
ATO2	163,5063694	-1,492616717	0,883881	-1,68870778	0,091275	0,744233
_C114350WA	364,4368047	-1,565356142	0,9276824	-1,68738376	0,09153	0,744233
_C200740CA	219,2654666	-1,351210999	0,8020556	-1,68468495	0,092049	0,744233
_C202150CA	136,9647689	-1,354047965	0,802581	-1,68711682	0,091581	0,744233

_C204300CA	37,78743768	-1,48879243	0,8835504	-1,68501134	0,091986	0,744233
_C307850WA	198,0415398	-1,461383717	0,8692605	-1,68118037	0,092728	0,744233
_C400950CA	27,45636116	-1,513878521	0,8967505	-1,68818248	0,091376	0,744233
_C700880CA	73,30786878	-1,28565109	0,7651619	-1,68023402	0,092912	0,744233
DRS1	582,4002422	1,118697735	0,6655891	1,680763299	0,092809	0,744233
FAR1	8,498058911	-2,667881778	1,5860117	-1,68213245	0,092543	0,744233
FMA1	211,6467547	-1,30571184	0,7742491	-1,68642352	0,091714	0,744233
GLE1	583,6013372	-1,527613083	0,9090022	-1,68053844	0,092853	0,744233
PLB4.5	54,60100514	-1,327002719	0,7854243	-1,68953612	0,091117	0,744233
SAP10	32,97458821	-1,865441321	1,1099129	-1,68070968	0,092819	0,744233
SAP30	6,85755415	-2,594458762	1,5400241	-1,68468713	0,092049	0,744233
THI4	15,86362814	-2,224794906	1,316956	-1,68934645	0,091153	0,744233
ZCF38	81,27363016	-1,619511771	0,96257	-1,68248733	0,092474	0,744233
_CR02470WA	161,7217026	-1,261166771	0,7520094	-1,67706254	0,09353	0,747031
PEX5	483,0445365	-1,289114151	0,7683618	-1,67774368	0,093397	0,747031
KIS2	81,56905966	-1,247228229	0,7443536	-1,67558578	0,093819	0,748263
_C101910WA	255,2967557	1,247673493	0,7450481	1,674621441	0,094009	0,748696
_C101160CA	71,26251801	1,526694934	0,919681	1,660026658	0,096909	0,755537
_C205770WA	27,13780827	-1,634107539	0,9791935	-1,66883001	0,095151	0,755537
_C205830CA	40,21114618	1,65343401	0,9943366	1,662851377	0,096342	0,755537
_C305160CA	480,1823667	1,505552071	0,9065413	1,660765097	0,096761	0,755537
_C503570WA	185,4516686	-1,453303589	0,8731121	-1,66450966	0,096011	0,755537
_C704160WA	32,06085085	-1,795784303	1,0800801	-1,66263996	0,096385	0,755537
_CR05470CA	29,70331872	-2,20562904	1,3244985	-1,66525603	0,095862	0,755537
_CR07080WA	336,7555513	1,254761927	0,7524522	1,667563671	0,095402	0,755537
CAN3	57,66132295	-1,282474354	0,7701644	-1,66519567	0,095874	0,755537
CTR1	462,4407309	1,236809729	0,7446244	1,660984608	0,096717	0,755537
JEN2	44,9211665	-7,940607882	4,7823975	-1,66038225	0,096838	0,755537
LAP3	21,75166917	-1,743373453	1,0481767	-1,66324383	0,096264	0,755537
MUP1	580,5489451	1,105556249	0,6641363	1,664652758	0,095982	0,755537
PWP1	454,6579415	1,310159724	0,7858325	1,667225078	0,09547	0,755537
SER2	289,8518618	1,166249573	0,7013597	1,662840872	0,096344	0,755537
_C703280CA	29,17647592	-1,875585441	1,1305331	-1,6590275	0,09711	0,755881
_CR05680CA	10,65784115	-2,143026595	1,2921821	-1,6584556	0,097226	0,755881
CSA1	170,352158	1,156185697	0,6976499	1,657257708	0,097467	0,756701
_C400580WA	45,03059357	-1,543878182	0,9321852	-1,65619259	0,097683	0,757315
_C104040CA	267,5831331	1,429616102	0,8671329	1,648670189	0,099215	0,759594
_C105270CA	204,6348988	1,187637715	0,7204886	1,648378211	0,099275	0,759594
_C113500CA	22,3841904	-1,900936955	1,1524488	-1,64947623	0,09905	0,759594
_C202390WA	50,7459836	-1,399770798	0,8500439	-1,64670405	0,099619	0,759594
_C207270WA	211,3536954	-1,323495306	0,8032065	-1,64776466	0,099401	0,759594
_C209760WA	175,9718119	-1,585989912	0,9629166	-1,64706878	0,099544	0,759594
_C301560WA	302,5398028	1,300608075	0,7889115	1,64861089	0,099227	0,759594
_CR04160CA	176,1479506	1,239439153	0,7500517	1,652471516	0,098438	0,759594
ENP2	200,5134906	1,652167744	1,0029585	1,647294269	0,099498	0,759594
HPA2	66,11867132	-1,681875515	1,0169774	-1,65379834	0,098168	0,759594
MAC1	35,15781467	1,441446582	0,8744548	1,64839463	0,099272	0,759594
MSO1	84,66762442	-1,33417244	0,8072995	-1,65263636	0,098405	0,759594
HHO1	354,7106335	-1,108374874	0,6740539	-1,6443416	0,100106	0,762259
_CR00600CA	38,31242298	-1,410549668	0,8586904	-1,64267545	0,10045	0,763834

_C102940CA	130,0846138	-1,381936338	0,8420443	-1,64116818	0,100763	0,765161
_C304510WA	58,95837286	1,639306721	1,001096	1,637512088	0,101524	0,766744
_C704340CA	38,6523182	-1,626604063	0,9932222	-1,6377041	0,101483	0,766744
PGK1	3443,771889	-1,186908847	0,7241671	-1,63899854	0,101214	0,766744
RRP8	209,1817646	1,206265642	0,7365157	1,637800417	0,101463	0,766744
_C108040WA	19,66117591	-1,669852654	1,0212224	-1,63515087	0,102017	0,767573
_C401430CA	34,58803705	-1,393788486	0,8526907	-1,63457679	0,102138	0,767573
_C504470CA	89,65320826	-1,59780953	0,9777318	-1,63420028	0,102217	0,767573
_C601750CA	36,93881553	-1,447921553	0,8862908	-1,63368685	0,102325	0,767573
FCR1	299,6464873	-1,366700441	0,8353712	-1,63603972	0,101831	0,767573
_C301950CA	73,65323059	-1,32884594	0,8140564	-1,63237569	0,1026	0,768603
_CR06530WA	105,5307433	1,220237172	0,7485066	1,630229051	0,103053	0,769916
_CR08990CA	445,1211257	-1,430630211	0,8773997	-1,63053418	0,102989	0,769916
_C109240CA	17,49945428	-1,635734618	1,0039094	-1,62936476	0,103236	0,770245
_C307550CA	74,91156754	1,240099563	0,7617351	1,62799329	0,103526	0,770341
HST1	209,90531	-1,35879765	0,8345223	-1,62823414	0,103475	0,770341
_CR09930WA	24,72602868	-1,634213037	1,0045233	-1,62685424	0,103768	0,771106
_C407240WA	321,6968585	-1,516926936	0,933592	-1,62482863	0,104199	0,773274
AUT7	75,80505418	-1,491376618	0,9224431	-1,61676809	0,105928	0,773457
_C101510WA	10,53510452	-1,90395003	1,1790802	-1,61477574	0,106359	0,773457
_C107940WA	31,7977445	-1,850153551	1,1401768	-1,62269003	0,104656	0,773457
_C111850WA	327,1789869	-1,197851378	0,7415482	-1,61533845	0,106237	0,773457
_C206480WA	76,58998411	1,263304388	0,7827238	1,613984844	0,106531	0,773457
_C209810CA	21,2021973	-1,539108959	0,9505447	-1,61918634	0,105407	0,773457
_C304350CA	12,81706479	-2,027001244	1,249116	-1,62274854	0,104643	0,773457
_C401590CA	7,219086352	-2,625814365	1,6202806	-1,62059231	0,105105	0,773457
_C504690CA	11,42855739	-2,127834252	1,3153782	-1,61765961	0,105736	0,773457
_C601950CA	140,3097334	1,142172032	0,7057731	1,618327539	0,105592	0,773457
CAR1	318,1613287	-1,28607172	0,7946982	-1,61831459	0,105595	0,773457
CTF5	14,63865589	-1,728253698	1,0673348	-1,61922363	0,105399	0,773457
DDR48	98,72060666	-1,642347568	1,0168798	-1,61508523	0,106292	0,773457
MET16	103,9123207	1,183703686	0,7335336	1,613700629	0,106592	0,773457
RIB3	172,6821441	-1,188360001	0,7350003	-1,61681564	0,105918	0,773457
RVS162	34,89862553	-1,538562766	0,9486061	-1,62191946	0,104821	0,773457
SPT7	311,9268165	-1,135176789	0,702509	-1,61588922	0,106118	0,773457
_C406280CA	14,61531482	-1,793293997	1,1130068	-1,61121568	0,107133	0,776363
_C203970WA	39,91534974	-1,388165556	0,8623392	-1,60976743	0,107449	0,777451
NAP1	1318,767146	-1,261951238	0,784189	-1,60924372	0,107563	0,777451
CDC43	16,22089283	-1,744965787	1,0852827	-1,6078445	0,107869	0,77865
_C301430WA	36,13696239	1,422120724	0,8860053	1,605092855	0,108473	0,781994
_C302920WA	121,3150729	-1,320541959	0,8252983	-1,60007829	0,109581	0,787934
NOP14	255,3042168	1,193794567	0,7458818	1,600514411	0,109485	0,787934
BRF1	109,8881599	1,166877482	0,7330541	1,591802752	0,111429	0,788433
_C112570CA	58,21013342	1,344375761	0,8428374	1,595059362	0,110699	0,788433
_C207640WA	136,5013255	-1,159278734	0,7273308	-1,59388109	0,110963	0,788433
_C402150CA	9,103617971	-2,089377201	1,3118037	-1,59275143	0,111216	0,788433
_C402770CA	51,71413486	1,369434265	0,8604675	1,591500306	0,111497	0,788433
_C406860CA	66,10884347	-1,496167916	0,9389658	-1,59342109	0,111066	0,788433
_C407140WA	88,1798891	1,280285391	0,8007576	1,598842621	0,109856	0,788433
_C407200CA	173,7513164	-1,321840618	0,8299192	-1,59273414	0,11122	0,788433

_CR09460CA	33,27646586	-1,393351434	0,8732525	-1,59558829	0,110581	0,788433
FRE10	578,1687647	1,368807289	0,8584287	1,594549781	0,110813	0,788433
PDK2	158,0592994	-1,376118999	0,8614979	-1,5973562	0,110186	0,788433
PES1	1898,40356	1,04418065	0,6550472	1,594053943	0,110924	0,788433
SRD1	48,55979779	-1,277569185	0,8010037	-1,59496044	0,110721	0,788433
_C305300CA	23,1698661	-1,471584327	0,9256059	-1,58986057	0,111866	0,789226
_CR09750CA	73,13909659	-1,34091635	0,8441777	-1,58842909	0,112189	0,789226
NOP15	126,0455537	1,48781775	0,9370009	1,587851007	0,11232	0,789226
PTC8	67,86578981	-1,253207433	0,7883835	-1,58959115	0,111927	0,789226
SPB1	412,8491469	1,303821021	0,8210976	1,587900226	0,112309	0,789226
_C110500WA	49,70992072	-1,378623212	0,8705827	-1,58356368	0,113293	0,791071
_C208420WA	33,58910858	1,371907392	0,8660258	1,584141494	0,113162	0,791071
_C208720WA	187,6657568	-1,30885041	0,826271	-1,58404494	0,113183	0,791071
IFM3	11,98808072	-1,92412437	1,2149736	-1,58367585	0,113268	0,791071
MED8	36,05944633	-1,492993633	0,9428116	-1,58355464	0,113295	0,791071
_C502620CA	350,4018855	-1,337150299	0,8450735	-1,58228883	0,113584	0,791095
ECM17	675,106116	1,168775292	0,7384193	1,582807031	0,113465	0,791095
RCT1	1677,130447	-1,408433389	0,8907335	-1,58120623	0,113831	0,791824
_C112810WA	133,2058391	-1,307888639	0,8288005	-1,57805005	0,114554	0,793225
LYS142	47,4690002	-1,253156262	0,794226	-1,57783344	0,114604	0,793225
PGA58	11,55773031	-1,965066132	1,2452142	-1,57809487	0,114544	0,793225
PHR1	870,4412418	1,104137052	0,6994435	1,578593697	0,114429	0,793225
_C106650WA	116,5142961	-1,315814522	0,8354701	-1,57493916	0,11527	0,794865
_C305510WA	28,51617046	-1,463698162	0,9290731	-1,57543918	0,115155	0,794865
HSP104	583,9711436	-1,04322679	0,6622901	-1,57518099	0,115215	0,794865
_C107960WA	43,42042264	1,498326474	0,95286	1,572451932	0,115846	0,795352
_C301140WA	93,50041287	-1,5877492	1,0107117	-1,57092188	0,116201	0,795352
_C301260CA	42,32865747	-1,29918194	0,8268179	-1,57130366	0,116112	0,795352
_C406240WA	34,83053562	-1,319553963	0,8394205	-1,57198198	0,115955	0,795352
_C504900CA	165,041361	1,267214085	0,8062009	1,571834122	0,115989	0,795352
RPP1	23,52081644	1,515831215	0,9633295	1,573533519	0,115595	0,795352
CYB2	59,33781214	-1,48193785	0,9439864	-1,56987202	0,116445	0,796041
_CR09700WA	20,08935162	-1,706461101	1,0887835	-1,56730985	0,117042	0,79718
MNN12	23,72568644	-1,452810783	0,9267567	-1,56762908	0,116968	0,79718
PRB1	95,27982332	-1,239809963	0,7905315	-1,56832449	0,116805	0,79718
ECM15	161,6553825	-1,308185994	0,836009	-1,56479903	0,11763	0,800201
_C114080WA	606,7649817	1,288677329	0,8252464	1,561566675	0,11839	0,801443
_C601360WA	11,19217183	-1,880153656	1,2032495	-1,56256339	0,118155	0,801443
MOH1	5,19588302	-2,552902472	1,6330264	-1,56329529	0,117983	0,801443
SSN6	1607,960284	-1,129031875	0,7227379	-1,56215959	0,11825	0,801443
DIP2	337,8787307	1,405294187	0,9006155	1,560370827	0,118672	0,802375
CTA2	618,7006864	-1,496925638	0,9604712	-1,55853257	0,119107	0,802382
HTB1	1334,563487	-1,056019042	0,6776902	-1,55826224	0,119171	0,802382
PHO15	197,5877951	-1,08056784	0,6935953	-1,55792269	0,119252	0,802382
UFE1	262,3628019	-1,34336195	0,8622241	-1,55801948	0,119229	0,802382
_CR02510WA	221,1137282	-1,233539068	0,7925258	-1,55646541	0,119597	0,802764
SFU1	132,3323116	1,177792205	0,7567092	1,556466117	0,119597	0,802764
HOS3	109,3345677	-1,131425921	0,7293216	-1,55134014	0,12082	0,809014
NOP4	1233,456708	1,022246331	0,6589254	1,551384013	0,12081	0,809014
RPC53	97,19942954	1,595418563	1,0305992	1,5480495	0,12161	0,813324

_C603880WA	7,864891267	-2,278218743	1,4723475	-1,54733768	0,121782	0,813491
_C104850CA	19,06237643	-1,753319859	1,1349057	-1,54490359	0,12237	0,813495
_C105480CA	8,420711602	-2,136739454	1,3833014	-1,54466661	0,122427	0,813495
_C701210CA	68,78466058	1,200090545	0,7761795	1,546150814	0,122068	0,813495
_CR09610CA	122,8416993	-1,444624213	0,9358211	-1,54369703	0,122662	0,813495
MPP10	1246,180246	1,064025621	0,6884447	1,54554995	0,122213	0,813495
NOT4	632,2652722	-1,270862743	0,823056	-1,54407807	0,122569	0,813495
RRS1	126,8960988	1,386281409	0,898941	1,542127284	0,123043	0,815048
PRA1	7,351403669	-2,412419486	1,5652945	-1,54119205	0,12327	0,815581
_C405010WA	151,7833088	1,406897238	0,9138214	1,539575702	0,123664	0,81624
SUT1	25,47573757	-1,796475684	1,1667112	-1,53977754	0,123615	0,81624
_C407130WA	399,5632812	1,060608167	0,6893189	1,538632039	0,123894	0,816789
PEX17	45,55228121	-1,471828459	0,9572851	-1,53750267	0,12417	0,816941
PGA56	2498,768412	-1,065478859	0,6930692	-1,53733394	0,124212	0,816941
_C202710CA	151,1436521	1,174554393	0,765903	1,533554994	0,125139	0,818697
_C502640WA	17,23031683	-1,565982348	1,0212713	-1,5333657	0,125186	0,818697
HTA1	815,7684196	-1,102816145	0,7192703	-1,53324291	0,125216	0,818697
KRE30	1377,410438	1,300488547	0,8480534	1,533498483	0,125153	0,818697
tP(AGG)1	11,53600535	-2,180150908	1,4216347	-1,53355217	0,12514	0,818697
_C106970CA	133,8417445	-1,099411932	0,7177487	-1,53175041	0,125584	0,820138
FET33	50,17359594	1,249814666	0,8163925	1,530899157	0,125794	0,820546
TSA1B	14,50883656	-1,885768499	1,2349309	-1,52702353	0,126755	0,825843
_CR03200CA	88,33754655	1,322683526	0,8666268	1,526243492	0,126949	0,826137
_C700520WA	1606,14468	-1,19998874	0,7866324	-1,5254759	0,12714	0,826413
_C100100CA	360,1866627	-1,356339048	0,8923273	-1,5200018	0,128511	0,833141
_C406210CA	1010,491637	0,99889594	0,6571256	1,520099063	0,128486	0,833141
_CR10590WA	87,36269534	-1,166186434	0,7680613	-1,51835083	0,128926	0,833141
HAL9	66,91044817	-1,175977909	0,7741181	-1,51911955	0,128732	0,833141
PGA23	11,51483898	-2,195896644	1,4457568	-1,51885617	0,128799	0,833141
TPK2	219,8195359	-1,064749898	0,7021461	-1,5164221	0,129413	0,835314
_CR08310CA	9,994014263	-2,491516479	1,6446429	-1,51492851	0,129791	0,836779
_CR10500CA	43,45181839	-1,381757448	0,913759	-1,51216844	0,130491	0,838375
CHS5	907,1565484	-1,261936754	0,8341256	-1,51288573	0,130309	0,838375
RTS1	384,1221381	-1,023911691	0,6769575	-1,51251997	0,130402	0,838375
BRO1	125,6669888	-1,18415202	0,7863822	-1,50582253	0,132113	0,841813
_C104110WA	12,18607185	-1,741211158	1,1550018	-1,50753972	0,131672	0,841813
_C200270CA	161,3749125	-1,4359463	0,9518264	-1,508622	0,131395	0,841813
_C300420WA	181,1714238	1,12087874	0,7439334	1,506692278	0,13189	0,841813
_C404440WA	499,7971574	-1,308474126	0,867976	-1,50750032	0,131682	0,841813
_C602230WA	227,295286	1,391962549	0,9258207	1,503490346	0,132713	0,841813
_CR01430WA	25,64816991	1,388714092	0,9203615	1,508878951	0,13133	0,841813
CHR1	112,0868377	1,447010929	0,9627465	1,503003074	0,132838	0,841813
FET3	24,97190695	-1,406781457	0,9344211	-1,50551118	0,132193	0,841813
INO4	41,05644465	-1,323116761	0,8793474	-1,50465759	0,132412	0,841813
RAD59	25,57822859	1,472014362	0,9797848	1,502385337	0,132998	0,841813
RIX7	172,8192365	1,111989681	0,7387801	1,505170022	0,13228	0,841813
RRN11	24,80284558	1,423794143	0,9474437	1,502774377	0,132897	0,841813
_CR02550CA	56,68923258	1,226407985	0,8168748	1,501341513	0,133267	0,842559
SPB4	109,7175197	1,106431378	0,7373929	1,500463929	0,133494	0,843034
HRR25	342,1079595	-1,060470884	0,7076176	-1,49864974	0,133965	0,844597

PKH3	195,7644941	-1,037799089	0,6926346	-1,4983356	0,134046	0,844597
CCC2	102,1902784	1,172769854	0,7834853	1,496862578	0,134429	0,846049
_C301940CA	67,35094412	-1,378903985	0,9226906	-1,49443816	0,135061	0,849065
_C109330WA	51,41118784	1,49625498	1,0019085	1,493404785	0,135331	0,849801
_CR06290CA	128,3566107	-1,282765727	0,8594331	-1,49257197	0,135549	0,850208
_C602560WA	231,9734265	-1,202446725	0,8064105	-1,49110987	0,135933	0,85165
MCM6	242,3821129	1,120743322	0,7530273	1,488317023	0,136667	0,855288
_C602200CA	7,695472495	-2,237165123	1,5050203	-1,4864684	0,137155	0,857375
_C106490CA	6,862785434	-2,292316452	1,5457195	-1,48300936	0,138072	0,857685
_C210540WA	76,95614088	-1,46485353	0,9879078	-1,4827836	0,138132	0,857685
_CR09500CA	36,52592257	-1,24648012	0,840204	-1,48354464	0,13793	0,857685
DBP3	375,3045602	1,04521465	0,7042479	1,484157252	0,137767	0,857685
IFE1	10,17445419	-1,897411254	1,2793888	-1,48306069	0,138058	0,857685
PZF1	26,57977583	1,356931712	0,9135389	1,485357336	0,137449	0,857685
_C300340WA	62,22251151	1,258621295	0,8521764	1,476949214	0,139689	0,863664
_C502220CA	26,34198143	-1,466246709	0,9917362	-1,47846452	0,139283	0,863664
_CR10040WA	60,33379312	-1,28638448	0,8710357	-1,47684464	0,139717	0,863664
ETR1	607,2400382	-1,233786566	0,8349217	-1,47772729	0,139481	0,863664
_CR08550WA	196,1521292	-1,111435021	0,7528991	-1,47620706	0,139888	0,86376
_C101710WA	99,17716081	-1,158440782	0,7941489	-1,45871988	0,144642	0,866137
_C102450CA	56,10940715	1,593417892	1,0914916	1,45985357	0,14433	0,866137
_C104430CA	35,21767158	-1,442011071	0,9810029	-1,46993564	0,141579	0,866137
_C201390WA	233,7446905	-1,002005793	0,6849313	-1,46292892	0,143487	0,866137
_C201960CA	13,70472186	-1,691711447	1,1559498	-1,46348177	0,143336	0,866137
_C300310CA	38,2656754	-1,512170543	1,0262656	-1,47346903	0,140625	0,866137
_C400030CA	146,3260313	-1,309140726	0,8971672	-1,45919378	0,144512	0,866137
_C503470CA	79,87213882	-1,298376307	0,8808928	-1,47393226	0,1405	0,866137
_C503530CA	14,49689728	1,780350335	1,2156928	1,464473889	0,143065	0,866137
_C600180CA	18,74100226	-1,782603723	1,2152161	-1,46690263	0,142403	0,866137
_C700200WA	125,9368666	-1,308925738	0,8969827	-1,45925424	0,144495	0,866137
_C701940CA	235,1000588	-1,169348296	0,798442	-1,46453749	0,143047	0,866137
_C703380WA	39,36390008	1,235509446	0,8430181	1,465578764	0,142763	0,866137
_CR01510CA	12,33367784	-1,827292337	1,245298	-1,46735343	0,14228	0,866137
_CR02930WA	1163,843737	-1,16244908	0,7956911	-1,46093011	0,144035	0,866137
_CR06980WA	9,151081318	2,024825963	1,3803077	1,466938109	0,142393	0,866137
CDC46	171,6815272	1,08494807	0,7403499	1,465453027	0,142797	0,866137
GLC3	150,3155866	-1,023446961	0,7010612	-1,45985405	0,14433	0,866137
IST2	102,5159665	-1,080103704	0,7386606	-1,46224625	0,143674	0,866137
PEL1	147,7652813	1,03844277	0,7102698	1,46203997	0,14373	0,866137
PET127	116,4043375	1,34243681	0,9126458	1,470928557	0,14131	0,866137
POX1	74,19964011	-1,347288935	0,9147654	-1,47282454	0,140798	0,866137
PSP1	190,0495091	-1,005702691	0,6893424	-1,4589305	0,144584	0,866137
QCR7	553,571758	-1,105735525	0,7568972	-1,4608793	0,144049	0,866137
RPO41	603,8242631	1,408505752	0,9598726	1,467388161	0,14227	0,866137
SUA72	14,13192787	-1,629946423	1,1086101	-1,47026126	0,141491	0,866137
VTC4	373,2480787	-1,10568603	0,7529855	-1,46840283	0,141995	0,866137
ZCF24	23,21719482	-1,557752517	1,0639098	-1,46417722	0,143146	0,866137
HSX11	21,05078071	1,419303778	0,974325	1,456704735	0,145198	0,868528
ALS6	27,59149383	-1,284614343	0,889099	-1,4448496	0,1485	0,87341
ATP17	1074,326623	-1,154308945	0,7964296	-1,44935463	0,147239	0,87341

_C103510CA	447,658804	-1,26902726	0,8742456	-1,45156834	0,146622	0,87341
_C106760CA	134,2235184	1,532156025	1,0583147	1,447731932	0,147692	0,87341
_C109390WA	18,74345808	1,559957346	1,0789743	1,445778046	0,148239	0,87341
_C110440WA	131,0304265	1,068904269	0,7396775	1,445095017	0,148431	0,87341
_C201070WA	251,8294367	1,065286189	0,73468	1,450000204	0,147058	0,87341
_C203760CA	230,9555316	-1,01559257	0,7018396	-1,44704375	0,147885	0,87341
_C404810CA	99,48108991	1,249709061	0,8623156	1,449247814	0,147268	0,87341
_C406140CA	16,55175987	-1,482554779	1,0230814	-1,4491073	0,147308	0,87341
_C504050WA	112,6549893	-1,063906617	0,7326198	-1,45219479	0,146447	0,87341
_C700120WA	221,6459233	-1,331234223	0,9192964	-1,44810118	0,147589	0,87341
_C703990CA	65,68901661	1,169461929	0,8064697	1,450100188	0,147031	0,87341
_CR05480WA	237,5963386	-1,083589449	0,7500252	-1,44473748	0,148532	0,87341
NGS1	120,1619265	1,053901145	0,7283734	1,446924359	0,147918	0,87341
SOF1	169,869717	1,351450311	0,9300946	1,453024626	0,146217	0,87341
NAM7	281,1155142	1,420614091	0,9838975	1,443863949	0,148777	0,873929
_C603200WA	79,42825179	1,276586916	0,8849941	1,44248078	0,149167	0,875291
CTN1	18,65263379	-1,887435777	1,3102562	-1,440509	0,149723	0,877629
_C206120CA	13,854478	1,589306	1,1070541	1,435617311	0,151111	0,880615
_C307670WA	64,16144746	-1,306379304	0,909526	-1,43632973	0,150909	0,880615
_C502590CA	48,51424124	1,563569831	1,0879974	1,437108013	0,150687	0,880615
_CR01440CA	865,8141207	-1,173972863	0,8177521	-1,43560973	0,151113	0,880615
PDX3	472,8485869	-1,066337961	0,7429136	-1,43534586	0,151189	0,880615
RPA135	1016,859343	1,127353799	0,7857205	1,434802607	0,151343	0,880615
SMP3	22,38504064	1,510753116	1,0527754	1,43501941	0,151282	0,880615
BMT9	6,245552875	-2,127237499	1,4842897	-1,43316867	0,15181	0,882403
_C104960CA	77,90630957	-1,057057679	0,7447532	-1,41933962	0,1558	0,883696
_C112650CA	15,76237524	-1,526926353	1,0757869	-1,41935758	0,155795	0,883696
_C202950WA	44,40668349	-1,492451943	1,0494191	-1,42216966	0,154977	0,883696
_C205750WA	176,9763536	1,227125987	0,8639741	1,420327343	0,155512	0,883696
_C210090CA	74,08425925	1,332285558	0,9386745	1,419326472	0,155804	0,883696
_C403740WA	50,41930451	1,211391972	0,8491575	1,426580958	0,153701	0,883696
_C407060WA	717,392977	1,055046927	0,7434311	1,419159053	0,155853	0,883696
_C502580WA	139,0560491	-1,111357803	0,7771332	-1,43007381	0,152696	0,883696
_C704310CA	264,4104507	-1,186202799	0,8342175	-1,42193466	0,155045	0,883696
_C704320WA	24,17109022	-1,690114538	1,1894426	-1,42092988	0,155337	0,883696
_CR00670CA	33,7428335	1,227810568	0,8624359	1,423654318	0,154547	0,883696
CYC3	118,6015679	1,074020772	0,7567156	1,419318921	0,155806	0,883696
DBF4	92,66438121	1,067197714	0,7502055	1,422540515	0,154869	0,883696
DBP2	834,7277704	1,194157749	0,834487	1,431008288	0,152428	0,883696
EHT1	478,2356171	-0,954288473	0,6691704	-1,42607696	0,153846	0,883696
FMO1	9,728256516	-1,930524568	1,3567507	-1,42290297	0,154764	0,883696
HIT1	11,56632268	1,704452352	1,1980618	1,422674798	0,15483	0,883696
HSM3	20,50150427	-1,417506223	0,995615	-1,4237493	0,154519	0,883696
IFD3	38,64933774	-1,316970457	0,9275893	-1,41977758	0,155672	0,883696
IFH1	220,3206926	1,026620935	0,7204305	1,425010307	0,154154	0,883696
IRO1	71,49046198	-1,076613619	0,7580842	-1,42017692	0,155556	0,883696
SET6	28,73246944	1,285504923	0,8996881	1,42883395	0,153052	0,883696
ZCF16	59,35808494	-1,250170935	0,8744018	-1,4297443	0,15279	0,883696
ZCF26	10,41051647	-1,972100169	1,3866424	-1,42221251	0,154965	0,883696
PXA1	109,0238858	-1,086753138	0,7662736	-1,41823128	0,156123	0,884327

COF1	774,1449971	-1,114622773	0,7863945	-1,4173837	0,156371	0,884826
_C404990CA	18,08666825	-1,642487351	1,1604273	-1,415416	0,156947	0,885376
SLF1	28,16401183	1,271521458	0,8981496	1,415712399	0,15686	0,885376
VPS70	74,47473139	-1,174148541	0,8293308	-1,4157783	0,15684	0,885376
_C704150WA	96,11062404	1,033319165	0,7306205	1,414303502	0,157273	0,886316
DBP8	102,8918415	1,279171124	0,9051496	1,413215217	0,157592	0,887217
_C604480CA	134,0594619	-1,172433485	0,8311016	-1,41069813	0,158334	0,890486
EPL1	913,7213157	-1,119459157	0,79405	-1,40980948	0,158596	0,891059
_C306520CA	22,8557025	1,337545286	0,9505476	1,40713137	0,159388	0,894606
_C204770WA	206,1022709	-1,136707518	0,8108674	-1,40184136	0,160963	0,897173
_C301890CA	588,1532626	-1,018264728	0,7255589	-1,40342112	0,160491	0,897173
_C302410CA	75,08455203	-1,105242876	0,7870728	-1,40424487	0,160246	0,897173
_C600200CA	298,8157559	-1,112005213	0,7920705	-1,40392199	0,160342	0,897173
KEM1	446,7766718	-0,933441918	0,6656576	-1,40228546	0,16083	0,897173
MED3	71,46906623	-1,250901437	0,8919897	-1,40237204	0,160804	0,897173
UGA11	53,98353151	-1,303423413	0,9298263	-1,40179242	0,160977	0,897173
_C104600CA	301,0130346	1,026843423	0,7335743	1,399781085	0,161579	0,89802
_C110880WA	145,7766638	1,216899394	0,869081	1,400213973	0,161449	0,89802
_CR03590CA	30,20465035	-1,600937428	1,1438229	-1,39963755	0,161622	0,89802
_CR04170WA	537,0070453	1,258453956	0,8994595	1,399122455	0,161776	0,89802
_C100190CA	6,672200294	-2,272419474	1,6252905	-1,39816203	0,162064	0,898721
SAS10	338,8091998	0,944231216	0,6759382	1,396919441	0,162438	0,899892
_C210140WA	28,08719493	-1,317408527	0,9447956	-1,39438463	0,163201	0,900774
_C304800CA	77,41207726	-1,168075038	0,8377881	-1,39423685	0,163246	0,900774
_C703820CA	11,16432794	-1,683522474	1,2065169	-1,39535753	0,162908	0,900774
DNM1	262,3819801	-0,944763042	0,6772449	-1,39500953	0,163013	0,900774
_C114180WA	15,05677421	-1,552571427	1,1150414	-1,3923891	0,163805	0,901593
DAG7	80,43654807	1,078212899	0,77436	1,392392277	0,163804	0,901593
FGR41	204,4907316	-0,981233707	0,7048413	-1,39213425	0,163882	0,901593
_C105210CA	178,3055872	1,053020632	0,7587163	1,387897799	0,165168	0,904366
_C112680WA	312,7999411	1,279522158	0,9210013	1,389272848	0,16475	0,904366
_C402690WA	77,08121023	-1,22337454	0,882158	-1,38679758	0,165503	0,904366
_CR06600CA	20,64025804	-1,391465698	1,0015116	-1,38936558	0,164722	0,904366
HMT1	394,893723	0,995693604	0,7180855	1,386594682	0,165565	0,904366
MCR1	654,3696735	0,94446631	0,6815664	1,385728993	0,16583	0,904366
PUS4	277,4871406	0,940828121	0,6780711	1,387506559	0,165287	0,904366
TKL1	4097,117569	0,918478407	0,6614243	1,388637139	0,164943	0,904366
YOR1	142,6913678	1,077943599	0,7779303	1,385655777	0,165852	0,904366
_C100330CA	131,9545618	-1,144821433	0,8331188	-1,3741395	0,169398	0,906597
_C103870CA	142,5799907	1,355528893	0,9885417	1,371240915	0,1703	0,906597
_C108780WA	167,1950042	-1,14569236	0,8312308	-1,37830842	0,168108	0,906597
_C110360CA	199,1822366	1,194099553	0,868832	1,374373415	0,169326	0,906597
_C111570WA	7,884111311	1,905263964	1,3845962	1,376043049	0,168808	0,906597
_C114520WA	2079,411474	-1,22651476	0,890658	-1,37708833	0,168485	0,906597
_C200750WA	8,512783065	-1,773927989	1,2948776	-1,36995808	0,1707	0,906597
_C305860CA	45,4763065	1,494183517	1,0901921	1,370568982	0,170509	0,906597
_C402860WA	224,455649	-1,110928437	0,8079165	-1,37505347	0,169115	0,906597
_C406470WA	89,43994728	1,074129446	0,7798773	1,377305724	0,168418	0,906597
_C503870CA	51,52256585	-1,129229406	0,8245632	-1,36948792	0,170847	0,906597
_C602250WA	9,962602011	1,711337876	1,2524305	1,366413489	0,171809	0,906597

_C604450WA	89,86935614	1,017101499	0,7440719	1,36693983	0,171644	0,906597
_C700070CA	1221,892861	-1,187352115	0,8646966	-1,37314307	0,169708	0,906597
_C700590WA	53,42344366	-1,196209485	0,8669301	-1,37982224	0,167641	0,906597
_C702220CA	21,77741448	-1,292821852	0,9355629	-1,38186527	0,167013	0,906597
_CR01420WA	212,1772941	0,957197394	0,6927214	1,381792696	0,167035	0,906597
_CR06820WA	54,53461356	-1,239684033	0,9023584	-1,37382671	0,169495	0,906597
_CR09740WA	96,40759369	1,102467064	0,8058863	1,368018202	0,171306	0,906597
CSH3	354,804244	-1,130026473	0,8268743	-1,3666242	0,171743	0,906597
CSP37	178,6194948	-0,975898481	0,7093492	-1,37576605	0,168894	0,906597
CTA24	706,9614055	-1,226207039	0,8922754	-1,37424732	0,169365	0,906597
CTA26	293,0330222	-1,180936357	0,864338	-1,36629001	0,171848	0,906597
ECM21	142,8903911	-0,972992533	0,7118428	-1,3668644	0,171668	0,906597
FAA21	96,96996697	-1,009493725	0,7390807	-1,36587745	0,171977	0,906597
FAT1	215,0498855	0,943609397	0,6895755	1,36839175	0,171189	0,906597
PNG2	182,8027766	0,962786362	0,6968383	1,381649515	0,167079	0,906597
PTC6	115,7262688	-1,108031801	0,8093189	-1,36909175	0,170971	0,906597
RAD14	154,6319534	-1,20665554	0,8831939	-1,36624074	0,171863	0,906597
SIP5	86,56662727	-1,064958027	0,7735519	-1,37671182	0,168601	0,906597
STD1	128,7821234	-1,153777987	0,8425526	-1,36938398	0,170879	0,906597
TOM1	1181,522315	-0,936945776	0,682302	-1,3732127	0,169686	0,906597
URA3	74,98530544	1,105573904	0,8017393	1,378969328	0,167904	0,906597
UTP21	282,9172916	1,419936242	1,0281386	1,381074739	0,167256	0,906597
YCP4	1133,176743	0,945563763	0,6902542	1,369877661	0,170725	0,906597
PDR17	117,5273577	1,035692356	0,758582	1,365300553	0,172159	0,906691
_C201750CA	53,85507604	-1,265852182	0,9293173	-1,36213127	0,173156	0,907036
_C300010CA	436,4483925	-1,31928024	0,9685975	-1,36205207	0,173181	0,907036
_CR07320CA	243,1540282	1,154077406	0,846397	1,363517915	0,172719	0,907036
HTS1	1313,270985	0,900584711	0,6610551	1,362344481	0,173089	0,907036
PTC2	981,4807646	-1,054753212	0,7731477	-1,36423244	0,172494	0,907036
TUF1	1909,245829	0,943854613	0,6930022	1,361979303	0,173204	0,907036
PEX2	86,61781239	-1,122654801	0,8253554	-1,36020781	0,173764	0,908253
RKI1	69,87925475	1,074058139	0,7893846	1,360627207	0,173632	0,908253
MLH3	99,4291575	-1,064093076	0,783565	-1,35801499	0,174459	0,911027
_C204120CA	291,193619	0,931908461	0,6865279	1,357422588	0,174647	0,911034
_C604260CA	38,13024071	1,137784011	0,8391444	1,355885853	0,175135	0,911034
_CR05460WA	71,81619164	-1,23936771	0,9139088	-1,35611748	0,175062	0,911034
_CR07680CA	6,61078807	-1,949992652	1,4376112	-1,35641167	0,174968	0,911034
IML2	275,3833684	-0,941297205	0,6944644	-1,35542894	0,175281	0,911034
_C307920WA	218,7970266	-1,171413179	0,8649788	-1,35426806	0,175651	0,91125
_CR04680CA	13,19864025	-1,553561633	1,1467359	-1,35476852	0,175491	0,91125
_C104350CA	34,27680233	-1,405316	1,0400299	-1,35122647	0,176623	0,911864
_C110910CA	281,8063124	-1,147694744	0,849613	-1,35084411	0,176745	0,911864
_C502020CA	353,1389216	-1,235137124	0,9136864	-1,3518173	0,176434	0,911864
_C505460CA	45,84201475	-1,238401867	0,9162542	-1,35159201	0,176506	0,911864
_C601470WA	11,18452461	-1,574172824	1,1642594	-1,3520808	0,176349	0,911864
MDH1	123,4650913	-0,96048991	0,7110449	-1,35081472	0,176755	0,911864
_C600550WA	262,8988707	1,039393221	0,7702342	1,349450939	0,177192	0,912425
NMA111	339,7724225	1,019733025	0,7555753	1,349611315	0,177141	0,912425
_CR00700WA	15,03704832	-1,454807584	1,0790452	-1,34823598	0,177582	0,913587
_C101390CA	81,68362824	1,037785213	0,7702257	1,347378094	0,177858	0,91416

_C201510CA	20,563932	1,307874429	0,9727047	1,344574994	0,178763	0,917956
_CR01260WA	36,53541544	-1,148648395	0,8546206	-1,34404488	0,178934	0,917987
_C113820CA	39,90818864	1,478105253	1,1005898	1,343011893	0,179268	0,918005
PGA7	53,86844474	1,366101644	1,0168188	1,343505528	0,179108	0,918005
_CR00160CA	56,19771923	-1,097792483	0,8183633	-1,34144878	0,179775	0,918961
MHP1	354,9615871	-0,957998841	0,7141706	-1,34141461	0,179786	0,918961
_C102380CA	12,83493334	-1,589245504	1,1911966	-1,33415889	0,182152	0,920041
_C104090CA	23,59248148	-1,284710833	0,9598254	-1,3384839	0,180739	0,920041
_C114430CA	252,8165945	-1,191588165	0,8914453	-1,33669245	0,181323	0,920041
_C209460CA	126,1596974	-0,945680116	0,7070269	-1,33754479	0,181045	0,920041
_C502070CA	112,9569963	0,975480275	0,730383	1,335573665	0,181689	0,920041
_CR01380WA	72,63694883	1,059833178	0,7909352	1,339974694	0,180254	0,920041
_CR01780WA	50,17303142	1,353290945	1,0104658	1,339274385	0,180481	0,920041
_CR05730CA	145,7432789	-0,935335237	0,6989642	-1,33817331	0,18084	0,920041
GPX3	21,03036259	-1,307886241	0,9770472	-1,33861107	0,180697	0,920041
HAS1	624,7491602	1,158396424	0,8681098	1,33438936	0,182076	0,920041
PHO84	1345,663849	-1,135750741	0,8501235	-1,33598329	0,181555	0,920041
SYS1	103,4438815	-1,292656987	0,9685752	-1,33459636	0,182008	0,920041
UTP8	258,2346846	1,053875835	0,7892728	1,335249122	0,181795	0,920041
_C105330CA	58,28193802	1,110512062	0,8333017	1,332665025	0,182642	0,921677
_CR01950WA	231,2906492	0,982168051	0,7375047	1,33174481	0,182944	0,921775
RNR3	50,61683079	1,102474281	0,8279351	1,331595057	0,182993	0,921775
_C206800CA	28,22075137	1,194270009	0,8975588	1,330575778	0,183329	0,922622
_C208700CA	167,2375724	-1,152433296	0,8674291	-1,32856194	0,183993	0,922622
_C505440CA	931,4458214	-0,900539721	0,6773206	-1,32956208	0,183663	0,922622
DIM1	47,52655389	1,344130988	1,0107426	1,32984497	0,183569	0,922622
PCL7	188,7181347	-0,921539933	0,6933939	-1,32902804	0,183839	0,922622
_CR07300WA	6,545114261	-2,178156453	1,6419178	-1,32659286	0,184643	0,92505
AHA1	202,3476556	0,936316264	0,7068839	1,324568653	0,185314	0,926259
ARO9	17,73427532	1,369870303	1,0451015	1,310753332	0,189941	0,926259
BMT5	61,36260803	-1,036517676	0,7833286	-1,32322207	0,185762	0,926259
_C104160CA	12,8566583	-1,460722902	1,111514	-1,3141741	0,188788	0,926259
_C107990CA	80,3874124	-1,296220209	0,9913762	-1,30749581	0,191044	0,926259
_C113810WA	698,3314542	-1,121832885	0,8590453	-1,30590654	0,191584	0,926259
_C200940WA	136,1057769	-1,285178775	0,9778101	-1,31434391	0,18873	0,926259
_C203140CA	223,5209851	-0,98569399	0,7467165	-1,3200378	0,186822	0,926259
_C207040WA	77,34622913	0,979903998	0,7481263	1,309811004	0,19026	0,926259
_C304380CA	328,3637139	1,016531989	0,7724858	1,315923206	0,1882	0,926259
_C307600WA	154,1169237	0,922209188	0,7035034	1,310880905	0,189898	0,926259
_C403950CA	133,4077911	-1,332258783	1,0093717	-1,31988922	0,186872	0,926259
_C500260WA	509,602266	0,945606061	0,7237957	1,306454409	0,191398	0,926259
_C501200WA	21,08381024	-1,584138864	1,2024583	-1,31741694	0,187699	0,926259
_C501430CA	90,5666693	0,978610557	0,7482635	1,307842195	0,190927	0,926259
_C501610WA	46,41861621	1,426747677	1,0932934	1,304999762	0,191893	0,926259
_C601450CA	17,56521015	-1,362237696	1,0316054	-1,32050268	0,186667	0,926259
_C701030CA	563,5280508	1,197067408	0,9055355	1,321944239	0,186187	0,926259
_C701400CA	102,5801721	-1,107711061	0,8386105	-1,32088869	0,186538	0,926259
_C702080WA	29,96158134	-1,153852693	0,8838101	-1,30554362	0,191708	0,926259
_C702600CA	210,0601262	-0,972033617	0,7413137	-1,31123117	0,18978	0,926259
_CR00110WA	93,62674556	-0,959485381	0,726178	-1,3212812	0,186408	0,926259

_CR01770CA	360,6846484	-1,080374466	0,8238528	-1,31136831	0,189733	0,926259
_CR03360WA	217,2661286	0,921450521	0,7026863	1,311325547	0,189748	0,926259
_CR04300WA	735,3184498	0,964622669	0,7371731	1,308542893	0,190689	0,926259
_CR06260WA	31,28385633	-1,243523467	0,9479003	-1,31187164	0,189563	0,926259
_CR08650CA	301,4237199	-1,132060932	0,8630121	-1,31175553	0,189603	0,926259
_CR10770WA	79,61395693	-1,207284721	0,9138052	-1,32116209	0,186447	0,926259
CHO1	73,06159718	1,005165294	0,7657942	1,312578964	0,189325	0,926259
DAD1	7,468344494	-1,754504982	1,3436022	-1,30582179	0,191613	0,926259
DDI1	305,2207909	-1,097390637	0,8297205	-1,32260283	0,185967	0,926259
ERG251	255,9719387	-0,901081083	0,6835928	-1,31815477	0,187452	0,926259
LEU5	248,0836722	-1,215503355	0,9279498	-1,30988044	0,190236	0,926259
NOG1	510,6089905	1,104436269	0,8372014	1,319200155	0,187102	0,926259
PGA11	12,38494485	-1,588685639	1,2037828	-1,31974442	0,18692	0,926259
PHO23	173,0633954	-1,062453775	0,8122235	-1,30808063	0,190846	0,926259
RME1	67,10875089	-1,004807976	0,7636098	-1,31586581	0,188219	0,926259
RPF2	162,9140028	1,190441216	0,8999921	1,322724058	0,185927	0,926259
RTF1	310,4984444	-1,006319351	0,7710667	-1,3051003	0,191859	0,926259
SMM1	24,88703436	1,473616673	1,1225491	1,312741366	0,18927	0,926259
TLO5	105,5069735	-1,20147893	0,9141955	-1,31424732	0,188763	0,926259
TOK1	62,94597649	1,013274726	0,7712882	1,313743261	0,188933	0,926259
_C205300CA	227,5105412	1,11988686	0,8585945	1,304325713	0,192123	0,926561
_CR00350WA	267,046177	-0,893026711	0,6855388	-1,30266401	0,192689	0,928489
_C106630WA	109,9446063	-0,95467299	0,7357045	-1,29763103	0,194414	0,929446
_C107950CA	41,27368401	1,209704045	0,9315612	1,298577062	0,194089	0,929446
_C110750CA	92,60718244	-1,075288159	0,8281793	-1,29837606	0,194158	0,929446
_C110950CA	240,2670328	0,925179653	0,712363	1,298747543	0,194031	0,929446
_C201190CA	15,74579374	-1,474071068	1,1386501	-1,29457774	0,195466	0,929446
_C204570WA	1072,58861	0,862472311	0,6663815	1,294262067	0,195575	0,929446
_C306550CA	18,80514185	-1,270075658	0,9810274	-1,29463826	0,195445	0,929446
_C307280CA	120,848232	-0,927118582	0,7152619	-1,29619448	0,194908	0,929446
_C307290WA	108,3475506	0,965894669	0,7460615	1,294658178	0,195438	0,929446
_C702450WA	70,26372285	1,001926699	0,7744099	1,293793723	0,195737	0,929446
CAT2	177,470582	-0,896581212	0,6908347	-1,29782313	0,194348	0,929446
ERG2	68,08721146	1,056349847	0,8167766	1,293315521	0,195902	0,929446
GDA1	189,8426032	1,112539373	0,8564318	1,299040185	0,19393	0,929446
ISU1	112,3193375	0,931103302	0,7184901	1,295916687	0,195004	0,929446
OPT7	29,23996333	-1,368333069	1,0563785	-1,29530565	0,195215	0,929446
SHE9	135,3896019	-1,069818987	0,8241828	-1,29803605	0,194275	0,929446
TDH3	17310,89967	-0,891975749	0,6893161	-1,29400111	0,195665	0,929446
TSR1	581,3668754	1,076225564	0,8317199	1,293976038	0,195674	0,929446
_CR07600WA	217,5640291	0,938921461	0,7267388	1,291965515	0,196369	0,930867
_C601520WA	8,152779153	-1,698096884	1,3152336	-1,29109907	0,196669	0,931494
CRH12	50,25252757	-1,053356974	0,8166755	-1,28981095	0,197116	0,932816
_C101140CA	296,5138338	-1,000463781	0,7777499	-1,2863566	0,198319	0,937442
_C112470WA	7,104637589	-1,830379031	1,4243514	-1,28506143	0,198771	0,937442
_C114470WA	420,6946095	-1,184627608	0,9231207	-1,28328573	0,199392	0,937442
_C701020CA	81,26514326	1,07713674	0,8393518	1,283295945	0,199388	0,937442
CCT8	1275,289284	0,846485206	0,6589842	1,284530404	0,198956	0,937442
CEF1	209,8920063	-1,029246704	0,8021348	-1,28313428	0,199445	0,937442
MAK5	784,8789975	0,879271338	0,6848064	1,283970663	0,199152	0,937442

RAD57	48,049854	-1,100774826	0,8563993	-1,28535226	0,198669	0,937442
NPL6	930,8515011	-1,094035767	0,8539393	-1,28116334	0,200136	0,939896
_C602020CA	152,9205319	-1,127587056	0,881156	-1,27966787	0,200662	0,941568
AYR2	31,78035133	-1,127555441	0,8815517	-1,2790577	0,200877	0,941779
HSE1	187,6455952	-1,016230927	0,7953396	-1,27773208	0,201344	0,943173
_C601780CA	45,17906942	-1,099619018	0,8613254	-1,27665928	0,201723	0,94415
_C205640WA	176,7674228	-1,29638767	1,0164734	-1,27537785	0,202176	0,944677
HYR1	29,77329284	1,124761794	0,8816179	1,275792878	0,202029	0,944677
_C700330CA	105,7712664	0,980299776	0,7699152	1,27325677	0,202927	0,946594
NOP1	1336,440361	0,940324623	0,7383894	1,273480587	0,202848	0,946594
_C300050CA	49,42243224	-1,263962471	0,9973766	-1,26728704	0,205053	0,947887
_C306280WA	180,9674037	-1,073341518	0,8472365	-1,26687353	0,205201	0,947887
_C306940WA	283,8936601	-1,064265647	0,841437	-1,26481921	0,205936	0,947887
_C503630CA	19,59216805	1,294962722	1,0223436	1,266660952	0,205277	0,947887
_C701960WA	68,72278664	-0,964482676	0,7623669	-1,26511616	0,20583	0,947887
_CR02700WA	24,95712909	1,19807041	0,9466111	1,26564159	0,205641	0,947887
_CR03330WA	102,0003805	-1,029477054	0,8122134	-1,26749574	0,204978	0,947887
_CR08710WA	495,171296	-0,944333896	0,7458622	-1,26609694	0,205478	0,947887
COX11	182,9663516	0,895699376	0,7079498	1,265201786	0,205799	0,947887
MEC1	117,916263	0,990471204	0,7790303	1,271415551	0,203581	0,947887
MED18	66,56557639	0,975373793	0,7693553	1,267780612	0,204876	0,947887
PMC1	203,3208495	0,879533019	0,6938256	1,267657137	0,20492	0,947887
RPS42	386,7984796	0,94081357	0,7432236	1,265855273	0,205565	0,947887
STT3	340,0652278	0,87555823	0,6914931	1,26618514	0,205447	0,947887
TCO89	143,360066	-0,918299305	0,725291	-1,26611157	0,205473	0,947887
TLO11	37,98050912	-1,189801175	0,939797	-1,26601939	0,205506	0,947887
BZZ1	113,3233238	-0,923288715	0,7306636	-1,26363038	0,206363	0,948169
_C302630CA	235,7739292	-0,890907737	0,7052669	-1,26322069	0,20651	0,948169
_C400390WA	484,1917839	-1,124250993	0,8896411	-1,263713	0,206333	0,948169
_C307650CA	35,97087535	1,080881179	0,8564388	1,262064661	0,206926	0,948534
_CR03270WA	35,90261573	1,077077093	0,853704	1,261651667	0,207074	0,948534
CRC1	114,7306753	-0,975333974	0,7735203	-1,26090293	0,207344	0,948534
MET13	107,1064212	-1,071789043	0,8499931	-1,26093848	0,207331	0,948534
SKN1	77,3324406	1,057170778	0,838608	1,260625738	0,207444	0,948534
_C202180WA	26,81836165	-1,117338675	0,8870889	-1,25955659	0,207829	0,948597
RPC11	53,50611371	0,990195908	0,7863901	1,259166318	0,20797	0,948597
SCH9	123,9565312	1,125645103	0,8939537	1,259176004	0,207967	0,948597
CWC22	23,79375063	1,213788419	0,9652287	1,257513853	0,208568	0,950541
UTP13	195,6898586	1,289638284	1,0261909	1,256723568	0,208854	0,951064
_C300160CA	239,9091446	-1,080991625	0,8611917	-1,25522758	0,209396	0,951355
ELP3	132,9663187	1,015324613	0,8087524	1,255420879	0,209326	0,951355
MET1	116,771695	1,013250508	0,8072875	1,255129618	0,209432	0,951355
_CR02390WA	165,307364	-0,877630695	0,699816	-1,25408774	0,20981	0,951737
NAG6	664,0067176	-1,023843178	0,816492	-1,25395375	0,209859	0,951737
HIP1	114,9046218	0,905924863	0,7240524	1,25118687	0,210866	0,955526
_C501920CA	52,58889926	-1,079932961	0,8639191	-1,25003944	0,211285	0,955863
_C700580CA	35,14055381	-1,137120415	0,9093965	-1,2504121	0,211149	0,955863
_C102030CA	61,12784467	-1,223352111	0,9793196	-1,24918581	0,211597	0,956495
_C202550CA	7,962194013	-1,665342664	1,3341947	-1,24820058	0,211958	0,957345
_C111950WA	5,202642595	-2,100589217	1,6861121	-1,24581825	0,212831	0,958949

_C306140WA	25,20514219	-1,164052449	0,934131	-1,24613402	0,212715	0,958949
NOP8	109,5078562	0,911121936	0,7310987	1,246236607	0,212678	0,958949
_C108630WA	91,54814974	1,158439623	0,9302741	1,245267005	0,213034	0,959083
_C113240WA	18,1910537	-1,233989314	0,9928175	-1,24291658	0,213899	0,96064
_C114530WA	241,8722987	-0,998891527	0,8031426	-1,24372865	0,213599	0,96064
_C209000CA	23,0634699	-1,370220489	1,1023643	-1,24298336	0,213874	0,96064
ALG9	103,3051862	-0,971421329	0,7839257	-1,23917518	0,215281	0,963648
_C101860WA	224,1938534	0,847789825	0,6843887	1,238754891	0,215436	0,963648
_C111050WA	7,261647468	1,828793828	1,4750688	1,239802391	0,215048	0,963648
_C502010CA	159,9162272	1,00666493	0,8115429	1,240433417	0,214815	0,963648
MSN5	671,4078362	-0,824968251	0,6659168	-1,23884591	0,215403	0,963648
AGP2	230,7754678	-0,916319229	0,7444321	-1,23089705	0,218361	0,96381
ASH2	136,2984537	0,874211642	0,7083323	1,234182959	0,217135	0,96381
BMS1	915,3760996	0,815616589	0,6636527	1,228980961	0,219079	0,96381
_C102390WA	50,73511469	-1,036147536	0,8429483	-1,22919459	0,218999	0,96381
_C107100CA	16,431992	-1,270560359	1,0297524	-1,23385034	0,217259	0,96381
_C111010CA	100,9369622	-1,188397339	0,9608324	-1,23684141	0,216146	0,96381
_C111600WA	55,1991689	-1,153633088	0,9373468	-1,23074314	0,218419	0,96381
_C112740WA	76,2583273	-0,921827653	0,7501176	-1,22891085	0,219105	0,96381
_C200790CA	106,9513796	0,970483246	0,7893933	1,229403911	0,21892	0,96381
_C202820CA	45,10609887	0,999671953	0,8119756	1,231160129	0,218263	0,96381
_C303460CA	13,29028198	1,531205653	1,2453976	1,229491403	0,218888	0,96381
_C305430WA	16,92366682	-1,279990801	1,036762	-1,23460425	0,216978	0,96381
_C404520WA	224,1933045	0,970939539	0,7899108	1,229176133	0,219006	0,96381
_C505040WA	63,47652441	-1,021400269	0,8303396	-1,23009937	0,21866	0,96381
_C602800WA	45,09128097	1,040421097	0,8427532	1,234550192	0,216998	0,96381
_C604530CA	313,1828351	1,05548565	0,8572138	1,231298062	0,218211	0,96381
_CR09140CA	34,24380905	-1,177735319	0,9594687	-1,22748698	0,21964	0,96381
CSO99	37,35507058	-1,023282135	0,82753	-1,23654997	0,216254	0,96381
HPT1	295,5399978	0,900226358	0,7331578	1,227875332	0,219494	0,96381
SMI1	798,3095431	-0,927701705	0,7528853	-1,23219525	0,217876	0,96381
UBA4	83,79893119	0,928290843	0,7525458	1,233533952	0,217377	0,96381
VPH2	24,20956062	-1,175215587	0,9570701	-1,22793048	0,219473	0,96381
YVC1	197,0885978	0,910664722	0,7376996	1,234465529	0,217029	0,96381
ZCF15	70,91290499	-0,938510895	0,7644242	-1,22773567	0,219546	0,96381
_C400660WA	207,710806	1,181667702	0,9648342	1,224736535	0,220675	0,967586
ATP16	2256,469337	-0,957558818	0,7826335	-1,22350857	0,221138	0,968796
PDC2	67,05105023	0,949902218	0,7767522	1,222915344	0,221362	0,968796
RPL7	196,18405	1,060264833	0,8672086	1,222617919	0,221474	0,968796
AAH1	39,45959841	1,029278324	0,8464214	1,216035382	0,223971	0,96884
AMO2	30,13364558	-1,067176251	0,8823944	-1,20940959	0,226506	0,96884
ARX1	479,2381991	0,904828138	0,7488438	1,208300241	0,226932	0,96884
_C106800WA	45,18335243	1,07185747	0,8781487	1,220587669	0,222242	0,96884
_C109350WA	172,7277129	1,059704626	0,8730323	1,213820669	0,224816	0,96884
_C109540WA	40,66845401	1,062871307	0,8802844	1,207418131	0,227271	0,96884
_C203170WA	16,33236114	-1,278532602	1,0526868	-1,21454222	0,224541	0,96884
_C206510WA	38,7178269	-1,152726972	0,9466468	-1,21769484	0,22334	0,96884
_C209680WA	67,63504579	-0,9943018	0,8177441	-1,21590824	0,22402	0,96884
_C300840CA	52,54819796	0,950957108	0,7881979	1,206495312	0,227627	0,96884
_C303070WA	38,60686809	1,006842134	0,8331726	1,208443684	0,226877	0,96884

_C400320CA	72,90512596	-1,065096644	0,8769236	-1,21458313	0,224525	0,96884
_C403170WA	40,0381134	1,01887976	0,8354433	1,219567842	0,222629	0,96884
_C504360CA	118,8994896	-0,994418381	0,8185706	-1,21482292	0,224434	0,96884
_C602380WA	274,0684708	1,112694715	0,9158542	1,214925637	0,224394	0,96884
_C603210CA	31,15929055	1,34253409	1,1114455	1,207917126	0,227079	0,96884
_C604470CA	234,3886655	-0,968408387	0,7996131	-1,21109617	0,225859	0,96884
_C604550CA	161,924348	-1,033875422	0,8487254	-1,21815065	0,223167	0,96884
_C702460CA	136,7464746	0,918597733	0,7590584	1,210180563	0,22621	0,96884
_CR02570CA	30,23571461	-1,140025498	0,9388193	-1,21431836	0,224626	0,96884
_CR02670CA	102,5185946	-0,898106244	0,7397409	-1,21408213	0,224716	0,96884
_CR05550CA	461,3866151	0,941536943	0,774229	1,216096193	0,223948	0,96884
_CR05970CA	245,4053282	0,834226359	0,6907973	1,207628258	0,22719	0,96884
_CR06570CA	6,034359958	-1,832106001	1,5146157	-1,20961771	0,226426	0,96884
HAP3	8,796409365	-1,638966259	1,3587417	-1,20623828	0,227726	0,96884
HGT18	35,27231253	-1,166272477	0,9588907	-1,21627259	0,223881	0,96884
HST3	123,567749	0,864483017	0,7120118	1,214141488	0,224694	0,96884
NEP1	128,2354774	1,140693466	0,9364497	1,218104378	0,223184	0,96884
NHP2	324,8305337	0,918152558	0,7605498	1,207222198	0,227347	0,96884
OPT4	6,956385187	-1,764211497	1,4559893	-1,21169265	0,22563	0,96884
PHO113	25,17109731	-1,101319526	0,9120805	-1,20748065	0,227247	0,96884
REG1	640,3041455	-0,796862641	0,6606771	-1,20613034	0,227767	0,96884
RPT4	538,9828673	0,842668832	0,6918215	1,218043685	0,223207	0,96884
SSP96	21,95021312	-1,364852804	1,1184278	-1,22033167	0,222339	0,96884
SYN8	26,43871966	-1,082551413	0,8936009	-1,21144847	0,225724	0,96884
ZCF5	8,154307444	-1,654302898	1,3714316	-1,2062599	0,227717	0,96884
_C405380CA	12,52152375	-1,326798223	1,1014261	-1,20461848	0,228351	0,969835
_CR00460CA	61,61935786	0,981391751	0,8146097	1,204738664	0,228304	0,969835
POP4	25,7702659	1,096641346	0,9107898	1,204055321	0,228568	0,970017
_C112700WA	21,63841964	-1,237693355	1,0306466	-1,20089014	0,229794	0,970192
_C306530WA	22,12338646	1,139525576	0,9486324	1,201229891	0,229662	0,970192
DQD1	247,3183041	-0,930189631	0,7741821	-1,20151266	0,229552	0,970192
DTD2	328,094451	-0,978899116	0,8147834	-1,20142257	0,229587	0,970192
LYS22	133,3605971	1,047525107	0,8714397	1,202062612	0,229339	0,970192
PIN3	564,1487637	-0,98319409	0,8179821	-1,20197502	0,229373	0,970192
ZCF13	64,50855509	-0,910426378	0,7581899	-1,20078931	0,229833	0,970192
_C106660WA	109,6742578	-0,857280759	0,7149765	-1,19903351	0,230515	0,971593
PEX7	220,2852398	-1,004075569	0,8371912	-1,19933836	0,230396	0,971593
_C112150CA	12,73424496	-1,425317492	1,1907339	-1,19700755	0,231304	0,972702
_C204180CA	65,73531332	-0,993377173	0,829498	-1,19756425	0,231087	0,972702
TAF145	612,4473278	-0,970202203	0,8102998	-1,19733736	0,231175	0,972702
DAL1	84,8900728	-1,01650678	0,8495897	-1,19646791	0,231514	0,97285
_C114330WA	65,91318754	0,929999428	0,7809703	1,190825582	0,233722	0,973744
_C201630WA	27,0014332	1,080224084	0,9081129	1,189526172	0,234233	0,973744
_C303740WA	30,49808688	-1,199542597	1,0055473	-1,19292513	0,232899	0,973744
_C306420CA	76,12963833	1,020598282	0,8579281	1,189608144	0,2342	0,973744
_C505260WA	19,51944185	-1,292412337	1,0837703	-1,19251505	0,233059	0,973744
_C601370WA	39,90495738	-0,977943714	0,8187183	-1,19448127	0,23229	0,973744
_CR10750CA	127,5732515	0,878937225	0,7386991	1,189844784	0,234107	0,973744
HGH1	121,8325973	1,028398309	0,8627176	1,192045068	0,233244	0,973744
IFF4	21,31922018	-1,209933523	1,0160233	-1,19085217	0,233712	0,973744

PDE1	15,96921283	-1,21266173	1,0151733	-1,19453664	0,232268	0,973744
POT1-2	35,90282877	-1,101739654	0,9264489	-1,18920716	0,234358	0,973744
PYC2	1365,219205	1,120086666	0,9377084	1,194493607	0,232285	0,973744
SOH1	54,24671398	-1,074325968	0,901661	-1,1914966	0,233459	0,973744
WAR1	69,44220772	-0,914722707	0,7687768	-1,18984166	0,234109	0,973744
YPT1	479,6771905	-0,877718099	0,7376639	-1,18986182	0,234101	0,973744
_CR01090WA	43,56745454	-0,956463859	0,8052746	-1,18774863	0,234932	0,9754
_C108800WA	47,00612151	-0,965354329	0,8141126	-1,18577493	0,235711	0,977902
_C206200CA	120,2367113	0,860912469	0,7263694	1,185226794	0,235928	0,97807
_C204480WA	18,94083904	-1,26792216	1,0703383	-1,18459944	0,236176	0,978367
_C401280CA	69,58758774	0,896794692	0,7577655	1,183472601	0,236622	0,978754
POL32	317,9234702	-0,971149065	0,8204742	-1,18364368	0,236554	0,978754
_C110960WA	68,18547338	0,91133268	0,770858	1,182231564	0,237114	0,979017
_C201030WA	144,1040898	0,847230975	0,7165125	1,182437126	0,237032	0,979017
RHO1	897,6675742	-0,887077685	0,750503	-1,18197751	0,237215	0,979017
SGA1	32,51758129	-1,050378216	0,8892019	-1,18125952	0,2375	0,979465
_C501050CA	148,9837468	0,885641408	0,7506295	1,179864844	0,238054	0,979786
MED5	33,11308873	-1,041221326	0,8825914	-1,17973205	0,238107	0,979786
PIL1	1027,46044	-0,777854043	0,6592165	-1,17996749	0,238013	0,979786
_C604650WA	990,9545479	-1,041832857	0,8835221	-1,17918149	0,238326	0,979961
_C101950CA	53,29176106	-0,972766334	0,8255693	-1,17829764	0,238678	0,980682
_C207510WA	16,72029099	-1,215315273	1,0321797	-1,17742606	0,239025	0,981384
_C108760WA	26,22849644	-1,061821595	0,9029573	-1,17593774	0,23962	0,982296
_C113690CA	211,006872	-0,857103646	0,7287343	-1,17615376	0,239533	0,982296
_C202120WA	83,24906451	1,089536631	0,9270356	1,175291012	0,239878	0,982296
_CR00990WA	23,50354849	-1,082884677	0,9215274	-1,17509768	0,239956	0,982296
GUT2	629,6923082	-0,780244464	0,6643578	-1,17443406	0,240221	0,982659
CHS3	361,5834766	0,856492522	0,7304134	1,172613329	0,240951	0,984918
AMO1	25,5989499	-1,191757586	1,0179754	-1,17071352	0,241714	0,985312
_C207420WA	193,8506819	-0,859181138	0,7333019	-1,17166088	0,241333	0,985312
_CR01220WA	16,14218827	-5,486191155	4,6866275	-1,17060535	0,241757	0,985312
RIM9	21,04235345	-1,173723688	1,0026455	-1,17062674	0,241749	0,985312
_C206850WA	57,32788444	0,914370108	0,7823506	1,168747198	0,242505	0,986189
_C404720WA	583,5067269	0,81900848	0,7006986	1,168845583	0,242466	0,986189
PTR22	1542,702535	-0,942360484	0,805897	-1,16933112	0,24227	0,986189
_C405400CA	30,68105876	1,143431502	0,978721	1,16829159	0,242689	0,986213
_C505200CA	78,96016982	-1,000818463	0,8584424	-1,16585392	0,243674	0,989489
_C208770CA	34,41466829	-1,150843433	0,9875006	-1,16541041	0,243853	0,989494
_C304330CA	115,0266158	-0,969395759	0,8323124	-1,16470186	0,24414	0,989934
_C114160WA	88,25877162	0,929312592	0,7985312	1,163777502	0,244514	0,990729
ARP2	550,10398	-0,859680253	0,7404476	-1,16102773	0,245631	0,99345
_C207180WA	9,911753029	-1,419875429	1,225041	-1,15904317	0,246439	0,99345
_C300910WA	27,74867474	-1,061948731	0,9162123	-1,15906404	0,24643	0,99345
_C303440CA	27,42262789	-1,056954139	0,9112694	-1,15987015	0,246102	0,99345
_CR03760WA	94,99399316	1,014400431	0,8745901	1,159858124	0,246107	0,99345
NPR1	173,4776055	-0,808241414	0,6958852	-1,16145801	0,245456	0,99345
RCH1	27,17233445	-1,063012693	0,9166674	-1,15964927	0,246192	0,99345
_C105380CA	96,45776098	0,934776345	0,808083	1,156782652	0,247361	0,995325
_C113770CA	54,47157946	-1,126301832	0,9752427	-1,15489384	0,248134	0,995325
_C205490WA	13,63926473	1,261064786	1,09218	1,154630955	0,248242	0,995325

_C207800WA	34,29985884	1,182949349	1,0223422	1,157097241	0,247233	0,995325
_C700300WA	202,3617075	-0,982777102	0,8501938	-1,15594476	0,247704	0,995325
CYP5	579,6726937	-0,975822845	0,8443544	-1,15570292	0,247803	0,995325
RFG1	71,21229634	-0,915519261	0,7916147	-1,15652124	0,247468	0,995325
YCF1	312,8081332	0,78009077	0,6757573	1,154394965	0,248338	0,995325
_C307740WA	259,5565252	0,785083093	0,6816123	1,15180295	0,249402	0,995891
_C500320WA	279,3998273	-0,969037128	0,8418421	-1,15109129	0,249695	0,995891
_CR04490CA	28,36799419	-1,046544884	0,909254	-1,15099286	0,249735	0,995891
_CR07310WA	53,77525117	0,937999616	0,8144378	1,151714309	0,249438	0,995891
_CR08590WA	130,049604	0,879069454	0,7629788	1,152154484	0,249258	0,995891
SSU81	33,3981555	-0,981095015	0,8522121	-1,15123342	0,249636	0,995891
THI13	16,7816154	-1,235358935	1,0727772	-1,15155218	0,249505	0,995891
CTR2	80,8668375	-1,014129632	0,8819014	-1,14993536	0,25017	0,996911
_C103690WA	187,9900679	-0,831989953	0,7252946	-1,14710631	0,251338	0,998829
_C109570WA	75,36976761	1,133721229	0,9876552	1,147891677	0,251013	0,998829
_CR00450CA	11,5400257	-1,370045095	1,1944343	-1,14702423	0,251372	0,998829
YAK1	70,93326067	-0,962936321	0,8386063	-1,14825787	0,250862	0,998829
GPI2	14,72775283	-1,347216591	1,175039	-1,14652924	0,251576	0,998927
_C106770WA	156,8508832	0,815288305	0,7117199	1,145518411	0,251994	0,998962
_C111970CA	86,88501521	-0,905276226	0,7901872	-1,14564782	0,251941	0,998962
_C400210WA	29,87497663	-1,218266511	1,0646068	-1,1443347	0,252485	0,998962
FGR50	146,2213637	-1,020183093	0,8911221	-1,14482972	0,25228	0,998962
PEX19	426,930626	-0,970974352	0,848194	-1,14475504	0,252311	0,998962
_C100790WA	16,07504467	-1,186072549	1,0371311	-1,14360909	0,252786	0,99944
AAF1	380,0322783	-0,137179119	0,6722701	-0,20405359	0,838312	0,999787
AAP1	59,48492281	-0,712560657	1,0131627	-0,70330326	0,481867	0,999787
AAT1	378,6531807	-0,324751237	0,8651467	-0,37537132	0,707384	0,999787
AAT21	215,1634447	-0,132079736	0,6850334	-0,19280773	0,84711	0,999787
AAT22	39,76965367	-0,576940977	1,0108879	-0,57072697	0,568185	0,999787
ABC1	274,7784605	0,39813281	0,8765516	0,454203529	0,649682	0,999787
ABD1	172,8429909	0,36116456	0,7223529	0,499983521	0,617087	0,999787
ABG1	115,6344058	0,369000534	0,7284184	0,506577749	0,612451	0,999787
ABP1	1189,186592	-0,892423174	0,7876856	-1,13296872	0,257227	0,999787
ABP140	33,6897149	1,144843526	1,117068	1,024864639	0,305427	0,999787
ABZ1	243,6715135	0,077549171	0,6858475	0,113070581	0,909975	0,999787
ACB1	70,4464052	-0,679047935	0,7495416	-0,905951	0,364962	0,999787
ACC1	4554,843828	0,861757069	0,7861629	1,096155845	0,273011	0,999787
ACE2	167,0906277	0,084171271	0,7711261	0,109153704	0,913081	0,999787
ACF2	107,1429637	0,695676533	0,7239519	0,960943097	0,336581	0,999787
ACH1	556,3726303	-0,275932653	0,6741861	-0,40928259	0,682332	0,999787
ACO1	5968,116402	0,223313888	0,7157283	0,31200929	0,755033	0,999787
ACO2	467,9485384	0,593672615	0,8832401	0,67215313	0,501486	0,999787
ACP1	84,53899712	0,092706273	0,8785272	0,105524645	0,91596	0,999787
ACP12	369,0794506	-0,366281202	0,7094924	-0,51625812	0,605674	0,999787
ACS2	2461,99724	0,677386341	0,7223376	0,937769777	0,348363	0,999787
ACT1	2103,933646	0,020569042	0,6945383	0,02961542	0,976374	0,999787
ADA2	62,05222488	0,36718575	0,771297	0,476062742	0,63403	0,999787
ADE1	496,2585948	0,273998613	0,6676074	0,410418802	0,681499	0,999787
ADE12	1113,694201	0,495362337	0,6615278	0,748815606	0,453968	0,999787
ADE13	1101,477491	0,608657535	0,7053579	0,862905984	0,388189	0,999787

ADE17	2520,843493	0,385416651	0,7025592	0,548589539	0,583287	0,999787
ADE2	586,139288	0,732827553	0,7249611	1,010850913	0,312088	0,999787
ADE4	462,0449384	0,677362896	0,8906133	0,7605578	0,446921	0,999787
ADE5%2C7	1338,254415	0,505252785	0,7461477	0,677148464	0,498312	0,999787
ADE6	1024,575845	0,26132221	0,8456548	0,30901761	0,757308	0,999787
ADE8	217,1458802	0,791022108	0,7037742	1,123971373	0,261025	0,999787
ADK1	690,357857	-0,463295932	0,6615043	-0,70036724	0,483698	0,999787
ADO1	126,455614	-0,658452349	0,854917	-0,77019445	0,441185	0,999787
ADP1	201,6055936	0,051317083	0,7085895	0,072421456	0,942267	0,999787
AFG1	27,5510888	-0,937828661	0,9239944	-1,01497228	0,310119	0,999787
AFG3	308,2683902	0,02865745	0,7097147	0,040378831	0,967791	0,999787
AFL1	637,4976519	-0,130455662	0,7181343	-0,18165915	0,85585	0,999787
AFP99	7,058677984	-0,383361527	1,3854465	-0,27670612	0,782006	0,999787
AFT2	58,20702627	0,072909135	0,8830341	0,08256661	0,934196	0,999787
AGC1	89,96026893	-0,524166038	0,7939003	-0,66024162	0,509099	0,999787
AGE1	105,1397025	-0,555219902	0,742331	-0,74794114	0,454496	0,999787
AGE2	153,2237187	-0,419289907	0,7002109	-0,59880516	0,549303	0,999787
AGE3	99,02568476	0,168447324	0,8472104	0,198825847	0,842399	0,999787
AGM1	95,63515226	0,095795822	0,7401188	0,129433038	0,897015	0,999787
AGO1	79,45768	-0,161922858	0,7605866	-0,21289207	0,831411	0,999787
AGP3	71,86645155	-0,072753684	0,8001636	-0,09092352	0,927553	0,999787
AHP1	213,8654013	0,478024552	0,6878797	0,6949246	0,487103	0,999787
AHP2	83,60799457	0,05997477	0,7657024	0,078326477	0,937568	0,999787
AHR1	88,60474196	-0,219631142	0,8260258	-0,26588896	0,790325	0,999787
AIP2	930,8837937	0,794596542	0,7115604	1,116695861	0,264124	0,999787
AKL1	290,5494508	-0,242227214	0,6877184	-0,35221861	0,724674	0,999787
AKR1	71,7028108	0,135187025	0,780818	0,173135135	0,862545	0,999787
ALA1	721,425514	0,606087891	0,8944204	0,677631972	0,498005	0,999787
ALG1	29,53208826	0,326658016	0,8749221	0,373356681	0,708883	0,999787
ALG11	22,42398891	0,70929373	0,9570334	0,74113788	0,45861	0,999787
ALG2	41,81980056	0,090451406	0,8240503	0,109764423	0,912596	0,999787
ALG5	22,75479151	-0,047916891	0,9437336	-0,05077375	0,959506	0,999787
ALG6	26,81753251	0,005958897	1,0938933	0,005447421	0,995654	0,999787
ALG7	110,5636578	-0,137566793	0,767786	-0,17917335	0,857802	0,999787
ALG8	26,47032521	-0,199283726	0,917885	-0,21711187	0,828121	0,999787
ALI1	109,6823923	-0,086890612	0,7964442	-0,10909819	0,913125	0,999787
ALK2	36,27174241	0,146120546	0,8505563	0,171794098	0,863599	0,999787
ALO1	556,5411916	0,130267918	0,6769656	0,192429155	0,847406	0,999787
ALR1	184,5375726	0,318245384	0,7954714	0,400071457	0,689104	0,999787
ALS3	47,53616029	0,005625229	0,8135729	0,006914228	0,994483	0,999787
ALS5	76,34963999	-0,31499635	0,7705725	-0,40878223	0,682699	0,999787
ALS9	65,86066035	-0,468706748	0,7857861	-0,59648131	0,550854	0,999787
ALT1	115,919216	-0,481393878	0,7283915	-0,6608999	0,508677	0,999787
AMN1	30,03455569	-0,452781244	1,0281632	-0,44037879	0,659663	0,999787
AMS1	21,52082648	-1,044364638	0,9676603	-1,0792678	0,280468	0,999787
ANB1	1810,668708	0,111949588	0,6900836	0,162226117	0,871128	0,999787
ANP1	101,1247928	0,482506983	0,7468064	0,646093757	0,518219	0,999787
AOX1	30,56254976	-0,968609939	0,9030781	-1,07256499	0,283466	0,999787
APA2	89,85869174	-0,224955037	0,7975934	-0,28204226	0,777911	0,999787
APC1	96,02954871	-0,345540653	0,7563179	-0,45687226	0,647763	0,999787

APE2	1199,940439	-0,424162021	0,7163187	-0,5921415	0,553756	0,999787
APE3	165,56319	-0,27488453	0,7004677	-0,39242997	0,694741	0,999787
APG7	53,4085544	-0,999690841	0,9124458	-1,09561669	0,273247	0,999787
APL2	98,7367756	-0,360867204	0,7768379	-0,46453348	0,642266	0,999787
APL4	117,7119953	-0,737141294	0,8299417	-0,88818447	0,374442	0,999787
APL5	222,2940489	-0,733255454	0,7075459	-1,03633629	0,300045	0,999787
APM1	343,1219186	-0,430499797	0,7487068	-0,57499115	0,565297	0,999787
APM4	36,00822496	0,166099604	0,8539154	0,1945153	0,845772	0,999787
APN1	167,7219699	0,255566264	0,6963458	0,367010539	0,713611	0,999787
APN2	19,53648127	0,42737454	1,009562	0,423326688	0,672057	0,999787
APR1	400,6013426	-0,265741243	0,7044185	-0,37724909	0,705989	0,999787
APT1	220,7835746	0,303313705	0,6847955	0,44292593	0,657819	0,999787
ARC1	964,1461829	0,300217353	0,659088	0,455504184	0,648747	0,999787
ARC15	117,638799	0,260552698	0,7121748	0,365854959	0,714473	0,999787
ARC19	18,40159427	-0,948611794	1,0353471	-0,91622584	0,359548	0,999787
ARC35	20,44998433	-0,752340408	1,0192193	-0,73815363	0,460421	0,999787
ARC40	196,4814282	-0,040327288	0,686537	-0,05874015	0,953159	0,999787
ARD	1637,291154	-0,730643641	0,7947981	-0,91928208	0,357948	0,999787
ARE2	40,47058694	-0,262475	0,8370215	-0,31358215	0,753838	0,999787
ARF2	507,2158565	-0,002794176	0,7511742	-0,00371974	0,997032	0,999787
ARF3	50,36139721	-0,236619248	0,7968439	-0,29694556	0,766508	0,999787
ARG1	41,52941945	0,425160446	0,8183436	0,519537784	0,603386	0,999787
ARG2	31,7977081	0,100062063	0,957973	0,104451865	0,916811	0,999787
ARG5%2C6	187,5973201	0,352849907	0,8147839	0,433059514	0,664972	0,999787
ARG8	11,36722129	-0,87493616	1,1544947	-0,75785204	0,44854	0,999787
ARG81	68,21913862	-0,079607379	0,8084676	-0,098467	0,921561	0,999787
ARH2	50,46852664	0,074110869	0,7992814	0,092721872	0,926125	0,999787
ARL1	121,8515972	-0,048992149	0,7527349	-0,06508553	0,948106	0,999787
ARL3	68,91166092	-0,550021423	0,7638032	-0,7201088	0,471458	0,999787
ARO1	1195,089955	0,654128446	0,7555707	0,865740869	0,386632	0,999787
ARO10	74,33012912	-0,408359663	0,8003431	-0,51023074	0,60989	0,999787
ARO2	514,8325328	0,420152685	0,7081213	0,593334374	0,552957	0,999787
ARO3	537,5886907	0,47129776	0,7964515	0,591746931	0,55402	0,999787
ARO4	805,0726396	0,484100597	0,7807065	0,62008018	0,535205	0,999787
ARO7	59,33895043	0,375230472	0,7832012	0,479098465	0,631869	0,999787
ARO8	1182,455641	0,554929528	0,6847387	0,810425287	0,417696	0,999787
ARO80	74,66824312	-0,296459205	0,7609857	-0,38957263	0,696853	0,999787
ARP1	25,96376113	0,172642876	0,9062328	0,190506093	0,848913	0,999787
ARP3	364,7552869	-0,189441718	0,6766916	-0,27995283	0,779514	0,999787
ARP4	96,47720925	-0,358004073	0,7287137	-0,4912822	0,623227	0,999787
ARP8	187,6943097	-0,498020381	0,7598995	-0,65537665	0,512225	0,999787
ARP9	87,91692643	0,002560068	0,8006819	0,003197359	0,997449	0,999787
ARV1	5,166099733	-1,535140457	1,5391918	-0,99736785	0,318586	0,999787
ASC1	3900,994822	0,358373665	0,7047544	0,508508587	0,611097	0,999787
ASE1	51,71480106	0,205229154	0,8360674	0,245469625	0,806093	0,999787
ASG1	86,485606	-0,338444163	0,8864657	-0,38179047	0,702617	0,999787
ASH1	254,026594	-0,699418097	0,7323853	-0,95498653	0,339585	0,999787
ASK1	87,54102384	-0,612501637	0,7439163	-0,82334749	0,41031	0,999787
ASM3	26,07097478	-0,661401686	0,9382681	-0,70491762	0,480862	0,999787
ASN1	1824,431631	0,509142567	0,8126628	0,626511484	0,53098	0,999787

ATF1	139,2139254	0,274883846	0,8251637	0,33312644	0,739039	0,999787
ATG1	68,04433415	0,240395772	0,757766	0,317242735	0,751059	0,999787
ATG9	39,98149909	-0,857989007	0,8891391	-0,96496606	0,334562	0,999787
ATM1	49,72172292	-0,49965501	0,800107	-0,62448527	0,532309	0,999787
ATO5	5,271372986	-1,723698929	1,7600344	-0,97935523	0,327404	0,999787
ATP1	6374,003205	0,199546303	0,6752383	0,295519842	0,767597	0,999787
ATP14	409,1251488	-0,371053907	0,6746705	-0,54997793	0,582335	0,999787
ATP18	277,4089365	-0,19880951	0,6762995	-0,29396668	0,768783	0,999787
ATP19	442,4165323	-0,30560527	0,6741469	-0,45332147	0,650317	0,999787
ATP2	7511,25044	0,315395484	0,6758577	0,466659628	0,640743	0,999787
ATP20	97,64530705	0,086065244	0,8906227	0,096634914	0,923016	0,999787
ATP3	1026,56649	-0,013406338	0,6600476	-0,02031117	0,983795	0,999787
ATP4	987,792592	0,015092052	0,6757821	0,02233272	0,982183	0,999787
ATP5	1002,752701	-0,125885731	0,6604852	-0,19059583	0,848842	0,999787
ATP7	106,8239266	0,088169148	0,8977955	0,098206269	0,921769	0,999787
ATS1	85,16086641	-0,308077226	0,9535106	-0,32309786	0,746621	0,999787
ATX1	53,76099779	0,905497204	0,8089084	1,119406314	0,262967	0,999787
AUR1	89,3285981	0,252050987	0,8663454	0,29093592	0,7711	0,999787
AVT1	42,80492548	-0,124367105	0,8327962	-0,14933678	0,881288	0,999787
AVT4	20,72628077	-0,348705156	0,9805322	-0,35562845	0,722119	0,999787
AVT7	41,69368306	0,600856835	0,8289661	0,724826751	0,468558	0,999787
AXL2	44,00381151	-0,030791885	0,8904829	-0,03457886	0,972416	0,999787
AYR1	99,59836683	0,117129539	0,7749891	0,151137022	0,879868	0,999787
BAT21	177,0896816	0,491476846	0,6932721	0,708923445	0,478372	0,999787
BAT22	202,2028922	-0,403724757	0,7393329	-0,54606627	0,58502	0,999787
BCK1	85,0600665	0,043706975	0,7475994	0,058463092	0,95338	0,999787
BCR1	88,59920025	-0,574112481	0,8338698	-0,68849177	0,491143	0,999787
BCY1	424,9989604	-0,650759442	0,669447	-0,97208514	0,331008	0,999787
BDF1	637,5522118	0,65846101	0,6686948	0,984695904	0,324773	0,999787
BEM1	133,4707005	-0,164545033	0,8396918	-0,19595885	0,844642	0,999787
BEM2	310,4521044	-0,226149515	0,781231	-0,28947843	0,772215	0,999787
BEM3	68,69915163	-0,195268396	0,7955284	-0,24545747	0,806102	0,999787
BET2	99,7777082	-0,239031005	0,7706183	-0,31018079	0,756423	0,999787
BFA1	110,6858673	-0,800027877	0,792994	-1,00887008	0,313037	0,999787
BFR1	901,9283541	0,132801942	0,7544443	0,176026164	0,860273	0,999787
BGL2	299,2507588	0,054046232	0,6859442	0,078791005	0,937199	0,999787
BGL22	37,04528093	-0,457293656	0,8356586	-0,54722548	0,584224	0,999787
BIG1	15,59366731	-0,173573991	1,0665904	-0,16273725	0,870725	0,999787
BIO2	44,72150191	0,209165647	0,8669068	0,241278136	0,80934	0,999787
BIO32	9,209284709	-0,231503532	1,2535669	-0,18467585	0,853483	0,999787
BLM3	441,4356872	-0,340494203	0,6672971	-0,5102588	0,60987	0,999787
BMH1	2051,610095	0,039143063	0,6599894	0,059308627	0,952706	0,999787
BMT1	106,8425343	0,216659562	0,7458822	0,290474223	0,771453	0,999787
BMT3	33,94863118	0,912574426	0,8902155	1,025116345	0,305308	0,999787
BMT6	318,4649768	0,164201908	0,7578088	0,216679851	0,828458	0,999787
BMT8	33,37287403	-0,327955283	0,8697975	-0,37704785	0,706138	0,999787
BNA4	352,9390473	0,319140715	0,6866435	0,464783683	0,642086	0,999787
BNI1	320,6604693	0,14245912	0,734353	0,193992692	0,846182	0,999787
BNI4	172,7016672	-0,123135436	0,7755031	-0,15878135	0,873841	0,999787
BNR1	154,8031157	-0,186139145	0,6979963	-0,26667639	0,789718	0,999787

BOI2	278,2804135	-0,008445103	0,71991	-0,01173078	0,99064	0,999787
BPH1	55,58836227	-0,574917899	0,7760647	-0,74081179	0,458808	0,999787
BRE1	269,5546482	0,272775886	0,7447061	0,36628662	0,714151	0,999787
BRN1	108,2640913	0,764103547	0,7539135	1,013516155	0,310814	0,999787
BTS1	21,42559776	0,386725259	0,943329	0,409957987	0,681837	0,999787
BUB1	138,5778595	0,351095131	0,7213415	0,486725257	0,626453	0,999787
BUB2	17,7961817	0,260654959	0,9875653	0,263936933	0,791829	0,999787
BUD14	80,88447523	0,259787343	0,7900661	0,328817212	0,742294	0,999787
BUD16	10,17339551	0,524355864	1,3388661	0,391641761	0,695323	0,999787
BUD2	52,73126787	-0,905051206	0,8056958	-1,12331631	0,261303	0,999787
BUD31	20,49190496	0,331056041	0,9548208	0,346720603	0,728801	0,999787
BUD5	77,64572809	0,202358781	0,925779	0,218582165	0,826976	0,999787
BUD6	143,5829712	-0,339054544	0,7143372	-0,47464215	0,635042	0,999787
BUD7	79,78027186	-0,492688461	0,9253724	-0,53242181	0,594434	0,999787
BUL4	14,64428066	-0,519154283	1,0451789	-0,49671334	0,619391	0,999787
BUR2	45,96335656	0,041260259	0,7968055	0,051782094	0,958702	0,999787
_C100090WA	24,96625187	-0,085353845	0,9089485	-0,09390394	0,925185	0,999787
_C100160CA	2192,897457	0,387721048	0,6598449	0,587594199	0,556805	0,999787
_C100200CA	14,25321233	-0,960898351	1,1423016	-0,84119497	0,400239	0,999787
_C100210CA	569,2957086	-0,820511371	0,7505283	-1,09324506	0,274286	0,999787
_C100270WA	12,26115547	0,134333053	1,2269186	0,109488153	0,912815	0,999787
_C100320WA	56,78636658	-0,535314876	0,8180289	-0,65439604	0,512857	0,999787
_C100380CA	81,35470949	-0,775987815	0,7394853	-1,04936205	0,294012	0,999787
_C100410CA	77,18507104	-0,194874475	0,7437711	-0,26200868	0,793315	0,999787
_C100430WA	135,0973775	0,076945338	0,7171078	0,107299547	0,914551	0,999787
_C100450CA	162,7327604	0,322055988	0,7338558	0,438854569	0,660767	0,999787
_C100460WA	287,7304397	-0,012734647	0,7362529	-0,01729657	0,9862	0,999787
_C100470CA	65,6954903	0,05264189	0,7603753	0,069231459	0,944805	0,999787
_C100510WA	126,5406713	-0,310953535	0,7335043	-0,4239287	0,671618	0,999787
_C100520WA	83,19937504	0,002457228	0,8033814	0,003058606	0,99756	0,999787
_C100530CA	76,11746343	-0,699224236	0,7413125	-0,94322465	0,345566	0,999787
_C100570CA	89,14570251	-0,463558393	0,7502622	-0,61786186	0,536666	0,999787
_C100630WA	41,04315981	-0,794623844	0,9127517	-0,87058055	0,383983	0,999787
_C100660CA	105,1732417	-0,324897148	0,7189652	-0,45189553	0,651344	0,999787
_C100700WA	30,82571461	-0,10263104	0,9353564	-0,10972399	0,912628	0,999787
_C100760WA	88,67526443	-0,307113767	0,7687678	-0,39948833	0,689533	0,999787
_C100810WA	13,25480357	-0,391730994	1,3038973	-0,30043086	0,763849	0,999787
_C100830WA	21,21723188	0,221670763	0,9419362	0,235335228	0,813949	0,999787
_C100860WA	24,84770662	-0,573808374	0,9542572	-0,60131418	0,547631	0,999787
_C100880WA	31,65913722	-0,931838658	0,9677492	-0,96289276	0,335601	0,999787
_C100890WA	69,65980544	0,083265615	0,7825784	0,106399067	0,915266	0,999787
_C100910WA	27,94414926	0,330427612	0,885806	0,373024801	0,70913	0,999787
_C100920WA	71,21395826	-0,138137209	0,810227	-0,17049198	0,864623	0,999787
_C100970WA	18,59539417	-0,216666672	0,9865259	-0,21962594	0,826162	0,999787
_C100980WA	73,80014684	-0,152934439	0,7511806	-0,2035921	0,838672	0,999787
_C101000CA	71,85582504	-0,218449246	0,807161	-0,270639	0,786669	0,999787
_C101010WA	249,1527785	0,218475983	0,6800517	0,321263776	0,748011	0,999787
_C101040WA	41,610971	-0,735445347	0,9306896	-0,79021546	0,429402	0,999787
_C101050CA	69,59419491	0,660818812	0,8708936	0,75878247	0,447983	0,999787
_C101070CA	40,39202277	0,043992903	0,9024955	0,048745839	0,961122	0,999787

_C101080WA	27,74262601	-0,370639366	0,8825325	-0,41997249	0,674506	0,999787
_C101090CA	92,61989239	0,647565343	0,7450237	0,86918763	0,384745	0,999787
_C101130WA	174,5997812	0,118940199	0,8183375	0,145343701	0,88444	0,999787
_C101150CA	115,4971559	0,71820602	0,7341619	0,978266497	0,327943	0,999787
_C101190CA	99,73937936	-0,224889899	0,7236549	-0,31076954	0,755976	0,999787
_C101210WA	124,998376	0,832037799	0,7482556	1,111970061	0,266151	0,999787
_C101250WA	240,2155999	0,255749118	0,7008336	0,364921334	0,71517	0,999787
_C101290CA	36,66586849	-0,260437332	0,8287738	-0,31424416	0,753336	0,999787
_C101300WA	135,7632817	-0,12688029	0,7117132	-0,17827446	0,858507	0,999787
_C101340CA	20,6468525	-0,573676936	1,0187804	-0,56310166	0,573366	0,999787
_C101400CA	129,448138	0,071782415	0,748676	0,095879147	0,923617	0,999787
_C101460WA	116,7191254	0,051922547	0,7500783	0,069222837	0,944812	0,999787
_C101470WA	582,8089793	-0,205048192	0,7325404	-0,27991382	0,779544	0,999787
_C101490WA	426,863455	-0,071344237	0,6668164	-0,10699233	0,914795	0,999787
_C101530CA	734,1613561	0,76825923	0,7577991	1,013803277	0,310677	0,999787
_C101540WA	93,14460943	-0,300948225	0,729113	-0,41275936	0,679783	0,999787
_C101580WA	116,5096363	0,750638816	0,8780236	0,854918683	0,392596	0,999787
_C101590CA	758,5381837	-0,037094005	0,7281374	-0,05094369	0,95937	0,999787
_C101620CA	11,40496916	-0,974811795	1,1248126	-0,86664374	0,386137	0,999787
_C101630WA	62,04765286	-0,150887922	0,7634042	-0,19765141	0,843318	0,999787
_C101680CA	119,4301127	0,434264363	0,7157412	0,606733757	0,544028	0,999787
_C101720CA	9,524457893	-0,39658731	1,2826101	-0,30920333	0,757167	0,999787
_C101750WA	58,75708027	-0,355136615	0,8003222	-0,44374205	0,657229	0,999787
_C101800WA	59,65375351	-0,210115505	0,7699916	-0,27288024	0,784945	0,999787
_C101820CA	49,20974499	0,233893581	0,7906181	0,295836354	0,767355	0,999787
_C101850CA	70,29927374	-0,144473357	0,8317094	-0,17370654	0,862096	0,999787
_C101890CA	139,8066436	-0,876154406	0,7932915	-1,10445462	0,269396	0,999787
_C101900CA	69,82994792	-0,092870965	0,7955635	-0,11673608	0,907069	0,999787
_C101940CA	57,13232194	0,300231217	0,9258917	0,324261717	0,74574	0,999787
_C102000WA	53,12182935	0,12573513	0,7866626	0,15983361	0,873012	0,999787
_C102020WA	13,87420506	-0,116238298	1,0775469	-0,10787307	0,914096	0,999787
_C102090CA	26,93921639	0,574898108	0,9230656	0,622813925	0,533407	0,999787
_C102100WA	15,47857214	0,023650161	1,0373538	0,022798549	0,981811	0,999787
_C102160WA	16,50128575	-0,104288482	1,0181056	-0,10243385	0,918412	0,999787
_C102200CA	10,60519809	-0,264158207	1,1511245	-0,2294784	0,818497	0,999787
_C102210WA	140,9201881	-0,568611081	0,7154392	-0,79477204	0,426746	0,999787
_C102220CA	26,01961302	0,296569185	0,8990914	0,329854308	0,74151	0,999787
_C102240WA	228,6015842	0,371931041	0,8267404	0,449876438	0,6528	0,999787
_C102290CA	41,56959134	-0,817797447	0,8651825	-0,94523109	0,344541	0,999787
_C102300WA	13,43226897	-0,027680154	1,1262862	-0,02457648	0,980393	0,999787
_C102310CA	13,88539017	0,599063343	1,1535908	0,519303153	0,603549	0,999787
_C102320CA	134,2224943	0,082394613	0,8410814	0,097962708	0,921962	0,999787
_C102330CA	397,1721065	0,211999257	0,9242914	0,229364092	0,818586	0,999787
_C102370CA	72,11673413	-0,154010962	0,8776897	-0,17547314	0,860708	0,999787
_C102410CA	51,28534422	0,027057506	0,7939918	0,034077815	0,972815	0,999787
_C102430CA	307,3656599	0,224224082	0,7437609	0,301473352	0,763054	0,999787
_C102440CA	37,22861234	-0,014306615	0,8300779	-0,01723527	0,986249	0,999787
_C102470WA	9,117530605	0,152040567	1,2486878	0,121760271	0,903089	0,999787
_C102490CA	52,24969509	0,52655173	0,9331014	0,564302784	0,572548	0,999787
_C102500WA	12,18123405	-0,49053732	1,1077836	-0,4428097	0,657903	0,999787

_C102510WA	27,18745209	-0,363819341	0,9203249	-0,39531619	0,69261	0,999787
_C102650WA	464,9176565	-0,664775802	0,7209994	-0,92201991	0,356518	0,999787
_C102680CA	21,16114569	-0,957162933	0,9949948	-0,9619778	0,336061	0,999787
_C102700CA	59,08813004	-0,84689305	0,8549158	-0,99061579	0,321873	0,999787
_C102720WA	56,30135166	-0,257487485	0,813859	-0,31637849	0,751715	0,999787
_C102730WA	10,94645157	-0,727699531	1,1629884	-0,62571522	0,531502	0,999787
_C102740CA	21,3331445	-0,837308394	1,0415495	-0,80390645	0,421451	0,999787
_C102750CA	327,5092142	-0,126200702	0,7236205	-0,17440178	0,86155	0,999787
_C102760WA	375,4987657	0,12453047	0,672746	0,185107701	0,853145	0,999787
_C102770WA	93,06403366	0,103076468	0,7617768	0,135310596	0,892366	0,999787
_C102780WA	102,0660298	-0,566759158	0,7753034	-0,73101596	0,464769	0,999787
_C102830WA	64,5517323	0,24775353	0,7964534	0,311070986	0,755747	0,999787
_C102850WA	89,4872068	0,651444316	0,7351921	0,886087203	0,375571	0,999787
_C102890CA	53,21512936	-0,233767693	0,8277119	-0,28242639	0,777617	0,999787
_C102900CA	48,01383233	0,5418449	0,8203603	0,66049627	0,508935	0,999787
_C102910CA	35,67222732	-0,850085423	0,8568201	-0,99213992	0,321129	0,999787
_C102950WA	8,023923596	-1,023988199	1,3068873	-0,78353214	0,433315	0,999787
_C102960CA	119,467781	0,021791603	0,7281531	0,029927227	0,976125	0,999787
_C102970WA	291,8007096	0,432235083	0,6749325	0,640412352	0,521905	0,999787
_C103040WA	33,56926074	-0,589472831	0,8548099	-0,68959527	0,490449	0,999787
_C103050WA	86,84896899	0,5006667	0,7408844	0,675768952	0,499187	0,999787
_C103100WA	460,6973132	0,044150036	0,6658723	0,066304056	0,947136	0,999787
_C103140WA	29,78604364	-1,035167797	0,9278437	-1,1156705	0,264563	0,999787
_C103170CA	9,848318355	0,564615326	1,2129148	0,465502885	0,641571	0,999787
_C103180WA	34,43155429	-0,019728684	0,8487332	-0,02324486	0,981455	0,999787
_C103240WA	82,77183882	0,327910338	0,8118803	0,403890005	0,686294	0,999787
_C103260WA	39,80337442	-0,161120446	0,8510488	-0,18931987	0,849842	0,999787
_C103280WA	294,0400653	0,610280693	0,7059378	0,864496444	0,387315	0,999787
_C103310WA	172,5783898	-0,244247474	0,6957992	-0,35103155	0,725565	0,999787
_C103330CA	51,01068151	-0,463413888	0,8082475	-0,57335641	0,566403	0,999787
_C103360WA	485,8924695	-0,558092277	0,6756611	-0,82599438	0,408807	0,999787
_C103370WA	4777,025126	0,234488697	0,6593473	0,355637618	0,722112	0,999787
_C103400WA	103,1318768	-0,766190938	0,7329297	-1,04538126	0,295847	0,999787
_C103410WA	13,59179546	-0,866389992	1,1500454	-0,75335288	0,451238	0,999787
_C103430WA	52,83227854	-0,586398257	0,8506556	-0,68934859	0,490604	0,999787
_C103440CA	178,7608736	0,469982364	0,7117968	0,660276015	0,509077	0,999787
_C103450CA	229,0943048	-0,760098251	0,6919136	-1,09854504	0,271967	0,999787
_C103460CA	63,24565125	-0,024156082	0,7647514	-0,03158684	0,974802	0,999787
_C103490WA	206,7578194	-0,978427512	0,8573352	-1,14124265	0,253769	0,999787
_C103540CA	32,07949352	0,454198071	0,8720052	0,520866226	0,60246	0,999787
_C103600WA	89,3477761	0,3180509	0,7462753	0,426184406	0,669973	0,999787
_C103620CA	212,119034	0,753889053	0,7515421	1,003122911	0,315802	0,999787
_C103630WA	68,14075618	-0,037951503	0,7546407	-0,05029082	0,959891	0,999787
_C103720CA	108,5513186	-0,380229348	0,7300754	-0,52080832	0,6025	0,999787
_C103790CA	1715,457445	0,536527847	0,6553689	0,818665358	0,412977	0,999787
_C103830CA	77,75000609	0,99971519	0,927097	1,078328635	0,280887	0,999787
_C103840WA	26,82961711	-0,146187945	0,8894113	-0,16436485	0,869444	0,999787
_C103910CA	52,49719673	0,02507305	0,8041679	0,031178874	0,975127	0,999787
_C103920CA	8,899260755	-0,472219412	1,2209378	-0,38676779	0,698928	0,999787
_C103930WA	36,33803925	0,6937568	0,9203212	0,753820266	0,450957	0,999787

_C103940WA	174,0095705	0,133861546	0,8570493	0,156188852	0,875884	0,999787
_C103950CA	8,050638608	-0,248785394	1,2633058	-0,19693205	0,843881	0,999787
_C103960CA	32,79603481	-0,247876647	0,8887408	-0,2789077	0,780316	0,999787
_C103990WA	22,85426896	0,074202418	1,0428544	0,071153188	0,943276	0,999787
_C104030WA	63,56879377	0,243465937	0,7895798	0,308348729	0,757817	0,999787
_C104120CA	95,59213106	-0,129474128	0,7799701	-0,16599883	0,868158	0,999787
_C104180WA	851,8983221	-0,322900383	0,7003926	-0,46102768	0,644779	0,999787
_C104190CA	42,83907579	-0,559660545	0,81928	-0,68311264	0,494536	0,999787
_C104220CA	36,66239387	0,450866292	0,8551187	0,52725577	0,598016	0,999787
_C104230WA	184,743724	-0,01769565	0,6976078	-0,02536619	0,979763	0,999787
_C104280CA	10,17484768	-0,251543047	1,1687101	-0,21523135	0,829587	0,999787
_C104340CA	14,40057116	-0,294189012	1,0535576	-0,27923393	0,780065	0,999787
_C104360CA	300,8663097	-0,42387846	0,6736485	-0,62922795	0,5292	0,999787
_C104370CA	105,3773201	0,327682831	0,8779836	0,373222051	0,708983	0,999787
_C104470CA	27,28507236	-0,11608679	1,0185803	-0,1139692	0,909262	0,999787
_C104490WA	503,8708863	0,413715282	0,7153983	0,578300613	0,563061	0,999787
_C104510WA	21,48884394	1,059154993	0,9910226	1,068749551	0,285183	0,999787
_C104530CA	113,1330739	0,571147528	0,7554221	0,756064102	0,449611	0,999787
_C104560WA	23,18860368	0,136568686	0,9353409	0,146009528	0,883914	0,999787
_C104580CA	49,75254627	-0,313584429	0,8059732	-0,38907551	0,69722	0,999787
_C104590WA	113,1739644	-0,058228289	0,9368779	-0,06215142	0,950442	0,999787
_C104620WA	256,4549179	-0,423475978	0,7473045	-0,56667125	0,570938	0,999787
_C104630CA	97,34009435	0,751812288	0,7313831	1,027932203	0,303982	0,999787
_C104640WA	150,9664254	0,170284604	0,6983737	0,243830202	0,807362	0,999787
_C104700CA	35,80951707	-0,226720383	0,8570626	-0,26453187	0,79137	0,999787
_C104720WA	8,084683481	-0,478695166	1,3348094	-0,35862435	0,719876	0,999787
_C104780CA	58,29703423	-0,26845713	0,8152588	-0,32929071	0,741936	0,999787
_C104790WA	160,279502	-0,146417864	0,762941	-0,19191245	0,847811	0,999787
_C104820CA	37,86079743	0,750520447	0,9511275	0,789084968	0,430062	0,999787
_C104840CA	14,05618498	-0,648262402	1,0615067	-0,61070024	0,541398	0,999787
_C104860WA	93,44964909	-0,377548494	0,744429	-0,50716521	0,612039	0,999787
_C104910CA	73,0550108	0,137466299	0,7565679	0,181697251	0,85582	0,999787
_C104930CA	5,709117691	0,565061908	1,4649307	0,385726031	0,6997	0,999787
_C104970WA	376,3770254	-0,253314977	0,6796308	-0,3727244	0,709354	0,999787
_C104980CA	95,37582964	0,381702084	0,8382009	0,455382562	0,648834	0,999787
_C104990CA	80,16373318	0,295599326	0,7697253	0,384032223	0,700955	0,999787
_C105010CA	22,08541598	-0,311583012	0,9317325	-0,3344125	0,738068	0,999787
_C105100WA	16,33798591	-0,28703392	1,0302006	-0,27861945	0,780537	0,999787
_C105120WA	38,33084912	-0,704154636	0,8469621	-0,83138864	0,405754	0,999787
_C105180CA	49,54021262	0,113077203	0,8843468	0,127865226	0,898256	0,999787
_C105190CA	54,46791026	0,217560046	0,8838015	0,246163932	0,805555	0,999787
_C105200CA	39,77036468	-0,160675544	0,8436886	-0,19044412	0,848961	0,999787
_C105220CA	6,752027971	0,127552038	1,3692768	0,093152849	0,925782	0,999787
_C105230WA	21,73592738	1,154975673	1,0196864	1,132677288	0,25735	0,999787
_C105280WA	36,07358141	0,043661391	0,8472914	0,051530548	0,958903	0,999787
_C105320CA	26,12972984	-0,40266859	0,8890016	-0,45294472	0,650589	0,999787
_C105370CA	43,54256655	-0,124125589	0,8091125	-0,15340956	0,878075	0,999787
_C105390CA	122,1604639	0,096194239	0,7099359	0,135497084	0,892219	0,999787
_C105400CA	26,34937573	-0,921005639	0,9372388	-0,98267976	0,325765	0,999787
_C105420WA	36,91240502	-0,499532811	0,8568274	-0,58300286	0,559891	0,999787

_C105490CA	148,6406636	-0,264390672	0,7107729	-0,3719763	0,70991	0,999787
_C105520WA	11,43836762	-1,13098661	1,1529782	-0,98092631	0,326629	0,999787
_C105610WA	70,35500141	0,681125071	0,7885804	0,863735711	0,387733	0,999787
_C105630CA	1113,37028	0,447318467	0,9508981	0,470416813	0,638057	0,999787
_C105650WA	36,48074301	0,510841012	0,8675313	0,588844484	0,555966	0,999787
_C105660CA	326,9454956	-0,165299154	0,8429034	-0,19610688	0,844527	0,999787
_C105670WA	129,3775125	-0,702026752	0,7270379	-0,96559862	0,334245	0,999787
_C105720WA	3501,383271	0,232994014	0,6607623	0,352613991	0,724378	0,999787
_C105740CA	9,932678165	-0,906216415	1,2405057	-0,73052174	0,465071	0,999787
_C105750CA	122,9121651	0,254920704	0,7171918	0,355442869	0,722258	0,999787
_C105780WA	122,641136	-0,198655309	0,7437197	-0,26711047	0,789384	0,999787
_C105790WA	44,80823665	0,581162215	0,8435934	0,688912677	0,490878	0,999787
_C105800CA	14,90858624	-0,117216909	1,0354897	-0,1131995	0,909872	0,999787
_C105900WA	77,03831285	-0,030709645	0,8414322	-0,03649687	0,970886	0,999787
_C105930CA	75,43806563	-0,138725951	0,8176253	-0,16966935	0,86527	0,999787
_C105950CA	85,68210639	0,429182661	0,8388847	0,511611054	0,608923	0,999787
_C105980WA	29,51101663	-0,588345975	0,8851757	-0,66466574	0,506264	0,999787
_C105990CA	22,7056992	-0,114957801	0,9590038	-0,11987211	0,904584	0,999787
_C106000WA	7,98649309	-0,192176488	1,3838913	-0,13886675	0,889555	0,999787
_C106030CA	6,083287163	0,735782937	1,4408924	0,51064392	0,6096	0,999787
_C106050CA	169,6464052	-0,590085816	0,6951751	-0,84883044	0,395976	0,999787
_C106070WA	83,00168349	0,279110163	0,7905019	0,353079683	0,724029	0,999787
_C106090CA	75,23008155	0,133623451	0,8925702	0,149706382	0,880996	0,999787
_C106200WA	68,38461217	0,243426508	0,7624628	0,319263437	0,749527	0,999787
_C106250WA	11,99707943	0,103232795	1,1154559	0,092547625	0,926263	0,999787
_C106270WA	28,63220486	0,172473017	0,9522071	0,181129738	0,856266	0,999787
_C106320WA	40,11242968	-0,595281622	0,8862465	-0,67168851	0,501782	0,999787
_C106350WA	83,48260986	-0,623473676	0,7466826	-0,83499159	0,403722	0,999787
_C106360WA	30,76270389	-0,554683181	0,859716	-0,64519352	0,518802	0,999787
_C106380CA	32,00488659	-0,288275609	0,9286539	-0,31042309	0,756239	0,999787
_C106390WA	16,46828194	-0,461368751	1,0189153	-0,45280381	0,65069	0,999787
_C106420CA	53,80401894	-0,140520437	0,8002232	-0,17560156	0,860607	0,999787
_C106440CA	46,64978319	0,659240374	1,0484473	0,628777816	0,529495	0,999787
_C106470WA	1574,084527	0,03555088	0,6541566	0,054346128	0,956659	0,999787
_C106480CA	80,73419573	-0,698283955	0,8124267	-0,85950393	0,390063	0,999787
_C106500WA	23,57774447	-0,017510617	0,9213569	-0,01900525	0,984837	0,999787
_C106510CA	75,98551881	0,504907446	0,7828766	0,644938769	0,518967	0,999787
_C106530CA	203,0631719	0,394583909	0,7657237	0,515308468	0,606337	0,999787
_C106540CA	41,85479058	1,039931621	0,9720431	1,069841051	0,284691	0,999787
_C106560WA	88,56543678	-0,890105001	0,8165659	-1,09005899	0,275687	0,999787
_C106590CA	347,9936183	0,492010448	0,673203	0,730850035	0,464871	0,999787
_C106600WA	1051,888955	-0,717317938	0,7346389	-0,97642253	0,328855	0,999787
_C106640CA	58,82620399	-0,052610099	0,8383678	-0,06275301	0,949963	0,999787
_C106670WA	109,705716	-0,711108338	0,7681667	-0,92572135	0,354591	0,999787
_C106700WA	130,4612238	0,392031783	0,7482316	0,523944447	0,600317	0,999787
_C106710WA	80,57481673	-0,498362182	0,7593688	-0,6562848	0,511641	0,999787
_C106720CA	44,31015009	-0,371683036	0,8105248	-0,45857081	0,646542	0,999787
_C106750WA	15,22715099	-0,828709989	1,1054038	-0,74968985	0,453442	0,999787
_C106820WA	104,757544	-0,227891235	0,7178168	-0,31747826	0,750881	0,999787
_C106840CA	75,50014205	-0,412562055	0,7751847	-0,53221133	0,59458	0,999787

_C106870CA	7,546009162	-1,221337229	1,308434	-0,93343434	0,350596	0,999787
_C106890CA	2937,152676	0,147084977	0,6547441	0,224644992	0,822255	0,999787
_C106910CA	127,6680328	0,446199475	0,7073426	0,630811001	0,528164	0,999787
_C106920CA	51,62783794	-0,681223371	0,8294698	-0,82127569	0,411489	0,999787
_C106980CA	96,07537862	-0,699909914	0,8163375	-0,85737818	0,391236	0,999787
_C107010WA	39,69204667	0,424058054	0,8320261	0,509669162	0,610283	0,999787
_C107050CA	47,65986546	0,41516981	0,7959968	0,521572226	0,601968	0,999787
_C107060CA	24,42918773	-0,115803655	0,9290985	-0,12464089	0,900808	0,999787
_C107090CA	167,5028614	0,229902817	0,7424185	0,309667424	0,756814	0,999787
_C107130CA	182,985938	-0,404941321	0,7952046	-0,50922913	0,610592	0,999787
_C107150WA	20,55057796	0,453162795	0,9597647	0,472160314	0,636812	0,999787
_C107210CA	113,0799588	-0,017312792	0,7442946	-0,02326067	0,981442	0,999787
_C107220WA	39,81875768	-0,811978034	0,9210135	-0,88161362	0,377986	0,999787
_C107260CA	120,6707646	-0,652931811	0,7571175	-0,86239164	0,388472	0,999787
_C107280CA	17,20197285	0,074327072	0,9922359	0,074908669	0,940287	0,999787
_C107320WA	12,07926454	-1,290745163	1,4111044	-0,91470566	0,360346	0,999787
_C107340WA	432,6176668	0,119213419	0,6839796	0,174293826	0,861635	0,999787
_C107360WA	127,8382768	-0,026652603	0,7950351	-0,03352381	0,973257	0,999787
_C107390WA	162,8971211	-0,052891992	0,7251004	-0,07294437	0,94185	0,999787
_C107440WA	157,2525331	-0,720594212	0,7101896	-1,01465049	0,310272	0,999787
_C107470CA	31,71345257	0,706928204	0,9254458	0,763878575	0,44494	0,999787
_C107490CA	35,54625138	0,021384325	1,079042	0,019817881	0,984189	0,999787
_C107510WA	24,96930252	0,135647222	0,9645717	0,140629485	0,888163	0,999787
_C107560WA	20,99494168	-0,28169297	0,9523761	-0,29577913	0,767399	0,999787
_C107570CA	10,54844687	0,811999903	1,174459	0,691382059	0,489325	0,999787
_C107590CA	46,23999474	-0,529583517	0,8514223	-0,62199865	0,533943	0,999787
_C107610CA	5,448197743	-1,388652351	1,4951049	-0,92879926	0,352993	0,999787
_C107630WA	91,10987315	0,083460579	0,7919266	0,105389279	0,916067	0,999787
_C107640CA	7,603718392	-1,540712678	1,4548885	-1,05899019	0,289604	0,999787
_C107650WA	8,244292023	-1,578091288	1,4035268	-1,1243756	0,260854	0,999787
_C107660WA	15,56871013	-0,153058917	1,0577882	-0,14469713	0,88495	0,999787
_C107690CA	56,22085995	-0,208221204	0,8610975	-0,24180909	0,808928	0,999787
_C107810CA	25,21147796	0,192882768	0,9046293	0,213217478	0,831157	0,999787
_C107820WA	28,34527498	0,436506904	0,8894191	0,490777518	0,623584	0,999787
_C107830CA	20,74793437	0,036455267	0,9528221	0,038260309	0,96948	0,999787
_C107840WA	117,9103534	0,064028905	0,7674489	0,083430842	0,933509	0,999787
_C107850CA	20,00393313	0,358481325	0,9826152	0,364823728	0,715243	0,999787
_C107860WA	5,261391722	0,476976097	1,6586391	0,287570748	0,773675	0,999787
_C107920WA	82,8437273	-0,246441386	0,7563379	-0,32583503	0,744549	0,999787
_C107980CA	26,58052408	-0,76944426	0,9249478	-0,8318786	0,405477	0,999787
_C108050WA	409,4722579	-0,390889818	0,6830044	-0,57230937	0,567112	0,999787
_C108080CA	217,6516301	0,179071195	0,6865637	0,260822421	0,794229	0,999787
_C108110WA	596,0075877	0,500450895	0,862781	0,58004397	0,561885	0,999787
_C108140WA	48,70690269	-0,314641362	0,8703979	-0,36149143	0,717732	0,999787
_C108160WA	200,2669174	-0,785327808	0,7792018	-1,00786187	0,313521	0,999787
_C108180CA	397,8313169	-0,040087976	0,6824071	-0,05874496	0,953155	0,999787
_C108340CA	63,74603789	-0,549105872	0,8184162	-0,67093719	0,502261	0,999787
_C108390CA	169,8046953	-0,287652729	0,737303	-0,39014182	0,696432	0,999787
_C108440CA	8,941998671	-0,0586322	1,2962216	-0,04523316	0,963921	0,999787
_C108470WA	90,62018006	-0,625478697	0,8225929	-0,76037455	0,447031	0,999787

_C108490WA	186,8771016	-0,317921429	0,7032883	-0,45204994	0,651233	0,999787
_C108520CA	94,52493804	0,683692745	0,7964568	0,858417867	0,390662	0,999787
_C108540CA	374,5596787	0,046659415	0,6725934	0,069372399	0,944693	0,999787
_C108610CA	506,3623011	0,463547165	0,782085	0,592706866	0,553377	0,999787
_C108660CA	39,09743265	0,246373663	0,8460585	0,291201685	0,770897	0,999787
_C108680CA	86,16097418	-0,567298412	0,7990774	-0,70994173	0,47774	0,999787
_C108690WA	55,4384809	0,0185956	0,7791037	0,02386794	0,980958	0,999787
_C108700WA	417,87306	0,131862629	0,6705058	0,196661423	0,844093	0,999787
_C108710WA	50,28517778	0,040204401	0,796492	0,05047684	0,959742	0,999787
_C108730WA	81,56289756	-0,190795132	0,7451394	-0,25605295	0,79791	0,999787
_C108770WA	30,45638886	-0,799761814	0,9173556	-0,87181219	0,383311	0,999787
_C108820CA	13,40466631	-0,101292625	1,1075709	-0,09145475	0,927131	0,999787
_C108840WA	8,032528821	-0,101941698	1,3124646	-0,07767196	0,938089	0,999787
_C108860CA	68,53408127	0,101787248	0,7517085	0,135407868	0,892289	0,999787
_C108900WA	13,50752939	-0,234288829	1,0802548	-0,21688293	0,8283	0,999787
_C108910CA	13,00635002	0,353099453	1,086255	0,325061306	0,745135	0,999787
_C108920WA	79,1764637	-0,120817742	0,8911589	-0,13557374	0,892158	0,999787
_C108930CA	68,09368516	-0,00877335	0,7554904	-0,01161279	0,990735	0,999787
_C108960WA	16,57893872	0,650099946	1,0188583	0,638067054	0,52343	0,999787
_C108970WA	174,2030502	0,003045226	0,8360319	0,003642476	0,997094	0,999787
_C109000WA	28,85702353	-1,188570523	1,0790604	-1,10148652	0,270685	0,999787
_C109020WA	164,9868601	0,291224565	0,7934903	0,367017151	0,713606	0,999787
_C109040CA	60,61798165	0,727627504	0,7737521	0,940388382	0,347018	0,999787
_C109060CA	6,910666815	-0,969319883	1,3842161	-0,70026627	0,483761	0,999787
_C109070WA	46,11741153	-0,556801959	0,9161726	-0,60774791	0,543355	0,999787
_C109110WA	7,348664447	-1,411885405	1,3233782	-1,06687971	0,286026	0,999787
_C109130WA	23,17661282	0,030686735	0,9543557	0,032154402	0,974349	0,999787
_C109210CA	69,7011342	-0,781291922	0,9445949	-0,82711854	0,40817	0,999787
_C109220WA	57,26050189	-0,707028556	0,8465372	-0,83520085	0,403605	0,999787
_C109280WA	76,2533773	-0,193717243	0,7420178	-0,26106818	0,79404	0,999787
_C109310CA	9,207756418	-0,292822384	1,2150101	-0,24100408	0,809552	0,999787
_C109320CA	154,5927701	-0,521771333	0,8573914	-0,60855675	0,542818	0,999787
_C109340CA	21,79349605	-0,204437121	1,0008502	-0,20426346	0,838148	0,999787
_C109360CA	175,0095731	-0,382446305	0,7006485	-0,54584616	0,585172	0,999787
_C109430WA	66,48761875	0,231200944	0,808578	0,285935235	0,774928	0,999787
_C109440WA	42,16968858	-0,478417785	0,8386711	-0,57044743	0,568374	0,999787
_C109470CA	90,35645301	0,685656815	0,7560604	0,906881021	0,36447	0,999787
_C109520CA	68,19838812	0,112592551	0,7545263	0,149222829	0,881378	0,999787
_C109560CA	6,508406292	0,520414212	1,3843522	0,375926161	0,706972	0,999787
_C109610WA	67,06415463	-0,701951438	0,7778419	-0,9024346	0,366826	0,999787
_C109620CA	38,20028255	0,164074104	0,8972998	0,18285315	0,854913	0,999787
_C109670CA	33,8483783	-0,32373242	0,8483019	-0,38162405	0,70274	0,999787
_C109740CA	5,583889001	-0,133249515	1,4614804	-0,09117435	0,927354	0,999787
_C109750WA	67,65744303	0,765192533	0,8111495	0,943343388	0,345505	0,999787
_C109770WA	20,24788979	-0,240314548	1,0431024	-0,23038442	0,817793	0,999787
_C109780CA	49,09645542	-0,80211714	0,8152219	-0,98392486	0,325152	0,999787
_C109790CA	139,2627746	0,561408344	0,7425411	0,756063714	0,449611	0,999787
_C109810WA	62,27429147	0,354568088	0,7882347	0,449825513	0,652836	0,999787
_C109840CA	44,50008289	-0,249890372	0,8064814	-0,30985261	0,756673	0,999787
_C109850CA	180,7420563	-0,565473995	0,7186959	-0,78680564	0,431396	0,999787

_C109910CA	32,93212432	0,838190968	0,8781406	0,954506617	0,339827	0,999787
_C109950CA	52,28045517	-0,064982508	0,7994785	-0,08128112	0,935218	0,999787
_C109980CA	34,18857426	-0,411971062	0,8790888	-0,46863417	0,639331	0,999787
_C110080WA	32,92578278	-0,030525896	0,8518437	-0,03583509	0,971414	0,999787
_C110090CA	200,0182995	-0,309920588	0,698106	-0,44394488	0,657082	0,999787
_C110110WA	43,21284602	-0,780278221	0,9355893	-0,83399648	0,404283	0,999787
_C110120CA	273,7763003	-0,595151447	0,8085271	-0,73609339	0,461674	0,999787
_C110190WA	84,75953658	-0,187613024	0,769769	-0,24372641	0,807443	0,999787
_C110200CA	68,38742259	0,119669458	0,874772	0,136800737	0,891188	0,999787
_C110230CA	110,5534432	-0,391440459	0,7162393	-0,54652192	0,584707	0,999787
_C110280CA	177,1052644	0,166637978	0,7675161	0,217113344	0,82812	0,999787
_C110310WA	8,205574459	-1,328894063	1,321908	-1,00528481	0,31476	0,999787
_C110320WA	14,39220717	-1,078813314	1,0623438	-1,01550301	0,309866	0,999787
_C110330CA	20,2892586	-0,446881059	1,0354546	-0,43157958	0,666047	0,999787
_C110340WA	11,61438086	0,137662741	1,1177895	0,12315623	0,901983	0,999787
_C110350CA	187,546127	0,606151543	0,7773449	0,779771704	0,435525	0,999787
_C110410WA	28,323639	-0,366553667	0,8942439	-0,40990347	0,681877	0,999787
_C110420CA	82,87928317	-0,123864873	0,7766097	-0,15949437	0,873279	0,999787
_C110470WA	252,9740645	0,739324405	0,7267426	1,017312544	0,309005	0,999787
_C110520WA	7,769827656	0,316738898	1,2899399	0,245545478	0,806034	0,999787
_C110540CA	93,68380145	-0,699174881	0,7518128	-0,92998539	0,352379	0,999787
_C110560CA	9,361798696	-0,199954607	1,2352106	-0,16187895	0,871401	0,999787
_C110580CA	9,530976234	0,16607436	1,215624	0,136616555	0,891334	0,999787
_C110620WA	1163,603711	0,733238026	0,7603345	0,964362383	0,334864	0,999787
_C110630CA	22,85209426	0,058829295	0,9788821	0,06009845	0,952077	0,999787
_C110680CA	602,6279494	-0,379373887	0,7195842	-0,52721264	0,598046	0,999787
_C110690WA	94,0214961	-0,04203298	0,7576356	-0,05547915	0,955757	0,999787
_C110710CA	33,9829476	-0,668400591	0,8401005	-0,79561984	0,426253	0,999787
_C110730WA	110,1336434	-0,409229779	0,7351686	-0,55664749	0,577768	0,999787
_C110820CA	101,8817343	-0,724503686	0,7293609	-0,9933404	0,320544	0,999787
_C110840CA	1130,322038	0,199927579	0,6633015	0,301412822	0,7631	0,999787
_C110890CA	74,2625958	0,185859085	0,7447356	0,249563862	0,802925	0,999787
_C110920WA	52,38704579	-0,910562426	0,8133738	-1,11948826	0,262932	0,999787
_C111020WA	22,75222332	-0,678485634	0,9543331	-0,71095265	0,477114	0,999787
_C111080WA	135,7971762	-0,502663609	0,9343297	-0,53799384	0,590581	0,999787
_C111090CA	138,8071108	-0,056409531	0,723356	-0,07798308	0,937842	0,999787
_C111100WA	22,43535081	0,15770972	0,9522757	0,165613502	0,868461	0,999787
_C111110CA	22,59382455	0,400698166	0,9473601	0,4229629	0,672322	0,999787
_C111120CA	108,5112072	-0,008750392	0,8058355	-0,01085878	0,991336	0,999787
_C111140WA	50,55393344	-0,479828406	0,8008347	-0,59916033	0,549066	0,999787
_C111150WA	60,13096303	-0,644362019	0,8455302	-0,76208041	0,446012	0,999787
_C111250WA	65,9027965	0,784516454	0,7751874	1,012034604	0,311522	0,999787
_C111280WA	59,89962734	-0,492303422	0,8404319	-0,58577432	0,558027	0,999787
_C111290WA	18,1785746	-0,620042295	1,0751909	-0,57668113	0,564155	0,999787
_C111300CA	76,96549667	-0,131754341	0,7441528	-0,17705281	0,859467	0,999787
_C111370CA	28,27114936	0,083508744	0,8865187	0,094198511	0,924951	0,999787
_C111410CA	5,605131496	1,379815087	1,5938878	0,865691497	0,386659	0,999787
_C111430WA	36,37368854	0,932492438	1,1246389	0,829148272	0,407021	0,999787
_C111510CA	50,31561341	-0,149022615	0,8787951	-0,16957607	0,865344	0,999787
_C111530CA	6,673880836	1,134552712	1,4791277	0,767041767	0,443057	0,999787

_C111560CA	48,71774589	0,6914081	0,8056854	0,858161378	0,390803	0,999787
_C111580WA	92,4434214	0,098505796	0,8102885	0,121568789	0,903241	0,999787
_C111610CA	80,32365115	0,841445315	0,8391094	1,002783771	0,315965	0,999787
_C111620WA	10,64544395	-0,353068609	1,215598	-0,29044849	0,771473	0,999787
_C111680CA	7,187533541	-1,059782482	1,3798797	-0,76802526	0,442472	0,999787
_C111690WA	73,14704831	-0,784310852	0,7835694	-1,00094619	0,316853	0,999787
_C111720WA	35,21537265	0,220291034	0,9781497	0,225211986	0,821814	0,999787
_C111730WA	333,4483521	0,663058456	0,7458688	0,888974687	0,374017	0,999787
_C111740WA	49,40347453	0,219633328	0,788562	0,278523858	0,78061	0,999787
_C111760CA	96,3840216	-0,601656489	0,7229178	-0,83226128	0,405261	0,999787
_C111770CA	268,4938158	0,50153504	0,7129231	0,703491093	0,48175	0,999787
_C111780WA	5,394832152	0,084392136	1,6244282	0,051951903	0,958567	0,999787
_C111790WA	21,28016647	0,405755426	1,0923315	0,37145813	0,710296	0,999787
_C111860WA	765,0501414	0,038168734	0,8206188	0,046512137	0,962902	0,999787
_C111880WA	62,58943534	0,757947487	0,8793821	0,861909186	0,388737	0,999787
_C111900CA	77,22279568	0,029661922	0,7810148	0,037978692	0,969705	0,999787
_C111920WA	119,7788283	-0,537214388	0,73714	-0,72878206	0,466135	0,999787
_C111930WA	293,967978	-0,686243395	0,6774065	-1,01304514	0,311039	0,999787
_C111940CA	15,47752632	-0,515953544	1,0876877	-0,47435818	0,635245	0,999787
_C111960CA	25,5639294	-0,808360168	0,9069947	-0,89125128	0,372794	0,999787
_C111990WA	121,7162498	-0,714485231	0,7110024	-1,00489842	0,314946	0,999787
_C112120WA	38,40392299	-0,48328721	0,8618175	-0,56077674	0,57495	0,999787
_C112170CA	35,3707313	-0,337014895	0,8444708	-0,39908412	0,689831	0,999787
_C112180CA	49,49433506	0,340448169	0,8076248	0,421542467	0,673359	0,999787
_C112240CA	46,7623863	0,463930824	0,9062902	0,511900972	0,60872	0,999787
_C112250CA	135,7515952	0,15683764	0,8386608	0,187009631	0,851653	0,999787
_C112350WA	152,8499823	0,120276035	0,8845843	0,135968993	0,891846	0,999787
_C112370WA	16,87312234	-0,795065971	1,0226059	-0,77749012	0,43687	0,999787
_C112400CA	40,11283386	-0,003555907	0,8244056	-0,0043133	0,996558	0,999787
_C112430WA	60,97133243	0,069831586	0,7725063	0,09039614	0,927972	0,999787
_C112530CA	19,94373184	0,200838829	0,9584972	0,209535132	0,834031	0,999787
_C112540WA	10,25654439	-0,413748729	1,2199765	-0,33914482	0,734501	0,999787
_C112580WA	46,33994196	-0,003099072	0,8009579	-0,00386921	0,996913	0,999787
_C112610WA	77,89786024	0,591523843	0,9052985	0,653402026	0,513497	0,999787
_C112630CA	16,76340494	0,899481437	1,0660862	0,843723019	0,398824	0,999787
_C112660WA	16,52204101	-0,048450528	1,1492566	-0,04215815	0,966373	0,999787
_C112670CA	12,96240117	0,370294614	1,0976213	0,337361001	0,735845	0,999787
_C112710CA	56,3707227	-0,164261108	0,784904	-0,20927541	0,834233	0,999787
_C112750CA	18,9538581	0,223372528	0,9761055	0,228840553	0,818993	0,999787
_C112760WA	64,66383533	0,495771245	0,9193471	0,53926451	0,589704	0,999787
_C112820CA	59,35948784	0,135245554	0,7986872	0,169334826	0,865533	0,999787
_C112830CA	39,19692279	-0,438912398	0,9289579	-0,47247823	0,636585	0,999787
_C112840WA	6,251177644	0,514789201	1,5310857	0,336224944	0,736701	0,999787
_C112860CA	18,52843331	-0,491543424	0,9889564	-0,49703247	0,619166	0,999787
_C112870CA	6,328766186	-0,37576917	1,3947384	-0,2694191	0,787607	0,999787
_C112880CA	24,50638279	-0,967749285	0,9253428	-1,04582788	0,295641	0,999787
_C112900WA	41,30094112	-0,608093617	0,8132027	-0,74777617	0,454595	0,999787
_C112910WA	6,357338541	-0,192018124	1,5360049	-0,1250114	0,900515	0,999787
_C112920CA	19,3937892	0,256996106	1,0190985	0,25217985	0,800902	0,999787
_C112930CA	10,91884891	-0,905185684	1,2247686	-0,73906671	0,459866	0,999787

_C112950WA	43,31938854	-0,244732878	0,8646756	-0,28303433	0,777151	0,999787
_C112990WA	15,67405951	0,382342249	1,0404291	0,367485159	0,713257	0,999787
_C113010WA	87,70425797	0,362606258	0,9771148	0,371098931	0,710564	0,999787
_C113030CA	434,9337342	0,868620919	0,8691013	0,999447271	0,317578	0,999787
_C113060CA	452,9967867	0,605329116	0,9742085	0,621354805	0,534366	0,999787
_C113190WA	75,87736085	0,025606534	0,8421784	0,03040512	0,975744	0,999787
_C113250WA	126,111427	-0,285339927	0,8853772	-0,32228065	0,74724	0,999787
_C113260WA	171,1099134	-0,434652838	0,8254694	-0,52655236	0,598504	0,999787
_C113270WA	17,63441015	0,110809902	0,9942453	0,111451266	0,911259	0,999787
_C113280CA	94,29229187	0,132415089	0,7260069	0,182388197	0,855278	0,999787
_C113320CA	205,1380598	-0,595652877	0,7400188	-0,80491585	0,420868	0,999787
_C113330CA	88,45504349	-0,222363366	0,7372791	-0,30159999	0,762957	0,999787
_C113370WA	38,58040008	0,578299494	0,8786258	0,658186362	0,510418	0,999787
_C113380WA	14,07333099	-1,104943029	1,1452498	-0,96480523	0,334642	0,999787
_C113390WA	10,62636446	-0,258505641	1,1488149	-0,2250194	0,821964	0,999787
_C113490CA	18,17261486	-0,887568417	0,9832176	-0,90271822	0,366675	0,999787
_C113530WA	27,5654839	-0,588042037	0,8857205	-0,66391378	0,506745	0,999787
_C113560WA	17,32228762	0,494562593	1,0646559	0,46452811	0,642269	0,999787
_C113650CA	32,90776198	-0,697007806	0,8595093	-0,81093693	0,417402	0,999787
_C113750CA	22,09725459	-1,094669753	1,0022377	-1,0922257	0,274734	0,999787
_C113790CA	37,81870709	0,084506347	0,8569212	0,098616246	0,921443	0,999787
_C113840WA	111,5376677	-0,150750082	0,7216651	-0,20889203	0,834533	0,999787
_C113880CA	166,2966124	-0,245042381	0,7516897	-0,32598872	0,744433	0,999787
_C113950CA	5,183321876	-0,706407913	1,5503598	-0,45564126	0,648648	0,999787
_C114030WA	35,48372213	-0,04696035	0,9214987	-0,05096084	0,959357	0,999787
_C114040WA	83,53912462	-0,52933855	0,7364248	-0,7187951	0,472267	0,999787
_C114050CA	103,0403082	0,245135001	0,7597095	0,322669381	0,746946	0,999787
_C114060WA	214,8849929	0,451405328	0,7290019	0,619210046	0,535778	0,999787
_C114090WA	332,218369	0,659117232	0,6856956	0,96123878	0,336432	0,999787
_C114170WA	222,0721023	0,575870212	0,6854131	0,840179726	0,400808	0,999787
_C114200WA	72,14017964	-0,132861834	0,7533478	-0,17636188	0,86001	0,999787
_C114240WA	312,6826294	-0,025499317	0,7154154	-0,03564267	0,971567	0,999787
_C114250CA	179,6190835	-0,731691985	0,7629892	-0,95898074	0,337568	0,999787
_C114270WA	20,96668669	-0,109443096	0,9461689	-0,11566973	0,907914	0,999787
_C114310WA	140,3365184	-0,595658408	0,7967727	-0,74758887	0,454708	0,999787
_C114320CA	153,526612	-0,536053089	0,7431519	-0,7213237	0,47071	0,999787
_C114380CA	80,32669704	0,71652987	0,7440659	0,962992446	0,335551	0,999787
_C114410WA	232,8951146	-0,952351233	0,8429817	-1,12974123	0,258585	0,999787
_C114450CA	783,4063806	-0,108042279	0,6950333	-0,15544906	0,876467	0,999787
_C114460WA	981,7988557	-0,808977924	0,8080719	-1,00112123	0,316768	0,999787
_C114550CA	23,24020091	-0,926359218	0,9258627	-1,00053631	0,317051	0,999787
_C114560CA	108,3901361	-0,865105964	0,7643195	-1,1318643	0,257691	0,999787
_C114580CA	80,96935868	-0,528223693	0,8465371	-0,62398174	0,53264	0,999787
_C200060CA	424,8742289	-0,608755143	0,7349048	-0,8283456	0,407475	0,999787
_C200090WA	81,7612296	-0,408338804	0,8473928	-0,48187664	0,629894	0,999787
_C200110WA	64,19658974	-0,296510312	0,8613024	-0,34425809	0,730652	0,999787
_C200180CA	27,31575498	-0,791112752	0,9882924	-0,80048449	0,42343	0,999787
_C200190CA	200,6631307	0,052342602	0,7673845	0,068209099	0,945619	0,999787
_C200200WA	68,17539373	-0,214059921	0,8335982	-0,2567903	0,797341	0,999787
_C200220CA	222,1784436	-0,223822973	0,7784303	-0,28753118	0,773706	0,999787

_C200230WA	61,37651258	0,053548248	0,8093972	0,066158181	0,947252	0,999787
_C200290WA	64,12157054	-0,297633167	0,8132479	-0,36598084	0,714379	0,999787
_C200320WA	67,56764234	0,009232015	0,7762344	0,011893334	0,990511	0,999787
_C200350WA	115,4793412	0,082878077	0,7372432	0,112416193	0,910493	0,999787
_C200360CA	351,1018133	0,205477646	0,6901605	0,297724446	0,765913	0,999787
_C200390CA	146,9899273	-0,412602367	0,7382005	-0,55892996	0,57621	0,999787
_C200400CA	400,5719276	0,12657087	0,7024312	0,180189699	0,857004	0,999787
_C200420WA	58,66430373	-0,808801375	0,7998937	-1,01113607	0,311951	0,999787
_C200490WA	177,7037905	-0,381862259	0,6916328	-0,55211706	0,580868	0,999787
_C200540WA	137,3956218	-0,451066463	0,7850594	-0,57456349	0,565587	0,999787
_C200550WA	16,76003099	0,523993705	1,04885	0,49958877	0,617365	0,999787
_C200570WA	215,8717555	-0,01277101	0,7002309	-0,01823828	0,985449	0,999787
_C200590WA	57,77170919	-0,352617539	0,8323158	-0,42365833	0,671815	0,999787
_C200600CA	207,2095531	0,506421228	0,7134429	0,709827253	0,477811	0,999787
_C200620CA	25,79297225	0,720941347	0,9229806	0,781101272	0,434743	0,999787
_C200630CA	40,98061161	0,132900374	0,8315117	0,159829823	0,873015	0,999787
_C200640WA	30,45468377	-0,01081746	0,878202	-0,01231774	0,990172	0,999787
_C200650WA	105,9749406	-0,350886069	0,7789456	-0,45046287	0,652377	0,999787
_C200690WA	11,95450546	0,460622566	1,1253844	0,409302432	0,682318	0,999787
_C200700WA	368,7700052	0,694425188	0,7261241	0,95634504	0,338898	0,999787
_C200730CA	101,9076671	-0,032236548	0,7678011	-0,04198555	0,96651	0,999787
_C200810CA	39,48399816	0,452304393	0,8280636	0,546219389	0,584915	0,999787
_C200820WA	49,67807757	0,649902099	0,7930878	0,819457936	0,412525	0,999787
_C200830CA	182,8832899	-0,003837859	0,7011477	-0,00547368	0,995633	0,999787
_C200840WA	149,965999	0,454352628	0,7017058	0,647497309	0,51731	0,999787
_C200870WA	36,49498586	0,256821173	0,8533507	0,300956175	0,763448	0,999787
_C200880WA	59,40220714	0,81166988	0,8230135	0,986216951	0,324027	0,999787
_C200890WA	6,000073852	-0,646596913	1,5035182	-0,43005594	0,667155	0,999787
_C200920WA	22,26496827	-0,004810697	0,9436297	-0,00509808	0,995932	0,999787
_C201060CA	7,473322851	0,508374868	1,3295406	0,38236883	0,702188	0,999787
_C201160WA	65,53000867	0,427056817	0,8804457	0,485046176	0,627644	0,999787
_C201170CA	147,6482578	-0,169151816	0,7951775	-0,21272208	0,831544	0,999787
_C201220WA	161,3376688	-0,2834323	0,7689221	-0,36860991	0,712419	0,999787
_C201230WA	6,847726301	-0,883586802	1,4117564	-0,62587766	0,531395	0,999787
_C201240CA	33,75363204	0,306459794	0,8947474	0,342509854	0,731967	0,999787
_C201250WA	22,96833016	-0,949684497	1,0513047	-0,90333899	0,366346	0,999787
_C201260WA	41,05561435	0,008874218	0,8145819	0,010894199	0,991308	0,999787
_C201310WA	45,87983281	-0,846312499	0,8010723	-1,0564746	0,290751	0,999787
_C201320WA	29,18914831	0,037188274	0,8944749	0,041575539	0,966837	0,999787
_C201340WA	129,0405371	-0,000392017	0,7099282	-0,00055219	0,999559	0,999787
_C201370CA	331,123551	-0,728273012	0,7763347	-0,93809148	0,348197	0,999787
_C201440CA	19,6283115	0,703749598	1,0891872	0,646123612	0,518199	0,999787
_C201490CA	78,24093611	-0,152108186	0,7877214	-0,19309897	0,846881	0,999787
_C201530CA	376,9859946	0,590407801	0,6710579	0,879816533	0,378959	0,999787
_C201540WA	21,75109888	-1,200824545	1,0704097	-1,12183633	0,261932	0,999787
_C201660CA	11,51892376	0,390473754	1,1286844	0,345954767	0,729377	0,999787
_C201680CA	84,93904073	-0,249523685	0,7796474	-0,32004683	0,748933	0,999787
_C201690WA	338,0313208	0,043436734	0,7639046	0,056861464	0,954656	0,999787
_C201720CA	29,41521763	0,722374288	1,0534519	0,685721189	0,492889	0,999787
_C201740CA	53,65967569	0,371544879	0,9289376	0,399967524	0,68918	0,999787

_C201760CA	7,653457117	-1,251556348	1,3363293	-0,93656281	0,348983	0,999787
_C201800WA	5,366906208	-0,258336642	1,4864047	-0,17379966	0,862023	0,999787
_C201820CA	34,05857813	0,234664663	0,8722282	0,269040454	0,787899	0,999787
_C201860CA	10,07423543	0,752684133	1,2432124	0,605434843	0,54489	0,999787
_C201870CA	25,23779833	0,718995294	0,9144147	0,78629019	0,431697	0,999787
_C201910WA	12,6081227	-0,892130873	1,0862555	-0,8212901	0,411481	0,999787
_C201930CA	30,19165932	0,104762187	0,9077319	0,115410929	0,908119	0,999787
_C202000WA	175,9946769	0,036722694	0,7787344	0,047156891	0,962388	0,999787
_C202020WA	190,6794528	-0,273851979	0,75325	-0,36356056	0,716186	0,999787
_C202040WA	65,12116653	-0,117880495	0,8055567	-0,1463342	0,883658	0,999787
_C202050CA	120,9328014	0,17565159	0,9980533	0,175994196	0,860299	0,999787
_C202080WA	8,526695699	0,421183042	1,2545703	0,335718972	0,737083	0,999787
_C202090WA	135,1077651	-0,13729916	0,7250642	-0,18936138	0,84981	0,999787
_C202170WA	100,8487669	0,879210711	0,8895589	0,988367089	0,322973	0,999787
_C202190CA	19,82968942	-0,508794192	0,9824348	-0,51789107	0,604534	0,999787
_C202200WA	88,84635188	0,044806916	0,7438738	0,060234568	0,951969	0,999787
_C202270CA	72,97248379	0,864406284	0,9058548	0,954243803	0,33996	0,999787
_C202280WA	99,99968685	0,552794609	0,7743189	0,71391074	0,475282	0,999787
_C202290CA	16,28542607	-0,366734138	1,108632	-0,33079881	0,740796	0,999787
_C202310WA	245,4450417	0,086531122	0,7007403	0,1234853	0,901723	0,999787
_C202360CA	7,727736151	-0,247158737	1,2877564	-0,19192973	0,847797	0,999787
_C202410WA	132,3496376	-0,755628024	0,7749291	-0,97509313	0,329514	0,999787
_C202420CA	87,99154077	-0,111996021	0,8673943	-0,12911778	0,897264	0,999787
_C202440WA	62,68619811	0,212365663	0,8560179	0,248085539	0,804068	0,999787
_C202490CA	96,88002128	-0,156644157	0,7351558	-0,21307612	0,831268	0,999787
_C202520WA	48,20571146	0,094009631	0,8386835	0,112091908	0,910751	0,999787
_C202540WA	693,2713783	-0,34594056	0,6672126	-0,51848625	0,604119	0,999787
_C202570WA	14,83180345	-0,249549386	1,1152964	-0,22375163	0,822951	0,999787
_C202580WA	44,78056956	0,120045996	0,8137974	0,147513363	0,882727	0,999787
_C202620WA	133,241526	-0,363694575	0,7657018	-0,47498199	0,6348	0,999787
_C202630WA	33,03555192	0,674747944	0,858243	0,786196828	0,431752	0,999787
_C202660WA	21,50898787	-0,268828112	0,9345618	-0,2876515	0,773614	0,999787
_C202670CA	9,11423279	0,468147817	1,2304317	0,380474431	0,703593	0,999787
_C202700CA	29,75231612	-0,784274856	0,9319705	-0,84152326	0,400055	0,999787
_C202720WA	359,1926402	0,299726198	0,7687149	0,389905521	0,696606	0,999787
_C202730WA	202,4754744	-0,783372014	0,7332273	-1,06838908	0,285345	0,999787
_C202770WA	477,5841024	0,233834746	0,6663886	0,350898486	0,725665	0,999787
_C202800WA	53,13553699	-0,452610645	0,7901744	-0,57279839	0,566781	0,999787
_C202840CA	41,193246	-0,351445132	0,856903	-0,41013411	0,681708	0,999787
_C202870WA	53,27442539	0,211388127	0,7791156	0,271318062	0,786146	0,999787
_C202900WA	17,39723068	0,083710013	1,0225076	0,081867376	0,934752	0,999787
_C202910WA	13,44483604	-0,951936893	1,2872723	-0,73949923	0,459604	0,999787
_C202930CA	272,5049329	0,608261923	0,680705	0,89357636	0,371549	0,999787
_C202960CA	55,89086671	0,578581223	0,7861556	0,735962712	0,461753	0,999787
_C203020CA	472,5205892	0,720358609	0,7189179	1,002004007	0,316342	0,999787
_C203130WA	73,69291441	0,654025046	0,7742223	0,844750981	0,39825	0,999787
_C203150CA	38,04964469	-1,013139161	0,9016395	-1,12366329	0,261156	0,999787
_C203210WA	45,42130237	-0,075924454	0,8154478	-0,09310768	0,925818	0,999787
_C203260WA	41,37636072	-0,881085549	0,8141994	-1,08214963	0,279186	0,999787
_C203290WA	26,47566193	-0,827362886	1,0205609	-0,81069426	0,417541	0,999787

_C203340WA	67,79758077	-0,152462377	0,7911355	-0,19271336	0,847183	0,999787
_C203360WA	320,6431869	0,391667401	0,6824939	0,573876794	0,566051	0,999787
_C203410WA	205,908165	0,103750219	0,7580605	0,136862711	0,891139	0,999787
_C203420CA	63,07680276	-0,158082891	0,7671714	-0,2060594	0,836745	0,999787
_C203450WA	57,26464519	0,196314117	0,7902319	0,24842597	0,803805	0,999787
_C203460CA	103,5316011	0,022314282	0,723126	0,030858084	0,975383	0,999787
_C203490CA	57,00575938	-0,469787446	0,7705246	-0,60969821	0,542062	0,999787
_C203500WA	150,625839	-0,269774919	0,7645897	-0,35283619	0,724211	0,999787
_C203530WA	97,14240292	0,165999955	0,7418481	0,223765416	0,82294	0,999787
_C203550CA	147,753029	0,232740047	0,7182691	0,324029043	0,745916	0,999787
_C203560CA	188,9331177	0,559130673	0,8941272	0,625336809	0,53175	0,999787
_C203570CA	17,74146462	-0,690442504	1,0177468	-0,67840299	0,497516	0,999787
_C203690CA	22,83075701	0,906000398	0,951546	0,952135183	0,341028	0,999787
_C203700WA	31,61317053	0,223142879	0,8784222	0,25402692	0,799475	0,999787
_C203800CA	134,4274044	0,024553007	0,7787519	0,031528664	0,974848	0,999787
_C203830WA	84,69410052	0,513829302	0,8626719	0,595625398	0,551426	0,999787
_C203880CA	19,15426516	0,052381652	1,0647687	0,049195335	0,960764	0,999787
_C203890WA	7,317352869	-0,96908645	1,3859952	-0,69919897	0,484428	0,999787
_C203900CA	163,9080346	-0,560681296	0,7036203	-0,79685209	0,425537	0,999787
_C203910CA	61,43686252	-0,187629972	0,7627095	-0,2460045	0,805679	0,999787
_C203930CA	87,25600865	-0,036619958	0,7357204	-0,04977429	0,960302	0,999787
_C203950WA	151,8969012	0,9273583	0,9689611	0,957064502	0,338535	0,999787
_C204080WA	654,591068	0,570187722	0,6693457	0,85185839	0,394293	0,999787
_C204100WA	20,45432797	-0,503355447	0,9582909	-0,52526371	0,5994	0,999787
_C204110WA	6,873641487	0,488634596	1,3533961	0,361043308	0,718067	0,999787
_C204130WA	133,6846366	0,002366691	0,7117406	0,003325215	0,997347	0,999787
_C204160WA	76,49162021	-0,281643655	0,769253	-0,36612619	0,714271	0,999787
_C204170CA	552,524949	-0,603407554	0,6932114	-0,87045247	0,384053	0,999787
_C204320WA	18,14195562	-1,065179577	1,027752	-1,03641693	0,300008	0,999787
_C204330CA	35,99301951	-0,291104331	0,853571	-0,34104289	0,733071	0,999787
_C204340CA	9,528813225	-0,887993843	1,2699041	-0,69926053	0,484389	0,999787
_C204360WA	107,8219536	-0,233167325	0,747367	-0,31198504	0,755052	0,999787
_C204370WA	42,07387888	0,230002198	1,0206062	0,225358423	0,8217	0,999787
_C204380CA	81,15191352	0,449483785	0,7893741	0,569417966	0,569073	0,999787
_C204390WA	7,788907141	0,230947572	1,3935351	0,16572785	0,868371	0,999787
_C204400WA	100,0493388	0,401542113	0,9638807	0,416589003	0,676979	0,999787
_C204410WA	19,33753806	-0,340503293	1,0630474	-0,32030867	0,748734	0,999787
_C204490WA	496,5600313	-0,704043831	0,8138768	-0,86504961	0,387012	0,999787
_C204500WA	390,0391707	0,047270633	0,77524	0,060975486	0,951379	0,999787
_C204520CA	16,02603441	-1,070480232	1,1681121	-0,91641911	0,359447	0,999787
_C204670WA	8,170001296	-1,148871549	1,4095645	-0,81505426	0,415041	0,999787
_C204700CA	19,814306	0,912747026	0,9691798	0,941772688	0,346309	0,999787
_C204730WA	10,67666178	-0,082765394	1,3505134	-0,06128439	0,951133	0,999787
_C204750WA	25,92690091	0,052708607	0,9093809	0,05796098	0,95378	0,999787
_C204780WA	852,986901	-0,66725032	0,7843934	-0,85065778	0,394959	0,999787
_C204790CA	111,4860304	0,251533803	1,0313068	0,243898127	0,80731	0,999787
_C204820WA	95,01545467	1,067762696	0,9401035	1,135792661	0,256043	0,999787
_C204830WA	30,61435675	0,361243238	0,9315456	0,387789118	0,698172	0,999787
_C204850CA	534,0451036	-0,76582291	0,750448	-1,02048763	0,307497	0,999787
_C204870CA	1518,478646	-0,461589779	0,7033195	-0,65630168	0,51163	0,999787

_C204960CA	45,0929157	0,209647771	0,8340066	0,25137422	0,801525	0,999787
_C204990WA	9,171213556	-0,153986748	1,2360803	-0,12457665	0,900859	0,999787
_C205020WA	70,1143203	-0,277542658	0,7634516	-0,36353668	0,716204	0,999787
_C205040CA	101,1170566	0,736488197	0,7593261	0,96992348	0,332085	0,999787
_C205060CA	11,19080171	-0,575215267	1,1543536	-0,49830074	0,618272	0,999787
_C205080CA	34,8841754	0,908507836	0,9810529	0,926053878	0,354418	0,999787
_C205160CA	784,8043945	0,031803291	0,7175091	0,044324581	0,964646	0,999787
_C205170WA	169,8683655	-0,410221672	0,7722978	-0,53117031	0,595301	0,999787
_C205220CA	156,3444883	0,316277869	0,9372828	0,337441241	0,735784	0,999787
_C205270WA	118,1373223	0,769016623	0,9831992	0,782157475	0,434122	0,999787
_C205290CA	32,32640825	-0,220771077	0,8934639	-0,24709567	0,804834	0,999787
_C205330CA	12,08005151	1,129800444	1,1813189	0,956389025	0,338876	0,999787
_C205350CA	11,2067368	-0,66932132	1,1445869	-0,58477109	0,558702	0,999787
_C205380WA	55,09786331	-0,096742688	0,7855935	-0,12314599	0,901991	0,999787
_C205390CA	8,950451644	-1,373689846	1,2611435	-1,08924152	0,276047	0,999787
_C205400WA	5,996688218	0,078849749	1,4379776	0,054833779	0,956271	0,999787
_C205410WA	1651,571606	0,540119089	0,7128609	0,757678062	0,448644	0,999787
_C205480CA	20,9240249	0,134567414	0,9619587	0,139888967	0,888748	0,999787
_C205510CA	32,90025415	0,903639226	0,8784439	1,028681779	0,303629	0,999787
_C205520WA	49,04409365	0,647376579	0,8287828	0,781117262	0,434734	0,999787
_C205540CA	97,47477602	0,184281151	0,7244994	0,254356528	0,79922	0,999787
_C205550WA	54,64370681	-0,210876878	0,7892441	-0,26718843	0,789324	0,999787
_C205560WA	32,8869774	0,044188119	0,8665805	0,050991359	0,959332	0,999787
_C205570CA	15,23414587	-0,636251016	1,0865974	-0,58554438	0,558182	0,999787
_C205580WA	134,5953075	-0,184638163	0,8572488	-0,21538458	0,829467	0,999787
_C205590CA	34,33737356	-0,09705625	1,0123487	-0,09587235	0,923622	0,999787
_C205670CA	20,76756075	0,478635552	0,958277	0,499475168	0,617445	0,999787
_C205710CA	1453,405853	0,401829134	0,670355	0,599427387	0,548888	0,999787
_C205720CA	437,1223275	0,339444498	0,7069691	0,480140527	0,631127	0,999787
_C205760CA	312,7775881	-0,328589142	0,7009239	-0,4687943	0,639217	0,999787
_C205800CA	111,0332198	0,401886231	0,7853163	0,511750797	0,608825	0,999787
_C205820WA	28,22078168	-0,482844888	0,8769205	-0,55061421	0,581898	0,999787
_C205850CA	31,30717385	-0,419684913	1,1013135	-0,38107669	0,703146	0,999787
_C205860CA	18,07015695	-0,570600377	0,9780402	-0,58341196	0,559616	0,999787
_C205910WA	106,2478443	0,039181142	0,7297168	0,053693628	0,957179	0,999787
_C205960CA	23,79568527	0,432681811	0,9738072	0,44431979	0,656811	0,999787
_C205980CA	45,69050325	0,765061476	0,8062907	0,948865614	0,342689	0,999787
_C206030WA	34,4303961	0,953233303	0,9476949	1,0058441	0,314491	0,999787
_C206040CA	10,15062473	-0,354335249	1,206221	-0,2937565	0,768944	0,999787
_C206080CA	108,0763286	0,551953935	0,7232117	0,763198242	0,445345	0,999787
_C206090WA	49,12000757	0,033053687	0,8335335	0,039654899	0,968368	0,999787
_C206110WA	81,30407026	-0,346719704	0,7373546	-0,47022114	0,638197	0,999787
_C206160WA	20,38254692	-0,894288817	0,9994219	-0,89480607	0,370891	0,999787
_C206230WA	267,8212913	0,8162707	0,7520953	1,085328834	0,277776	0,999787
_C206260WA	17,43296895	-0,78272049	1,0039217	-0,77966292	0,435589	0,999787
_C206270WA	12,88466037	-0,519599511	1,0907733	-0,47635886	0,633819	0,999787
_C206300WA	41,9421554	-0,523026741	0,8343346	-0,62687891	0,530739	0,999787
_C206320WA	168,4792125	-0,488645335	0,6970278	-0,70104134	0,483277	0,999787
_C206430CA	15,57146105	-1,109103168	1,0654658	-1,0409561	0,297896	0,999787
_C206440CA	24,31490407	-0,528662122	0,9234306	-0,57249796	0,566985	0,999787

_C206490WA	65,7222287	-0,16067768	0,7548779	-0,21285255	0,831442	0,999787
_C206520CA	27,02606826	0,224287315	0,9123029	0,245847418	0,8058	0,999787
_C206530WA	7,779003166	-0,04045011	1,298343	-0,03115518	0,975146	0,999787
_C206550WA	8,44066704	-1,287930562	1,2622597	-1,02033726	0,307569	0,999787
_C206600WA	147,6283983	-0,529179518	0,7726356	-0,68490184	0,493406	0,999787
_C206620WA	12,50220304	-0,841347059	1,0970226	-0,76693685	0,443119	0,999787
_C206650CA	21,71189292	-0,406901489	0,9329537	-0,43614329	0,662733	0,999787
_C206660WA	16,90023676	0,084521526	0,9980445	0,084687134	0,93251	0,999787
_C206710WA	5,608758363	-0,053767439	1,4462403	-0,03717739	0,970344	0,999787
_C206750CA	12,69262307	0,15814511	1,1033668	0,143329592	0,88603	0,999787
_C206760CA	9,52043754	-0,992263798	1,2009002	-0,82626663	0,408653	0,999787
_C206770WA	17,29824147	-0,86175534	1,0068494	-0,85589297	0,392057	0,999787
_C206780CA	30,42008723	-0,872129259	0,8837516	-0,98684891	0,323717	0,999787
_C206790WA	6,78583161	0,404683856	1,4947521	0,270736438	0,786594	0,999787
_C206890CA	113,0973251	0,600821687	0,7585413	0,792075122	0,428317	0,999787
_C206920CA	111,6957364	-0,167828912	0,7393258	-0,22700263	0,820422	0,999787
_C206950CA	93,66490025	0,744662509	0,7470235	0,996839517	0,318842	0,999787
_C206990WA	9,013150925	-0,941184106	1,4714829	-0,63961608	0,522422	0,999787
_C207000WA	183,9755496	0,484846295	0,7308312	0,663417643	0,507063	0,999787
_C207010WA	355,1786831	0,382539953	0,6795011	0,562971823	0,573454	0,999787
_C207020CA	28,59447461	-0,32639524	0,9819055	-0,33241002	0,73958	0,999787
_C207030CA	120,9070353	0,730579899	0,8079371	0,904253404	0,365861	0,999787
_C207060WA	87,51180407	-0,426565874	0,8866346	-0,48110673	0,630441	0,999787
_C207080CA	29,58955032	0,561178821	0,9354291	0,599915908	0,548562	0,999787
_C207100WA	234,8879661	0,131527472	0,7632565	0,172324072	0,863183	0,999787
_C207110CA	121,9133676	0,335860984	0,7188355	0,467229279	0,640336	0,999787
_C207140WA	99,96490871	-0,383370826	0,7221906	-0,53084438	0,595527	0,999787
_C207190CA	414,8122559	0,737058201	0,8243996	0,894054496	0,371293	0,999787
_C207200WA	75,69762023	0,344814522	0,7569156	0,455552169	0,648712	0,999787
_C207220WA	85,91611326	-0,374599421	0,7586106	-0,49379669	0,62145	0,999787
_C207240CA	7,656589824	0,182025657	1,3878213	0,131159289	0,895649	0,999787
_C207250CA	6,386475417	-0,589440919	1,4490811	-0,40676874	0,684178	0,999787
_C207290WA	353,577399	0,466613126	0,7096998	0,6574796	0,510873	0,999787
_C207360WA	130,685935	0,524599496	0,7744429	0,677389496	0,498159	0,999787
_C207370WA	94,73932545	-0,423136738	0,739376	-0,57228896	0,567126	0,999787
_C207390CA	181,8762585	0,08630511	0,7111333	0,121362769	0,903404	0,999787
_C207410WA	78,5548865	0,067202359	0,8065666	0,08331905	0,933598	0,999787
_C207430CA	52,98825429	-0,460831828	0,81636	-0,56449585	0,572417	0,999787
_C207440CA	74,25147989	0,508745221	0,7679817	0,662444417	0,507686	0,999787
_C207520CA	75,21391592	0,310246535	0,750861	0,413187727	0,679469	0,999787
_C207540WA	241,1039058	0,067517104	0,6802079	0,099259505	0,920932	0,999787
_C207550WA	501,4882105	0,053721428	0,6760307	0,079465958	0,936662	0,999787
_C207580WA	23,04083967	-0,411906567	0,9931118	-0,41476353	0,678315	0,999787
_C207610CA	259,00485	-0,158251215	0,677672	-0,23352186	0,815356	0,999787
_C207650CA	124,3321784	-0,086065642	0,7136638	-0,12059689	0,90401	0,999787
_C207680WA	148,7898356	0,959606307	0,8489819	1,130302494	0,258349	0,999787
_C207690WA	15,23880687	0,012669476	1,0709868	0,011829723	0,990561	0,999787
_C207720CA	35,73740697	-0,294301892	0,8416081	-0,34968995	0,726571	0,999787
_C207740WA	1673,305903	-0,583926796	0,7867709	-0,74218147	0,457977	0,999787
_C207760WA	46,02171813	-0,126738871	0,8696956	-0,14572784	0,884136	0,999787

_C207810WA	109,8614647	0,545435504	0,7179606	0,759701113	0,447433	0,999787
_C207860WA	20,37931354	0,23644795	0,9503088	0,248811696	0,803506	0,999787
_C207910CA	28,28546717	0,801544066	0,9579292	0,836746647	0,402735	0,999787
_C207920WA	41,71018267	0,482726516	0,8317535	0,580372085	0,561664	0,999787
_C207980WA	43,62023006	0,426420253	0,8149693	0,52323477	0,600811	0,999787
_C208020CA	42,49417783	0,117764508	0,837698	0,140581108	0,888201	0,999787
_C208160CA	103,7813415	0,001288872	0,7646229	0,001685631	0,998655	0,999787
_C208200WA	13,25520298	0,240636842	1,0769848	0,223435697	0,823196	0,999787
_C208240WA	134,7112966	-0,341768867	0,7059671	-0,48411441	0,628305	0,999787
_C208270CA	83,10793437	-0,257151205	0,8806379	-0,29200562	0,770282	0,999787
_C208320CA	16,64084511	-0,494868097	1,005669	-0,49207851	0,622664	0,999787
_C208330WA	13,34567002	-0,350926021	1,0852025	-0,32337378	0,746412	0,999787
_C208380CA	53,32167205	0,13949778	0,7832263	0,178106608	0,858639	0,999787
_C208390WA	30,64045566	-1,005474581	0,8915235	-1,12781609	0,259398	0,999787
_C208450WA	89,42155144	0,269502212	0,8401912	0,320762941	0,74839	0,999787
_C208460CA	7,000651394	-0,901596568	1,3721848	-0,65705186	0,511148	0,999787
_C208510WA	10,66813861	-0,303608505	1,25934	-0,24108541	0,809489	0,999787
_C208530CA	18,69983829	1,260480758	1,1770264	1,070902723	0,284213	0,999787
_C208540CA	16,95336112	-0,086929729	0,9978528	-0,08711678	0,930579	0,999787
_C208620WA	52,06884422	0,489082071	0,7847519	0,623231448	0,533132	0,999787
_C208650WA	267,8790822	-0,14901997	0,6782762	-0,21970398	0,826102	0,999787
_C208660CA	19,15089715	-0,502683426	1,0148511	-0,49532727	0,620369	0,999787
_C208740WA	38,22822727	-0,787246829	0,8746372	-0,90008382	0,368076	0,999787
_C208830WA	110,0952373	-0,282743266	0,7139602	-0,39602102	0,69209	0,999787
_C208840WA	14,75179305	0,709127504	1,2395807	0,572070475	0,567274	0,999787
_C208850CA	111,9375943	-0,186086929	0,717328	-0,25941681	0,795314	0,999787
_C208860WA	102,0821824	-0,341236875	0,7682573	-0,44417003	0,65692	0,999787
_C208900WA	82,52195272	-0,11305284	0,7386423	-0,15305492	0,878355	0,999787
_C208910CA	121,4100803	-0,437437684	0,7452548	-0,58696394	0,557228	0,999787
_C208920WA	129,3146236	-0,280723223	0,707463	-0,39680272	0,691513	0,999787
_C208930WA	78,70820032	0,05665564	0,8974563	0,063129134	0,949664	0,999787
_C208960CA	31,36142033	-0,011154299	0,8817932	-0,01264956	0,989907	0,999787
_C208990CA	58,27064943	-0,84622405	0,7787615	-1,08662795	0,277201	0,999787
_C209030WA	18,5982985	-1,285062975	1,1335796	-1,13363279	0,256949	0,999787
_C209040WA	248,1615041	-0,259732735	0,6884232	-0,37728644	0,705961	0,999787
_C209050CA	42,94045756	0,196739181	0,8151053	0,241366577	0,809271	0,999787
_C209070CA	12,60996835	-0,458573388	1,1007231	-0,41661103	0,676963	0,999787
_C209100CA	98,25978177	0,476596004	0,9558256	0,498622351	0,618045	0,999787
_C209160WA	27,69061191	0,715713058	0,9151835	0,782043191	0,434189	0,999787
_C209180WA	16,22160367	-0,37430167	1,020011	-0,36695848	0,71365	0,999787
_C209250WA	47,61136221	-0,618380791	0,8874874	-0,69677694	0,485942	0,999787
_C209290WA	27,67453627	-0,246318016	0,8878424	-0,27743438	0,781447	0,999787
_C209310CA	15,30858308	0,671626596	1,0383841	0,646799741	0,517762	0,999787
_C209360WA	55,70451977	0,998321686	0,90961	1,097527161	0,272411	0,999787
_C209420WA	54,20876576	-0,145356436	0,779498	-0,18647442	0,852073	0,999787
_C209480WA	13,80433987	0,704473038	1,2509619	0,563145064	0,573336	0,999787
_C209510CA	187,9255255	-0,126426597	0,6911643	-0,1829183	0,854862	0,999787
_C209560CA	72,44889892	0,051975851	0,7654637	0,067901128	0,945864	0,999787
_C209570CA	26,14888545	-0,102237309	0,8961521	-0,11408477	0,909171	0,999787
_C209600CA	63,76411604	0,536340808	0,8025796	0,668271201	0,50396	0,999787

_C209610WA	41,97729767	0,861209711	0,8269866	1,041382963	0,297698	0,999787
_C209620WA	109,1990677	0,453076165	0,7231949	0,626492471	0,530992	0,999787
_C209650WA	1092,228351	-0,57141481	0,7880224	-0,72512508	0,468375	0,999787
_C209670CA	103,6696566	-0,41939822	0,7236208	-0,57958288	0,562196	0,999787
_C209690CA	47,51257799	0,082704551	0,804022	0,102863543	0,918071	0,999787
_C209780CA	158,8976722	-0,142985006	0,7376239	-0,19384542	0,846297	0,999787
_C209790WA	29,58794591	0,24928699	0,9052404	0,275382092	0,783023	0,999787
_C209830CA	54,02880121	0,160329567	0,8847896	0,181206433	0,856206	0,999787
_C209860CA	8,936450028	-0,689077647	1,2464147	-0,55284781	0,580368	0,999787
_C209870WA	59,19949751	-0,128722053	0,794313	-0,16205458	0,871263	0,999787
_C209910CA	22,66852162	-0,466178287	0,9274352	-0,50265324	0,615208	0,999787
_C209920WA	9,994713414	0,954159412	1,2644465	0,754606374	0,450485	0,999787
_C209930WA	84,49105607	-0,039591473	0,7582667	-0,05221313	0,958359	0,999787
_C209960WA	15,90546079	-0,283388116	1,0130511	-0,27973722	0,779679	0,999787
_C209970CA	186,7213222	-0,322072438	0,7435164	-0,43317464	0,664888	0,999787
_C210000CA	7,025520756	-0,793733981	1,352094	-0,58704054	0,557176	0,999787
_C210010CA	99,24847289	0,43042354	0,7284665	0,590862466	0,554613	0,999787
_C210050WA	171,102392	-0,43453766	0,7296528	-0,59554028	0,551482	0,999787
_C210120WA	30,54626684	0,458610405	0,9676432	0,473945776	0,635539	0,999787
_C210160WA	11,21783409	0,334600355	1,2127408	0,27590426	0,782622	0,999787
_C210170CA	67,18888323	-0,258729945	0,7550801	-0,3426523	0,73186	0,999787
_C210200WA	30,77565852	-0,311471779	0,8691212	-0,35837555	0,720062	0,999787
_C210230WA	25,36157601	0,371403553	0,9058854	0,409989575	0,681814	0,999787
_C210330CA	6,933766653	-0,301162945	1,3405903	-0,2246495	0,822252	0,999787
_C210460CA	219,599819	0,224909613	0,6834406	0,329084364	0,742092	0,999787
_C210470CA	99,00562301	-0,331561529	0,800813	-0,41403116	0,678851	0,999787
_C210630WA	61,14585378	-0,616336669	0,9104274	-0,67697509	0,498422	0,999787
_C210670WA	984,7930573	-0,62331426	0,8060596	-0,77328561	0,439353	0,999787
_C210680WA	106,7131589	-0,691924361	0,7793747	-0,88779428	0,374651	0,999787
_C210700CA	82,62619894	-0,705244396	0,7787684	-0,90558941	0,365153	0,999787
_C210720CA	107,7191634	0,411971913	0,7471721	0,551374869	0,581377	0,999787
_C210760CA	198,8624388	0,419740409	0,7077597	0,593054934	0,553144	0,999787
_C210790CA	49,27433209	-0,81244122	0,9619089	-0,84461344	0,398327	0,999787
_C210810WA	80,02757591	0,386858761	0,7431292	0,520580759	0,602659	0,999787
_C210820CA	161,320413	0,353638622	0,7577801	0,46667712	0,640731	0,999787
_C210830WA	24,48301717	0,725336271	0,9295354	0,780321325	0,435202	0,999787
_C210850CA	210,2411414	0,336805016	0,6852594	0,491499995	0,623073	0,999787
_C210860CA	637,0971039	-0,068495158	0,6813183	-0,10053328	0,919921	0,999787
_C300030CA	352,6904102	-0,057020662	0,8092278	-0,07046305	0,943825	0,999787
_C300100WA	64,6995024	0,343902025	0,8066035	0,426358217	0,669847	0,999787
_C300130CA	189,0467951	0,482228744	0,6954856	0,693369875	0,488077	0,999787
_C300270CA	142,820612	-0,079179465	0,7278722	-0,1087821	0,913375	0,999787
_C300300WA	78,04229741	0,108026933	0,8180961	0,132046759	0,894947	0,999787
_C300360WA	21,85000012	-0,302899802	0,952128	-0,3181293	0,750387	0,999787
_C300380CA	37,27214432	0,136518339	0,8302928	0,164421927	0,869399	0,999787
_C300390WA	6,321195092	1,633920065	1,4587342	1,120094418	0,262674	0,999787
_C300450CA	488,5461905	0,501098731	0,6752037	0,742144526	0,458	0,999787
_C300470WA	110,9083497	0,052927229	0,7380943	0,071707954	0,942834	0,999787
_C300500CA	39,88602914	0,043138317	0,8588078	0,05023047	0,959939	0,999787
_C300510WA	24,0891218	0,74583518	0,9220278	0,808907449	0,418568	0,999787

_C300520WA	46,14460801	-0,188641984	0,806755	-0,2338281	0,815118	0,999787
_C300530CA	50,59029358	0,060603261	0,7954447	0,076187903	0,93927	0,999787
_C300570CA	137,2460363	-0,250460453	0,765404	-0,3272265	0,743497	0,999787
_C300610WA	18,70220148	-0,492389518	0,9840139	-0,50038877	0,616801	0,999787
_C300620CA	31,4135516	0,531396219	0,8968149	0,592537238	0,553491	0,999787
_C300630WA	117,9219262	0,314300923	0,7871842	0,399272376	0,689693	0,999787
_C300640WA	106,876345	-0,018954374	0,7170033	-0,02643555	0,97891	0,999787
_C300660WA	74,15826293	0,541010602	0,8230309	0,657339317	0,510963	0,999787
_C300730WA	141,1531187	-0,204717593	0,90766	-0,22554435	0,821556	0,999787
_C300790WA	52,26208653	0,323524252	0,7916049	0,408694075	0,682764	0,999787
_C300830CA	130,3960509	0,087131454	0,8665358	0,100551479	0,919907	0,999787
_C300890CA	62,83373867	0,207940248	0,7877272	0,263974956	0,791799	0,999787
_C300940WA	203,2572413	-0,527780311	0,7662429	-0,68878984	0,490956	0,999787
_C300950CA	28,07790112	0,553529378	1,0095801	0,548276828	0,583502	0,999787
_C300960WA	30,18272388	0,747285971	1,0558582	0,707752209	0,479099	0,999787
_C301020WA	70,14864629	0,512355106	0,7725719	0,663181105	0,507215	0,999787
_C301070CA	8,812979169	-0,447793107	1,2613852	-0,35500107	0,722589	0,999787
_C301090WA	45,14443564	-0,057321389	0,8228887	-0,06965874	0,944465	0,999787
_C301100WA	24,40429498	0,652988783	1,018771	0,640957384	0,52155	0,999787
_C301120WA	27,20185312	-0,240948834	0,8860216	-0,27194464	0,785665	0,999787
_C301150CA	29,99011745	0,647782898	0,9602231	0,674617104	0,499919	0,999787
_C301170WA	72,77003285	-0,144631955	0,9012283	-0,16048315	0,8725	0,999787
_C301190CA	78,07503284	0,049996275	0,7551021	0,066211278	0,94721	0,999787
_C301210CA	186,930034	-0,809551633	0,7742966	-1,04553171	0,295777	0,999787
_C301220WA	9,878025516	-0,629536364	1,228967	-0,51224837	0,608477	0,999787
_C301280WA	40,48321252	0,047708348	0,817339	0,05837033	0,953454	0,999787
_C301310WA	25,96713507	0,388397687	0,9183074	0,422949536	0,672332	0,999787
_C301420CA	76,56028617	-0,528407354	0,787227	-0,67122613	0,502076	0,999787
_C301510WA	127,1140486	-0,353290003	0,7162502	-0,49324943	0,621836	0,999787
_C301520CA	188,9849003	0,99096224	0,8763611	1,130769277	0,258152	0,999787
_C301570WA	21,72725772	-0,026448636	0,9319432	-0,02838009	0,977359	0,999787
_C301590WA	58,76045998	-0,323573992	0,7665436	-0,42212079	0,672937	0,999787
_C301610WA	557,941304	0,045208694	0,7203106	0,06276278	0,949955	0,999787
_C301620WA	8,540785134	-0,300352075	1,2597803	-0,23841624	0,811558	0,999787
_C301660WA	61,91850449	-0,865327535	0,7737535	-1,11835041	0,263417	0,999787
_C301670WA	94,85184668	-0,579318525	0,7261086	-0,79784008	0,424963	0,999787
_C301680CA	35,04226851	-0,221576306	0,8426903	-0,2629392	0,792597	0,999787
_C301690WA	65,44607875	0,057031878	0,7589082	0,075149902	0,940095	0,999787
_C301720CA	415,4193653	-0,124146485	0,860793	-0,14422339	0,885324	0,999787
_C301770CA	30,01988904	0,880902801	0,9912723	0,888658761	0,374187	0,999787
_C301780CA	29,64952917	-0,410277342	0,9605844	-0,42711223	0,669298	0,999787
_C301790CA	26,53310062	0,268730495	0,8941261	0,300551017	0,763757	0,999787
_C301800CA	121,6300211	-0,485098147	0,716952	-0,67661174	0,498652	0,999787
_C301820WA	24,67457893	-0,356900681	0,9139212	-0,39051582	0,696155	0,999787
_C301850WA	113,2404898	0,34521451	0,9743931	0,35428669	0,723124	0,999787
_C301900CA	49,81039605	0,224043805	0,7975203	0,28092552	0,778768	0,999787
_C301910CA	112,8586263	0,020153725	0,7544576	0,026712865	0,978689	0,999787
_C302030WA	98,51710316	0,578895546	0,7255993	0,797817165	0,424977	0,999787
_C302080WA	31,9087068	-0,45058155	0,8797408	-0,51217532	0,608528	0,999787
_C302090CA	56,53807221	-0,490469691	0,7994172	-0,61353405	0,539523	0,999787

_C302120WA	80,65803697	-0,703405895	0,7408986	-0,94939557	0,342419	0,999787
_C302140CA	54,46061564	-0,231178779	0,8266637	-0,27965274	0,779744	0,999787
_C302160CA	297,6956033	-0,180361308	0,6787826	-0,26571291	0,79046	0,999787
_C302180CA	267,9793364	0,593264551	0,6887817	0,861324545	0,389059	0,999787
_C302190CA	338,1231004	-0,40488795	0,7307979	-0,55403548	0,579555	0,999787
_C302210CA	54,81803358	-0,179672235	0,8540027	-0,21038838	0,833365	0,999787
_C302260CA	243,6906902	-0,558108722	0,70427	-0,7924641	0,42809	0,999787
_C302270WA	19,93172929	0,554420176	1,0515678	0,527231999	0,598032	0,999787
_C302290WA	23,21555993	0,212606148	0,9963641	0,213381992	0,831029	0,999787
_C302330CA	5,738824852	-1,91817026	1,8230742	-1,05216246	0,292725	0,999787
_C302340WA	7,182061024	-0,570712796	1,4402028	-0,39627252	0,691904	0,999787
_C302350WA	751,2434864	0,687495396	0,6710404	1,024521668	0,305589	0,999787
_C302390WA	18,99121248	-0,564519793	0,96903	-0,58256171	0,560188	0,999787
_C302420CA	7,964357022	-0,226009084	1,3048397	-0,17320831	0,862488	0,999787
_C302490CA	377,1051718	0,049557907	0,8672946	0,057140798	0,954433	0,999787
_C302530WA	44,4408513	-0,346442632	0,8071065	-0,42924032	0,667748	0,999787
_C302570WA	15,84470091	-0,552506383	1,0673089	-0,51766306	0,604693	0,999787
_C302580CA	10,06925707	-0,698626871	1,1705777	-0,59682228	0,550626	0,999787
_C302620CA	31,64334846	-0,097907306	0,863918	-0,11332939	0,909769	0,999787
_C302670WA	16,68123152	1,106445226	1,0555793	1,048187694	0,294552	0,999787
_C302690CA	36,2926394	0,367317489	0,9256148	0,396836212	0,691488	0,999787
_C302710WA	169,5939661	-0,092440074	0,6979773	-0,13243994	0,894636	0,999787
_C302720WA	26,83162194	-0,230307121	1,0073564	-0,22862527	0,81916	0,999787
_C302760CA	193,794143	0,666837022	0,7020488	0,949844255	0,342191	0,999787
_C302770CA	56,29188234	-0,735558553	0,8570559	-0,85823871	0,390761	0,999787
_C302820CA	65,84126117	0,13084708	0,7605027	0,172053407	0,863396	0,999787
_C302840WA	7,28120942	0,473659023	1,3248362	0,357522714	0,720701	0,999787
_C302850CA	13,25689521	0,858103023	1,1275314	0,761045798	0,44663	0,999787
_C302870CA	19,09970626	-0,108048851	1,0033129	-0,10769208	0,91424	0,999787
_C302930WA	5,382523934	-1,564007298	1,7010047	-0,91946087	0,357855	0,999787
_C302970CA	139,8533482	-0,295910925	0,7714153	-0,38359485	0,701279	0,999787
_C303060WA	16,60406101	-0,149478829	1,0019494	-0,14918801	0,881405	0,999787
_C303080WA	31,60889841	-0,166960948	0,8747026	-0,1908774	0,848622	0,999787
_C303100CA	172,8140656	-0,504417373	0,6979282	-0,72273534	0,469842	0,999787
_C303110WA	58,74931952	0,80388642	0,7750512	1,037204343	0,299641	0,999787
_C303150WA	86,03086076	0,058106726	0,7568328	0,076776175	0,938802	0,999787
_C303160CA	23,6728842	-0,348414698	0,9480326	-0,3675134	0,713236	0,999787
_C303190CA	96,13624165	0,446964288	0,7300476	0,612239956	0,540379	0,999787
_C303200CA	33,20230876	0,393831793	0,8598875	0,458003856	0,64695	0,999787
_C303210WA	17,67819505	-0,352858103	1,0029209	-0,35183044	0,724965	0,999787
_C303230CA	51,66533288	-0,405187792	0,7980044	-0,50775131	0,611628	0,999787
_C303250WA	96,76461567	-0,441941418	0,7366597	-0,59992612	0,548555	0,999787
_C303260WA	353,5477639	0,673187964	0,6710021	1,003257582	0,315737	0,999787
_C303270WA	34,67946094	-0,634120501	0,8488059	-0,74707359	0,455019	0,999787
_C303300CA	281,5097236	0,637671913	0,6896623	0,924614714	0,355166	0,999787
_C303310CA	25,11586745	0,437485817	0,9156713	0,477776061	0,63281	0,999787
_C303330CA	76,68538487	0,307013057	0,7534221	0,407491442	0,683647	0,999787
_C303340CA	132,968197	0,360325134	0,7457248	0,483187839	0,628962	0,999787
_C303370CA	22,05659647	0,032866763	1,0078019	0,032612325	0,973984	0,999787
_C303380WA	10,55119185	-0,089678798	1,2412778	-0,07224716	0,942405	0,999787

_C303410CA	603,8866112	-0,558550307	0,6911436	-0,8081538	0,419002	0,999787
_C303430CA	20,75156123	-0,370279213	0,9597448	-0,38581008	0,699637	0,999787
_C303470WA	45,61839416	0,248018157	0,8220106	0,30172135	0,762864	0,999787
_C303510CA	51,60792339	0,042898685	0,8572641	0,050041386	0,960089	0,999787
_C303530WA	12,77851117	-0,97284351	1,1561576	-0,84144543	0,400098	0,999787
_C303540WA	57,60211954	-0,052910797	0,7886493	-0,0670904	0,94651	0,999787
_C303560WA	84,82931064	0,401812437	0,7379271	0,544515094	0,586087	0,999787
_C303590WA	207,4187479	0,394653237	0,693019	0,569469555	0,569038	0,999787
_C303670WA	66,56916438	-0,379930678	0,765056	-0,49660506	0,619468	0,999787
_C303680WA	145,5084525	-0,043445698	0,715815	-0,06069403	0,951603	0,999787
_C303760WA	68,75439119	-0,331684518	0,8163471	-0,40630328	0,68452	0,999787
_C303820CA	49,08297516	0,783091587	0,8512791	0,919899912	0,357625	0,999787
_C303830WA	49,84050839	0,055823224	0,8002371	0,069758357	0,944386	0,999787
_C303840CA	88,49157233	0,353041251	0,7338955	0,481051114	0,63048	0,999787
_C303860WA	53,53184846	-0,563172571	0,9094184	-0,61926672	0,535741	0,999787
_C303930WA	194,5857527	-0,311794392	0,7550356	-0,41295323	0,679641	0,999787
_C304080WA	1078,443266	-0,52179183	0,6874089	-0,75907047	0,44781	0,999787
_C304120CA	45,22274572	0,461185374	0,8323949	0,554046401	0,579547	0,999787
_C304130WA	91,22003823	-0,903637906	0,837332	-1,07918708	0,280504	0,999787
_C304190WA	5,330045987	-0,907285601	1,4805147	-0,6128177	0,539997	0,999787
_C304230WA	44,63527999	0,28858553	0,8162648	0,353543997	0,723681	0,999787
_C304260WA	85,10344407	0,372001912	0,7612355	0,488681786	0,625067	0,999787
_C304360WA	118,4440215	-0,695328302	0,7656943	-0,90810174	0,363824	0,999787
_C304370CA	122,6687377	0,610997866	0,9288623	0,657791682	0,510672	0,999787
_C304410CA	208,8257703	0,402781138	0,6889425	0,584636836	0,558792	0,999787
_C304420WA	132,2208289	-0,426192001	0,7235128	-0,5890594	0,555821	0,999787
_C304460WA	158,169072	-0,380301785	0,7516714	-0,50594154	0,612898	0,999787
_C304540CA	42,46231935	-0,069079529	0,8245426	-0,08377921	0,933232	0,999787
_C304590WA	309,6797349	-0,262856279	0,6931151	-0,37923898	0,70451	0,999787
_C304620CA	31,85944245	0,71576644	0,8760138	0,817072118	0,413887	0,999787
_C304630WA	27,79744377	-0,960330105	0,8778992	-1,09389568	0,274001	0,999787
_C304650WA	11,18058038	-1,197982592	1,1424909	-1,04857083	0,294376	0,999787
_C304690CA	28,98425588	-0,272021953	0,8810478	-0,30874823	0,757513	0,999787
_C304700WA	75,63872595	-0,43632096	0,8068605	-0,54076381	0,58867	0,999787
_C304720CA	41,18117802	-0,149344302	0,8509131	-0,17551063	0,860678	0,999787
_C304740CA	54,83775964	-0,240783483	0,780488	-0,30850377	0,757699	0,999787
_C304750WA	30,24124579	-0,435295912	0,8753777	-0,49726642	0,619001	0,999787
_C304780CA	117,5947096	0,021534495	0,7160437	0,030074276	0,976008	0,999787
_C304790WA	49,68590159	0,120083914	0,7926999	0,151487224	0,879591	0,999787
_C304810CA	4480,960697	-0,133482203	0,6952842	-0,19198221	0,847756	0,999787
_C304840CA	35,78522984	-0,877252971	0,9925296	-0,88385569	0,376774	0,999787
_C304940WA	62,89966541	-0,010648495	0,7818792	-0,0136191	0,989134	0,999787
_C304970CA	111,4768923	-0,397838573	0,7913907	-0,50270819	0,615169	0,999787
_C305010CA	50,18753889	0,617956316	0,8292168	0,745228877	0,456133	0,999787
_C305020WA	23,94056373	0,154611905	0,9603682	0,160992326	0,872099	0,999787
_C305060WA	14,94721599	-0,144218933	1,0394849	-0,13874077	0,889655	0,999787
_C305090CA	20,22406033	0,393549344	0,9596594	0,410092721	0,681738	0,999787
_C305110WA	35,53637688	-0,844700507	1,0125764	-0,83420914	0,404163	0,999787
_C305120CA	88,28824924	0,706357542	0,7465249	0,946194172	0,34405	0,999787
_C305150WA	809,6383983	-0,232302945	0,7940131	-0,29256814	0,769852	0,999787

_C305280CA	117,6238757	0,440126783	0,7609273	0,578408467	0,562988	0,999787
_C305290CA	39,31803638	0,174192369	0,8447197	0,206213215	0,836624	0,999787
_C305320WA	105,2286129	-0,525220087	0,9926252	-0,52912224	0,596721	0,999787
_C305380WA	9,125177825	-0,220409879	1,2139669	-0,18156168	0,855927	0,999787
_C305410WA	117,7412706	-0,18229833	0,7869402	-0,23165461	0,816806	0,999787
_C305420WA	52,2096262	0,081066571	0,8020659	0,101072207	0,919493	0,999787
_C305440CA	27,77376079	0,348775606	0,8934396	0,390374009	0,69626	0,999787
_C305460WA	35,69126463	0,74677857	1,1362963	0,657204078	0,51105	0,999787
_C305540CA	5,305252751	0,622763921	1,5118843	0,411912412	0,680404	0,999787
_C305550CA	22,80518257	-0,208538341	0,9254526	-0,22533659	0,821717	0,999787
_C305680WA	35,32169173	0,431538014	0,8714632	0,495187855	0,620468	0,999787
_C305760WA	44,90990165	-0,035724271	0,8328447	-0,04289428	0,965786	0,999787
_C305790CA	11,68705548	-0,071381281	1,1435704	-0,06241967	0,950229	0,999787
_C305800WA	35,36907895	1,147775101	1,0249162	1,119872157	0,262768	0,999787
_C305850WA	12,2609856	-0,164528529	1,2187025	-0,13500303	0,892609	0,999787
_C305880CA	398,656821	-0,435717533	0,6719804	-0,64840812	0,516721	0,999787
_C305900WA	19,07571285	0,132731077	0,9712209	0,136664141	0,891296	0,999787
_C305940CA	14,90801596	0,514422715	1,0435587	0,492950452	0,622048	0,999787
_C305950WA	29,51534742	0,624920885	0,9323689	0,670250668	0,502698	0,999787
_C305970CA	295,9759751	-0,360632274	0,7121769	-0,50638018	0,61259	0,999787
_C306050CA	87,77050206	0,274380348	1,0487779	0,261619105	0,793615	0,999787
_C306110CA	117,7843203	-0,100053955	0,7151929	-0,13989786	0,888741	0,999787
_C306150WA	79,18465106	0,263477852	0,7692137	0,342528808	0,731953	0,999787
_C306240CA	37,2608643	0,566698614	0,9017781	0,628423583	0,529726	0,999787
_C306260CA	42,8109485	0,923264209	0,9087259	1,015998519	0,30963	0,999787
_C306270CA	36,08919914	-0,11734563	0,8330653	-0,14086006	0,88798	0,999787
_C306290WA	119,2443571	-0,810966516	0,7857769	-1,03205689	0,302045	0,999787
_C306350WA	10,43730761	-0,158987023	1,1803629	-0,13469334	0,892854	0,999787
_C306400CA	84,92708299	0,714540169	0,7457628	0,958133315	0,337996	0,999787
_C306410CA	32,40841539	0,66998776	0,8838617	0,758023256	0,448437	0,999787
_C306440WA	690,8726289	-0,141980506	0,6862037	-0,20690724	0,836082	0,999787
_C306540CA	6,025184448	-1,318496471	1,4421812	-0,91423774	0,360592	0,999787
_C306600CA	19,84031593	-0,011314757	0,9961327	-0,01135868	0,990937	0,999787
_C306610WA	90,04433265	0,318357427	0,8505541	0,374294167	0,708185	0,999787
_C306630WA	19,79603803	0,258207384	1,0245356	0,252023837	0,801023	0,999787
_C306640WA	16,79254758	0,340090837	1,0040511	0,338718655	0,734822	0,999787
_C306670CA	6,232109851	-0,817384576	1,4607465	-0,55956636	0,575775	0,999787
_C306680CA	34,43436964	0,124089481	0,8819293	0,140702306	0,888105	0,999787
_C306690CA	58,716434	-0,147642359	0,8902737	-0,16583929	0,868283	0,999787
_C306700CA	320,4677882	-0,206122662	0,8853502	-0,23281483	0,815905	0,999787
_C306710WA	71,74798343	-0,37865862	0,7461661	-0,50747231	0,611823	0,999787
_C306730WA	10,69300797	-0,234927883	1,2082239	-0,19444069	0,845831	0,999787
_C306740WA	68,6310431	0,401450932	0,9871994	0,406656358	0,68426	0,999787
_C306750WA	56,93918607	-0,190082864	0,8707695	-0,21829298	0,827201	0,999787
_C306800CA	27,94230461	-0,5680403	1,0596785	-0,53604968	0,591924	0,999787
_C306810WA	43,44140147	-0,003442594	0,8180003	-0,00420855	0,996642	0,999787
_C306820CA	152,7102894	-0,216924583	0,7492587	-0,28951894	0,772184	0,999787
_C306830CA	249,5861434	0,78452822	0,7631412	1,028024965	0,303938	0,999787
_C306920WA	85,58213114	-0,732241385	0,7349223	-0,99635212	0,319079	0,999787
_C306950WA	20,23147324	-0,804771524	1,0484821	-0,76755865	0,442749	0,999787

_C306970WA	53,64296533	-0,32746318	0,7893246	-0,41486503	0,678241	0,999787
_C306990WA	8,048540032	0,85392169	1,2903261	0,661787502	0,508107	0,999787
_C307030CA	30,14884528	-0,320898505	0,8713079	-0,3682952	0,712653	0,999787
_C307050WA	29,0457337	-0,38339561	0,8858929	-0,43277872	0,665176	0,999787
_C307100CA	50,27537448	-0,640664867	0,8020418	-0,79879236	0,424411	0,999787
_C307110WA	38,93332229	0,003697523	0,9667226	0,003824803	0,996948	0,999787
_C307120WA	14,18665664	-1,0272486	1,0831789	-0,94836467	0,342944	0,999787
_C307130WA	21,80885492	0,380729778	0,9864779	0,385948611	0,699535	0,999787
_C307140CA	22,69225041	0,369376064	0,9295878	0,397354666	0,691106	0,999787
_C307170CA	143,0783884	-0,106993454	0,7273	-0,14711049	0,883045	0,999787
_C307230WA	132,4935675	0,573538975	0,7797206	0,735569829	0,461993	0,999787
_C307330WA	28,72259478	0,317287079	1,0564408	0,30033589	0,763921	0,999787
_C307350WA	88,82722575	-0,384691787	0,8264762	-0,46546021	0,641602	0,999787
_C307370WA	60,88249335	-0,018624343	0,9115457	-0,02043161	0,983699	0,999787
_C307390CA	83,96867408	0,679202494	0,9046369	0,750801246	0,452772	0,999787
_C307400WA	90,16980041	0,771305407	0,7788292	0,99033967	0,322008	0,999787
_C307420WA	306,2635263	0,660994842	0,8963147	0,737458416	0,460844	0,999787
_C307430WA	124,4766823	-0,604793437	0,7831032	-0,77230362	0,439935	0,999787
_C307450CA	16,06908969	-0,419872222	1,0284727	-0,40824829	0,683091	0,999787
_C307460WA	233,1911859	-0,100271134	0,68802	-0,1457387	0,884128	0,999787
_C307470WA	41,81474608	-0,486951392	0,8098745	-0,6012677	0,547662	0,999787
_C307480WA	134,3270724	0,652077348	0,7157778	0,91100523	0,362293	0,999787
_C307490WA	132,9851392	-0,81805889	0,7388906	-1,10714488	0,268231	0,999787
_C307540CA	46,42354992	0,037369814	0,8431762	0,044320288	0,964649	0,999787
_C307570CA	20,49706012	-0,054897771	0,9760025	-0,05624757	0,955145	0,999787
_C307580WA	197,4794203	-0,485039674	0,7952183	-0,60994533	0,541898	0,999787
_C307590WA	14,1473688	-0,210815997	1,109535	-0,19000391	0,849306	0,999787
_C307630CA	193,0456354	0,099869463	0,7259308	0,137574364	0,890577	0,999787
_C307680WA	159,3661307	-0,065154448	0,7825951	-0,08325436	0,933649	0,999787
_C307690CA	85,89339305	0,323455055	0,7422614	0,435769728	0,663004	0,999787
_C307700WA	147,9532663	0,185002627	0,7064481	0,261877158	0,793416	0,999787
_C307760CA	17,24158399	-0,577168434	1,1506378	-0,50160738	0,615944	0,999787
_C307770CA	79,75636443	-0,011343162	0,7828953	-0,01448874	0,98844	0,999787
_C307880CA	41,83158066	-0,941341348	0,9340951	-1,00775749	0,313571	0,999787
_C307900CA	77,03812447	-0,644468296	0,802629	-0,80294666	0,422006	0,999787
_C307940WA	194,035248	-0,965637599	0,878756	-1,09886884	0,271825	0,999787
_C400020WA	622,6599418	-0,552978819	0,7180397	-0,77012291	0,441227	0,999787
_C400060WA	79,49359307	-0,170184618	0,8712483	-0,19533424	0,845131	0,999787
_C400070CA	584,3705573	-0,138349105	0,7919166	-0,17470161	0,861314	0,999787
_C400100CA	86,46790992	0,265810865	0,748235	0,355250522	0,722402	0,999787
_C400190WA	6,067275952	-0,504430318	1,4312941	-0,35242953	0,724516	0,999787
_C400230WA	123,3421818	-0,493364096	0,7814422	-0,63135074	0,527811	0,999787
_C400260WA	231,2355068	-0,812445099	0,7662813	-1,06024387	0,289034	0,999787
_C400290CA	9,321887821	-1,284191985	1,4721554	-0,87232094	0,383033	0,999787
_C400330CA	556,7643699	-0,700967831	0,7291963	-0,96128827	0,336407	0,999787
_C400380WA	113,0717459	0,243229013	0,7227492	0,33653306	0,736469	0,999787
_C400410WA	69,46583367	0,438707048	0,7586489	0,578274122	0,563079	0,999787
_C400420CA	403,1505999	-0,740986795	0,6851603	-1,0814795	0,279484	0,999787
_C400460CA	201,2888475	0,583263763	0,7130228	0,818015569	0,413348	0,999787
_C400470CA	261,5554387	-0,566777506	0,7780358	-0,72847231	0,466325	0,999787

_C400490WA	30,20654758	0,503187076	0,9668982	0,520413686	0,602775	0,999787
_C400500WA	54,55469886	-0,703393342	0,7966558	-0,88293253	0,377273	0,999787
_C400560CA	42,08099105	-0,38020569	0,8553263	-0,4445154	0,65667	0,999787
_C400610WA	38,8804744	-0,891343351	0,9038432	-0,98617031	0,32405	0,999787
_C400690CA	30,37202212	0,596854568	1,0366638	0,575745537	0,564787	0,999787
_C400700CA	63,69123759	-0,516288763	0,7701872	-0,6703419	0,50264	0,999787
_C400710WA	116,5573666	-0,211349468	0,711272	-0,29714295	0,766357	0,999787
_C400730CA	9,21177677	0,229662711	1,2723774	0,180498889	0,856761	0,999787
_C400740WA	33,4739442	0,336697212	0,852449	0,39497638	0,69286	0,999787
_C400750CA	66,74033998	-0,00108338	0,7725363	-0,00140237	0,998881	0,999787
_C400770CA	84,86527945	0,077895702	0,7896795	0,098642175	0,921422	0,999787
_C400780CA	92,89259444	-0,100814717	0,7903396	-0,12755873	0,898498	0,999787
_C400820WA	82,66140548	0,277646221	0,7995254	0,347263806	0,728393	0,999787
_C400840WA	140,542138	-0,314134668	0,7293942	-0,43067885	0,666702	0,999787
_C400860CA	38,15618045	0,2445834	0,92698	0,263849694	0,791896	0,999787
_C400880WA	70,28569481	0,896418579	0,9587586	0,934978378	0,349799	0,999787
_C400920CA	6,596057989	-1,470611084	1,5688638	-0,93737331	0,348567	0,999787
_C401020CA	186,3062791	-0,518895646	0,7453218	-0,69620348	0,486301	0,999787
_C401090CA	8,981915473	0,430097976	1,2255985	0,350928929	0,725642	0,999787
_C401120CA	21,11832472	-0,341905182	0,9616297	-0,35554765	0,722179	0,999787
_C401130CA	11,8429551	-0,34881924	1,225378	-0,28466256	0,775903	0,999787
_C401140CA	52,07079631	-0,114877046	0,7882858	-0,1457302	0,884134	0,999787
_C401230CA	24,27378227	-0,732833752	0,921045	-0,79565465	0,426233	0,999787
_C401240CA	31,43869151	-0,379589458	0,927884	-0,40909151	0,682473	0,999787
_C401290WA	272,5129538	-0,811998504	0,8517526	-0,95332675	0,340425	0,999787
_C401420WA	69,70026798	0,605736504	0,8744378	0,692715384	0,488488	0,999787
_C401460CA	31,63087514	0,078633575	0,8663992	0,090759053	0,927684	0,999787
_C401470WA	16,18972181	0,330575229	1,0796612	0,306184215	0,759464	0,999787
_C401510WA	79,67499777	-0,296505469	0,7374603	-0,40206293	0,687638	0,999787
_C401560CA	17,55199666	-0,569120656	1,0220555	-0,5568393	0,577637	0,999787
_C401580CA	20,97152448	-0,762375131	1,013943	-0,75189151	0,452116	0,999787
_C401650CA	64,0772035	0,171026845	0,7908708	0,216251308	0,828792	0,999787
_C401660WA	88,55445041	-0,291435942	0,8461963	-0,34440703	0,73054	0,999787
_C401670CA	38,32751507	0,737710102	0,8567058	0,861100852	0,389183	0,999787
_C401710CA	392,5020927	0,392214987	0,719865	0,544845216	0,58586	0,999787
_C401720CA	1015,4126	0,260422332	0,7076709	0,367999234	0,712874	0,999787
_C401730CA	105,9975402	-0,040500727	0,7295645	-0,05551356	0,955729	0,999787
_C401780CA	87,52997237	-0,117587377	0,7671837	-0,15327147	0,878184	0,999787
_C401790WA	37,43085336	0,310570565	0,9126134	0,340309013	0,733624	0,999787
_C401820CA	207,5405653	0,756021577	0,6882834	1,098416116	0,272023	0,999787
_C401830CA	58,74702288	-0,316787682	0,7828403	-0,40466452	0,685724	0,999787
_C401840CA	162,2044081	0,122884604	0,7061937	0,174009771	0,861858	0,999787
_C401910WA	74,40113865	-0,372413578	0,7723709	-0,48216936	0,629686	0,999787
_C401930CA	77,49388422	-0,147479076	0,8186698	-0,18014475	0,857039	0,999787
_C401950WA	112,1399301	-0,298885645	0,7200434	-0,41509396	0,678073	0,999787
_C401960CA	79,43942981	-0,561388187	0,7885965	-0,7118827	0,476537	0,999787
_C401970WA	14,21553467	0,126299526	1,1144759	0,113326387	0,909772	0,999787
_C401990WA	18,85285236	0,07668647	0,9920472	0,077301233	0,938384	0,999787
_C402040WA	40,50753614	-0,7631934	0,8629617	-0,88438848	0,376487	0,999787
_C402080WA	17,87562758	-0,345871099	0,9956543	-0,3473807	0,728305	0,999787

_C402090CA	57,84090703	0,770359719	0,8271285	0,931366415	0,351664	0,999787
_C402100CA	14,54697792	-0,699513674	1,0381018	-0,67383918	0,500414	0,999787
_C402160CA	67,45017805	0,635373254	0,7650166	0,830535257	0,406236	0,999787
_C402170CA	26,18196655	-0,349267287	0,9037637	-0,38645864	0,699157	0,999787
_C402200CA	11,15361244	-0,396504907	1,1698109	-0,33894788	0,734649	0,999787
_C402330CA	12,81720535	0,541811023	1,1107089	0,487806505	0,625687	0,999787
_C402400CA	31,35868803	-0,706362129	0,8805974	-0,80213974	0,422472	0,999787
_C402420CA	25,1385738	0,05312188	0,9639151	0,055110539	0,95605	0,999787
_C402430WA	148,1645738	-0,385556384	0,7100752	-0,54297963	0,587144	0,999787
_C402450WA	16,59938832	-0,172189212	1,0584701	-0,16267745	0,870772	0,999787
_C402470CA	156,9236732	0,156808721	0,7667514	0,2045105	0,837955	0,999787
_C402510WA	27,79019004	-0,3288052	0,8820625	-0,3727686	0,709321	0,999787
_C402590CA	67,26668153	0,588248508	0,7606233	0,773376883	0,439299	0,999787
_C402600CA	206,9080592	-0,909918934	0,8423174	-1,08025661	0,280028	0,999787
_C402670WA	14,91768563	-0,898800325	1,0392982	-0,8648147	0,387141	0,999787
_C402700WA	17,83980726	0,213945844	1,1234524	0,19043606	0,848967	0,999787
_C402720CA	58,0277951	0,6605225	0,7837018	0,842823732	0,399327	0,999787
_C402740WA	102,1766884	-0,706969379	0,7977948	-0,88615443	0,375534	0,999787
_C402780WA	303,6752825	0,42930423	0,8409059	0,51052586	0,609683	0,999787
_C402850WA	223,5641553	0,695698395	0,7778387	0,894399276	0,371108	0,999787
_C402880CA	48,7522673	0,584928211	0,9008068	0,649338168	0,51612	0,999787
_C402920WA	176,9116506	-0,926788649	0,882622	-1,05004022	0,2937	0,999787
_C402950CA	22,12380449	-0,143987519	0,9498368	-0,15159185	0,879509	0,999787
_C402960WA	166,0678055	0,561888777	0,7440797	0,755145961	0,450161	0,999787
_C403020WA	141,8672574	0,095991863	0,7351033	0,130582821	0,896105	0,999787
_C403040WA	112,6866957	0,476285877	0,748316	0,636476902	0,524466	0,999787
_C403050CA	108,2922374	-0,190636793	0,7469977	-0,255204	0,798566	0,999787
_C403080WA	127,5084146	0,311074681	0,9032764	0,344384834	0,730557	0,999787
_C403090WA	95,36653828	-0,365519899	0,7421334	-0,49252586	0,622348	0,999787
_C403110WA	351,9982236	0,205671183	0,672381	0,305884864	0,759692	0,999787
_C403130WA	111,3261096	0,351262553	0,7158835	0,490669971	0,62366	0,999787
_C403140CA	34,43870735	0,424532709	0,8427897	0,50372321	0,614456	0,999787
_C403150WA	124,2648395	0,570249839	0,710346	0,802777584	0,422103	0,999787
_C403160CA	22,43706183	-1,195483404	1,1247823	-1,0628576	0,287847	0,999787
_C403190WA	146,9202705	0,281374694	0,7050092	0,399107859	0,689814	0,999787
_C403200CA	39,13753778	-0,481497859	0,8445946	-0,57009344	0,568614	0,999787
_C403230CA	222,2663926	0,393476016	0,6922467	0,568404356	0,56976	0,999787
_C403290WA	148,7998375	-0,143391288	0,7822865	-0,18329767	0,854564	0,999787
_C403300CA	8,48283467	0,317583053	1,2570979	0,252631919	0,800553	0,999787
_C403320WA	46,23023725	-0,071604089	0,8609673	-0,08316702	0,933719	0,999787
_C403350CA	64,30306207	-0,182961723	0,7641512	-0,23943132	0,810771	0,999787
_C403370CA	11,82123014	-0,419675933	1,1185822	-0,3751856	0,707522	0,999787
_C403400WA	145,6054661	0,435914962	0,7005527	0,622244337	0,533781	0,999787
_C403410WA	143,7887919	0,034680355	0,8221458	0,042182732	0,966353	0,999787
_C403530WA	31,2766026	-0,625348956	0,9266611	-0,67484104	0,499777	0,999787
_C403560WA	139,8720446	0,252716562	0,7061198	0,357894763	0,720422	0,999787
_C403580WA	13,68426633	-0,123731633	1,0691045	-0,1157339	0,907863	0,999787
_C403680CA	25,9929799	0,218842584	1,0270857	0,213071394	0,831271	0,999787
_C403690CA	22,89838884	-0,763825725	0,9621097	-0,79390709	0,42725	0,999787
_C403700WA	193,063108	0,286106556	0,6913797	0,413819699	0,679006	0,999787

_C403730CA	30,10263274	0,409639208	0,8927646	0,458843477	0,646347	0,999787
_C403750CA	41,08369254	0,478021067	0,8374459	0,570808286	0,56813	0,999787
_C403780CA	70,01086106	0,81306201	0,9649223	0,842619185	0,399441	0,999787
_C403810WA	31,93327049	-0,923053215	0,8528637	-1,08229869	0,27912	0,999787
_C403830WA	77,64795453	0,835856713	0,888247	0,941018329	0,346695	0,999787
_C403850WA	89,30187256	0,055746862	0,8167892	0,068251227	0,945586	0,999787
_C403860CA	15,90328609	-0,288328656	1,0228681	-0,28188254	0,778034	0,999787
_C403880WA	18,1440425	-0,979450945	1,0123815	-0,96747214	0,333308	0,999787
_C403920WA	25,38598153	-0,694171116	0,9080082	-0,76449872	0,44457	0,999787
_C403930CA	51,31505138	-0,212437648	0,7846731	-0,27073394	0,786596	0,999787
_C403960WA	12,21986379	-0,529169821	1,1011278	-0,48057077	0,630822	0,999787
_C403990CA	11,85004372	-0,650951832	1,1743377	-0,55431402	0,579364	0,999787
_C404010WA	76,26090522	0,670176644	0,7519093	0,891299843	0,372768	0,999787
_C404090CA	35,49778226	0,485445797	0,8663201	0,560353828	0,575238	0,999787
_C404110WA	9,407199709	-1,201662846	1,2267852	-0,97952181	0,327322	0,999787
_C404120CA	39,7117795	-0,265471126	0,8218182	-0,32302903	0,746673	0,999787
_C404130WA	55,42832284	0,637003594	0,8809109	0,723119176	0,469607	0,999787
_C404140WA	5,825499923	0,823993912	1,4599105	0,564414	0,572472	0,999787
_C404160WA	99,31192126	-0,232309155	0,8437025	-0,27534488	0,783051	0,999787
_C404180CA	32,31241256	-0,22304028	0,8823705	-0,25277396	0,800443	0,999787
_C404230WA	14,46600374	0,000530186	1,0752272	0,000493093	0,999607	0,999787
_C404240CA	190,4102547	0,20536545	0,7506105	0,273597896	0,784394	0,999787
_C404250WA	22,2050199	-0,840064824	0,9735388	-0,86289815	0,388193	0,999787
_C404270WA	15,64646855	-0,359477217	1,2407796	-0,28971882	0,772031	0,999787
_C404280CA	26,80958555	-0,681947552	0,9469229	-0,72017223	0,471419	0,999787
_C404300CA	9,61910347	0,651436902	1,2525562	0,520085973	0,603004	0,999787
_C404330CA	332,384771	0,044006226	0,6977707	0,063066886	0,949713	0,999787
_C404350WA	76,72152139	0,508052749	0,7460488	0,680991321	0,495877	0,999787
_C404360WA	10,04559864	-1,305945651	1,2536345	-1,04172755	0,297538	0,999787
_C404380CA	6,210943482	-0,825943043	1,482126	-0,5572691	0,577344	0,999787
_C404390WA	1231,701416	0,561542111	0,7081149	0,79300989	0,427772	0,999787
_C404460CA	164,9883957	0,098305805	0,6962143	0,141200493	0,887712	0,999787
_C404490CA	8,904174679	-0,928711159	1,2732908	-0,72937867	0,46577	0,999787
_C404500CA	116,032468	0,620768885	0,7373803	0,841857122	0,399868	0,999787
_C404510WA	192,9672995	0,338095079	0,6990758	0,483631506	0,628647	0,999787
_C404550CA	73,77570144	-0,641889795	0,8154004	-0,78720811	0,43116	0,999787
_C404560CA	94,65703767	0,491106721	0,7453657	0,658880217	0,509973	0,999787
_C404600CA	72,81158471	-0,68836214	0,7878875	-0,87368079	0,382292	0,999787
_C404650WA	48,02100301	0,554786773	0,8104711	0,684523798	0,493644	0,999787
_C404660CA	130,7927219	-0,375403477	0,7106132	-0,52828106	0,597304	0,999787
_C404670CA	42,6798609	-0,062838764	0,906283	-0,0693368	0,944722	0,999787
_C404750WA	101,3741773	-0,438070324	0,7201735	-0,60828439	0,542999	0,999787
_C404760CA	26,5510453	0,717374534	0,937345	0,765326021	0,444077	0,999787
_C404800WA	376,5469342	0,102937915	0,6709001	0,153432563	0,878057	0,999787
_C404820CA	150,1513398	0,683356203	0,8088922	0,844804994	0,39822	0,999787
_C404860WA	32,74500787	0,208367246	0,9451426	0,220461172	0,825512	0,999787
_C404870CA	589,9187458	-0,259686337	0,6796462	-0,38209045	0,702394	0,999787
_C404920WA	134,8231445	0,18120589	0,7038262	0,257458279	0,796825	0,999787
_C404930CA	26,25285995	0,084217078	0,8936091	0,094243759	0,924916	0,999787
_C404970CA	228,0145801	-0,516084922	0,6929478	-0,74476741	0,456412	0,999787

_C404980WA	25,62773894	0,421696603	0,9000476	0,468526979	0,639408	0,999787
_C405000WA	8,648474322	-0,845264189	1,2710248	-0,66502573	0,506034	0,999787
_C405080CA	24,9708425	-0,236774377	0,9103851	-0,26008156	0,794801	0,999787
_C405160CA	33,09584913	-0,693730182	0,8571765	-0,80932007	0,418331	0,999787
_C405180CA	196,6937109	-0,050241811	0,884887	-0,05677766	0,954722	0,999787
_C405210WA	37,15166444	0,279761162	0,8452108	0,330995724	0,740648	0,999787
_C405220CA	14,53071378	-0,267703916	1,0498573	-0,25499075	0,79873	0,999787
_C405230CA	74,84229125	0,762411477	0,8361354	0,911827757	0,361859	0,999787
_C405260WA	94,26444089	0,442953551	0,7377391	0,600420341	0,548226	0,999787
_C405310WA	7,999688952	0,091214483	1,3288788	0,068640184	0,945276	0,999787
_C405330CA	137,8614423	0,691572236	0,7663984	0,902366464	0,366862	0,999787
_C405340WA	44,41744579	-0,819014529	0,8395102	-0,97558616	0,32927	0,999787
_C405350WA	29,41040438	-0,223389754	0,9103176	-0,2453976	0,806149	0,999787
_C405360CA	5,543401913	0,718973508	1,488294	0,483085665	0,629035	0,999787
_C405390WA	86,19660685	-0,484317201	0,749169	-0,64647256	0,517973	0,999787
_C405440CA	37,00641112	-0,941461917	0,8409802	-1,11948171	0,262935	0,999787
_C405590WA	226,8018286	0,330537194	0,6821846	0,484527522	0,628012	0,999787
_C405610CA	413,770665	-0,754661471	0,6826656	-1,10546287	0,268959	0,999787
_C405620CA	36,45790202	-0,135970569	0,8941925	-0,15205962	0,87914	0,999787
_C405630WA	274,2645213	-0,085162363	0,6899433	-0,12343385	0,901764	0,999787
_C405640CA	88,90170861	0,502572705	0,7539439	0,666591615	0,505033	0,999787
_C405650WA	359,2748547	0,440088752	0,7595806	0,579383854	0,56233	0,999787
_C405730WA	8,589236801	-0,705873711	1,2798505	-0,55152823	0,581272	0,999787
_C405740CA	219,7084539	-0,473123918	0,7082966	-0,66797433	0,50415	0,999787
_C405790WA	89,65423978	0,195182957	0,8105526	0,240802334	0,809708	0,999787
_C405800CA	20,6724444	-0,097561253	0,9617925	-0,1014369	0,919204	0,999787
_C405810WA	382,1572307	0,363028294	0,6747347	0,53803115	0,590556	0,999787
_C405820WA	28,47260224	0,268028985	0,8926973	0,300246217	0,763989	0,999787
_C405850CA	106,4247274	0,320122432	0,8415389	0,380401222	0,703648	0,999787
_C405860WA	77,62127936	0,550990284	0,7664747	0,718862969	0,472225	0,999787
_C405890WA	8,232935883	-0,538079687	1,3285935	-0,40499949	0,685478	0,999787
_C405970WA	125,6789072	-0,260160973	0,7484347	-0,34760676	0,728136	0,999787
_C405980CA	45,91313536	0,189030624	0,8099927	0,233373236	0,815472	0,999787
_C406020CA	68,5851199	-0,272492385	0,7582561	-0,35936722	0,71932	0,999787
_C406040WA	17,87014337	0,391486351	0,9938376	0,393913815	0,693645	0,999787
_C406080WA	8,743690185	-0,662346077	1,269537	-0,52172253	0,601864	0,999787
_C406090CA	80,61825164	0,44225047	0,7870268	0,561925554	0,574167	0,999787
_C406130WA	506,5645259	0,106551449	0,6897179	0,154485543	0,877227	0,999787
_C406160WA	15,30545038	0,026224957	1,0283207	0,025502704	0,979654	0,999787
_C406170CA	144,5334003	0,734347222	0,7023098	1,045617265	0,295738	0,999787
_C406230CA	128,5114352	0,357395201	0,7399362	0,483008162	0,62909	0,999787
_C406290WA	28,23832711	-0,37830288	0,9454687	-0,40012206	0,689067	0,999787
_C406370CA	42,50691594	-0,649138592	0,9513352	-0,68234477	0,495021	0,999787
_C406410WA	36,27439382	0,05833982	0,8844807	0,065959406	0,94741	0,999787
_C406430CA	58,1791298	-0,453193934	0,85626	-0,52927143	0,596617	0,999787
_C406440CA	19,20586716	-0,366506846	0,9842843	-0,37235874	0,709626	0,999787
_C406450WA	24,54315519	-0,781775358	0,9193539	-0,85035304	0,395129	0,999787
_C406460CA	51,60626639	-0,773724987	0,8477601	-0,91266977	0,361416	0,999787
_C406590WA	35,72669724	-0,085982826	0,8753651	-0,0982251	0,921754	0,999787
_C406650WA	192,0087793	0,032561921	0,6918441	0,047065401	0,962461	0,999787

_C406660WA	6,451831867	-1,42726814	1,4572565	-0,97942134	0,327372	0,999787
_C406670WA	14,39695599	-0,401115892	1,0475272	-0,38291692	0,701781	0,999787
_C406680CA	328,83215	0,476777305	0,8360079	0,570302378	0,568473	0,999787
_C406690CA	294,8464066	-0,15036787	0,7729379	-0,1945407	0,845753	0,999787
_C406730CA	134,3297621	-0,660847822	0,724875	-0,91167146	0,361942	0,999787
_C406740CA	126,9851625	0,065794869	0,7155822	0,091945927	0,926741	0,999787
_C406770WA	76,28778054	-0,642500472	0,7774661	-0,82640316	0,408575	0,999787
_C406800WA	267,4272833	0,690430785	0,7452064	0,926496057	0,354188	0,999787
_C406810CA	242,2323962	0,110102201	0,6818061	0,161486085	0,871711	0,999787
_C406840WA	14,10028608	0,577115316	1,0818564	0,533449079	0,593723	0,999787
_C406880CA	71,6681429	-0,126720595	0,7522417	-0,16845728	0,866224	0,999787
_C406910WA	8,932252875	1,378447632	1,2661126	1,088724344	0,276275	0,999787
_C406940CA	39,40280137	-0,095049467	0,8242355	-0,11531833	0,908193	0,999787
_C406950WA	14,92428009	0,092045795	1,0347654	0,088953301	0,929119	0,999787
_C406960WA	17,63657909	0,458573838	1,1006147	0,416652477	0,676933	0,999787
_C406990WA	24,75193217	-0,686812717	1,0257989	-0,66953936	0,503151	0,999787
_C407010CA	21,65748743	-0,764655195	1,0578781	-0,72281973	0,469791	0,999787
_C407080CA	99,69864611	-0,247905173	0,7867402	-0,31510424	0,752683	0,999787
_C407100CA	254,618997	0,601736411	0,7736077	0,777831496	0,436668	0,999787
_C407160WA	11,07578267	0,564655476	1,1717721	0,481881666	0,62989	0,999787
_C407170CA	9,916490154	0,741970769	1,2685481	0,584897623	0,558617	0,999787
_C407220CA	171,1442838	0,397274656	0,6946044	0,571943806	0,56736	0,999787
_C500030WA	540,9138611	0,67606529	0,6643521	1,017631015	0,308853	0,999787
_C500040CA	446,7923425	-0,06305851	0,6953886	-0,09068096	0,927746	0,999787
_C500050WA	63,95045838	-0,083274823	0,8012097	-0,10393636	0,91722	0,999787
_C500060CA	137,3011848	0,101141056	0,7745719	0,130576722	0,89611	0,999787
_C500080CA	314,7267291	-0,241310583	0,6985713	-0,34543444	0,729768	0,999787
_C500120WA	20,036773	0,272705702	0,9573351	0,284859188	0,775752	0,999787
_C500130CA	178,396367	0,470177341	0,7025103	0,669281784	0,503316	0,999787
_C500140CA	36,19964517	-0,855514801	1,0225831	-0,83662129	0,402805	0,999787
_C500150CA	661,6868965	0,603496964	0,7335975	0,822654088	0,410705	0,999787
_C500160WA	94,41800203	0,293408029	0,7783185	0,376976824	0,706191	0,999787
_C500170WA	235,5358548	-0,772814961	0,77974	-0,99111873	0,321628	0,999787
_C500230CA	11,93841812	-1,06755499	1,235733	-0,86390426	0,38764	0,999787
_C500250CA	19,82124238	-0,187635883	0,9980622	-0,1880002	0,850876	0,999787
_C500280CA	99,65691677	0,494177417	0,7494398	0,659395791	0,509642	0,999787
_C500310CA	397,6199129	0,798899739	0,7061227	1,131389425	0,257891	0,999787
_C500330CA	96,16819488	0,2547244	0,7265793	0,350580293	0,725903	0,999787
_C500340WA	89,10356884	0,149966459	0,7453132	0,201212677	0,840532	0,999787
_C500350CA	126,431551	0,59111442	0,7354048	0,80379461	0,421516	0,999787
_C500380WA	37,51069866	0,255272089	0,8288492	0,307983741	0,758095	0,999787
_C500400CA	114,4224956	0,032941688	0,9350509	0,035229835	0,971896	0,999787
_C500510WA	17,70958852	-0,276929003	1,0859618	-0,25500804	0,798717	0,999787
_C500550CA	68,75428935	0,468778729	0,7822752	0,599250416	0,549006	0,999787
_C500560WA	132,6979318	-0,321201311	0,7330745	-0,43815645	0,661273	0,999787
_C500570WA	195,0151084	0,37271136	0,8325346	0,447682748	0,654382	0,999787
_C500580WA	45,05621942	0,318659007	0,8725934	0,365186119	0,714972	0,999787
_C500610CA	29,73450607	-0,044965223	0,90209	-0,04984561	0,960245	0,999787
_C500750CA	146,9234077	-0,763529739	0,7078608	-1,07864391	0,280746	0,999787
_C500760WA	41,74231269	-0,534743717	0,8090442	-0,66095732	0,50864	0,999787

_C500780CA	37,33903482	-0,099926559	0,8502529	-0,11752569	0,906443	0,999787
_C500790CA	436,43377	0,050133601	0,6826643	0,073438144	0,941457	0,999787
_C500800CA	360,9521905	-0,736821484	0,7155052	-1,02979193	0,303108	0,999787
_C500820WA	168,616898	0,571750291	0,9295278	0,615097558	0,53849	0,999787
_C500840WA	30,56350184	-0,431893869	0,8637527	-0,50002028	0,617061	0,999787
_C500850CA	12,64504735	0,824252028	1,2762036	0,645862501	0,518368	0,999787
_C500920WA	12,32713495	1,29722944	1,268012	1,023041898	0,306288	0,999787
_C500950CA	120,112485	0,063089513	0,7134041	0,088434466	0,929531	0,999787
_C501000CA	118,8424292	0,220220229	0,722287	0,304892971	0,760448	0,999787
_C501030WA	6,190658992	-0,712233878	1,4642225	-0,48642463	0,626666	0,999787
_C501040WA	6,472998236	-1,413043408	1,4577899	-0,96930523	0,332393	0,999787
_C501070CA	126,8250178	0,727229177	0,75565	0,96238895	0,335854	0,999787
_C501090CA	150,4966278	0,308802271	0,7049817	0,43802877	0,661365	0,999787
_C501140CA	227,4340292	0,13058914	1,0025343	0,130259026	0,896361	0,999787
_C501170WA	10,9903126	-0,677571215	1,1465662	-0,59095692	0,554549	0,999787
_C501190WA	96,21577768	-0,129706023	0,7545261	-0,17190395	0,863513	0,999787
_C501220WA	38,36373464	0,698339072	0,9502282	0,734917242	0,46239	0,999787
_C501230CA	212,0449036	-0,620205083	0,7231024	-0,8577002	0,391058	0,999787
_C501290CA	178,9661046	-0,368867966	0,7786646	-0,47371867	0,635701	0,999787
_C501300CA	11,21196808	-0,568677331	1,1490744	-0,49490034	0,62067	0,999787
_C501310WA	32,28022503	-0,323973193	0,916582	-0,35345793	0,723745	0,999787
_C501350WA	40,64972696	0,452232814	0,8400415	0,538345803	0,590338	0,999787
_C501410CA	74,74171624	-0,035653975	0,8686728	-0,0410442	0,967261	0,999787
_C501440CA	39,21931078	-0,323616188	0,8753332	-0,36970627	0,711601	0,999787
_C501470CA	10,42314205	-0,429696063	1,2034475	-0,35705426	0,721051	0,999787
_C501490CA	67,35909474	0,103923241	0,8824491	0,117766844	0,906252	0,999787
_C501530CA	53,86259243	0,124025467	0,8567613	0,144760819	0,8849	0,999787
_C501540WA	25,58412491	0,058636486	1,091385	0,053726671	0,957153	0,999787
_C501550CA	19,16256472	-0,358768668	0,9651694	-0,37171573	0,710105	0,999787
_C501560CA	34,6002329	0,143111589	0,8712861	0,164253271	0,869532	0,999787
_C501590WA	29,20807554	-0,62696269	0,8840221	-0,7092161	0,47819	0,999787
_C501710CA	16,93194183	0,719123361	1,0283906	0,69927067	0,484383	0,999787
_C501730WA	5,266535187	0,848452241	1,6324423	0,519744087	0,603242	0,999787
_C501870WA	357,7276858	0,226235001	0,8204077	0,275759252	0,782733	0,999787
_C501880CA	37,26242307	-0,7010124	0,9181965	-0,76346668	0,445185	0,999787
_C501890WA	86,73368168	-0,387555089	0,7549747	-0,51333521	0,607717	0,999787
_C501900CA	8,397840142	0,947777918	1,2725132	0,744807927	0,456388	0,999787
_C501930WA	42,54857755	0,385313901	0,8524118	0,452027868	0,651249	0,999787
_C501940WA	153,4819734	0,002639734	0,7024278	0,003758015	0,997002	0,999787
_C501960CA	629,0220335	0,515977989	0,6715885	0,768294806	0,442312	0,999787
_C501980CA	71,34564102	-0,314622617	0,7661128	-0,410674	0,681312	0,999787
_C502040WA	190,6094676	0,206241938	0,6888683	0,29939242	0,764641	0,999787
_C502050WA	195,1044289	0,058041759	0,7422556	0,078196459	0,937672	0,999787
_C502060WA	23,8738324	0,191561279	0,9448304	0,202746732	0,839333	0,999787
_C502140CA	51,31546248	-0,248276108	0,8078448	-0,30733143	0,758591	0,999787
_C502190CA	246,6730097	-0,628678871	0,7135154	-0,88110068	0,378263	0,999787
_C502200WA	5,709117691	0,565061908	1,4649307	0,385726031	0,6997	0,999787
_C502210WA	166,875999	0,266503244	0,7112018	0,374722378	0,707867	0,999787
_C502230WA	465,046688	-0,11727989	0,6952931	-0,16867692	0,866051	0,999787
_C502280CA	39,88334266	0,881907917	0,8508272	1,036529953	0,299955	0,999787

_C502310CA	18,76256212	-0,780352103	1,0623286	-0,73456757	0,462603	0,999787
_C502320CA	40,5432487	0,454806113	0,8214364	0,553671752	0,579804	0,999787
_C502330WA	96,10050455	0,373095477	0,7393186	0,50464776	0,613806	0,999787
_C502350CA	7,89844198	0,324541576	1,3076691	0,248183256	0,803993	0,999787
_C502360CA	6,344142678	-0,611736698	1,4543002	-0,42063991	0,674018	0,999787
_C502370CA	28,33697542	0,718282175	1,0021051	0,716773263	0,473514	0,999787
_C502380WA	886,8991532	-0,482642332	0,6752431	-0,71476825	0,474752	0,999787
_C502390CA	50,92915567	-0,790956645	0,7855709	-1,00685578	0,314004	0,999787
_C502400WA	194,5339152	-0,25413981	0,7131272	-0,35637376	0,721561	0,999787
_C502410CA	23,89057784	0,449648561	0,9369512	0,479906059	0,631294	0,999787
_C502440CA	35,63847526	0,523816189	0,8408539	0,622957459	0,533312	0,999787
_C502450WA	67,91485064	-0,185935997	0,8188804	-0,22706124	0,820376	0,999787
_C502480WA	260,6182421	0,337808795	0,70062	0,482156972	0,629694	0,999787
_C502500CA	81,5042254	-0,422230102	0,8490063	-0,49732272	0,618961	0,999787
_C502510CA	68,85829617	-0,673295887	0,8915066	-0,75523375	0,450109	0,999787
_C502560CA	51,74315096	-0,406680107	0,7943766	-0,51194877	0,608687	0,999787
_C502660CA	218,7640703	0,199562249	0,792579	0,251788459	0,801205	0,999787
_C502680WA	16,66442741	-0,734652578	1,0010055	-0,73391466	0,463001	0,999787
_C502730CA	117,2429866	0,25871246	0,7418331	0,348747508	0,727279	0,999787
_C502740WA	50,63983211	0,969784253	1,0360852	0,936008213	0,349269	0,999787
_C502750CA	72,30409658	0,263524777	0,7831479	0,336494266	0,736498	0,999787
_C502780WA	90,27434619	0,522500277	0,9989897	0,523028674	0,600954	0,999787
_C502810WA	7,03259769	0,091889186	1,3352419	0,068818378	0,945134	0,999787
_C502820CA	41,17384223	0,16907051	0,8211923	0,205884192	0,836881	0,999787
_C502830WA	55,26528878	-0,221692199	0,8045167	-0,27555946	0,782886	0,999787
_C502850WA	290,1085453	0,617440627	0,780393	0,791191893	0,428832	0,999787
_C502920WA	36,38360537	0,089900259	0,8334891	0,107860145	0,914107	0,999787
_C502990WA	31,73437887	0,180328123	0,858443	0,21006418	0,833618	0,999787
_C503000CA	55,87331075	0,328146244	0,8500162	0,386047049	0,699462	0,999787
_C503010WA	78,87232946	-0,329170121	0,7530904	-0,43709243	0,662044	0,999787
_C503040WA	80,90318478	-0,217090931	0,789717	-0,27489713	0,783395	0,999787
_C503080CA	45,35128508	-0,234647027	0,8765152	-0,26770446	0,788927	0,999787
_C503100CA	86,69567496	-0,137873946	0,7335376	-0,18795757	0,85091	0,999787
_C503110CA	36,38111331	-0,049509112	0,8391683	-0,05899783	0,952954	0,999787
_C503140CA	570,9545541	-0,547945628	0,6629541	-0,82652125	0,408508	0,999787
_C503150WA	138,194708	0,771229822	0,7089336	1,087873081	0,276651	0,999787
_C503170CA	103,2405207	0,639941493	0,8725811	0,73338916	0,463321	0,999787
_C503260CA	33,75283338	-0,212902302	0,8448128	-0,25201122	0,801032	0,999787
_C503290CA	67,76916066	0,092381096	0,8157557	0,113246025	0,909836	0,999787
_C503300CA	48,78246978	-0,471354941	0,8004697	-0,58884798	0,555963	0,999787
_C503370CA	10,16189305	-1,014895829	1,2992723	-0,78112634	0,434728	0,999787
_C503400CA	172,5209643	-0,658172227	0,7687832	-0,85612198	0,39193	0,999787
_C503410CA	77,07009287	0,52272697	1,0767858	0,48545122	0,627356	0,999787
_C503440WA	36,92291441	0,496220094	1,0016483	0,495403509	0,620315	0,999787
_C503450WA	29,90507727	-1,138736071	1,0395514	-1,09541107	0,273337	0,999787
_C503460CA	12,62486238	0,091102887	1,151508	0,079116155	0,93694	0,999787
_C503550WA	603,0506482	0,445914059	0,801631	0,556258475	0,578034	0,999787
_C503560WA	32,05447307	-0,851082921	0,9453169	-0,90031497	0,367953	0,999787
_C503640WA	16,45894132	0,481423387	1,0101703	0,476576468	0,633664	0,999787
_C503650CA	8,205333226	-0,731159366	1,367704	-0,5345889	0,592934	0,999787

_C503690WA	14,33828299	-0,617082939	1,0644665	-0,57971102	0,56211	0,999787
_C503700CA	48,04192931	0,190509544	0,8312777	0,229176782	0,818732	0,999787
_C503710CA	9,145785601	-0,252801432	1,2630975	-0,20014403	0,841368	0,999787
_C503740WA	12,96812661	-0,950186005	1,0784175	-0,88109289	0,378268	0,999787
_C503780CA	23,58934184	-0,531559912	1,0230998	-0,51955825	0,603372	0,999787
_C503800WA	107,1896083	-0,284580619	0,7512456	-0,37881168	0,704828	0,999787
_C503810CA	11,33719677	-0,194458234	1,1360838	-0,1711654	0,864094	0,999787
_C503830CA	56,73997248	0,012978224	0,7792424	0,016654926	0,986712	0,999787
_C503840WA	14,22068982	-0,406588855	1,0642128	-0,38205598	0,70242	0,999787
_C503850WA	101,3197755	0,608560154	0,743013	0,819043724	0,412761	0,999787
_C503930CA	12,91105803	-0,429519684	1,0944124	-0,39246603	0,694714	0,999787
_C503940CA	17,24985423	0,397300106	1,0323717	0,384842104	0,700354	0,999787
_C503950WA	43,12444231	-0,025189301	0,8506125	-0,02961313	0,976376	0,999787
_C503960WA	105,4943873	-0,562777279	0,743818	-0,75660617	0,449286	0,999787
_C503980WA	82,52546246	0,117643331	0,7769583	0,151415251	0,879648	0,999787
_C504080CA	59,40502958	0,439059998	0,8098698	0,542136548	0,587724	0,999787
_C504120CA	25,56028391	0,972716213	0,9112935	1,067401673	0,28579	0,999787
_C504140WA	5,963530991	-0,401742844	1,4200551	-0,28290653	0,777248	0,999787
_C504150CA	22,50498647	-0,65212726	0,9699379	-0,67233915	0,501368	0,999787
_C504170WA	113,9005413	0,54262475	0,7565106	0,717273119	0,473206	0,999787
_C504180WA	20,32452033	-1,077125289	0,9937251	-1,0839268	0,278397	0,999787
_C504290CA	211,0164237	0,518612365	0,6961031	0,745022314	0,456258	0,999787
_C504300CA	442,797646	-0,610503752	0,7187761	-0,84936567	0,395678	0,999787
_C504310WA	25,56626805	-0,463940586	0,9493046	-0,48871627	0,625043	0,999787
_C504320CA	42,44188261	-0,494934041	0,8180047	-0,60505034	0,545146	0,999787
_C504330WA	82,75397719	-0,024251865	0,7709022	-0,03145907	0,974903	0,999787
_C504340WA	60,52189444	0,885027524	0,7961932	1,111573821	0,266321	0,999787
_C504350CA	128,386776	0,381430881	0,7518238	0,507340792	0,611916	0,999787
_C504410CA	87,27277403	-0,645177561	0,7470388	-0,86364663	0,387782	0,999787
_C504420WA	107,8044412	-0,194075361	0,7420694	-0,26153263	0,793682	0,999787
_C504530WA	81,74129033	0,612615217	0,9858871	0,621384756	0,534346	0,999787
_C504550WA	46,83090001	-0,30089864	0,7977976	-0,3771616	0,706054	0,999787
_C504580CA	25,01179344	0,681227846	0,9091296	0,749318728	0,453665	0,999787
_C504610WA	68,88755398	0,463139634	0,7803886	0,593473061	0,552865	0,999787
_C504620CA	40,41768503	0,611823227	0,9546885	0,640861621	0,521613	0,999787
_C504630WA	46,45907627	-0,765545115	0,818284	-0,93554941	0,349505	0,999787
_C504640CA	410,703521	0,148303484	0,6999914	0,21186473	0,832213	0,999787
_C504710WA	77,25645876	-0,313945903	0,7967169	-0,39404952	0,693544	0,999787
_C504720CA	147,3336539	-0,110229065	0,7204469	-0,15300096	0,878398	0,999787
_C504770WA	52,57694235	0,210230601	0,8067835	0,260578693	0,794417	0,999787
_C504790CA	19,55370341	0,617314144	0,98153	0,628930483	0,529395	0,999787
_C504840CA	32,51159007	0,955151052	0,8618905	1,108204609	0,267773	0,999787
_C504860CA	9,458784083	-0,435353603	1,2052649	-0,36120991	0,717943	0,999787
_C504890CA	12,56030575	0,129214143	1,1087293	0,116542548	0,907223	0,999787
_C504920CA	209,5701117	-0,138284135	0,7363532	-0,18779594	0,851037	0,999787
_C504950CA	63,46147585	0,522620305	0,8628257	0,605707837	0,544709	0,999787
_C504960WA	156,1230723	0,477619389	0,7175838	0,665593839	0,505671	0,999787
_C504980WA	6,256091568	0,030102879	1,404534	0,021432646	0,982901	0,999787
_C504990WA	45,38146185	0,014196176	0,8488774	0,016723469	0,986657	0,999787
_C505000CA	79,65638789	0,015866801	0,7589619	0,020905926	0,983321	0,999787

_C505020CA	101,4295398	-0,081862111	0,7422781	-0,11028497	0,912183	0,999787
_C505070WA	36,60147581	-0,007038265	0,8582643	-0,00820058	0,993457	0,999787
_C505090WA	10,31053894	0,332006836	1,1761693	0,282278105	0,77773	0,999787
_C505130CA	45,83386628	-0,082619342	0,8168883	-0,10113909	0,91944	0,999787
_C505140WA	35,75929587	-0,043024867	0,8371147	-0,05139662	0,959009	0,999787
_C505150CA	17,13846205	0,621537475	1,0298458	0,60352481	0,54616	0,999787
_C505210WA	62,49836982	-0,10883443	0,7632621	-0,14259116	0,886613	0,999787
_C505230CA	1921,613948	0,283945369	0,6602388	0,430064647	0,667149	0,999787
_C505250CA	180,7412078	0,888912126	0,8013753	1,109233217	0,26733	0,999787
_C505270CA	290,1133497	0,363671123	0,735363	0,494546412	0,62092	0,999787
_C505350WA	99,09959153	0,291499578	0,7374033	0,395305478	0,692617	0,999787
_C600080CA	79,63823146	-0,480724161	0,8913363	-0,53932969	0,589659	0,999787
_C600090WA	113,5054363	-0,321900806	0,8082555	-0,39826613	0,690434	0,999787
_C600110CA	126,9454804	0,153292816	0,8199771	0,186947685	0,851702	0,999787
_C600170CA	432,2777262	0,224473846	0,6778682	0,331146765	0,740534	0,999787
_C600190WA	285,1030182	0,041468908	0,7333944	0,056543803	0,954909	0,999787
_C600210WA	208,687457	-0,638425702	0,8054647	-0,79261787	0,428	0,999787
_C600240CA	55,24235421	-0,594688492	0,8346922	-0,71246442	0,476177	0,999787
_C600250WA	135,2997556	-0,489352027	0,7912087	-0,61848669	0,536255	0,999787
_C600260WA	235,3312458	0,052240436	0,6949162	0,07517516	0,940075	0,999787
_C600270WA	51,15747005	-0,096206077	0,7854672	-0,12248262	0,902517	0,999787
_C600310WA	207,4886763	-0,342415399	0,7399221	-0,46277223	0,643528	0,999787
_C600340CA	168,2585211	0,59513759	0,7085297	0,839961401	0,40093	0,999787
_C600360CA	23,05226601	-0,105316251	0,91884	-0,1146187	0,908747	0,999787
_C600380CA	60,88646212	-0,798874704	0,927589	-0,86123782	0,389107	0,999787
_C600390WA	104,1979434	-0,114888559	0,720561	-0,1594432	0,87332	0,999787
_C600400CA	37,22932435	-0,07336641	0,8502076	-0,08629234	0,931234	0,999787
_C600430CA	85,24787847	-0,714652741	0,7426069	-0,96235678	0,33587	0,999787
_C600490WA	15,22497036	-0,482666248	1,053538	-0,45813844	0,646853	0,999787
_C600500CA	31,50773926	-0,1712616	0,891848	-0,19203004	0,847719	0,999787
_C600520WA	10,65775216	0,359905353	1,1806963	0,304824664	0,7605	0,999787
_C600530CA	10,30377936	0,168184324	1,1966878	0,140541521	0,888232	0,999787
_C600540WA	37,43625452	0,416372419	0,8359612	0,498076237	0,61843	0,999787
_C600560WA	51,28339789	0,547122272	0,8328981	0,656889834	0,511252	0,999787
_C600640CA	35,10542571	0,822219256	0,8509612	0,966224185	0,333932	0,999787
_C600660CA	74,3247248	0,352496982	0,7527034	0,46830793	0,639564	0,999787
_C600680CA	35,35751089	0,749680415	0,8472747	0,884813841	0,376257	0,999787
_C600690WA	41,24304816	0,639136578	0,8173822	0,781931074	0,434255	0,999787
_C600700CA	49,38684722	-0,904799862	0,7970986	-1,13511654	0,256326	0,999787
_C600760WA	75,5923236	0,627326759	0,8177778	0,767111465	0,443015	0,999787
_C600770CA	70,36960839	0,042190572	0,755336	0,055856694	0,955456	0,999787
_C600800CA	105,8674396	-0,569372063	0,7279924	-0,78211263	0,434148	0,999787
_C600850WA	116,2387012	0,165670533	0,725185	0,22845279	0,819294	0,999787
_C600890WA	82,53493306	-0,808075575	0,7410749	-1,0904101	0,275533	0,999787
_C600900CA	23,74386542	0,09261966	0,9209451	0,100570225	0,919892	0,999787
_C600910CA	409,6747154	-0,445566055	0,6749465	-0,66015018	0,509157	0,999787
_C600920WA	334,9968591	0,562253295	0,7002713	0,802907774	0,422028	0,999787
_C600940CA	27,33554647	-0,920821271	0,9317479	-0,98827295	0,323019	0,999787
_C601000CA	57,19139435	0,495951719	0,8901506	0,55715485	0,577422	0,999787
_C601050WA	75,83912459	0,227764395	0,9488641	0,240039002	0,8103	0,999787

_C601080CA	17,27077937	0,6395013	1,0255464	0,623571303	0,532909	0,999787
_C601090CA	6,785502558	1,166897941	1,5216238	0,766876779	0,443155	0,999787
_C601120CA	5,190727862	-0,486578901	1,499166	-0,32456639	0,745509	0,999787
_C601200WA	6,791062894	0,54956557	1,4923259	0,368261094	0,712679	0,999787
_C601210WA	71,98709637	-0,29190654	0,7545061	-0,38688425	0,698842	0,999787
_C601220CA	116,0496568	-0,035129615	0,9224435	-0,03808322	0,969621	0,999787
_C601300WA	19,94181007	-0,50891018	0,9597208	-0,53026897	0,595925	0,999787
_C601330CA	10,16044089	-0,09344005	1,1762321	-0,07944014	0,936683	0,999787
_C601340CA	31,34740225	-0,034153863	0,9077675	-0,03762402	0,969987	0,999787
_C601350WA	117,2908712	-0,18647103	0,7420023	-0,25130789	0,801576	0,999787
_C601400WA	6,335208401	-0,928385945	1,5308652	-0,60644527	0,544219	0,999787
_C601420CA	84,234474	-0,057695192	0,7346065	-0,07853891	0,937399	0,999787
_C601460CA	83,17929422	0,879384573	0,7868154	1,117650477	0,263716	0,999787
_C601480WA	5,60505537	-0,123531219	1,4643017	-0,08436186	0,932769	0,999787
_C601550CA	92,87753547	-0,053164654	0,7422478	-0,07162656	0,942899	0,999787
_C601620WA	66,69398689	-0,092649979	0,7921424	-0,11696127	0,906891	0,999787
_C601850WA	479,7273304	-0,710299534	0,8367142	-0,84891533	0,395928	0,999787
_C601870CA	26,605534	0,333541577	0,8980037	0,371425629	0,710321	0,999787
_C601880WA	20,72910781	-0,5977335	0,9485604	-0,63014808	0,528598	0,999787
_C601920CA	96,32908455	0,27401827	0,7291626	0,375798571	0,707067	0,999787
_C601940WA	13,49279355	0,280211753	1,1058608	0,2533879	0,799968	0,999787
_C601960WA	26,39627455	0,530648764	0,9741144	0,54474996	0,585925	0,999787
_C601980CA	267,4385869	0,526560403	0,6820135	0,772067373	0,440075	0,999787
_C602030CA	76,16654305	-0,289403481	0,7826104	-0,36979255	0,711537	0,999787
_C602090CA	87,16000469	0,752828315	0,9062021	0,830750983	0,406114	0,999787
_C602110WA	9,618786111	0,226660114	1,1995366	0,188956393	0,850127	0,999787
_C602140WA	79,36321698	-0,634653657	0,7849138	-0,80856473	0,418766	0,999787
_C602150CA	15,37788376	0,135259594	1,0392007	0,130157332	0,896442	0,999787
_C602160WA	29,55511773	0,00255105	0,9336754	0,002732267	0,99782	0,999787
_C602180WA	32,33721749	-0,828695709	0,8997872	-0,92099082	0,357055	0,999787
_C602190CA	83,11817909	-0,434874969	0,8175207	-0,53194369	0,594765	0,999787
_C602210WA	31,69590846	-0,067103129	0,9422692	-0,07121439	0,943227	0,999787
_C602280WA	101,6937798	0,759547992	0,7569214	1,003470062	0,315634	0,999787
_C602300CA	22,83840423	0,734497024	0,9338336	0,786539504	0,431551	0,999787
_C602310WA	80,09359972	-0,657389375	0,7418923	-0,88609817	0,375565	0,999787
_C602350CA	96,65158299	0,008261768	0,7314922	0,011294403	0,990989	0,999787
_C602370CA	72,9605946	0,133898524	1,0462783	0,127976014	0,898168	0,999787
_C602410WA	75,38195289	0,603091509	0,746701	0,807674667	0,419278	0,999787
_C602420WA	8,538857431	-1,425767512	1,4748459	-0,96672306	0,333682	0,999787
_C602430WA	345,1009517	0,405085204	0,6766647	0,598649818	0,549406	0,999787
_C602460CA	98,07830078	0,510790596	0,8463128	0,603548243	0,546144	0,999787
_C602470WA	167,8392278	-0,758946125	0,6940331	-1,09353023	0,274161	0,999787
_C602490CA	32,95160422	0,443373418	1,060733	0,417987764	0,675956	0,999787
_C602530CA	26,15475145	0,295856939	0,9543507	0,310008609	0,756554	0,999787
_C602540CA	6,385023252	0,671512036	1,4174404	0,473749746	0,635678	0,999787
_C602580WA	53,03373259	-0,90486397	0,84301	-1,07337276	0,283104	0,999787
_C602630CA	127,3296074	0,288057638	0,7120726	0,404534079	0,68582	0,999787
_C602640CA	69,52718146	0,138889113	0,7869799	0,176483677	0,859914	0,999787
_C602650CA	58,22679333	-0,636865046	0,8750419	-0,72781087	0,466729	0,999787
_C602680WA	12,97730212	-1,152627764	1,0942829	-1,05331794	0,292195	0,999787

_C602690CA	579,3280586	-0,592887257	0,7038623	-0,84233413	0,399601	0,999787
_C602720CA	28,06053842	-0,552016939	0,8850691	-0,62369925	0,532825	0,999787
_C602740WA	192,1853642	-0,072010364	0,6934084	-0,10384986	0,917288	0,999787
_C602810CA	99,96172907	-0,036792705	0,8360395	-0,04400833	0,964898	0,999787
_C602930WA	289,3366636	0,295510314	0,7062056	0,418448016	0,67562	0,999787
_C602940CA	54,70770008	-0,541592955	0,7842384	-0,69059732	0,489819	0,999787
_C602960WA	33,50058193	0,875728317	0,9163836	0,955635132	0,339257	0,999787
_C602970CA	26,30590818	-0,365089614	0,8883307	-0,41098391	0,681084	0,999787
_C602980CA	145,718673	-0,397187888	0,7417445	-0,53547803	0,592319	0,999787
_C602990WA	45,25625178	-0,901001168	0,8140271	-1,10684415	0,268361	0,999787
_C603000CA	7,934015143	0,098793355	1,2757934	0,077436795	0,938276	0,999787
_C603040CA	25,35908972	-0,025690581	0,9263603	-0,02773282	0,977875	0,999787
_C603050CA	17,38814416	-1,167573661	1,1176329	-1,04468439	0,296169	0,999787
_C603110CA	8,396311851	0,826408933	1,2999625	0,635717523	0,524961	0,999787
_C603130WA	192,6227714	0,291263286	0,7319097	0,397949766	0,690667	0,999787
_C603150CA	43,70958108	-0,285249772	0,8375929	-0,34055897	0,733436	0,999787
_C603180CA	159,5030378	0,008273244	0,6990773	0,01183452	0,990558	0,999787
_C603220WA	24,46924509	1,20246873	1,136005	1,058506563	0,289825	0,999787
_C603260WA	85,64154217	0,611223641	0,7383373	0,827837905	0,407762	0,999787
_C603300CA	53,70230813	-0,645199497	0,7923808	-0,81425431	0,415499	0,999787
_C603310WA	62,70112262	-0,456501142	0,7759539	-0,5883096	0,556325	0,999787
_C603320WA	60,12062916	0,018124479	0,8139825	0,022266425	0,982235	0,999787
_C603330CA	21,59736803	-0,697352735	0,9353408	-0,74556008	0,455933	0,999787
_C603360CA	8,888480828	-1,356634836	1,2618035	-1,07515539	0,282305	0,999787
_C603380WA	498,9503862	0,403626006	0,6968523	0,579213097	0,562445	0,999787
_C603390WA	95,71090668	-0,099083984	0,8051826	-0,12305778	0,902061	0,999787
_C603430CA	105,5136835	-0,211639528	0,7771158	-0,27233976	0,785361	0,999787
_C603460WA	346,5434976	0,155075192	0,7039918	0,22027982	0,825653	0,999787
_C603470WA	113,8053873	-0,457815985	0,7310571	-0,62623832	0,531159	0,999787
_C603520CA	5,868402947	-0,804673641	1,4401492	-0,55874325	0,576337	0,999787
_C603530CA	23,13114161	-0,171360711	0,9460879	-0,18112556	0,856269	0,999787
_C603540WA	63,22591842	0,402545542	0,7644805	0,526560893	0,598499	0,999787
_C603550CA	439,1150472	0,115141016	0,7755407	0,148465464	0,881975	0,999787
_C603580WA	15,05378206	-0,214811473	1,0748995	-0,19984331	0,841603	0,999787
_C603590CA	25,21462236	0,193852525	0,9215951	0,210344561	0,833399	0,999787
_C603620CA	66,80811237	-0,1235159	0,7615809	-0,16218357	0,871161	0,999787
_C603730CA	120,8663558	0,173210103	1,0409115	0,166402328	0,86784	0,999787
_C603780CA	55,976216	-0,738029738	0,7821602	-0,94357872	0,345385	0,999787
_C603800CA	36,92711156	0,03275354	0,9515849	0,034419987	0,972542	0,999787
_C603810WA	47,13183743	-0,704875395	0,8017639	-0,87915577	0,379317	0,999787
_C603820CA	30,29195421	-0,405355727	0,8623928	-0,47003606	0,638329	0,999787
_C603910CA	31,17427356	0,812597208	0,8670192	0,937230942	0,34864	0,999787
_C603950CA	86,47767454	0,583068218	0,7808485	0,746711102	0,455238	0,999787
_C603960WA	541,1074174	0,302943672	0,696313	0,435068248	0,663513	0,999787
_C603990CA	11,04809796	-0,002326259	1,1342115	-0,00205099	0,998364	0,999787
_C604050WA	31,87619598	-0,520555855	0,9309847	-0,55914544	0,576062	0,999787
_C604060WA	66,29598302	-0,100702618	0,8964591	-0,11233375	0,910559	0,999787
_C604080WA	769,5331961	-0,059928866	0,716727	-0,08361463	0,933363	0,999787
_C604100WA	282,7796088	-0,037669417	0,6857063	-0,05493521	0,95619	0,999787
_C604110WA	73,1213615	-0,511690842	0,7559032	-0,67692638	0,498453	0,999787

_C604140CA	114,5293149	-0,158977422	0,716943	-0,22174345	0,824514	0,999787
_C604180WA	120,736984	0,311595911	0,8318918	0,374563019	0,707985	0,999787
_C604190CA	6,339228754	-0,047159318	1,3850576	-0,03404863	0,972838	0,999787
_C604230WA	11,98598214	-0,952538017	1,2516056	-0,76105286	0,446625	0,999787
_C604240WA	10,03166145	1,191573369	1,2386819	0,961968819	0,336065	0,999787
_C604250WA	531,720247	-0,107699728	0,6627995	-0,16249217	0,870918	0,999787
_C604280WA	111,0639036	0,053584041	0,7551875	0,070954615	0,943434	0,999787
_C604290WA	417,1776148	0,342810141	0,6676496	0,513458172	0,607631	0,999787
_C604300WA	38,35828091	-0,171489744	0,8640927	-0,1984622	0,842683	0,999787
_C604340WA	24,81743494	0,095619351	0,9433068	0,101366125	0,91926	0,999787
_C604370WA	20,66736653	-0,299593086	0,9465786	-0,316501	0,751622	0,999787
_C604400WA	10,97413628	-0,207982926	1,14063	-0,18234039	0,855316	0,999787
_C604410CA	46,08080323	-0,525040891	0,7993547	-0,65683097	0,51129	0,999787
_C604420WA	57,55499018	-0,525478993	0,9842403	-0,53389298	0,593416	0,999787
_C604440CA	100,4628666	-0,365939038	0,7561284	-0,48396412	0,628411	0,999787
_C604510CA	46,68351663	0,381667372	0,8086467	0,471982823	0,636939	0,999787
_C604560WA	1039,838329	-0,605130107	0,7997629	-0,75663685	0,449267	0,999787
_C700010CA	290,1825754	0,504466083	0,6947686	0,726092226	0,467782	0,999787
_C700040CA	109,3194728	-0,649189375	0,9753595	-0,66558981	0,505673	0,999787
_C700130WA	71,95928772	-0,766882439	0,8947926	-0,85705052	0,391417	0,999787
_C700140CA	157,9656484	0,001055937	0,8194144	0,001288649	0,998972	0,999787
_C700150WA	432,1532007	-0,27218422	0,7420418	-0,36680441	0,713765	0,999787
_C700190WA	240,8591025	-0,016298348	0,6823169	-0,02388677	0,980943	0,999787
_C700210CA	64,23334336	0,368008834	0,7640837	0,481634172	0,630066	0,999787
_C700220WA	36,60573639	0,619986981	0,8805037	0,704127646	0,481353	0,999787
_C700230WA	55,04620164	-0,001634459	0,7740327	-0,00211162	0,998315	0,999787
_C700240WA	54,60659959	-0,117632334	0,7781859	-0,15116226	0,879848	0,999787
_C700250CA	374,9804116	0,192962342	0,7151881	0,269806407	0,787309	0,999787
_C700270WA	162,0452917	-0,877469565	0,8574662	-1,02332841	0,306153	0,999787
_C700340CA	415,1164712	-0,019732681	0,6707128	-0,02942046	0,976529	0,999787
_C700380WA	70,83037104	0,19234667	0,7544512	0,254949137	0,798762	0,999787
_C700410CA	105,579991	0,065575356	0,7468821	0,087798811	0,930037	0,999787
_C700420CA	24,60254496	-0,06953883	0,9270404	-0,07501165	0,940205	0,999787
_C700450CA	34,75921249	-0,505213388	0,8366188	-0,60387524	0,545927	0,999787
_C700490CA	24,05590014	-0,156318763	0,9181006	-0,17026322	0,864803	0,999787
_C700600CA	73,60046808	-0,046258639	0,7491505	-0,06174812	0,950763	0,999787
_C700610CA	81,87861648	0,202180752	0,7532817	0,268399911	0,788392	0,999787
_C700630CA	19,42477173	0,397080586	0,9639019	0,411951254	0,680375	0,999787
_C700670CA	120,6932599	-0,395298384	0,7359503	-0,53712647	0,59118	0,999787
_C700680WA	9,520272433	0,377669798	1,2181465	0,310036447	0,756533	0,999787
_C700780WA	91,98157075	0,533410825	0,7450342	0,715954855	0,474019	0,999787
_C700790WA	29,28736208	-0,694495758	0,9001922	-0,77149719	0,440412	0,999787
_C700810WA	88,17576543	0,026355958	0,7404639	0,035593843	0,971606	0,999787
_C700830CA	299,3342414	-0,286489026	0,6798012	-0,42143062	0,673441	0,999787
_C700840CA	44,36750096	0,278946563	0,8507971	0,327864952	0,743014	0,999787
_C700850WA	18,87451289	-0,933267724	1,0036849	-0,92984131	0,352453	0,999787
_C700910CA	51,19255598	-0,263951893	0,795259	-0,33190684	0,73996	0,999787
_C700920CA	13,83156073	-0,84795347	1,125097	-0,7536714	0,451047	0,999787
_C700940WA	152,1675985	-0,081492053	0,6973266	-0,11686354	0,906968	0,999787
_C701040CA	45,76607627	0,43315864	0,8164871	0,530514962	0,595755	0,999787

_C701100CA	11,50903148	-0,602791605	1,1266784	-0,53501659	0,592638	0,999787
_C701160CA	73,72344002	-0,036814812	0,7759948	-0,04744209	0,962161	0,999787
_C701180WA	62,59027157	-0,149506008	0,8071882	-0,18521828	0,853058	0,999787
_C701190WA	29,51865692	0,187052545	0,8741257	0,213988149	0,830556	0,999787
_C701230CA	72,28047439	-0,533987674	0,7495053	-0,71245352	0,476184	0,999787
_C701270CA	74,74081474	0,687003454	0,773673	0,887976543	0,374553	0,999787
_C701280CA	27,32023325	0,646984822	0,9173586	0,705269265	0,480643	0,999787
_C701320WA	61,77739922	-0,478026937	0,7728776	-0,61850274	0,536244	0,999787
_C701350CA	26,93367351	0,195436639	1,0806618	0,180849024	0,856486	0,999787
_C701360CA	6,411344779	-0,481818059	1,3925037	-0,34600846	0,729336	0,999787
_C701370WA	15,14246213	0,52849287	1,0624486	0,497429125	0,618886	0,999787
_C701430CA	18,61333885	0,424423331	0,9918212	0,427923223	0,668707	0,999787
_C701440WA	9,507711289	1,084106023	1,255653	0,863380252	0,387928	0,999787
_C701460CA	27,91299686	0,149651212	1,0831682	0,138160646	0,890113	0,999787
_C701480WA	40,41416477	-0,105904023	0,8304169	-0,12753115	0,89852	0,999787
_C701490WA	68,45327103	0,373627578	0,7660186	0,487752633	0,625725	0,999787
_C701500WA	11,6520526	-0,737983278	1,1247508	-0,65613049	0,51174	0,999787
_C701510WA	70,0716829	-0,784759978	0,7509984	-1,04495557	0,296044	0,999787
_C701570CA	12,98517195	1,090460214	1,1626839	0,937881943	0,348305	0,999787
_C701590CA	30,44301604	-0,083800671	0,9604995	-0,08724697	0,930475	0,999787
_C701600WA	184,3913238	0,439834956	0,9100113	0,483329116	0,628862	0,999787
_C701610WA	110,192875	-0,363891179	0,7209845	-0,50471428	0,61376	0,999787
_C701660CA	140,8977772	0,793842777	0,8667862	0,915846111	0,359748	0,999787
_C701670WA	14,02068794	0,103929878	1,056622	0,098360506	0,921646	0,999787
_C701680CA	18,95996534	0,564655794	1,2169597	0,463988886	0,642656	0,999787
_C701690WA	13,66776096	0,636417667	1,0709563	0,594251733	0,552344	0,999787
_C701700WA	66,71505142	0,732692196	0,762449	0,960972082	0,336566	0,999787
_C701710WA	83,89209841	-0,180498421	0,7697439	-0,23449151	0,814603	0,999787
_C701720WA	38,8964024	-0,175422611	0,8262799	-0,2123041	0,83187	0,999787
_C701750WA	26,46678616	0,071897224	0,9064779	0,079314925	0,936782	0,999787
_C701830WA	23,52261628	-0,678376449	0,9236487	-0,73445292	0,462673	0,999787
_C701880CA	13,06074974	1,273675882	1,1191757	1,138048239	0,2551	0,999787
_C701910CA	18,61623149	0,069000971	1,0107859	0,068264672	0,945575	0,999787
_C701930CA	99,84193101	-0,35821395	0,7528316	-0,47582218	0,634201	0,999787
_C701950WA	43,88789789	0,932711095	0,8217123	1,135082246	0,256341	0,999787
_C701990CA	29,95891131	0,223738618	0,8969504	0,249443683	0,803018	0,999787
_C702090CA	20,88587762	-0,245190766	0,9510834	-0,25780155	0,79656	0,999787
_C702100WA	462,6751106	0,661063401	0,7504097	0,880936587	0,378352	0,999787
_C702110WA	104,5332569	-0,752861031	0,7238723	-1,0400467	0,298318	0,999787
_C702120CA	44,08564302	0,668718356	0,9561925	0,699355344	0,48433	0,999787
_C702140WA	6,065430302	-1,566953511	1,535296	-1,02061984	0,307435	0,999787
_C702170CA	73,64192603	-0,424086271	0,7520127	-0,56393495	0,572798	0,999787
_C702310CA	11,38194545	-0,552435569	1,1388061	-0,48510065	0,627605	0,999787
_C702340CA	41,98123713	0,394553956	0,9421686	0,418772142	0,675383	0,999787
_C702350CA	68,29464505	0,028670775	0,7530469	0,038073029	0,969629	0,999787
_C702420CA	29,38457701	-0,564768317	0,9185959	-0,61481693	0,538676	0,999787
_C702430CA	13,91742545	-0,43644339	1,0959091	-0,3982478	0,690448	0,999787
_C702470CA	63,40986921	-0,833142759	0,8021587	-1,03862585	0,298979	0,999787
_C702480WA	104,9122024	0,225134237	0,7205394	0,312452381	0,754697	0,999787
_C702510WA	104,897504	-0,421635164	0,7386101	-0,57084945	0,568102	0,999787

_C702530CA	33,26365063	-0,046194441	0,8911991	-0,05183403	0,958661	0,999787
_C702540WA	21,76894981	-0,241195409	0,9675231	-0,24929162	0,803135	0,999787
_C702550CA	29,42167154	0,265405536	0,8793818	0,301809236	0,762797	0,999787
_C702560WA	38,21582415	-0,222930885	0,8354425	-0,26684168	0,789591	0,999787
_C702630WA	49,09966442	-0,450968014	0,8172717	-0,55179695	0,581087	0,999787
_C702660CA	7,844676975	-0,008733335	1,4515889	-0,0060164	0,9952	0,999787
_C702670WA	14,80749845	-0,570929357	1,1904499	-0,47959124	0,631518	0,999787
_C702690CA	24,66572078	-0,065973489	0,9317633	-0,07080499	0,943553	0,999787
_C702780WA	642,2697457	0,294498989	0,6691684	0,44009695	0,659867	0,999787
_C702850WA	82,26550528	0,290690916	0,7384897	0,393628934	0,693855	0,999787
_C702860CA	238,1716298	0,627156842	0,7151495	0,876959084	0,380509	0,999787
_C702930CA	177,4555527	0,42774142	0,6957173	0,614820732	0,538673	0,999787
_C702960CA	54,51065887	0,473926584	0,7858404	0,603082487	0,546454	0,999787
_C703000CA	57,06793403	0,745488692	0,9961312	0,748384003	0,454229	0,999787
_C703010WA	950,0210173	-0,577165341	0,7505253	-0,76901515	0,441884	0,999787
_C703040WA	8,981839348	-0,56182769	1,2459308	-0,45093008	0,65204	0,999787
_C703050WA	51,42506059	0,563394018	0,7879869	0,714978883	0,474622	0,999787
_C703160WA	126,7894236	-0,058936512	0,7210945	-0,08173202	0,93486	0,999787
_C703210WA	37,26336922	-0,115133016	0,8720874	-0,13202004	0,894968	0,999787
_C703230CA	13,89715265	-1,189012327	1,1123658	-1,06890409	0,285113	0,999787
_C703240WA	115,5794379	-0,679092571	0,713214	-0,95215823	0,341017	0,999787
_C703260CA	25,01769568	-0,98974809	0,9290297	-1,06535673	0,286715	0,999787
_C703270WA	602,1179839	0,755042755	0,9908893	0,761984946	0,446069	0,999787
_C703310WA	19,56652341	-0,752330944	1,2167254	-0,61832435	0,536362	0,999787
_C703370CA	105,8887524	-0,356556856	0,72065	-0,49477119	0,620762	0,999787
_C703400CA	237,0106824	1,099830116	1,028578	1,06927247	0,284947	0,999787
_C703410CA	40,65761657	-0,22547176	0,8374824	-0,26922566	0,787756	0,999787
_C703480WA	70,93489228	0,456222483	0,9544055	0,478017451	0,632638	0,999787
_C703500WA	34,63314773	0,947229935	0,9112136	1,039525729	0,29856	0,999787
_C703580CA	22,22715004	-0,592564342	0,9240394	-0,64127609	0,521343	0,999787
_C703590CA	42,53618728	0,147544481	0,979299	0,150663367	0,880241	0,999787
_C703610CA	83,25673268	0,086595385	0,8078953	0,107186401	0,914641	0,999787
_C703620CA	197,3025322	-0,713282925	0,8174983	-0,87251914	0,382925	0,999787
_C703640CA	56,35067828	0,141288343	0,7843362	0,180137473	0,857045	0,999787
_C703650WA	38,72947569	-0,027548436	0,8613616	-0,03198243	0,974486	0,999787
_C703660CA	811,6693626	0,426234462	0,7876598	0,54114027	0,588411	0,999787
_C703680WA	11,90597174	0,312112955	1,1966914	0,260813237	0,794237	0,999787
_C703690WA	8,163241721	-1,350643911	1,320189	-1,02306861	0,306275	0,999787
_C703730CA	35,21209938	-0,865941455	0,8503192	-1,01837221	0,308501	0,999787
_C703760WA	28,96718023	0,405530723	0,8895947	0,455860072	0,648491	0,999787
_C703800WA	28,20571562	0,665158158	0,8978379	0,740844406	0,458788	0,999787
_C703830CA	31,07636048	-0,925571827	0,8722224	-1,06116499	0,288615	0,999787
_C703840WA	79,76063394	-0,542473391	0,7621202	-0,71179508	0,476592	0,999787
_C703860WA	138,3322376	-0,839877684	0,7723715	-1,08740125	0,27686	0,999787
_C703880CA	52,0231504	-0,230832661	0,7995476	-0,28870411	0,772808	0,999787
_C703890CA	584,1505192	-0,0409134	0,76204	-0,05368931	0,957183	0,999787
_C703970CA	85,84525526	-0,997734231	0,8947043	-1,11515533	0,264784	0,999787
_C703980WA	27,96242415	-0,170122091	0,8766866	-0,1940512	0,846136	0,999787
_C704000WA	135,7294665	-0,687707044	0,7768178	-0,88528748	0,376002	0,999787
_C704010WA	208,3546048	0,367789642	0,6910744	0,532199773	0,594588	0,999787

_C704060WA	14,97795728	0,330092198	1,1507192	0,286857289	0,774222	0,999787
_C704090CA	73,72270362	0,533276374	0,7564613	0,704961891	0,480834	0,999787
_C704110WA	49,10721096	0,161646442	0,7891104	0,204846416	0,837692	0,999787
_C704120WA	15,55029468	-1,114799332	1,0674873	-1,04432093	0,296337	0,999787
_C704170WA	9,921004666	-0,6483358	1,1769184	-0,55087573	0,581719	0,999787
_C704200CA	204,870446	0,223807557	0,9600736	0,233115004	0,815672	0,999787
_C704210CA	626,6924972	-0,302649472	0,7572372	-0,3996759	0,689395	0,999787
_C704240CA	232,9443051	0,283759617	0,6853823	0,414016562	0,678862	0,999787
_C704260WA	527,2495582	-0,398653064	0,7568755	-0,52670888	0,598396	0,999787
_CR00010CA	45,61670669	0,416772366	0,805068	0,517685891	0,604677	0,999787
_CR00040CA	44,19021712	-0,004333395	0,8027453	-0,00539822	0,995693	0,999787
_CR00060CA	591,1560242	-0,352711126	0,7319839	-0,48185637	0,629908	0,999787
_CR00130CA	168,5856266	-0,657303544	0,7901499	-0,83187193	0,405481	0,999787
_CR00220WA	19,75790838	-0,915230739	1,01198	-0,90439603	0,365785	0,999787
_CR00230WA	468,7770412	0,383177419	0,7684309	0,498649161	0,618027	0,999787
_CR00280CA	37,57677764	0,365627497	0,8746296	0,418036944	0,67592	0,999787
_CR00290WA	119,1326607	0,413800448	0,8435357	0,490554769	0,623741	0,999787
_CR00340CA	47,10938301	-0,389545406	0,9652086	-0,40358677	0,686517	0,999787
_CR00370WA	101,8856423	-0,230974276	0,7630936	-0,30268146	0,762133	0,999787
_CR00380WA	10,11030275	-1,157630777	1,2327085	-0,93909532	0,347682	0,999787
_CR00420WA	27,51984758	-0,305988679	1,0057082	-0,30425194	0,760936	0,999787
_CR00430CA	44,25525621	-0,220755313	0,811256	-0,27211547	0,785533	0,999787
_CR00470WA	194,2122595	-0,088280639	0,7624617	-0,1157837	0,907824	0,999787
_CR00490WA	207,043099	0,38792089	0,754929	0,513850821	0,607356	0,999787
_CR00570WA	176,2581829	0,506749289	0,8172239	0,620086241	0,535201	0,999787
_CR00630WA	27,44135377	0,645886938	0,8929364	0,723329149	0,469478	0,999787
_CR00660WA	7,787049798	0,904582002	1,4083122	0,642316391	0,520668	0,999787
_CR00690CA	28,95462601	-0,269668593	0,8809083	-0,30612562	0,759509	0,999787
_CR00750CA	38,76859383	-0,394903595	0,8277482	-0,47708179	0,633304	0,999787
_CR00810WA	19,6927803	0,726195404	0,9681533	0,750083067	0,453205	0,999787
_CR00820CA	48,88349907	-0,900155721	0,8921137	-1,00901452	0,312968	0,999787
_CR00870CA	5,64771716	-1,073422377	1,4617987	-0,73431612	0,462756	0,999787
_CR00880WA	13,02864526	-0,286755182	1,1940352	-0,2401564	0,810209	0,999787
_CR00890CA	19,51024872	-0,272751412	0,9751893	-0,27969073	0,779715	0,999787
_CR00910WA	768,767781	-0,602613229	0,673457	-0,89480583	0,370891	0,999787
_CR01020CA	32,51817977	0,88472684	0,9978029	0,886674914	0,375254	0,999787
_CR01050CA	41,00891459	0,36557713	0,9152898	0,399411333	0,68959	0,999787
_CR01070WA	5,788880936	-0,501753102	1,4417324	-0,34802098	0,727824	0,999787
_CR01080WA	60,51218112	-0,356027791	0,8495472	-0,41907948	0,675158	0,999787
_CR01100CA	70,99906611	0,357246176	0,7660079	0,466374	0,640948	0,999787
_CR01120CA	176,4894522	-0,499269437	0,7781307	-0,64162668	0,521116	0,999787
_CR01130WA	120,7467698	0,061436497	0,7090716	0,086643572	0,930955	0,999787
_CR01170WA	246,7728312	-0,167425397	0,6894602	-0,24283548	0,808133	0,999787
_CR01180WA	27,10165297	-0,646434361	0,9450118	-0,684049	0,493944	0,999787
_CR01280CA	26,63338958	-0,123838658	0,9466878	-0,13081256	0,895924	0,999787
_CR01300WA	151,4900416	0,136415606	0,7425753	0,183706081	0,854244	0,999787
_CR01320CA	169,15663	0,563991595	0,7729996	0,729614305	0,465626	0,999787
_CR01350CA	201,0003859	0,02484511	0,718491	0,034579571	0,972415	0,999787
_CR01360WA	15,20878235	0,466548967	1,1425078	0,408355165	0,683013	0,999787
_CR01370CA	226,7116003	0,924378321	0,8537167	1,082769445	0,278911	0,999787

_CR01410CA	151,409829	0,26635831	0,7231921	0,368309218	0,712643	0,999787
_CR01490CA	225,9447264	-0,021555357	0,6881559	-0,03132336	0,975012	0,999787
_CR01550CA	11,79240485	0,659401136	1,1313205	0,582859727	0,559988	0,999787
_CR01560WA	41,50931292	-0,663386974	0,8771408	-0,75630612	0,449466	0,999787
_CR01570WA	39,62629081	0,307320449	0,828256	0,37104526	0,710604	0,999787
_CR01600CA	31,26884878	0,671063897	0,9046424	0,741800149	0,458208	0,999787
_CR01630CA	8,881227093	0,65255693	1,2387976	0,526766383	0,598356	0,999787
_CR01640CA	5,973993558	-0,050500498	1,4133149	-0,03573195	0,971496	0,999787
_CR01670WA	21,84130707	-0,565976918	0,9493006	-0,59620412	0,551039	0,999787
_CR01720WA	31,8451304	-0,08029161	0,9019144	-0,08902354	0,929063	0,999787
_CR01790CA	29,80728613	-0,685738672	0,9061123	-0,75679213	0,449174	0,999787
_CR01810CA	6,832760914	-0,771992968	1,4592752	-0,52902492	0,596788	0,999787
_CR01820WA	319,0746196	-0,249255581	0,6730934	-0,3703135	0,711149	0,999787
_CR01910CA	8,021825019	0,174806721	1,3430522	0,130156315	0,896443	0,999787
_CR02060WA	40,01295699	-0,290660878	0,8177284	-0,35544917	0,722253	0,999787
_CR02100CA	74,71834614	-0,827042733	0,887252	-0,93213964	0,351264	0,999787
_CR02120CA	32,34002114	-0,342683692	0,9389412	-0,3649682	0,715135	0,999787
_CR02210WA	172,4652582	0,013383128	0,863261	0,015502992	0,987631	0,999787
_CR02260CA	26,83413755	-0,863508076	0,9782987	-0,88266305	0,377418	0,999787
_CR02270CA	14,6838801	-0,574746669	1,0786238	-0,53285184	0,594136	0,999787
_CR02300CA	205,4586865	0,090186656	0,7088526	0,127229076	0,898759	0,999787
_CR02380CA	11,34275711	-0,567760883	1,152997	-0,49242183	0,622421	0,999787
_CR02410WA	84,01139682	-0,54089444	0,763133	-0,70878136	0,47846	0,999787
_CR02420WA	401,4367823	0,055307412	0,780008	0,070906209	0,943472	0,999787
_CR02430CA	144,9834202	0,271662228	0,7288482	0,372728122	0,709351	0,999787
_CR02440WA	8,60437206	-0,398223272	1,2669856	-0,31430766	0,753287	0,999787
_CR02460WA	65,60577511	-0,088704596	0,787011	-0,11271074	0,91026	0,999787
_CR02500WA	31,46862228	-0,4001211	0,8596956	-0,46542185	0,641629	0,999787
_CR02590CA	32,7157891	0,170181654	0,8643603	0,196887414	0,843916	0,999787
_CR02610CA	217,4236995	-0,025311531	0,7610408	-0,0332591	0,973468	0,999787
_CR02620CA	105,9617855	0,051015328	0,7720763	0,066075501	0,947318	0,999787
_CR02680WA	34,13328689	-0,64123642	0,8475323	-0,75659233	0,449294	0,999787
_CR02690WA	47,0016762	0,071072984	0,8104072	0,087700336	0,930115	0,999787
_CR02770CA	46,355548	0,083972572	0,8021013	0,104690736	0,916621	0,999787
_CR02780WA	7,55800002	-0,834411623	1,352736	-0,61683257	0,537345	0,999787
_CR02880WA	39,07118725	0,687335785	0,8262406	0,831883297	0,405475	0,999787
_CR02890CA	48,69882082	0,233834337	0,9564162	0,244490142	0,806851	0,999787
_CR02910WA	51,75362046	-0,881403727	0,8044221	-1,09569804	0,273211	0,999787
_CR02940CA	19,78197198	-0,409741598	0,9690924	-0,42280962	0,672434	0,999787
_CR02950CA	141,2922384	0,573781847	0,7053903	0,813424653	0,415975	0,999787
_CR02960WA	13,3311871	-0,944519122	1,0904402	-0,86618153	0,386391	0,999787
_CR02970CA	169,2381291	0,542002938	0,7139187	0,759194181	0,447736	0,999787
_CR03000CA	48,24992508	-0,465176502	0,8900069	-0,52266616	0,601207	0,999787
_CR03020CA	68,53236332	0,908214065	0,8220205	1,104855779	0,269222	0,999787
_CR03070WA	137,2783132	0,295869854	0,7085272	0,417584336	0,676251	0,999787
_CR03080CA	29,46659125	-0,486651744	0,912168	-0,53351109	0,59368	0,999787
_CR03110WA	72,32806544	0,770699615	0,8331192	0,925077256	0,354926	0,999787
_CR03120WA	84,66088002	0,004445175	0,7752767	0,005733663	0,995425	0,999787
_CR03130WA	9,645501123	0,766201332	1,2506232	0,612655606	0,540104	0,999787
_CR03160WA	25,86934986	0,630369711	0,8998073	0,7005608	0,483577	0,999787

_CR03180WA	46,84513	0,160924504	0,8359441	0,192506302	0,847346	0,999787
_CR03190CA	10,82524916	-1,136613877	1,2973706	-0,87609033	0,380981	0,999787
_CR03220CA	14,27502511	-0,947898672	1,0570757	-0,89671784	0,369869	0,999787
_CR03240CA	73,42490816	-0,014931813	0,7650327	-0,01951788	0,984428	0,999787
_CR03250CA	146,8268755	-0,603495066	0,7021165	-0,85953691	0,390044	0,999787
_CR03260WA	43,97357623	-0,878483114	0,8172374	-1,07494237	0,282401	0,999787
_CR03310CA	99,69612502	-0,914503623	0,8142174	-1,12316885	0,261366	0,999787
_CR03340CA	79,96955641	-0,000593147	0,7863566	-0,0007543	0,999398	0,999787
_CR03350CA	137,7270789	0,422610949	0,7607244	0,555537541	0,578527	0,999787
_CR03400WA	129,1817581	0,421963214	0,8815016	0,478686862	0,632161	0,999787
_CR03410WA	34,88686305	-0,66824496	0,8646043	-0,77289111	0,439587	0,999787
_CR03430WA	29,1473028	0,311557134	0,8808389	0,35370503	0,72356	0,999787
_CR03440WA	23,86242945	-1,049342259	1,1303215	-0,92835733	0,353222	0,999787
_CR03460WA	109,492551	0,153722926	0,7213401	0,213107415	0,831243	0,999787
_CR03470WA	103,8483517	-0,811850217	0,7407366	-1,09600399	0,273077	0,999787
_CR03520CA	209,670962	0,06191247	0,6885198	0,089921121	0,92835	0,999787
_CR03530WA	884,0256953	0,02796159	0,6582035	0,042481681	0,966115	0,999787
_CR03540WA	40,87168811	0,842066331	0,8660953	0,97225595	0,330923	0,999787
_CR03560WA	36,60132948	-0,672754739	0,8805427	-0,7640228	0,444854	0,999787
_CR03620CA	38,85358836	-0,521211701	0,8237159	-0,63275663	0,526893	0,999787
_CR03640CA	137,2171668	-0,030352283	0,7111828	-0,0426786	0,965958	0,999787
_CR03660CA	31,94835782	1,103886841	1,1222549	0,983632938	0,325296	0,999787
_CR03690WA	32,74768859	-0,695619592	0,9508915	-0,73154467	0,464447	0,999787
_CR03700CA	84,07945726	-0,245480788	0,7472196	-0,32852562	0,742514	0,999787
_CR03740CA	111,4831312	0,481574129	0,7168725	0,671770918	0,50173	0,999787
_CR03780CA	8,272852685	-0,040680014	1,2552726	-0,03240731	0,974147	0,999787
_CR03840CA	26,50274705	0,743791156	0,9098392	0,81749741	0,413644	0,999787
_CR03860CA	52,08278124	0,045318353	0,8713987	0,052006449	0,958524	0,999787
_CR03870WA	52,17720437	-0,397800631	0,7893561	-0,50395588	0,614292	0,999787
_CR03910CA	127,5824151	0,340451297	0,722219	0,471396227	0,637358	0,999787
_CR03930CA	45,25325371	-0,32402526	0,8557055	-0,37866448	0,704937	0,999787
_CR03940WA	97,50193017	1,018793506	0,9753699	1,044520095	0,296245	0,999787
_CR03950WA	28,46784173	0,296280903	0,9740944	0,304160369	0,761006	0,999787
_CR03960CA	21,07960716	-0,269662627	0,9485923	-0,28427664	0,776198	0,999787
_CR03980WA	49,74575045	0,698435519	0,806806	0,865679637	0,386666	0,999787
_CR04020CA	62,77233221	0,156419322	0,7691407	0,203368939	0,838847	0,999787
_CR04080CA	269,6215764	0,32926367	0,7062558	0,466210189	0,641065	0,999787
_CR04120CA	132,8605221	0,746133845	0,7107825	1,049735853	0,29384	0,999787
_CR04140WA	341,5736185	0,801823968	0,8674737	0,924320755	0,355319	0,999787
_CR04150WA	21,72491791	0,583027483	0,9448877	0,617033608	0,537213	0,999787
_CR04180CA	483,9015011	-0,134769993	0,719281	-0,18736766	0,851372	0,999787
_CR04230WA	76,13353807	-0,074568034	0,7583627	-0,09832766	0,921672	0,999787
_CR04240CA	797,9499085	0,850415914	0,7529988	1,129372176	0,258741	0,999787
_CR04270CA	13,37448954	-0,992878923	1,1973517	-0,82922914	0,406975	0,999787
_CR04280CA	26,36892482	-0,04321983	0,8948288	-0,04829955	0,961478	0,999787
_CR04310CA	55,23757968	0,400562216	0,776394	0,515926495	0,605906	0,999787
_CR04330WA	53,4213263	-0,241054226	0,7816961	-0,30837334	0,757798	0,999787
_CR04350CA	54,60354301	-0,129675141	0,7746491	-0,16739856	0,867056	0,999787
_CR04370WA	19,85512214	0,378308499	0,9611004	0,393620171	0,693861	0,999787
_CR04390CA	128,8419757	0,060520302	0,7148059	0,084666764	0,932526	0,999787

_CR04410WA	5,907032692	-0,890774855	1,4317873	-0,62214189	0,533849	0,999787
_CR04450CA	359,8952271	-0,1134457	0,6707327	-0,16913698	0,865689	0,999787
_CR04460CA	39,25135775	-1,02209356	0,8977796	-1,13846826	0,254925	0,999787
_CR04500CA	73,46184334	0,441097698	0,7602604	0,580192881	0,561785	0,999787
_CR04580WA	240,6512167	0,456121082	0,7496278	0,608463368	0,54288	0,999787
_CR04600WA	181,6529308	0,261792958	0,7048457	0,371418842	0,710326	0,999787
_CR04650WA	61,7566967	-0,034609357	0,7713961	-0,04486587	0,964214	0,999787
_CR04710WA	38,74364258	-0,567804126	0,986085	-0,57581661	0,564739	0,999787
_CR04720CA	168,3005383	-0,731384186	0,7031016	-1,04022549	0,298235	0,999787
_CR04730WA	58,41034142	0,324912247	0,8047519	0,403742155	0,686402	0,999787
_CR04760CA	24,92408423	-0,633795831	0,917923	-0,69046733	0,4899	0,999787
_CR04770CA	22,2779229	0,297015517	0,9366536	0,317102844	0,751166	0,999787
_CR04820WA	49,58641838	0,144330442	0,9078506	0,158980394	0,873684	0,999787
_CR04830CA	24,08092291	-0,26088743	0,9334436	-0,27948924	0,779869	0,999787
_CR04860CA	10,36575018	0,13611169	1,255094	0,108447411	0,913641	0,999787
_CR04870CA	70,02577503	0,044575238	0,7584022	0,058775199	0,953131	0,999787
_CR04880WA	30,92109558	-0,479680132	0,8954557	-0,53568271	0,592178	0,999787
_CR04920WA	63,4897847	-0,614380419	0,7773782	-0,79032365	0,429339	0,999787
_CR04940WA	14,31341363	-0,671907012	1,0866834	-0,61830979	0,536371	0,999787
_CR05010WA	40,31870174	0,093133949	0,8400926	0,110861523	0,911726	0,999787
_CR05030WA	229,8609119	-0,707920005	0,7223471	-0,98002749	0,327073	0,999787
_CR05100WA	87,75476749	-0,291139835	0,7576472	-0,38426836	0,70078	0,999787
_CR05120WA	308,5270795	0,146417931	0,6751137	0,216878924	0,828303	0,999787
_CR05130CA	45,06634718	0,587983711	0,8042681	0,731079265	0,464731	0,999787
_CR05140WA	9,509568632	0,597344723	1,2270618	0,486808994	0,626394	0,999787
_CR05150WA	35,22213223	0,254889474	0,9456749	0,269531807	0,78752	0,999787
_CR05160CA	12,82098447	1,246973506	1,1642021	1,071097083	0,284126	0,999787
_CR05200CA	51,08243917	0,087167843	0,8019962	0,108688594	0,913449	0,999787
_CR05210WA	37,61057652	0,047442079	1,0791917	0,043960753	0,964936	0,999787
_CR05310WA	65,71656769	0,390970719	0,7663058	0,510201952	0,60991	0,999787
_CR05330WA	59,21002467	0,20329891	0,788375	0,257870816	0,796507	0,999787
_CR05350WA	6,543344736	-1,30817854	1,6700779	-0,78330392	0,433449	0,999787
_CR05360CA	56,06995631	-0,176528411	0,7737744	-0,22813938	0,819538	0,999787
_CR05370WA	51,24178785	0,728946634	0,8180913	0,891033319	0,372911	0,999787
_CR05440WA	80,88356532	-0,666922025	0,769301	-0,86691944	0,385986	0,999787
_CR05450CA	113,0872992	0,01454762	0,740232	0,019652784	0,98432	0,999787
_CR05500CA	43,80437991	-0,082507516	0,8727272	-0,09453987	0,92468	0,999787
_CR05540CA	53,28885673	-0,603003851	0,7904132	-0,76289694	0,445525	0,999787
_CR05560WA	84,23357926	0,313277747	0,7514232	0,416912534	0,676742	0,999787
_CR05640CA	5,252457445	0,208546788	1,5236068	0,13687704	0,891128	0,999787
_CR05700CA	49,5368446	-0,119875919	0,7881012	-0,15210728	0,879102	0,999787
_CR05710CA	9,662888373	-0,14279442	1,2115773	-0,11785829	0,90618	0,999787
_CR05760CA	27,69336976	-0,726547505	0,8918314	-0,8146691	0,415262	0,999787
_CR05800CA	57,82663138	-0,615129634	0,8667502	-0,70969653	0,477892	0,999787
_CR05840WA	51,13348833	-0,189649244	0,7837322	-0,24198221	0,808794	0,999787
_CR05860WA	72,07023372	-0,328334111	0,7806928	-0,42056763	0,674071	0,999787
_CR05880WA	32,64048187	-0,816192376	0,9124084	-0,89454718	0,371029	0,999787
_CR05890CA	210,6882913	0,394545179	0,6902973	0,571558358	0,567621	0,999787
_CR05900WA	27,80765341	-0,349897324	0,8810099	-0,39715482	0,691253	0,999787
_CR05910WA	118,6815933	-0,357970132	0,7300691	-0,49032364	0,623905	0,999787

_CR05960WA	14,48467805	-0,315916106	1,060115	-0,29800173	0,765702	0,999787
_CR05980WA	178,9406256	-0,263490297	0,7543446	-0,34929698	0,726866	0,999787
_CR06010WA	58,5827652	-0,575446976	0,8891278	-0,64720391	0,5175	0,999787
_CR06020WA	14,10536511	-0,600172443	1,0815627	-0,5549123	0,578955	0,999787
_CR06030CA	18,18122008	-0,492740723	0,994861	-0,49528602	0,620398	0,999787
_CR06060WA	34,76942213	-0,039501567	0,840642	-0,04698976	0,962521	0,999787
_CR06070WA	146,559351	-0,203054183	0,7666906	-0,26484502	0,791129	0,999787
_CR06090WA	93,35323299	-0,248157171	0,7275818	-0,34107116	0,73305	0,999787
_CR06100CA	7,222548112	-1,4820994	1,596883	-0,92812025	0,353345	0,999787
_CR06140WA	244,2810922	-0,562331322	0,7655712	-0,73452514	0,462629	0,999787
_CR06170WA	31,28206919	-0,528701945	0,8601622	-0,61465379	0,538783	0,999787
_CR06230WA	24,97202889	1,24286673	1,132829	1,09713537	0,272582	0,999787
_CR06250WA	11,26500462	-0,752665616	1,1481307	-0,65555746	0,512109	0,999787
_CR06320CA	31,85122018	0,582396528	0,9144411	0,636887951	0,524198	0,999787
_CR06330CA	62,40609817	0,5633486	0,7703842	0,731256684	0,464622	0,999787
_CR06380CA	17,11465714	-1,013873098	0,9995564	-1,01432306	0,310429	0,999787
_CR06430WA	8,144567413	-0,645621144	1,2977321	-0,49749957	0,618837	0,999787
_CR06450WA	31,18128013	0,539933317	0,9631487	0,560591853	0,575076	0,999787
_CR06470WA	89,69549274	-0,658646698	0,7281689	-0,90452463	0,365717	0,999787
_CR06520CA	23,57049766	-0,411303784	0,9593573	-0,42872845	0,668121	0,999787
_CR06550CA	30,12712624	-0,571633118	1,0470217	-0,54596113	0,585093	0,999787
_CR06560CA	60,97376598	-0,252794762	0,7716274	-0,32761247	0,743205	0,999787
_CR06620WA	27,93836631	-0,679495615	0,8762894	-0,77542374	0,438089	0,999787
_CR06630WA	6,361606054	-0,730468662	1,5821865	-0,46168303	0,644309	0,999787
_CR06640CA	55,23584163	-0,76610997	0,7767365	-0,98631898	0,323977	0,999787
_CR06680CA	210,0784606	0,292685051	0,8355478	0,350291195	0,72612	0,999787
_CR06740WA	30,04084449	-0,488123711	0,912413	-0,53498111	0,592663	0,999787
_CR06770CA	72,09727662	-0,009031655	0,7569095	-0,01193228	0,99048	0,999787
_CR06780WA	107,636388	-0,470992401	0,7523018	-0,62606839	0,53127	0,999787
_CR06790CA	26,58026999	0,595807895	0,9019988	0,660541767	0,508906	0,999787
_CR06840WA	65,4774546	0,334644288	0,885794	0,377790214	0,705586	0,999787
_CR06850CA	8,269796103	-0,171393886	1,3173197	-0,13010804	0,896481	0,999787
_CR06930WA	28,14706124	-0,71392398	0,8815213	-0,80987714	0,418011	0,999787
_CR06960WA	22,66125619	0,687862889	1,0800924	0,636855586	0,524219	0,999787
_CR06970CA	111,0918895	-0,667380097	0,8294231	-0,80463167	0,421032	0,999787
_CR07020WA	186,2095352	0,419152537	0,8624325	0,486011982	0,626959	0,999787
_CR07110CA	6,090046738	1,140148854	1,4583501	0,781807365	0,434328	0,999787
_CR07120CA	36,27164867	0,310861862	0,9131148	0,340441155	0,733524	0,999787
_CR07220CA	15,00179251	-0,944230917	1,0546303	-0,89531937	0,370616	0,999787
_CR07340CA	74,01623606	-0,008826879	0,747443	-0,01180943	0,990578	0,999787
_CR07350WA	98,23390166	0,068987121	0,8369908	0,08242279	0,934311	0,999787
_CR07370WA	75,2518172	0,25873844	0,7644974	0,33844253	0,73503	0,999787
_CR07460CA	150,5306902	0,208057334	0,7240971	0,28733346	0,773857	0,999787
_CR07470WA	33,51742344	-0,366002709	0,8777437	-0,4169813	0,676692	0,999787
_CR07480WA	126,7724439	-0,984049485	0,9124983	-1,07841239	0,28085	0,999787
_CR07510WA	44,19023474	-0,378570224	0,9096123	-0,41618856	0,677272	0,999787
_CR07570WA	35,31438049	-0,343100816	0,8894235	-0,38575639	0,699677	0,999787
_CR07640CA	49,73023933	0,054612518	0,8393052	0,065068725	0,948119	0,999787
_CR07660CA	57,73602266	0,739536696	0,7858806	0,941029336	0,34669	0,999787
_CR07670WA	27,7827595	1,114913242	1,1298059	0,986818432	0,323732	0,999787

_CR07690WA	47,65809709	0,045044834	0,8112966	0,055522029	0,955723	0,999787
_CR07740WA	11,14966821	-0,061783561	1,2600788	-0,0490315	0,960894	0,999787
_CR07750CA	109,7920421	0,739345152	0,8372469	0,883067021	0,3772	0,999787
_CR07760WA	75,31130765	0,05046214	0,744635	0,067767616	0,945971	0,999787
_CR07780WA	71,39455637	0,064561997	0,7917801	0,081540309	0,935012	0,999787
_CR07830CA	67,53168923	-0,849409135	1,0111012	-0,84008319	0,400862	0,999787
_CR07850WA	68,80443658	-0,356734673	0,7642226	-0,46679422	0,640647	0,999787
_CR07870WA	38,37454504	-0,33119792	0,8500449	-0,38962401	0,696815	0,999787
_CR07910CA	224,8818164	-0,418863927	0,7782066	-0,53824258	0,59041	0,999787
_CR07920WA	144,636795	0,075938401	0,7099993	0,106955606	0,914824	0,999787
_CR07940WA	26,5256302	-0,504378287	0,92838	-0,54328862	0,586931	0,999787
_CR08000CA	311,2325452	0,313258107	0,6778156	0,462158314	0,643968	0,999787
_CR08040WA	119,013532	-0,862549881	0,8302254	-1,03893464	0,298835	0,999787
_CR08050CA	220,1114926	-0,48858723	0,7363553	-0,66352108	0,506997	0,999787
_CR08080WA	5,479585447	-0,074601977	1,5993338	-0,04664566	0,962796	0,999787
_CR08110WA	15,72878829	0,775420838	1,0730662	0,722621646	0,469912	0,999787
_CR08130WA	167,6474429	0,365781769	0,7547705	0,484626466	0,627941	0,999787
_CR08170CA	83,3492963	0,349223056	0,7798288	0,44782018	0,654283	0,999787
_CR08190WA	18,17181503	-0,699479952	1,0381507	-0,67377498	0,500454	0,999787
_CR08200CA	186,8468057	-0,032411474	0,7555125	-0,04289998	0,965781	0,999787
_CR08280WA	71,2375374	0,666244972	0,8053962	0,827226355	0,408109	0,999787
_CR08290WA	179,886414	-0,283521035	0,7231316	-0,39207394	0,695004	0,999787
_CR08300CA	347,0558774	-0,522814843	0,6823235	-0,76622719	0,443541	0,999787
_CR08330WA	39,44690956	-0,331199706	0,8629501	-0,38379936	0,701127	0,999787
_CR08350WA	168,6987205	0,049393018	0,7133418	0,069241723	0,944797	0,999787
_CR08400CA	167,6060001	0,082465958	0,7510058	0,109807349	0,912562	0,999787
_CR08410WA	250,7735903	-0,501999905	0,786724	-0,63808893	0,523416	0,999787
_CR08420WA	106,6767788	-0,375741516	0,7463343	-0,50344935	0,614648	0,999787
_CR08430WA	234,838326	0,134095953	0,6848141	0,195813663	0,844756	0,999787
_CR08440WA	38,65114599	0,848099771	0,8315378	1,019917272	0,307768	0,999787
_CR08450CA	123,4502692	0,371797784	0,7249438	0,512864308	0,608046	0,999787
_CR08470WA	41,70775504	0,841098602	0,8516422	0,987619698	0,323339	0,999787
_CR08480CA	500,1466835	0,168292986	0,7473008	0,225201131	0,821823	0,999787
_CR08520CA	48,7562	0,725509791	0,8169122	0,888112359	0,37448	0,999787
_CR08560CA	355,5881422	-0,166633083	0,7101854	-0,23463322	0,814493	0,999787
_CR08580CA	13,70614947	0,973508408	1,1005287	0,884582488	0,376382	0,999787
_CR08610WA	349,0757975	-0,422347338	0,7708756	-0,54788002	0,583774	0,999787
_CR08620CA	144,4948747	-0,499588936	0,7904883	-0,6320004	0,527387	0,999787
_CR08640CA	214,2480418	0,315153779	0,7979849	0,394937038	0,692889	0,999787
_CR08680CA	166,5707056	0,491083171	0,7527789	0,652360433	0,514169	0,999787
_CR08720WA	8,349882635	-0,901677043	1,4340993	-0,62874099	0,529519	0,999787
_CR08900CA	197,3838051	-0,555373947	0,844989	-0,6572558	0,511016	0,999787
_CR08930CA	152,1414714	-0,296333997	0,7140346	-0,41501348	0,678132	0,999787
_CR08980CA	95,05000191	-0,511641664	0,7640497	-0,66964446	0,503084	0,999787
_CR09010CA	155,0742315	0,308948179	0,6988958	0,442051823	0,658452	0,999787
_CR09040WA	9,048800214	0,01003411	1,2423084	0,008076988	0,993556	0,999787
_CR09050CA	46,97431477	-0,065057183	0,8664827	-0,07508192	0,94015	0,999787
_CR09070CA	27,07871131	-0,347691596	0,8853436	-0,39271938	0,694527	0,999787
_CR09090CA	15,11478911	-0,529233688	1,0324195	-0,51261497	0,608221	0,999787
_CR09100CA	31,71537551	0,489725069	0,8913021	0,549448997	0,582697	0,999787

_CR09110CA	40,66018953	0,503444379	0,8390532	0,600014842	0,548496	0,999787
_CR09190CA	20,22656525	-0,906603586	0,9483298	-0,95600028	0,339072	0,999787
_CR09230CA	93,916676	0,166257122	0,7421352	0,22402537	0,822738	0,999787
_CR09240CA	132,2911896	0,531085434	0,7110298	0,746924278	0,455109	0,999787
_CR09310WA	77,96516302	0,181168495	0,8131009	0,222811825	0,823682	0,999787
_CR09330CA	512,9138909	-0,651910332	0,6664412	-0,97819627	0,327977	0,999787
_CR09410WA	24,66564466	-0,425282456	0,9110392	-0,46681027	0,640636	0,999787
_CR09440CA	97,86780353	-0,355036128	0,7270614	-0,4883166	0,625326	0,999787
_CR09480WA	67,21874857	-0,265058314	0,775551	-0,34176772	0,732526	0,999787
_CR09490WA	17,95602555	-0,144179065	1,0138992	-0,14220257	0,88692	0,999787
_CR09510CA	75,83879194	-0,178374817	0,929362	-0,19193254	0,847795	0,999787
_CR09520CA	94,14889705	-0,619152055	0,7367592	-0,84037229	0,4007	0,999787
_CR09530CA	43,75183277	-0,795666898	0,9314876	-0,8541895	0,393	0,999787
_CR09560CA	15,68075465	-0,733407464	1,0575646	-0,69348713	0,488004	0,999787
_CR09580CA	37,77438814	-0,821236671	0,8268195	-0,99324784	0,320589	0,999787
_CR09590WA	22,58441473	-0,436347925	0,9697659	-0,44995182	0,652745	0,999787
_CR09620CA	31,82161946	-0,152031352	1,0362771	-0,14670917	0,883362	0,999787
_CR09640CA	187,1378367	0,534209586	0,6960922	0,767440883	0,442819	0,999787
_CR09660WA	21,15855296	0,299476102	1,096043	0,273233907	0,784673	0,999787
_CR09670CA	68,54416881	-0,556595239	0,8173206	-0,68099989	0,495872	0,999787
_CR09810WA	11,88786195	0,406461606	1,1218709	0,362306948	0,717123	0,999787
_CR09830WA	20,3384974	0,743651467	0,9629026	0,772301881	0,439936	0,999787
_CR09840CA	8,491451588	-0,006914877	1,2662261	-0,00546101	0,995643	0,999787
_CR09920WA	383,4105133	-0,858245364	0,9573521	-0,89647825	0,369997	0,999787
_CR09990WA	178,4964683	-0,50792738	0,6991001	-0,72654458	0,467505	0,999787
_CR10000CA	12,14341006	-1,21639452	1,159745	-1,04884654	0,294249	0,999787
_CR10020CA	36,24502856	-0,628497967	0,8750187	-0,71826805	0,472592	0,999787
_CR10060WA	115,261206	0,346330067	0,7162992	0,483499195	0,628741	0,999787
_CR10130WA	44,69718637	-0,138271005	0,8012122	-0,17257725	0,862984	0,999787
_CR10140WA	523,9845846	-0,107317168	0,8451935	-0,12697348	0,898961	0,999787
_CR10170CA	56,25389905	0,653752307	0,8947317	0,730668577	0,464982	0,999787
_CR10180WA	5,462351611	0,922522356	1,6170046	0,570513147	0,56833	0,999787
_CR10190CA	9,67946987	-0,305538519	1,2219247	-0,25004693	0,802551	0,999787
_CR10230WA	175,3487389	-0,334774048	0,7070354	-0,47348979	0,635864	0,999787
_CR10280WA	61,91396427	0,380384442	0,7676439	0,495521986	0,620232	0,999787
_CR10290CA	126,8545854	0,246855793	0,7654185	0,322510868	0,747066	0,999787
_CR10380CA	50,47117112	0,530265598	0,817309	0,648794522	0,516471	0,999787
_CR10390WA	20,71259074	0,379998226	0,9490379	0,400403643	0,688859	0,999787
_CR10400WA	127,0112056	0,597147291	0,7105041	0,840455788	0,400653	0,999787
_CR10420WA	63,58076125	0,599368088	0,822119	0,729052724	0,465969	0,999787
_CR10430CA	12,97770153	-0,419683647	1,1349416	-0,36978435	0,711543	0,999787
_CR10440WA	39,94887591	0,182640016	0,817132	0,223513488	0,823136	0,999787
_CR10460WA	31,23474063	-0,558104477	0,8848232	-0,63075254	0,528202	0,999787
_CR10510WA	74,86181332	-0,18181916	0,7433285	-0,24460137	0,806765	0,999787
_CR10530WA	48,50429738	0,186898297	0,8036101	0,232573365	0,816093	0,999787
_CR10600CA	311,1287956	-0,599304832	0,7622402	-0,78624143	0,431726	0,999787
_CR10610CA	25,6293679	-0,863588082	0,9527537	-0,90641276	0,364717	0,999787
_CR10630WA	19,4086071	0,160911199	1,012703	0,158892777	0,873753	0,999787
_CR10640WA	22,46946604	0,730288615	0,9390816	0,77766257	0,436768	0,999787
_CR10730CA	115,0940371	0,608978073	0,7238468	0,841307932	0,400175	0,999787

_CR10740WA	49,54238632	0,524122939	0,7933327	0,66065966	0,508831	0,999787
_CR10760CA	54,58351621	-0,107990967	0,8594053	-0,12565778	0,900003	0,999787
_CR10800CA	288,1448864	0,043080157	0,6781671	0,063524399	0,949349	0,999787
_CR10810CA	199,2342464	0,066392302	0,8588794	0,077301075	0,938384	0,999787
_CR10820WA	140,2492357	-0,091215886	0,7846023	-0,11625748	0,907448	0,999787
_CR10830CA	251,2273008	0,384971945	0,715508	0,538040016	0,590549	0,999787
CAC2	34,44854213	-0,557792547	0,8401787	-0,66389753	0,506756	0,999787
CAG1	5,929486118	-0,019326948	1,4915209	-0,01295788	0,989661	0,999787
CAK1	12,82752143	-1,144506919	1,1134803	-1,02786456	0,304014	0,999787
CAM1	1557,012163	0,431621721	0,6865299	0,628700535	0,529545	0,999787
CAM1-1	789,3829712	-0,356684163	0,6615409	-0,53917175	0,589768	0,999787
CAN1	128,7937701	-0,49185022	1,0080492	-0,48792283	0,625605	0,999787
CAN2	158,7112342	0,3733362	0,8862611	0,421248558	0,673574	0,999787
CAP4	59,72159626	-0,124044466	0,766182	-0,16189947	0,871385	0,999787
CAS1	52,32786085	-0,229369989	0,946121	-0,24243197	0,808445	0,999787
CAS4	199,7176995	0,407067575	0,7626924	0,533724473	0,593532	0,999787
CAS5	215,8705566	-0,071287831	0,6910294	-0,1031618	0,917835	0,999787
CAT8	21,34102127	-0,658705153	0,9461217	-0,69621608	0,486294	0,999787
CBK1	289,8178628	0,06043231	0,7256495	0,083280303	0,933629	0,999787
CBP1	237,8700174	-0,683535427	0,6883861	-0,99295349	0,320733	0,999787
CBR1	124,3386407	0,846940957	0,7615948	1,112062435	0,266111	0,999787
CCE1	7,630433405	-0,561901088	1,3175067	-0,42648823	0,669752	0,999787
CCH1	157,3569902	0,413832818	0,7182716	0,576150856	0,564513	0,999787
CCN1	37,4487992	0,084722844	0,9097274	0,093129929	0,9258	0,999787
CCP1	250,6407268	-0,26697266	0,6785787	-0,39342916	0,694003	0,999787
CCR4	256,7704198	-0,099417735	0,6825862	-0,14564862	0,884199	0,999787
CCS1	336,7071515	0,550936639	0,7728664	0,712848449	0,47594	0,999787
CCT2	476,3759503	0,518829906	0,7280997	0,712580847	0,476105	0,999787
CCT3	596,5527694	0,36150197	0,677608	0,533497191	0,593689	0,999787
CCT5	722,8734859	0,700558958	0,7193599	0,973864329	0,330124	0,999787
CCT6	677,8334075	0,367614334	0,6701341	0,548568287	0,583302	0,999787
CCT7	373,8945565	0,381264307	0,7190088	0,530263732	0,595929	0,999787
CCW14	30,36896078	0,023283525	0,8745918	0,026622163	0,978761	0,999787
CDC10	282,9556034	0,135496636	0,6848155	0,197858608	0,843156	0,999787
CDC11	234,2667558	0,595710513	0,7037431	0,846488566	0,39728	0,999787
CDC12	353,6856876	0,06126804	0,6765705	0,090556772	0,927845	0,999787
CDC13	40,18533644	0,955309829	0,8628277	1,10718492	0,268214	0,999787
CDC14	99,81953709	-0,321521904	0,7556087	-0,42551377	0,670462	0,999787
CDC15	48,35999497	0,046652932	0,8904352	0,052393402	0,958215	0,999787
CDC19	6943,809558	-0,732420478	0,6950614	-1,05374937	0,291998	0,999787
CDC20	114,1837518	0,435406597	0,8445957	0,515520758	0,606189	0,999787
CDC21	48,42110152	-0,291140986	0,8061076	-0,3611689	0,717973	0,999787
CDC23	68,84308278	-0,293380931	0,7562457	-0,38794393	0,698058	0,999787
CDC24	193,2277401	-0,269202464	0,6977757	-0,38580083	0,699644	0,999787
CDC27	29,92285785	-0,862475333	0,9007333	-0,95752576	0,338302	0,999787
CDC28	86,05644097	0,295118468	0,8236756	0,358294515	0,720123	0,999787
CDC3	252,7926379	-0,283885272	0,6913292	-0,41063689	0,681339	0,999787
CDC37	205,849086	0,133267391	0,6849782	0,194557124	0,84574	0,999787
CDC39	430,3864403	-0,401550421	0,8376104	-0,47940001	0,631654	0,999787
CDC4	208,3524299	0,382066537	0,7247479	0,527171622	0,598074	0,999787

CDC42	198,4941597	0,272257494	0,6896461	0,394778571	0,693006	0,999787
CDC45	55,87679113	-0,209600073	0,8613904	-0,24332761	0,807752	0,999787
CDC47	349,793773	0,567044731	0,8144229	0,69625339	0,48627	0,999787
CDC48	2203,350734	-0,202429912	0,6636592	-0,30502087	0,76035	0,999787
CDC5	313,4997928	0,187972253	0,6910116	0,272024755	0,785603	0,999787
CDC50	97,04185626	0,25844281	0,72868	0,354672578	0,722835	0,999787
CDC53	105,5024807	-0,1583243	0,8317032	-0,19036154	0,849026	0,999787
CDC54	112,4208442	0,579167727	0,8690969	0,666401809	0,505154	0,999787
CDC55	283,3548764	-0,417757606	0,6792952	-0,61498684	0,538563	0,999787
CDC6	13,88683064	0,717209186	1,1227209	0,638813428	0,522944	0,999787
CDC60	1283,875977	0,278917068	0,8447148	0,330190808	0,741256	0,999787
CDC68	444,6721825	-0,310372504	0,6683645	-0,46437608	0,642378	0,999787
CDC7	86,6777489	-0,70850504	0,7355973	-0,96316965	0,335462	0,999787
CDC73	210,0387176	0,044805249	0,6998033	0,064025489	0,94895	0,999787
CDG1	35,80348596	-0,168895803	0,83592	-0,2020478	0,839879	0,999787
CDH1	91,49880795	-0,288899732	0,7522043	-0,38407083	0,700926	0,999787
CDL1	144,0690367	0,248656879	0,7501025	0,331497192	0,740269	0,999787
CDR3	25,71216318	0,59887887	0,9053366	0,661498592	0,508293	0,999787
CDS1	106,1152553	0,529693222	0,7329173	0,722718929	0,469853	0,999787
CEF3	11646,29021	-0,118195454	0,6519918	-0,18128364	0,856145	0,999787
CEK1	21,9623503	-0,040234263	0,934315	-0,04306285	0,965651	0,999787
CEM1	47,17817074	0,651051645	0,8008678	0,812932744	0,416257	0,999787
CET1	395,3753449	-0,109532567	0,698905	-0,15672025	0,875465	0,999787
CEX1	87,27997274	0,202954875	0,7672575	0,26451989	0,791379	0,999787
CFL1	52,09245091	-0,338922235	0,8596077	-0,39427548	0,693378	0,999787
CFL2	51,29732222	0,681429019	0,83916	0,812037057	0,41677	0,999787
CGR1	33,07844326	0,686703271	0,8592633	0,799176807	0,424188	0,999787
CGT1	106,7356146	0,665104986	0,780887	0,851730127	0,394364	0,999787
CHA1	70,32487818	-0,366875794	0,8719421	-0,42075706	0,673932	0,999787
CHC1	842,3568484	-0,413699944	0,7653558	-0,54053281	0,58883	0,999787
CHK1	77,14442577	-0,28198259	0,751999	-0,37497736	0,707677	0,999787
CHL4	24,99756344	-0,198380024	0,9460635	-0,20968997	0,83391	0,999787
CHO2	44,08758934	0,059854413	0,8481144	0,070573511	0,943737	0,999787
CHS1	286,7865653	0,75000897	0,7035283	1,066067989	0,286393	0,999787
CHS2	75,35997024	0,567856491	0,7600278	0,747152228	0,454972	0,999787
CHS4	259,0541966	0,714706662	0,7982662	0,895323668	0,370614	0,999787
CHS6	68,73987879	-0,021663254	0,8125097	-0,02666215	0,978729	0,999787
CHS7	45,53405197	0,238848312	0,8026987	0,297556609	0,766042	0,999787
CHS8	282,2508469	0,688744663	0,7733862	0,890557206	0,373167	0,999787
CHT1	55,08611152	0,545995998	0,8193077	0,666411443	0,505148	0,999787
CHT2	1753,563538	-0,08137629	0,6640517	-0,12254512	0,902467	0,999787
CHT3	300,376511	-0,572180899	0,6743633	-0,84847577	0,396173	0,999787
CHT4	17,99264454	0,248582212	1,003433	0,24773174	0,804342	0,999787
CIC1	159,6653329	0,220708435	0,7165716	0,308006114	0,758078	0,999787
CIS2	98,6464003	0,013179943	0,8148332	0,016175019	0,987095	0,999787
CIT1	2025,798879	-0,090556414	0,6561633	-0,13800896	0,890233	0,999787
CKA1	282,5452114	0,320985975	0,7130323	0,450170333	0,652588	0,999787
CKA2	41,23314995	0,487692899	0,9612336	0,507361464	0,611901	0,999787
CKB1	121,66835	-0,498925413	0,7348756	-0,67892498	0,497185	0,999787
CKB2	81,28078077	0,270043916	0,7667289	0,352202596	0,724686	0,999787

CKS1	53,03345511	0,195923896	0,7979255	0,245541582	0,806037	0,999787
CLA4	95,32944147	0,645404678	0,8995799	0,71745117	0,473096	0,999787
CLB2	254,9633666	0,29210099	0,7192166	0,406137735	0,684641	0,999787
CLB4	125,2820486	-0,278380771	0,7127428	-0,39057676	0,69611	0,999787
CLC1	395,4748749	-0,485366307	0,7131332	-0,68061099	0,496118	0,999787
CLG1	304,5040072	0,77805595	0,7358999	1,057285078	0,290382	0,999787
CLN3	76,30076436	0,161115393	0,8727759	0,184601108	0,853542	0,999787
CMD1	450,8568425	-0,267855798	0,7112882	-0,37657843	0,706487	0,999787
CMK2	138,3039814	-0,642428713	0,7637411	-0,8411603	0,400258	0,999787
CMP1	166,0882093	0,150278244	0,7371208	0,203871947	0,838454	0,999787
CNB1	87,96858017	0,4458838	0,7319019	0,609212483	0,542384	0,999787
CNH1	639,9719718	0,120719314	0,7626951	0,158279914	0,874236	0,999787
CNT	234,0713067	0,190926466	0,747777	0,255325389	0,798472	0,999787
COG4	101,0359872	-0,610909464	0,7461925	-0,81870225	0,412956	0,999787
COI1	56,60563155	0,697305671	0,8025719	0,868838888	0,384935	0,999787
COQ3	122,7395364	0,279743123	0,7207434	0,388131353	0,697919	0,999787
COQ4	60,39693846	-0,367255851	0,7669152	-0,47887411	0,632028	0,999787
COQ5	148,0446022	0,370408488	0,7882689	0,469901212	0,638426	0,999787
COQ6	231,4909485	0,07938111	0,7008826	0,113258774	0,909825	0,999787
COX13	382,1698154	-0,3910702	0,670375	-0,58336038	0,559651	0,999787
COX15	219,0532242	-0,231722695	0,701328	-0,33040558	0,741094	0,999787
COX17	22,55365482	0,850926263	1,1610356	0,732902806	0,463618	0,999787
COX19	36,86038616	-0,092941855	0,8475742	-0,10965631	0,912682	0,999787
COX4	873,198596	-0,294519723	0,6715509	-0,43856647	0,660976	0,999787
COX5	471,0128091	0,447755076	0,7351004	0,609107355	0,542453	0,999787
COX6	512,3694506	0,304611219	0,7565952	0,402607919	0,687237	0,999787
COX7	89,6545008	-0,477768551	0,7931455	-0,60237188	0,546927	0,999787
COX8	440,667639	0,071434284	0,6764137	0,105607384	0,915894	0,999787
COX9	216,0413335	-0,043372732	0,7171623	-0,06047827	0,951775	0,999787
CPA1	11,89366352	-0,270566059	1,1290242	-0,23964593	0,810605	0,999787
CPA2	112,6630149	0,444522271	0,8528446	0,521223081	0,602211	0,999787
CPH1	39,90573858	-0,133574772	0,8274958	-0,16142049	0,871762	0,999787
CPH2	590,4814123	-0,376682075	0,80691	-0,46682045	0,640628	0,999787
CPP1	44,52026671	0,465456789	0,9637327	0,482972931	0,629115	0,999787
CPR3	151,0741823	-0,663205791	0,7083362	-0,93628672	0,349126	0,999787
CPR6	803,0009442	0,334042557	0,6858204	0,487070005	0,626209	0,999787
CPY1	339,9870665	-0,045795314	0,7177324	-0,06380556	0,949125	0,999787
CRD2	75,80000778	0,101171782	0,7863112	0,128666339	0,897622	0,999787
CRH11	341,0795641	-0,1396757	0,6789251	-0,20573063	0,837001	0,999787
CRK1	132,1910387	-0,207563364	0,7368459	-0,28169167	0,77818	0,999787
CRL1	126,6564331	0,594866694	0,7732119	0,769344972	0,441689	0,999787
CRM1	290,2824963	-0,384251344	0,7859765	-0,48888401	0,624924	0,999787
CRN1	305,4003766	-0,481557132	0,6729395	-0,71560237	0,474237	0,999787
CRZ1	119,5643401	0,649940731	0,7331434	0,886512443	0,375341	0,999787
CRZ2	12,72819623	0,046999559	1,1359227	0,041375668	0,966996	0,999787
CSC25	172,5955701	0,077630753	0,7004614	0,110828025	0,911753	0,999787
CSE4	159,758141	-0,560374586	0,7399141	-0,75735086	0,44884	0,999787
CSH1	62,79847118	0,474767832	0,7723418	0,614712069	0,538745	0,999787
CSI2	46,20237574	0,317490902	1,0160881	0,312463953	0,754688	0,999787
CSM3	61,26825427	-0,078736135	0,8319228	-0,09464356	0,924598	0,999787

CSP1	5,919099676	1,061208744	1,4626923	0,725517443	0,468135	0,999787
CSP2	12,13551568	-0,165267616	1,1523748	-0,14341481	0,885963	0,999787
CSR1	28,5900303	0,627607719	0,8853408	0,708888279	0,478394	0,999787
CST20	242,7480794	0,126045495	0,7368942	0,171049643	0,864185	0,999787
CTA1	96,68775459	-0,152737347	0,7378491	-0,20700351	0,836007	0,999787
CTA3	749,4541892	-0,164611997	0,6837299	-0,24075587	0,809744	0,999787
CTA4	210,3227206	-0,757379069	0,6912559	-1,0956565	0,273229	0,999787
CTA6	67,39541531	-0,478628843	0,849642	-0,56333005	0,57321	0,999787
CTA7	132,3226683	0,127926293	0,8038254	0,159146861	0,873553	0,999787
CTA8	111,7535206	0,790549802	0,7516374	1,05177022	0,292905	0,999787
CTA9	91,12786693	-0,704003929	0,7328703	-0,96061193	0,336747	0,999787
CTF1	88,50072662	0,105656012	0,8300865	0,127283127	0,898716	0,999787
CTF18	68,65401424	-0,134294873	0,7532111	-0,17829646	0,85849	0,999787
CTF8	12,38018318	0,337052443	1,1086101	0,304031548	0,761104	0,999787
CTM1	234,4966987	-0,135143654	0,6937344	-0,19480604	0,845545	0,999787
CTP1	39,46162186	0,082172687	1,1698598	0,070241485	0,944001	0,999787
CUE5	406,5283612	-0,86581457	0,7716943	-1,12196582	0,261877	0,999787
CUP2	83,8607107	-0,252192067	0,7736222	-0,32598865	0,744433	0,999787
CUP5	258,3867623	-0,469592315	0,6812261	-0,68933397	0,490613	0,999787
CUP9	673,4635575	-0,304659464	0,9760949	-0,31212074	0,754949	0,999787
CWH41	101,0277383	0,141572514	0,7248779	0,195305322	0,845154	0,999787
CWH43	348,9580648	-0,314845231	0,7063121	-0,44575938	0,655771	0,999787
CWH8	13,59098394	0,076961861	1,1197283	0,068732622	0,945202	0,999787
CWT1	213,5758618	-0,588401173	0,7258746	-0,81060998	0,41759	0,999787
CYB5	100,6215873	0,37981732	0,7801274	0,486865742	0,626353	0,999787
CYC1	365,3798323	0,255037188	0,9061208	0,281460466	0,778357	0,999787
CYK3	63,12497811	0,340172483	0,8591221	0,395953602	0,692139	0,999787
CYM1	161,2413169	-0,066676155	0,8218361	-0,08113072	0,935338	0,999787
CYP1	1661,725104	-0,441731909	0,7001062	-0,63094987	0,528073	0,999787
CYR1	275,8449321	-0,007371266	0,7063295	-0,01043602	0,991673	0,999787
CYS4	595,429639	0,421312393	0,7029796	0,599323812	0,548957	0,999787
CYT1	1222,21288	-0,292780654	0,6607835	-0,44308108	0,657707	0,999787
CYT2	44,16814548	0,431956587	0,8550554	0,505179628	0,613433	0,999787
CZF1	73,19813952	-0,680599753	0,7620543	-0,89311185	0,371797	0,999787
DAD2	14,10527729	-0,529070547	1,095864	-0,48278853	0,629246	0,999787
DAD3	34,42807967	0,746918876	0,8548477	0,873744942	0,382257	0,999787
DAD4	59,83533418	-0,510137048	0,7979006	-0,63934913	0,522596	0,999787
DAL8	7,205073043	0,216873043	1,3629503	0,159120281	0,873574	0,999787
DAL81	38,7730721	0,598099994	0,9259794	0,645910725	0,518337	0,999787
DAM1	53,63146879	-0,779593055	0,865554	-0,90068684	0,367755	0,999787
DAO1	128,5533652	-0,657352158	0,8008571	-0,82081082	0,411754	0,999787
DAO2	54,95840346	-0,183065448	0,7752038	-0,23615137	0,813315	0,999787
DAP2	85,0382478	0,115251761	0,7865199	0,146533811	0,8835	0,999787
DBF2	70,7915538	0,401370549	0,8881463	0,451919421	0,651327	0,999787
DBP5	334,8523981	0,23052951	0,792436	0,290912465	0,771118	0,999787
DBR1	30,26844803	0,015555709	0,8649372	0,017984784	0,985651	0,999787
DCC1	15,72009524	0,416295728	1,0598514	0,392786868	0,694477	0,999787
DCG1	14,99341566	0,399063598	1,098088	0,36341678	0,716294	0,999787
DCK1	230,008497	-0,798210968	0,7604597	-1,04964268	0,293882	0,999787
DCK2	43,12815699	-0,239613029	0,8303943	-0,28855332	0,772923	0,999787

DCP2	597,1145818	-0,525904546	0,6714069	-0,7832874	0,433458	0,999787
DCR1	150,2219642	0,792433305	0,7071466	1,120606828	0,262455	0,999787
DCW1	266,7598684	0,029126871	0,7205899	0,040420869	0,967758	0,999787
DDC1	13,77384573	-0,367827055	1,0628523	-0,34607542	0,729286	0,999787
DED1	493,6867927	0,02337639	0,8587429	0,027221639	0,978283	0,999787
DED81	825,3214848	0,436665123	0,7619798	0,573066561	0,5666	0,999787
DEF1	864,3408158	-0,527011527	0,8894748	-0,59249745	0,553518	0,999787
DEM1	73,42398423	-0,304908093	0,825578	-0,36932683	0,711884	0,999787
DFG10	18,93704707	-0,000777608	1,052031	-0,00073915	0,99941	0,999787
DFG16	76,12108353	-0,518048036	0,8542509	-0,60643543	0,544226	0,999787
DFG5	151,8334398	-0,148347901	0,7781292	-0,19064687	0,848802	0,999787
DFI1	265,5325689	0,307725174	0,6947791	0,442910818	0,65783	0,999787
DFR1	17,81742419	0,751849166	0,999824	0,751981531	0,452062	0,999787
DHH1	112,193702	-0,51591219	0,7201397	-0,71640572	0,473741	0,999787
DIP5	647,6447955	0,598111379	0,697926	0,856983979	0,391454	0,999787
DIT1	7,300530139	-0,199474176	1,322848	-0,15079146	0,88014	0,999787
DIT2	5,522235544	0,711229832	1,4881929	0,47791507	0,632711	0,999787
DJP1	344,83433	-0,429713453	0,7861406	-0,54661145	0,584646	0,999787
DLD1	221,5033703	-0,636399684	0,7208407	-0,88285764	0,377313	0,999787
DLD2	56,99560032	0,49041101	0,801891	0,611568167	0,540824	0,999787
DNA2	87,64720007	0,51986773	0,759189	0,684767211	0,493491	0,999787
DOA1	246,1014275	-0,110087589	0,6808627	-0,16168839	0,871551	0,999787
DOA4	89,68924859	0,209101823	0,7469795	0,279929787	0,779531	0,999787
DOT1	229,7112172	-0,187292812	0,7902876	-0,23699324	0,812662	0,999787
DOT4	492,63718	0,138807642	0,7178647	0,193361841	0,846676	0,999787
DOT6	141,4293138	0,15256044	0,7083817	0,215364741	0,829483	0,999787
DPB2	45,04358332	-0,197255153	0,8180739	-0,24112144	0,809461	0,999787
DPB4	168,8307522	0,67510991	0,7071396	0,954705343	0,339727	0,999787
DPM1	181,8306513	-0,007349226	0,7087469	-0,01036932	0,991727	0,999787
DPM2	63,433003	0,321977164	0,8021694	0,40138299	0,688138	0,999787
DPM3	41,43799556	-0,123371498	0,8119283	-0,15194877	0,879227	0,999787
DPP1	22,91312469	-1,071357871	0,9470441	-1,13126497	0,257944	0,999787
DPP2	80,03413758	-0,567955763	0,8585541	-0,66152591	0,508275	0,999787
DPP3	6,457139276	0,293576134	1,3968024	0,210177289	0,833529	0,999787
DPS1-1	988,8737673	0,266823292	0,6741474	0,395793672	0,692257	0,999787
DRG1	283,466736	0,733210327	0,7895741	0,928614917	0,353089	0,999787
DSE1	81,80178772	-0,462416185	0,8087998	-0,5717313	0,567504	0,999787
DSL1	20,60250424	-0,994214855	0,9515257	-1,04486394	0,296086	0,999787
DUN1	60,04406306	0,595179008	0,8859528	0,67179539	0,501714	0,999787
DUO1	19,31018241	-0,929393085	1,0061327	-0,92372811	0,355628	0,999787
DUR1%2C2	86,93878979	0,385761704	0,803556	0,48006821	0,631179	0,999787
DUR3	6,593083461	-0,997441053	1,5064888	-0,66209658	0,507909	0,999787
DUR32	14,52862689	-0,343712432	1,0758028	-0,31949389	0,749352	0,999787
DUR4	41,07524666	0,11733396	0,8392469	0,139808624	0,888811	0,999787
DUR7	31,61229458	0,050803886	0,8859449	0,057344293	0,954271	0,999787
DUS4	57,92846514	0,644626544	0,7815887	0,824764458	0,409505	0,999787
DUT1	63,84885764	0,68540392	0,831117	0,824677984	0,409554	0,999787
DYN1	232,1844868	0,478236414	0,798672	0,598789485	0,549313	0,999787
EAF3	22,66675209	-0,303605458	0,9368541	-0,32406909	0,745886	0,999787
EAF6	73,58068945	-0,433303141	0,7482012	-0,57912651	0,562504	0,999787

EAF7	561,2511018	-0,86964641	0,8500271	-1,02308086	0,30627	0,999787
EAP1	51,4243636	-0,822443127	0,7937187	-1,03618963	0,300114	0,999787
EBP7	52,37130284	0,652315717	0,8049804	0,810349807	0,417739	0,999787
ECM14	158,2290873	0,73507412	0,6976952	1,053574789	0,292078	0,999787
ECM22	103,8909408	0,198168605	0,8062686	0,245784848	0,805849	0,999787
ECM25	36,21234095	-0,03994283	0,8316507	-0,04802837	0,961694	0,999787
ECM29	260,9124022	-0,33678712	0,8055814	-0,41806718	0,675898	0,999787
ECM3	47,04544825	0,562087247	0,8038565	0,699238262	0,484403	0,999787
ECM33	1478,929249	0,469773337	0,7248498	0,64809749	0,516922	0,999787
ECM331	10,2949329	-0,066945229	1,1779264	-0,05683312	0,954678	0,999787
ECM39	91,31396359	0,663675919	0,7404367	0,896330351	0,370076	0,999787
ECM4	184,8846831	-0,751348707	0,811149	-0,92627706	0,354302	0,999787
ECM42	46,19555073	0,299891854	0,8033382	0,373307108	0,70892	0,999787
ECM7	53,38012837	-0,513092207	0,8049398	-0,63742931	0,523845	0,999787
EDC3	68,96217376	0,34921537	0,7878171	0,443269612	0,657571	0,999787
EFB1	2582,779121	0,163719887	0,6551914	0,249881005	0,802679	0,999787
EFG1	580,3796968	-0,187750437	0,7464175	-0,25153541	0,8014	0,999787
EFH1	12,92788668	-1,417123345	1,3537331	-1,04682625	0,29518	0,999787
EFT2	13765,41336	0,152601279	0,6611722	0,230804137	0,817467	0,999787
EGD1	1428,13511	-0,078827081	0,664708	-0,11858904	0,905601	0,999787
EGD2	1858,315484	-0,58401645	0,7164896	-0,81510802	0,41501	0,999787
EIF4E	445,8280864	0,410335536	0,6678894	0,614376489	0,538967	0,999787
ELA1	7,452397716	-0,086032874	1,3436414	-0,06402964	0,948947	0,999787
ELC1	31,56198673	-0,391904585	0,9446258	-0,41487814	0,678231	0,999787
ELF1	703,0134436	0,70207014	0,7972883	0,880572465	0,378549	0,999787
EMC9	290,5071828	0,160728668	0,7082204	0,226947255	0,820465	0,999787
EMP24	156,3023023	-0,092268816	0,7128306	-0,12944003	0,897009	0,999787
EMP46	194,2214487	0,589365298	0,691923	0,851778715	0,394337	0,999787
EMP70	123,6175131	0,389170838	0,813626	0,478316628	0,632425	0,999787
ENA21	2013,149486	0,276941223	0,7314228	0,378633543	0,70496	0,999787
END3	29,92574932	-0,391376111	0,8948832	-0,43734882	0,661858	0,999787
ENG1	299,9107455	0,368953814	0,8325914	0,443139138	0,657665	0,999787
ENO1	11378,28977	-0,720191983	0,6753058	-1,06646794	0,286212	0,999787
ENP1	102,6751607	1,052518887	1,0721358	0,981702981	0,326246	0,999787
ENT3	725,6356094	0,23175043	0,6659464	0,348001644	0,727839	0,999787
ERB1	277,8189402	0,535273244	0,8502262	0,629565688	0,528979	0,999787
ERD1	7,196785177	-0,128680126	1,3252893	-0,09709588	0,92265	0,999787
ERF1	513,6041308	0,967016387	0,8620584	1,121752698	0,261968	0,999787
ERG10	554,2797826	-0,453006146	0,6642956	-0,68193454	0,49528	0,999787
ERG11	342,017162	-0,119057315	0,7052052	-0,16882648	0,865933	0,999787
ERG12	226,3885386	0,587112784	0,7003593	0,838302316	0,401861	0,999787
ERG13	668,0353361	0,113973615	0,739513	0,15411983	0,877515	0,999787
ERG20	87,02330485	-0,218709904	0,9010884	-0,24271747	0,808224	0,999787
ERG24	115,5208682	0,164748178	0,7195203	0,228969451	0,818893	0,999787
ERG25	60,60184025	0,320465133	0,840262	0,381387147	0,702916	0,999787
ERG26	123,0405323	0,337233828	0,7462515	0,451903691	0,651338	0,999787
ERG27	70,30639633	0,772092679	0,7642674	1,010238983	0,312381	0,999787
ERG28	41,25007928	-0,550944219	0,811955	-0,67854033	0,497429	0,999787
ERG3	89,4767228	-0,428529761	0,7532576	-0,56890202	0,569423	0,999787
ERG4	115,2574608	0,179998204	0,7185366	0,250506645	0,802196	0,999787

ERG6	323,5097311	0,452281946	0,9322257	0,485163585	0,62756	0,999787
ERG7	187,3830824	-0,131537541	0,6918755	-0,19011736	0,849217	0,999787
ERG8	236,2911655	0,470906843	0,6811964	0,691293818	0,489381	0,999787
ERG9	81,6123842	-0,178971522	0,9522329	-0,18794931	0,850916	0,999787
ERO1	227,4528549	0,238880707	0,7861567	0,303858904	0,761235	0,999787
ERP5	184,7768309	0,145717171	0,6934591	0,210130877	0,833566	0,999787
ERV1	69,12032437	0,420846699	0,7590767	0,554419167	0,579292	0,999787
ERV25	151,8437827	0,361767941	0,7025951	0,514902471	0,606621	0,999787
ERV29	123,7770946	-0,438493678	0,7092252	-0,61827145	0,536396	0,999787
ERV46	124,098202	0,081560675	0,7095283	0,114950561	0,908484	0,999787
ESA1	141,5117225	-0,420199229	0,7176831	-0,58549411	0,558216	0,999787
ESC4	25,34943867	-0,614870105	0,9479877	-0,64860555	0,516593	0,999787
ESP1	36,97113653	0,262001771	1,0139319	0,25840174	0,796097	0,999787
ESS1	228,3648998	0,654072581	0,7541779	0,867265638	0,385796	0,999787
EST1	250,6225122	0,165140386	0,7299673	0,226229848	0,821023	0,999787
EST3	5,239820175	-0,131091931	1,5073945	-0,08696591	0,930699	0,999787
EXG2	48,15421506	-0,10288078	0,8967851	-0,11472177	0,908666	0,999787
EXM2	31,28245691	0,071258874	0,860645	0,082797058	0,934013	0,999787
EXO1	138,003196	0,260267515	0,8871529	0,293373912	0,769236	0,999787
EXO70	51,68326687	-0,330300027	0,8759253	-0,37708697	0,706109	0,999787
EXO84	51,00818452	-0,834493703	0,845586	-0,9868821	0,3237	0,999787
FAA2	18,40650243	-0,938903896	1,0311479	-0,91054245	0,362537	0,999787
FAA2-1	70,70687792	-0,927315275	0,8847065	-1,04816152	0,294564	0,999787
FAA4	627,5252447	0,221629943	0,7917888	0,279910425	0,779546	0,999787
FAB1	185,022291	-0,661821977	0,6937019	-0,95404373	0,340062	0,999787
FAD2	282,5341619	0,627965641	0,6865696	0,914642416	0,360379	0,999787
FAS1	5471,958969	0,341706557	0,7523589	0,454180286	0,649699	0,999787
FAS2	6472,326217	0,374044011	0,7090301	0,527543224	0,597816	0,999787
FAV3	75,59987142	-0,315485095	0,8936587	-0,35302639	0,724069	0,999787
FCA1	123,3909075	0,19293515	0,7102591	0,271640521	0,785898	0,999787
FCR3	63,81048891	-0,252485125	0,8049021	-0,31368426	0,753761	0,999787
FCY21	331,7490415	0,218170628	0,7419772	0,294039539	0,768728	0,999787
FCY23	54,8788569	0,59927299	0,8172073	0,733318175	0,463364	0,999787
FCY24	14,75848819	-0,429155616	1,0350085	-0,4146397	0,678406	0,999787
FDH3	3181,12731	-0,176127186	0,7845733	-0,22448788	0,822378	0,999787
FEN1	52,3559509	-0,09885375	0,779784	-0,12677068	0,899122	0,999787
FEN12	147,3361051	-0,36728113	0,7021682	-0,52306718	0,600928	0,999787
FESUR1	277,5560692	0,24170269	0,7152806	0,337913113	0,735429	0,999787
FET34	272,0504544	-0,154317415	0,8057203	-0,19152729	0,848113	0,999787
FGR10	8,08902712	0,247723213	1,2885843	0,192244471	0,847551	0,999787
FGR14	150,3788435	-0,31941492	0,7098915	-0,44994891	0,652747	0,999787
FGR15	53,5881535	-0,144713253	0,7929116	-0,18250869	0,855184	0,999787
FGR16	22,24888093	-0,820208203	1,0037189	-0,81716927	0,413832	0,999787
FGR17	9,16695197	-0,245233145	1,257315	-0,19504512	0,845358	0,999787
FGR23	12,30251851	0,12224411	1,0985251	0,111280214	0,911394	0,999787
FGR24	44,46363378	-0,360049006	0,8105942	-0,44417912	0,656913	0,999787
FGR27	75,9856088	-0,051694037	0,7444848	-0,06943599	0,944643	0,999787
FGR28	11,37221134	-0,379064354	1,192157	-0,31796513	0,750511	0,999787
FGR29	42,84267928	-0,277577876	0,8092662	-0,34299948	0,731599	0,999787
FGR3	67,74922877	-0,315200887	0,7994799	-0,39425741	0,693391	0,999787

FGR32	408,768894	-0,450345191	0,7350442	-0,61267773	0,540089	0,999787
FGR34	88,15519866	-0,381278414	0,7455642	-0,51139579	0,609074	0,999787
FGR39	13,07692606	0,824249207	1,1175265	0,737565718	0,460778	0,999787
FGR43	11,52754068	0,140541252	1,1471269	0,12251587	0,90249	0,999787
FGR44	200,5424631	0,176093965	0,6871628	0,256262358	0,797748	0,999787
FGR51	68,648112	0,456264466	0,7542247	0,604944976	0,545216	0,999787
FGR6-1	10,46764949	-0,410399043	1,1561687	-0,35496468	0,722616	0,999787
FGR6-10	15,89685557	-0,71457658	1,0170946	-0,70256651	0,482326	0,999787
FGR6-3	5,390488512	-1,034507648	1,5205547	-0,68034885	0,496284	0,999787
FGR6-4	8,377485292	-0,67385977	1,2766761	-0,52782361	0,597622	0,999787
FHL1	153,5965169	0,317077617	0,7354712	0,43112172	0,66638	0,999787
FKH2	157,6811185	-0,047983696	0,786316	-0,06102343	0,951341	0,999787
FLC1	81,31677197	-0,077385808	0,7537381	-0,10266936	0,918225	0,999787
FLC2	152,9301675	0,088903101	0,6992061	0,127148629	0,898823	0,999787
FLC3	86,79741493	-0,106288415	0,7354367	-0,14452422	0,885087	0,999787
FLO8	249,7275334	-0,390154652	0,7757374	-0,5029468	0,615002	0,999787
FLO9	55,94922451	-0,174326006	0,8749592	-0,19923902	0,842076	0,999787
FLU1	114,1777722	0,929500744	1,0822943	0,858824385	0,390437	0,999787
FMO2	16,01009933	-1,001920173	1,1866493	-0,84432709	0,398487	0,999787
FMP27	198,5722925	-0,369039222	0,6877161	-0,53661566	0,591533	0,999787
FMT1	48,15926362	0,531280454	0,7939334	0,669175055	0,503384	0,999787
FOL1	104,2369245	0,58132806	0,8228692	0,706464744	0,479899	0,999787
FRE3	24,98459596	0,807934751	0,977581	0,826463208	0,408541	0,999787
FRE9	5,338651212	0,395752165	1,4993326	0,263952224	0,791817	0,999787
FRP2	89,20191425	0,983666711	0,9069118	1,084633317	0,278084	0,999787
FRP3	191,1118485	-0,451732773	0,7401294	-0,61034294	0,541635	0,999787
FRP6	124,8280749	-0,57582473	0,7076215	-0,81374685	0,41579	0,999787
FRS1	904,1372247	0,320413588	0,7148481	0,448226129	0,65399	0,999787
FRS2	848,2447181	0,679908154	0,7303816	0,930894411	0,351908	0,999787
FTH1	17,28938924	-0,7545856	1,0952487	-0,68896276	0,490847	0,999787
FTH2	51,77499542	0,771935685	0,8217763	0,939350105	0,347551	0,999787
FTR1	121,4091826	-0,55589763	0,8822874	-0,63006414	0,528653	0,999787
FUM11	236,1124291	0,482133955	0,7387158	0,652664982	0,513972	0,999787
FUM12	967,147997	0,60683403	0,6625652	0,915885724	0,359727	0,999787
FUN12	5823,432119	-0,794416334	0,7915118	-1,00366958	0,315538	0,999787
FUN31	85,73301023	-0,243609287	0,7800606	-0,31229534	0,754816	0,999787
FUR1	103,0473077	0,450748788	0,7310941	0,616540038	0,537538	0,999787
FUR4	9,507317804	-0,305954618	1,2816475	-0,23871979	0,811323	0,999787
FYV5	30,01747311	1,02318528	0,9196923	1,11252998	0,26591	0,999787
FZO1	156,786736	0,349262068	0,8389505	0,416308296	0,677184	0,999787
GAD1	260,7214113	-0,646856134	0,6867782	-0,94187055	0,346259	0,999787
GAL102	9,692918659	-1,431902204	1,4256108	-1,00441313	0,31518	0,999787
GAL4	546,2149074	-0,321854961	0,6683856	-0,48154082	0,630132	0,999787
GAP1	141,7942878	0,607620407	0,7557702	0,803975039	0,421411	0,999787
GAP4	399,6940794	0,210885396	0,7534271	0,279901511	0,779553	0,999787
GAP5	173,0512581	0,36152235	0,7192222	0,502657362	0,615205	0,999787
GAP6	456,9153779	-0,175963981	0,7839695	-0,22445258	0,822405	0,999787
GAR1	1076,388051	-0,188747536	0,7195854	-0,26230039	0,79309	0,999787
GBP2	998,5352723	0,039119097	0,6716112	0,058246645	0,953552	0,999787
GCD1	392,4453582	0,007193426	0,6870606	0,010469857	0,991646	0,999787

GCD11	1171,892427	0,584556386	0,6939912	0,842310944	0,399614	0,999787
GCD2	204,0962963	0,152767678	0,7716522	0,197974783	0,843065	0,999787
GCD7	153,2139742	0,687835455	0,7473692	0,920342211	0,357394	0,999787
GCF1	256,7836341	-0,204100448	0,7129474	-0,286277	0,774666	0,999787
GCN1	1453,919431	0,334738184	0,6926992	0,483237435	0,628927	0,999787
GCN2	54,53006957	-0,391366885	0,8092671	-0,48360658	0,628665	0,999787
GCN20	414,1090077	0,614999341	0,7472093	0,823061704	0,410473	0,999787
GCN3	101,788854	0,698795358	0,7429964	0,940509687	0,346956	0,999787
GCN4	290,9950088	0,592224541	0,8246425	0,718159159	0,472659	0,999787
GCR3	274,1561853	0,25968835	0,7661223	0,338964608	0,734636	0,999787
GCV1	244,3397473	0,266932929	0,6943475	0,384437087	0,700655	0,999787
GCV2	1291,363575	0,36529098	0,7335373	0,497985561	0,618494	0,999787
GCV3	264,1963307	-0,299822523	0,6798792	-0,44099379	0,659217	0,999787
GCY1	49,92205878	-0,481662557	0,9161792	-0,52572966	0,599076	0,999787
GDE1	130,0851628	-0,082909038	0,7254384	-0,11428819	0,909009	0,999787
GDI1	1034,116288	-0,334076804	0,7225348	-0,46236778	0,643818	0,999787
GDS1	411,9717412	-0,529184453	0,7599722	-0,69632082	0,486228	0,999787
GDT1	43,86771984	0,086837959	0,8051026	0,107859497	0,914107	0,999787
GEA2	92,64043197	0,05989849	0,8226735	0,072809556	0,941958	0,999787
GEF2	46,626408	-0,747751658	0,8334714	-0,89715337	0,369637	0,999787
GFA1	412,9043306	0,030288769	0,8195556	0,036957551	0,970519	0,999787
GGA2	300,8779202	0,014121112	0,6748703	0,020924188	0,983306	0,999787
GIG1	8,578215641	-1,062881485	1,2433446	-0,85485671	0,39263	0,999787
GIM5	59,34791385	-0,001227668	0,8231159	-0,00149149	0,99881	0,999787
GIN1	120,3793667	-0,124165972	0,7574438	-0,16392764	0,869788	0,999787
GIN4	468,7497386	0,012008422	0,6672032	0,017998147	0,98564	0,999787
GIR2	40,05615399	0,107524831	0,8716441	0,123358636	0,901823	0,999787
GIS2	744,8420728	0,123872249	0,6732218	0,183999167	0,854014	0,999787
GIT2	45,86566016	-0,286028642	0,8682748	-0,3294218	0,741837	0,999787
GLC7	341,5190059	-0,090727672	0,8268361	-0,10972872	0,912625	0,999787
GLE2	98,15808857	0,024625622	0,7657186	0,032160146	0,974344	0,999787
GLG2	5,965059282	-0,302362126	1,4303879	-0,2113847	0,832587	0,999787
GLN1	2126,578901	0,194470247	0,7503827	0,259161424	0,795511	0,999787
GLN3	122,9820165	0,567349216	0,7210359	0,786852912	0,431368	0,999787
GLN4	587,2116209	0,529198864	0,6831451	0,774650738	0,438546	0,999787
GLO1	315,0284479	0,375697676	0,6796164	0,552808467	0,580395	0,999787
GLO2	82,62327598	-0,038126872	0,777989	-0,04900696	0,960914	0,999787
GLO3	886,3683756	-0,330060865	0,7481134	-0,44119097	0,659075	0,999787
GLT1	900,1107473	-0,662191832	0,666085	-0,99415518	0,320147	0,999787
GLY1	531,8787907	0,289782487	0,7176906	0,403770787	0,686381	0,999787
GNA1	50,489782	-0,363173684	0,7840256	-0,46321662	0,643209	0,999787
GND1	3199,502633	0,409977583	0,6662557	0,615345685	0,538326	0,999787
GNP1	1564,384869	-0,785410416	0,702687	-1,11772443	0,263685	0,999787
GNP3	50,29599988	-1,04911389	0,9548417	-1,09873069	0,271886	0,999787
GOA1	19,98092112	0,10520992	0,9623717	0,109323583	0,912946	0,999787
GOR1	124,795582	-0,810763186	0,8657486	-0,936488	0,349022	0,999787
GPA2	91,76281727	0,810910382	0,7784502	1,041698497	0,297551	0,999787
GPD1	90,54843498	-0,356180437	0,7812847	-0,45589069	0,648469	0,999787
GPD2	838,9801041	-0,536563194	0,7283564	-0,73667674	0,461319	0,999787
GPI13	39,62748412	0,586216204	1,0021631	0,584950898	0,558581	0,999787

GPI7	45,32608651	-0,104295605	0,8167669	-0,12769322	0,898392	0,999787
GPI8	58,73814595	0,441512593	0,8277262	0,533404142	0,593754	0,999787
GPR1	207,8410562	-0,416252092	0,709864	-0,58638287	0,557618	0,999787
GPT1	19,88281971	-0,975487564	1,063506	-0,9172375	0,359018	0,999787
GRE3	46,53729361	-0,529697572	0,8227786	-0,64379113	0,519711	0,999787
GRF10	85,20162953	0,207294251	0,8511693	0,243540566	0,807587	0,999787
GRP1	43,52550952	-0,511847792	0,8116658	-0,630614	0,528293	0,999787
GRP2	80,4185879	-0,360820779	0,7393883	-0,48799905	0,625551	0,999787
GRR1	211,0215001	-0,194703542	0,684315	-0,28452329	0,776009	0,999787
GRS1	1458,680788	-0,485257563	0,6928052	-0,70042427	0,483662	0,999787
GRX3	134,1747663	0,012067151	0,8205721	0,014705777	0,988267	0,999787
GSC1	541,6478887	0,287979745	0,8312824	0,34642831	0,729021	0,999787
GSH2	246,2772479	0,407312998	0,6885899	0,591517505	0,554174	0,999787
GSL1	39,45004803	-0,191198292	0,8174396	-0,23389899	0,815063	0,999787
GSL2	78,32125778	-0,177243093	0,7575848	-0,23395809	0,815018	0,999787
GSP1	1513,282261	-0,113311592	0,6596226	-0,17178246	0,863609	0,999787
GTR1	18,2095512	-0,193200067	0,9751334	-0,19812681	0,842946	0,999787
GTT11	91,02852211	0,068128999	0,7557014	0,090153328	0,928165	0,999787
GUK1	73,82016655	0,211117472	0,9024734	0,233932082	0,815038	0,999787
GUP1	23,46611798	-0,808412931	0,9228352	-0,87601007	0,381025	0,999787
GUS1	954,1678272	0,377596486	0,7546397	0,500366582	0,616817	0,999787
GUT1	172,4266921	0,207334542	0,7342258	0,282385257	0,777648	0,999787
GVP36	121,6644465	-0,082448554	0,7793927	-0,10578564	0,915752	0,999787
GWT1	54,53126072	0,554445157	0,8164852	0,679063327	0,497098	0,999787
GYP1	117,8575477	-0,754658982	0,7536825	-1,00129556	0,316684	0,999787
GYP2	130,5376961	0,184253378	0,7778433	0,23687724	0,812752	0,999787
GYP5	141,9631305	0,142319139	0,7218802	0,197150635	0,84371	0,999787
GYP7	111,8704576	-0,185237343	0,7330905	-0,25268006	0,800515	0,999787
GYP8	18,69012281	-0,574433	0,9858683	-0,58266705	0,560117	0,999787
GZF3	173,6165422	0,516909579	0,7233235	0,7146313	0,474837	0,999787
HAC1	167,6355654	0,450068977	0,6980543	0,644747782	0,519091	0,999787
HAL21	73,19868426	0,03569375	0,7464393	0,047818689	0,961861	0,999787
HAL22	148,1000199	-0,026967981	0,7385464	-0,03651494	0,970872	0,999787
HAM1	48,62575155	0,414501163	0,9098696	0,45556106	0,648706	0,999787
HAP2	41,45658781	-0,33537367	0,8146337	-0,41168644	0,680569	0,999787
HAP31	31,75151319	0,327406638	0,8589356	0,381177154	0,703072	0,999787
HAP41	239,6840437	0,413731779	0,6845667	0,604370339	0,545597	0,999787
HAP42	60,34389023	-0,16860258	0,7735685	-0,2179543	0,827465	0,999787
HAP43	130,0846817	-0,212482835	0,7118227	-0,29850528	0,765318	0,999787
HAP5	113,903229	0,051147456	0,7125628	0,071779576	0,942777	0,999787
HAT1	94,99326222	-0,464488236	0,7250984	-0,64058645	0,521791	0,999787
HAT2	219,9765392	0,009069933	0,6996126	0,012964223	0,989656	0,999787
HBR1	226,8047377	0,314944976	0,6913263	0,455566304	0,648702	0,999787
HBR2	52,78428463	0,147965782	0,8400918	0,176130497	0,860191	0,999787
HCH1	112,6568257	0,32386645	0,7374875	0,43914842	0,660554	0,999787
HCM1	37,0123287	0,747180782	0,9881036	0,756176569	0,449543	0,999787
HCR1	2354,623659	-0,977726396	0,8575236	-1,14017432	0,254214	0,999787
HDA1	257,0496835	0,051927139	0,7402841	0,070144877	0,944078	0,999787
HEM1	222,0581719	0,591897735	0,7351322	0,805158172	0,420728	0,999787
HEM14	50,0513029	-0,526671176	0,8550477	-0,61595535	0,537924	0,999787

HEM15	363,6334724	0,582356133	0,670488	0,868555623	0,38509	0,999787
HEM2	220,9354199	-0,050097886	0,6934108	-0,07224849	0,942404	0,999787
HEM3	82,99437326	-0,25713011	0,9591097	-0,26809249	0,788628	0,999787
HEM4	20,93046118	0,181351609	0,984717	0,184166214	0,853883	0,999787
HET1	474,8995352	-0,430617466	0,7653088	-0,56267149	0,573659	0,999787
HEX1	60,14889025	-0,185358265	0,8997954	-0,20600046	0,836791	0,999787
HEX3	83,71002951	0,169137945	0,8059502	0,209861526	0,833776	0,999787
HFL1	56,7579114	0,3135183	0,7879814	0,397875233	0,690722	0,999787
HFL2	70,21771266	0,09801174	0,8016988	0,122255061	0,902697	0,999787
HGC1	7,755432554	-0,838179652	1,536447	-0,54553112	0,585388	0,999787
HGT14	19,78067916	-0,390000612	1,0067073	-0,38740219	0,698458	0,999787
HGT16	41,58036334	0,470731947	0,9182118	0,512661606	0,608188	0,999787
HGT20	69,94247274	0,235296894	0,7621344	0,308734112	0,757524	0,999787
HGT3	24,12270875	-0,731417915	0,9566074	-0,76459572	0,444512	0,999787
HGT5	34,35309096	-0,666285296	0,9388078	-0,7097143	0,477881	0,999787
HGT8	1117,210257	0,163756935	0,7048801	0,232318836	0,81629	0,999787
HHF1	876,6456948	-0,385960915	0,6620987	-0,58293566	0,559937	0,999787
HHF22	882,9705727	-0,320543684	0,6682958	-0,47964342	0,631481	0,999787
HHT1	225,8328902	-0,380421418	0,6856293	-0,55485003	0,578997	0,999787
HHT2	593,3764649	-0,412363753	0,6888514	-0,59862512	0,549423	0,999787
HHT21	1335,387624	-0,689636482	0,6588809	-1,04667856	0,295248	0,999787
HIR1	126,8649193	-0,067759738	0,7086906	-0,09561259	0,923828	0,999787
HIS1	213,6713906	0,208562004	0,6983007	0,298670769	0,765191	0,999787
HIS3	49,05535488	1,101657999	1,1132001	0,989631616	0,322354	0,999787
HIS4	922,0717784	0,716728517	0,6836256	1,048422622	0,294444	0,999787
HIS5	175,6379081	0,775527479	0,7487555	1,035755334	0,300316	0,999787
HIS7	281,7716054	0,460764185	0,7934959	0,580676203	0,561459	0,999787
HLJ1	105,4051116	-0,023781275	0,7257487	-0,03276792	0,97386	0,999787
HMG1	355,2406113	0,589704492	0,741193	0,795615345	0,426256	0,999787
HMI1	49,03390046	0,440563542	0,814927	0,540617166	0,588771	0,999787
HMX1	171,038478	-0,024072062	0,8174414	-0,02944806	0,976507	0,999787
HNM1	139,5148572	0,138169276	0,7038321	0,196309989	0,844368	0,999787
HNM4	8,05184954	-0,811470085	1,3717728	-0,59154845	0,554153	0,999787
HNT1	140,7339342	-0,384923358	0,81957	-0,46966499	0,638594	0,999787
HOC1	59,20148964	0,335327804	0,783541	0,427964604	0,668677	0,999787
HOF1	98,65594575	0,374102485	0,7294308	0,512869062	0,608043	0,999787
HOG1	210,4814764	-0,527562635	0,6892712	-0,76539197	0,444038	0,999787
HOL1	16,07584926	-0,32401268	1,0513156	-0,30819734	0,757932	0,999787
HOM2	612,2571908	0,258761671	0,666849	0,388036376	0,697989	0,999787
HOM3	140,8543726	-0,067365583	0,788722	-0,08541106	0,931935	0,999787
HOM6	274,5136887	-0,236130434	0,7053395	-0,33477556	0,737794	0,999787
HOS1	19,30832506	-0,661147725	0,9809244	-0,67400476	0,500308	0,999787
HOS2	31,43979584	0,666435063	0,8865116	0,751749974	0,452201	0,999787
HPC2	85,73670762	-0,224917343	0,7520088	-0,29908871	0,764872	0,999787
HRK1	506,6753866	-0,404917648	0,6760475	-0,59894849	0,549207	0,999787
HRQ2	7,265274335	0,620866653	1,3279216	0,46754768	0,640108	0,999787
HRT1	92,43737367	0,09415838	0,7303794	0,128917086	0,897423	0,999787
HRT2	98,30138716	-0,565529334	0,728374	-0,77642715	0,437497	0,999787
HSK3	8,350758587	-0,204980497	1,3046472	-0,15711566	0,875154	0,999787
HSL1	220,1919293	-0,525936723	0,856324	-0,61417961	0,539097	0,999787

HSP60	955,007957	0,590168693	0,7844024	0,752380052	0,451823	0,999787
HSP78	192,7004921	-0,25914587	0,7006307	-0,36987511	0,711476	0,999787
HSP90	7614,501813	0,017801578	0,6510979	0,027340861	0,978188	0,999787
HST2	20,10446926	-0,606677631	0,9491481	-0,63918122	0,522705	0,999787
HST7	155,8208498	-0,16451398	0,6964619	-0,23621389	0,813267	0,999787
HTA2	695,2392146	-0,616914287	0,6690639	-0,92205588	0,356499	0,999787
HTA3	322,1349925	-0,542959641	0,6737706	-0,80585237	0,420328	0,999787
HUT1	23,70207959	0,517322116	0,9837541	0,525865258	0,598982	0,999787
HXK1	200,249384	0,058119245	0,756708	0,076805376	0,938778	0,999787
HYM1	72,4196401	-0,791017173	0,7656294	-1,0331594	0,301529	0,999787
HYR3	183,1687487	-0,771710353	0,6906801	-1,11731959	0,263858	0,999787
HYR4	53,3409997	-0,443148418	0,7782577	-0,56941088	0,569077	0,999787
HYS2	88,22015357	0,81356471	0,7835734	1,038275064	0,299142	0,999787
HYU1	316,985436	-0,247740392	0,6865533	-0,36084653	0,718214	0,999787
IAH1	107,9765242	-0,538438136	0,7660917	-0,70283775	0,482157	0,999787
IDH1	1884,641161	0,192124413	0,6552846	0,293192343	0,769375	0,999787
IDH2	1006,447914	0,029035668	0,7113368	0,040818455	0,967441	0,999787
IDI1	150,0091362	0,558045416	0,7490557	0,744998521	0,456273	0,999787
IDP1	366,5731413	-0,177208303	0,6783712	-0,26122617	0,793918	0,999787
IDP2	108,2458654	0,132929259	0,7534729	0,176422084	0,859962	0,999787
IFA21	9,188764751	-0,304361266	1,2303265	-0,24738252	0,804612	0,999787
IFD6	6,74865403	-0,708242106	1,3471608	-0,52572945	0,599076	0,999787
IFF3	86,04033153	-0,885356889	0,8345516	-1,06087735	0,288746	0,999787
IFF5	34,26300571	0,310400706	0,896687	0,346163924	0,72922	0,999787
IFF6	34,90882924	-0,756563398	0,8549615	-0,88490932	0,376206	0,999787
IFF8	6,640888716	-1,53546463	1,3888674	-1,10555161	0,268921	0,999787
IFF9	123,5718594	-0,097724226	0,796133	-0,12274863	0,902306	0,999787
IFG3	48,03349496	-0,319711004	0,7909023	-0,40423579	0,686039	0,999787
IFI3	6,640723608	0,571333428	1,3971495	0,408927899	0,682593	0,999787
IFK2	37,6527677	-0,183179843	0,8622927	-0,21243349	0,831769	0,999787
IFU5	545,2612428	-0,67590778	0,8839024	-0,76468602	0,444459	0,999787
IHD2	6,004094204	0,191854838	1,4428363	0,132970625	0,894217	0,999787
ILS1	2217,082525	0,621112235	0,737693	0,841965767	0,399807	0,999787
ILV1	233,1311392	0,567688811	0,7319232	0,775612513	0,437978	0,999787
ILV2	350,5131024	0,256776518	0,8008874	0,320615013	0,748502	0,999787
ILV3	1310,739683	0,122583144	0,6610269	0,185443505	0,852881	0,999787
ILV5	1147,189085	-0,036970681	0,6889366	-0,0536634	0,957203	0,999787
ILV6	265,5371933	-0,415940407	0,6770448	-0,61434691	0,538986	0,999787
IME2	9,833605895	-0,802517389	1,3832334	-0,58017498	0,561797	0,999787
IMH3	1004,295765	0,689148399	0,8143274	0,846279322	0,397397	0,999787
IML1	125,9352035	0,602452732	0,7692617	0,783157015	0,433535	0,999787
IMP1	49,09653764	-0,811553261	0,9197582	-0,88235505	0,377585	0,999787
IMP2	57,09799495	-0,335214428	0,8367354	-0,40062176	0,688699	0,999787
INN1	137,3988538	0,15993623	0,7628981	0,209642969	0,833946	0,999787
INO1	35,75953711	-0,155605123	0,8389707	-0,18547147	0,852859	0,999787
INP51	56,06376009	0,093333059	0,7745921	0,120493176	0,904092	0,999787
INT1	351,6075113	0,178410487	0,9316024	0,191509255	0,848127	0,999787
IPK1	20,77626072	-0,465245729	1,0418284	-0,44656657	0,655188	0,999787
IPK2	57,37381585	-0,49880229	0,8155704	-0,61159929	0,540803	0,999787
IPL1	26,56981435	-0,466625994	0,908027	-0,51388997	0,607329	0,999787

IPP1	1191,645036	0,060609171	0,656506	0,092320822	0,926443	0,999787
IPT1	40,39829511	-0,089148223	0,9951929	-0,08957884	0,928622	0,999787
IQG1	190,7759371	0,351521787	0,9216474	0,381405928	0,702902	0,999787
IRA2	178,7971219	-0,186364919	0,8325782	-0,22384075	0,822881	0,999787
IRE1	94,49038467	-0,400180377	0,7391105	-0,54143512	0,588208	0,999787
IRR1	146,3336094	0,322465982	0,7140482	0,451602532	0,651555	0,999787
IRS4	139,3368136	0,48600612	0,7474463	0,650222136	0,515549	0,999787
ISA1	171,7099341	0,110301933	0,6971959	0,158207953	0,874293	0,999787
ISA2	46,1111148	0,423572604	0,7986172	0,530382525	0,595847	0,999787
ISC1	46,00514932	-0,770302979	0,7955163	-0,96830575	0,332892	0,999787
ISN1	139,307463	-0,732528753	0,7038262	-1,04078074	0,297977	0,999787
IST1	51,38282969	-0,494380938	0,8078173	-0,61199596	0,54054	0,999787
ISW2	99,09304504	0,29436754	0,8078563	0,364381085	0,715573	0,999787
ISY1	25,35087221	0,453879672	0,9036738	0,502260543	0,615484	0,999787
ITR1	95,63140838	-0,362239739	0,7760175	-0,46679326	0,640648	0,999787
ITS1	14,89392652	0,926543839	1,0624888	0,872050452	0,383181	0,999787
ITS2	12,90189421	-1,087641963	1,1867221	-0,91650938	0,3594	0,999787
JAB1	112,1880774	-0,694765303	0,7346764	-0,94567529	0,344314	0,999787
JEM1	76,946751	0,01895549	0,7604839	0,024925563	0,980114	0,999787
KAP120	157,9672769	0,35652403	0,7870668	0,452978102	0,650565	0,999787
KAR2	2378,692443	0,565446448	0,6668201	0,8479745	0,396452	0,999787
KAR3	31,13878345	0,283373043	0,9081434	0,312035584	0,755013	0,999787
KAR4	107,7454148	0,181262968	0,8339458	0,217355802	0,827931	0,999787
KAR9	18,29163564	0,77925038	0,99006	0,787073859	0,431239	0,999787
KCH1	44,73668514	0,558834849	0,8062843	0,693099037	0,488247	0,999787
KCS1	26,26212328	-0,046408967	0,9005946	-0,05153147	0,958902	0,999787
KEL1	379,0817426	-0,226911138	0,6902802	-0,32872325	0,742365	0,999787
KEX2	192,2919408	-0,014495342	0,6911867	-0,02097167	0,983268	0,999787
KGD1	1276,354125	0,215120977	0,7791386	0,276101035	0,78247	0,999787
KGD2	1537,438961	-0,374885723	0,6629357	-0,56549336	0,571738	0,999787
KIC1	186,3043236	-0,025610953	0,7158748	-0,03577574	0,971461	0,999787
KIN2	521,6388587	-0,165546501	0,6665968	-0,24834576	0,803867	0,999787
KIN3	227,8953609	-0,151724468	0,8247693	-0,1839599	0,854045	0,999787
KIP1	35,07897631	-0,605037115	0,9316987	-0,64939142	0,516085	0,999787
KIP2	37,67254041	0,701092802	1,0451768	0,670788745	0,502355	0,999787
KIP3	66,60576691	-0,342854414	0,8268063	-0,41467318	0,678381	0,999787
KIP4	44,93082679	0,05343133	0,8287551	0,0644718	0,948595	0,999787
KIS1	421,6442484	-0,720624309	0,6964227	-1,03475134	0,300785	0,999787
KNS1	11,96883613	-0,42955989	1,1197143	-0,38363347	0,70125	0,999787
KOG1	256,8910474	-0,765506357	0,7477896	-1,02369219	0,305981	0,999787
KRE1	12,69011931	0,604873185	1,1305517	0,535024768	0,592633	0,999787
KRE5	143,0438754	0,822465626	0,7553814	1,088808368	0,276238	0,999787
KRE6	137,0809412	0,227485614	0,899439	0,252919459	0,80033	0,999787
KRE9	273,2947823	-0,328412591	0,720109	-0,45605956	0,648347	0,999787
KRS1	1589,126462	0,267788401	0,7149043	0,374579379	0,707973	0,999787
KSR1	66,43224357	0,130923991	0,7725178	0,16947698	0,865421	0,999787
KTR2	19,98760456	-0,258155641	0,958658	-0,26928857	0,787708	0,999787
KTR4	19,84706381	0,543601888	1,0430512	0,521165129	0,602252	0,999787
LAB5	238,4755791	0,668277485	0,7563811	0,883519537	0,376956	0,999787
LAC1	152,3746483	-0,470221582	0,8138377	-0,57778302	0,563411	0,999787

LAG1		64,37967498	-0,171115864	0,7828606	-0,21857769	0,826979	0,999787
LAP4		78,91098259	-0,606482391	0,770525	-0,78710284	0,431222	0,999787
LAP41		199,9093757	-0,534948293	0,6949979	-0,76971207	0,441471	0,999787
LAS1		76,64748952	0,281648	0,7719108	0,36487118	0,715208	0,999787
LAT1		2138,736248	0,346626545	0,6552689	0,52898363	0,596817	0,999787
LCB2		168,7339443	0,473577802	0,8291451	0,571163991	0,567888	0,999787
LCB4		56,32217629	0,360404421	0,7818639	0,460955445	0,644831	0,999787
LEA1		31,40108997	0,398617527	0,8708299	0,457744412	0,647136	0,999787
LEM3		278,3042917	0,018709654	0,7254062	0,025791969	0,979423	0,999787
LEU3		176,6081355	0,452287412	0,7068033	0,639905619	0,522234	0,999787
LEU4		42,12582278	-0,872831475	0,8143232	-1,07184897	0,283788	0,999787
LHP1		250,3041133	0,433144685	0,7498075	0,577674479	0,563484	0,999787
LHS1		287,5447505	0,336506052	0,6873156	0,489594643	0,624421	0,999787
LIG1		152,8032042	0,420503853	0,8765241	0,479740209	0,631412	0,999787
LIG4		72,18868997	0,153806082	0,7532932	0,204178249	0,838214	0,999787
	04-lip	16,5116605	0,087842931	1,0214602	0,085997406	0,931468	0,999787
	05-lip	21,94771397	-0,236209627	0,960673	-0,24587931	0,805776	0,999787
	06-lip	19,04586513	-0,699441126	0,9641557	-0,72544418	0,46818	0,999787
	08-lip	73,85106731	-0,981349482	0,9859255	-0,99535864	0,319562	0,999787
LKH1		237,7590266	0,000407568	0,7730491	0,000527221	0,999579	0,999787
LMO1		102,5766772	-0,128641868	0,8154677	-0,15775225	0,874652	0,999787
LPD1		2168,817114	0,410623645	0,6571058	0,624897325	0,532038	0,999787
LPG20		22,91230148	0,008197088	0,9461312	0,008663796	0,993087	0,999787
LPI9		202,6678827	-0,715914215	0,6885021	-1,03981407	0,298426	0,999787
LPT1		121,8760576	-0,350749318	0,7098796	-0,49409693	0,621238	0,999787
LRG1		229,5596135	0,445324199	0,9301348	0,478773823	0,6321	0,999787
LRO1		177,2885312	0,170668311	0,7136072	0,239162812	0,810979	0,999787
LSC1		1282,863235	-0,440302204	0,6548444	-0,67237687	0,501344	0,999787
LSC2		1510,00269	0,319107229	0,6576528	0,485221449	0,627519	0,999787
LSM6		120,4329236	0,615287292	0,7137655	0,862029989	0,388671	0,999787
LSP1		514,2872014	-0,418012845	0,7633411	-0,54760954	0,58396	0,999787
LTE1		86,85075914	-0,356004129	1,0151747	-0,35068264	0,725826	0,999787
LTP1		82,26239828	-0,605694254	0,7479873	-0,80976545	0,418075	0,999787
LYP1		10,19939968	-0,661765785	1,1719541	-0,56466869	0,572299	0,999787
LYS1		106,6224959	0,041064977	0,8577461	0,047875445	0,961816	0,999787
LYS12		381,1774148	-0,561388981	0,6879801	-0,81599601	0,414502	0,999787
LYS14		13,53907051	0,001621628	1,0720203	0,001512684	0,998793	0,999787
LYS143		32,91831236	-0,66155857	0,8645696	-0,76518833	0,444159	0,999787
LYS144		28,1328067	-0,024148086	0,9170623	-0,026332	0,978993	0,999787
LYS2		436,1209309	0,047443821	0,7223507	0,065679762	0,947633	0,999787
LYS21		222,2090192	0,285918126	0,7574423	0,377478448	0,705818	0,999787
LYS4		194,2034812	0,162477484	0,7140223	0,227552403	0,819994	0,999787
LYS9		246,1897012	0,757799876	0,8386289	0,903617657	0,366198	0,999787
MAD2		9,857176506	-0,142723085	1,1874845	-0,12018943	0,904333	0,999787
MAK21		1455,595221	-0,101212249	0,7323288	-0,13820601	0,890078	0,999787
MAK32		31,0073714	-0,346533076	0,8665045	-0,39992069	0,689215	0,999787
MAL2		51,66461627	-0,745559631	0,9701177	-0,76852496	0,442175	0,999787
MAM33		152,2279345	0,699716272	0,9140316	0,765527447	0,443958	0,999787
MAS1		246,7515321	0,603731116	0,6815952	0,88576197	0,375746	0,999787
MAS2		107,80019	0,642169993	0,909984	0,705693707	0,480379	0,999787

MBF1	1157,081526	-0,164896404	0,7092445	-0,23249587	0,816153	0,999787
MBP1	147,8765568	-0,64970419	0,7180036	-0,904876	0,365531	0,999787
MCA1	185,5788562	-0,171230573	0,701821	-0,24398041	0,807246	0,999787
MCD1	99,67886895	1,033270985	0,9456197	1,092691879	0,274529	0,999787
MCD4	25,33984512	0,526907806	1,0700729	0,492403657	0,622434	0,999787
MCI4	239,3476877	0,0678353	0,6847245	0,099069473	0,921083	0,999787
MCM1	138,5054505	0,058402321	0,7521964	0,077642382	0,938113	0,999787
MCM2	136,8097569	0,649834189	0,8177934	0,794619044	0,426835	0,999787
MCT1	56,49637419	-0,304387954	0,776975	-0,39176028	0,695235	0,999787
MCU1	36,92668776	0,885534253	0,8892197	0,995855471	0,31932	0,999787
MDH1-1	2490,545412	-0,111842202	0,6819731	-0,16399797	0,869733	0,999787
MDJ1	147,2585915	0,41331471	0,7720166	0,535370254	0,592394	0,999787
MDJ2	14,18889578	0,329276737	1,103946	0,2982725	0,765495	0,999787
MDL1	127,7344409	0,444583241	0,7121222	0,624307529	0,532426	0,999787
MDL2	166,4880983	0,597658002	0,8871591	0,673676258	0,500517	0,999787
MDM10	23,62562585	0,229630725	0,914721	0,251039086	0,801784	0,999787
MDM34	52,82540067	0,31775377	0,7993158	0,397532204	0,690975	0,999787
MDN1	2444,924366	0,063250441	0,7628215	0,082916436	0,933918	0,999787
MDR1	75,17630185	1,065148305	0,9548974	1,115458391	0,264654	0,999787
MDS3	208,2074297	0,086542271	0,6858404	0,126184271	0,899586	0,999787
MEA1	7,334575012	-0,485308037	1,3221509	-0,36705948	0,713575	0,999787
MEC3	45,18918332	0,314420428	0,8354117	0,376365829	0,706645	0,999787
MED1	63,2543326	0,224050341	0,7758988	0,288762342	0,772763	0,999787
MED10	16,62023733	-0,455728632	1,0090781	-0,45162869	0,651536	0,999787
MED11	16,4259492	-0,469356528	1,0090478	-0,46514799	0,641825	0,999787
MED14	55,44370842	-0,553066019	1,0406721	-0,53145079	0,595106	0,999787
MED15	228,6925441	-0,434269905	0,7075268	-0,61378576	0,539357	0,999787
MED16	82,39141762	-0,516136929	0,7603916	-0,67877775	0,497279	0,999787
MED17	59,88431396	0,264441936	0,8664592	0,305198386	0,760215	0,999787
MED19	46,03871766	-0,766047038	0,8443233	-0,90729113	0,364253	0,999787
MED20	30,21162068	0,09229912	0,8641234	0,106812425	0,914938	0,999787
MED21	19,7180677	-0,623746076	0,9559084	-0,65251655	0,514068	0,999787
MED4	66,70632542	-0,681586123	0,77019	-0,88495845	0,376179	0,999787
MED7	14,85426265	-0,294664065	1,0538242	-0,27961407	0,779774	0,999787
MED9	45,19853679	-0,763397347	0,8597785	-0,88790005	0,374595	0,999787
MEP1	162,1546733	0,795723915	0,7037505	1,130690449	0,258185	0,999787
MEP2	53,01157197	-0,061694388	0,7813966	-0,078954	0,937069	0,999787
MES1	536,0683578	0,523827151	0,8197356	0,639019608	0,52281	0,999787
MET14	98,69411181	0,063985946	0,7503457	0,085275293	0,932043	0,999787
MET15	288,1970061	-0,142122127	0,7149284	-0,1987921	0,842425	0,999787
MET18	28,96694592	-0,359450195	0,8714915	-0,41245404	0,680007	0,999787
MET2	296,9593131	0,559493171	0,6941711	0,805987431	0,42025	0,999787
MET28	151,5124891	0,210351708	0,7840441	0,268290653	0,788476	0,999787
MET3	70,21970956	0,720206243	0,7565683	0,951938225	0,341128	0,999787
MET4	38,32060324	0,230684512	0,8278941	0,278640105	0,780521	0,999787
MET6	2337,998943	-0,548967584	0,7562218	-0,72593466	0,467879	0,999787
MET8	14,92388068	-0,478678267	1,1744353	-0,40758166	0,683581	0,999787
MEU1	214,142221	0,314289928	0,6875946	0,457086061	0,647609	0,999787
MEX67	258,4721015	0,583952718	0,7636193	0,764717126	0,44444	0,999787
MFG1	148,2232822	-0,62933094	0,7474004	-0,8420265	0,399773	0,999787

MGE1	246,8418569	0,263875076	0,7159193	0,368582139	0,712439	0,999787
MGM101	82,05889724	0,320977764	0,7380019	0,434928072	0,663615	0,999787
MIA40	339,4627554	0,671729048	0,7439914	0,902872063	0,366594	0,999787
MID1	38,81478181	0,092964813	0,8229729	0,112962186	0,910061	0,999787
MIF2	94,9925092	-0,052575533	0,7499869	-0,07010193	0,944113	0,999787
MIG1	469,4495292	-0,424557463	0,8307341	-0,51106298	0,609307	0,999787
MIH1	110,355828	-0,115492821	0,759179	-0,15212858	0,879086	0,999787
MIM1	44,3916817	-0,104396204	0,8109971	-0,12872575	0,897575	0,999787
MIR1	1727,081672	0,004548114	0,7459676	0,006096931	0,995135	0,999787
MIS11	1190,069491	-0,051166954	0,7699316	-0,06645649	0,947014	0,999787
MIT1	98,49289883	0,20703617	0,7339887	0,282069956	0,77789	0,999787
MKC1	279,2046936	-0,174486303	0,7139909	-0,2443817	0,806935	0,999787
MKK2	148,1453059	-0,712215629	0,7303779	-0,97513304	0,329494	0,999787
MLC1	119,9263206	0,028094827	0,7175148	0,039155744	0,968766	0,999787
MLH1	11,74017983	-0,311987449	1,132436	-0,27550116	0,782931	0,999787
MLP1	555,583097	-0,267016455	0,7028342	-0,37991388	0,704009	0,999787
MMD1	1006,990183	-0,721085343	0,794798	-0,90725616	0,364271	0,999787
MMS21	38,33306371	0,896963451	0,9100595	0,985609714	0,324325	0,999787
MMS22	46,89359336	-0,107914135	0,8463928	-0,12749888	0,898546	0,999787
MNL1	190,7009065	-0,048185356	0,6902331	-0,06981026	0,944345	0,999787
MNN10	279,7775209	0,251291451	0,7286532	0,344871137	0,730191	0,999787
MNN11	18,12287613	-0,983476695	1,0061902	-0,97742624	0,328358	0,999787
MNN13	10,63030869	-0,603467929	1,1520642	-0,5238145	0,600408	0,999787
MNN14	114,5696417	0,039520333	0,7133153	0,05540374	0,955817	0,999787
MNN15	31,8960626	0,289275345	0,8623764	0,335439791	0,737293	0,999787
MNN2	146,8347058	0,925920104	0,8295424	1,116181824	0,264344	0,999787
MNN21	104,7332028	0,23679457	0,7731932	0,306255352	0,75941	0,999787
MNN23	108,7758691	0,837576076	0,8640785	0,969328691	0,332381	0,999787
MNN24	15,32943209	0,391045255	1,0514042	0,371926653	0,709947	0,999787
MNN26	84,56340147	0,040224728	0,9634997	0,041748566	0,966699	0,999787
MNN4-4	22,18681521	0,429582815	1,0311727	0,416596383	0,676974	0,999787
MNN9	214,3276896	-0,097635873	0,6982893	-0,13982152	0,888801	0,999787
MNR2	259,5517458	0,233013125	0,6795914	0,342872378	0,731694	0,999787
MNS1	158,9832299	0,440984103	0,7152388	0,61655503	0,537528	0,999787
MNT1	474,7586848	-0,115658011	0,7620997	-0,1517623	0,879374	0,999787
MNT2	46,6516417	0,095678031	0,8736539	0,109514802	0,912794	0,999787
MNT3	8,426577605	-0,478699107	1,2614491	-0,3794835	0,704329	0,999787
MNT4	6,83670514	-1,37539351	1,3685002	-1,00503716	0,314879	0,999787
MOB1	71,48853808	0,406575425	0,7557942	0,537944639	0,590615	0,999787
MOB2	91,75361357	0,048474116	0,7406248	0,065450299	0,947815	0,999787
MODF	37,76672923	-0,466464925	0,8296142	-0,56226725	0,573934	0,999787
MON2	61,58696734	-0,46112753	0,9690016	-0,47587904	0,634161	0,999787
MP65	925,9708182	0,318414679	0,6890332	0,462118056	0,643997	0,999787
MPH1	104,4361699	-0,225735057	0,7192179	-0,31386184	0,753626	0,999787
MPS1	57,4702766	0,611452678	0,7969547	0,767236396	0,442941	0,999787
MPT5	77,81002218	-0,322272268	0,7561904	-0,42617872	0,669978	0,999787
MRE11	105,9768505	-0,081326249	0,7229637	-0,11249008	0,910435	0,999787
MRP17	150,5253337	0,708442008	0,815287	0,868947996	0,384876	0,999787
MRP2	184,7210683	-0,030361771	0,7532569	-0,04030733	0,967848	0,999787
MRP20	157,5877684	0,750530236	1,1187769	0,670848905	0,502317	0,999787

MRP7	88,21154458	0,367343396	1,1803434	0,311217383	0,755635	0,999787
MRP8	53,05092425	0,097662846	0,7805217	0,125125092	0,900425	0,999787
MRPL10	141,3228916	0,673293749	0,9186486	0,732917584	0,463609	0,999787
MRPL19	128,7453943	0,280112406	0,7065977	0,396424186	0,691792	0,999787
MRPL27	181,6449793	0,482942746	0,6921045	0,697788739	0,485309	0,999787
MRPL3	114,6840364	0,65594657	1,0478571	0,625988543	0,531322	0,999787
MRPL33	47,73036521	0,39834749	1,0628558	0,374789779	0,707817	0,999787
MRPL36	192,7188145	0,746328013	0,697729	1,06965317	0,284775	0,999787
MRPL37	48,08625402	0,794182097	0,8679956	0,914961015	0,360212	0,999787
MRPL40	237,8586453	0,859559853	0,7760645	1,10758812	0,26804	0,999787
MRPL6	59,75753359	0,540670646	1,0123932	0,534052046	0,593306	0,999787
MRPL8	214,2983008	0,773455773	0,8760389	0,882901151	0,37729	0,999787
MRPS9	192,7180997	0,482482937	0,815557	0,591599262	0,554119	0,999787
MRR1	73,07382451	0,748821906	0,796444	0,940206654	0,347112	0,999787
MRR2	127,3104424	-0,653729167	0,7414585	-0,88168003	0,37795	0,999787
MRS2	36,83882514	0,097305732	0,833675	0,116719025	0,907083	0,999787
MRS4	114,0142206	0,136809045	0,7304007	0,18730683	0,85142	0,999787
MRS7	546,745198	-0,080503807	0,662834	-0,12145396	0,903331	0,999787
MRT4	148,0894494	0,950683132	0,989853	0,960428647	0,33684	0,999787
MSB1	298,8186379	-0,493240904	0,699142	-0,70549461	0,480502	0,999787
MSB2	177,3318447	-0,403342009	0,7215162	-0,55902	0,576148	0,999787
MSC7	281,4139081	0,77013715	0,6777301	1,136347905	0,255811	0,999787
MSF1	151,2054998	0,859457404	0,7594895	1,131625203	0,257792	0,999787
MSH2	132,8287665	0,120070008	0,7053147	0,170236086	0,864824	0,999787
MSH3	42,94078185	-0,285572061	0,8354705	-0,34180989	0,732494	0,999787
MSH6	203,9238842	0,357605002	0,8379836	0,426744617	0,669565	0,999787
MSI3	1318,285169	0,277415724	0,8468373	0,327590338	0,743221	0,999787
MSK1	68,38004952	0,243855634	0,7676416	0,317668614	0,750736	0,999787
MSM1	53,81010756	0,647898705	0,858595	0,754603436	0,450487	0,999787
MSS11	77,08139744	-0,255749104	0,7631685	-0,33511488	0,737538	0,999787
MSS4	156,0715788	0,070110278	0,7626332	0,091931847	0,926752	0,999787
MSS51	220,6297536	0,932314825	0,8496157	1,097337054	0,272494	0,999787
MST1	98,7799752	0,361216327	0,7360234	0,49076748	0,623591	0,999787
MSU1	49,29358133	0,831630121	0,9517817	0,87376139	0,382248	0,999787
MSW1	50,08435369	0,23709457	0,8396196	0,282383322	0,77765	0,999787
MTLA1	45,18969033	-0,830574488	0,8644463	-0,9608167	0,336644	0,999787
MTM1	75,02225465	0,829016651	0,7612079	1,089080487	0,276118	0,999787
MTO1	57,63815539	0,773657361	0,8372263	0,924071945	0,355449	0,999787
MTR10	100,6588771	0,073841187	0,874975	0,084392339	0,932744	0,999787
MTR2	143,9377781	0,078564456	0,7808354	0,100615902	0,919855	0,999787
MTS1	696,7084428	0,560669963	0,683096	0,820777758	0,411773	0,999787
MTW1	7,240975258	-0,746447564	1,3252233	-0,56326172	0,573257	0,999787
MUB1	155,8816428	0,405382711	0,6999392	0,579168452	0,562476	0,999787
MUC1	253,9549234	0,285778701	0,7037471	0,406081558	0,684683	0,999787
MUQ1	332,9303339	-0,635916323	0,836532	-0,76018175	0,447146	0,999787
MVD	773,3054652	0,041727741	0,6680007	0,062466616	0,950191	0,999787
MXR1	130,2621848	0,617782602	0,7312573	0,844822477	0,39821	0,999787
MYO1	119,590688	-0,034267607	1,086655	-0,03153495	0,974843	0,999787
MYO2	395,803319	0,032712612	0,8451754	0,038705116	0,969125	0,999787
MYO5	495,0826801	-0,558433297	0,6717224	-0,83134538	0,405779	0,999787

NAB3	434,661666	0,271649173	0,6843732	0,396931364	0,691418	0,999787
NAG1	46,73850542	-0,359567922	0,8462199	-0,42491075	0,670902	0,999787
NAM2	155,1436783	0,443427067	0,7825177	0,566667158	0,57094	0,999787
NAN1	192,7941929	0,923735561	0,9548536	0,967410706	0,333339	0,999787
NAT2	207,8764293	-0,615750892	0,7980198	-0,77159856	0,440352	0,999787
NAT4	16,9990678	0,248141312	1,0046945	0,246981864	0,804922	0,999787
NAT5	118,5890757	-0,810103619	0,8740164	-0,92687467	0,353992	0,999787
NBN1	56,69943858	-0,533012821	0,8358061	-0,63772306	0,523654	0,999787
NBP35	98,99906842	0,151729274	0,7515804	0,201880298	0,84001	0,999787
NCB2	114,5359717	0,491839124	0,7160456	0,686882384	0,492157	0,999787
NCE102	385,6065111	-0,456173148	0,7055153	-0,64658153	0,517903	0,999787
NCE4	14,41587153	-1,097214349	1,0888699	-1,00766337	0,313616	0,999787
NCP1	373,3445623	0,247116902	0,7045237	0,350757387	0,72577	0,999787
NDE1	1209,683879	-0,574009472	0,7001587	-0,8198277	0,412314	0,999787
NDH51	605,4441568	0,083275642	0,7849825	0,106085989	0,915514	0,999787
NDT80	440,6783185	0,515536972	0,733066	0,703261373	0,481893	0,999787
NGG1	157,7356711	0,732294557	0,7128861	1,027225248	0,304314	0,999787
NGT1	6,635733558	-0,223349922	1,3640975	-0,16373458	0,86994	0,999787
NHP6A	708,0489979	-0,124091713	0,7084668	-0,17515531	0,860958	0,999787
NHX1	155,6914533	-0,256320428	0,7108392	-0,36058848	0,718407	0,999787
NIF3	84,12750258	-0,072823586	0,7574794	-0,09613936	0,92341	0,999787
NIK1	261,0443542	0,565999457	0,6810047	0,831124135	0,405904	0,999787
NIP1	366,2951901	0,032826662	0,9656454	0,033994532	0,972882	0,999787
NIP100	56,43005973	-0,390772537	0,7744557	-0,50457701	0,613856	0,999787
NIP7	54,04969603	1,108120137	0,9724103	1,139560277	0,25447	0,999787
NIT2	37,23064072	-0,674515239	0,9280898	-0,72677796	0,467362	0,999787
NIT3	184,3685381	-0,682914688	0,7623327	-0,89582239	0,370348	0,999787
NMD5	189,6817517	0,603435519	0,9565358	0,6308551	0,528135	0,999787
NMT1	91,58672314	0,383675894	0,7932294	0,48368847	0,628607	0,999787
NOC2	864,8432433	0,29227133	0,6798473	0,42990733	0,667263	0,999787
NOP10	340,0434193	-0,152603549	0,6888499	-0,22153383	0,824677	0,999787
NOP5	3392,921027	-0,100741685	0,743646	-0,13546994	0,89224	0,999787
NOP6	335,4926161	0,784116884	0,736945	1,064010094	0,287324	0,999787
NOT3	181,5934671	0,332850248	0,8191244	0,406348846	0,684486	0,999787
NOT5	477,2467008	-0,142231631	0,7286787	-0,19519115	0,845243	0,999787
NPL4	48,41730479	-0,154033382	0,9209987	-0,16724604	0,867176	0,999787
NPR2	37,92512091	-0,405228077	0,8405205	-0,48211565	0,629724	0,999787
NPT1	248,6585755	0,079103312	0,7247816	0,1091409	0,913091	0,999787
NRG1	1325,727069	0,188057656	0,7098366	0,26493092	0,791063	0,999787
NRM1	65,08178494	0,163500732	0,9750428	0,167685696	0,866831	0,999787
NRP1	61,66220914	0,036423321	0,8982899	0,040547401	0,967657	0,999787
NSP1	545,1802172	0,546713135	0,7420601	0,736750433	0,461274	0,999787
NTF2	172,0738002	0,072375934	0,7151165	0,101208591	0,919385	0,999787
NTG1	40,85266248	-0,813605884	0,9279418	-0,87678551	0,380603	0,999787
NUC2	801,1856799	0,114450673	0,6758931	0,169332507	0,865535	0,999787
NUF2	35,12901494	-0,020529981	0,9395266	-0,02185141	0,982566	0,999787
NUO1	307,1107549	-0,725210891	0,7016017	-1,03365045	0,3013	0,999787
NUO2	368,9739875	-0,197963182	0,6810901	-0,29065638	0,771314	0,999787
NUP	40,28995142	1,20234996	1,1101522	1,083049655	0,278786	0,999787
NUP159	498,8012274	-0,038989906	0,6641296	-0,05870828	0,953184	0,999787

NUP188	222,1377119	0,02714754	0,9566002	0,028379193	0,97736	0,999787
NUP49	119,8310697	0,272416848	0,7426087	0,366837662	0,71374	0,999787
NUP60	85,51189472	0,175469832	0,8292657	0,211596635	0,832422	0,999787
NUP82	200,590562	0,328067352	0,7725294	0,424666497	0,67108	0,999787
NUP84	92,42376654	0,111028966	0,8528005	0,130193359	0,896413	0,999787
NUP85	113,4764819	0,187502153	0,7921396	0,236703415	0,812887	0,999787
OAC1	12,08553572	0,073924523	1,1066713	0,066798987	0,946742	0,999787
OBPA	295,350738	-0,293969817	0,6794555	-0,43265497	0,665265	0,999787
OCA1	40,92401957	0,214039302	0,860506	0,248736566	0,803565	0,999787
OCA6	12,72907811	0,147677654	1,1164628	0,132272795	0,894769	0,999787
OCH1	82,93607312	0,750925985	0,7387224	1,016519803	0,309382	0,999787
OFD1	217,2167206	-0,114127519	0,6844977	-0,16673178	0,867581	0,999787
OGG1	16,14674266	0,367028726	1,0193671	0,360055489	0,718806	0,999787
OLE1	3927,89452	-0,046232452	0,6509209	-0,07102622	0,943377	0,999787
OLE2	132,3991564	0,044434738	0,7595994	0,058497592	0,953352	0,999787
OPI1	53,77608723	-0,088667346	0,7765378	-0,11418291	0,909093	0,999787
OPI3	20,60782334	-0,988446194	0,9585094	-1,03123262	0,302432	0,999787
OPT6	11,05913081	-0,648992412	1,1519789	-0,56337181	0,573182	0,999787
ORC1	168,2218293	0,128570478	0,7179163	0,179088396	0,857868	0,999787
ORC3	78,79947212	0,074317337	0,7430019	0,100023079	0,920326	0,999787
ORC4	106,2347762	0,579323074	0,7207139	0,80381842	0,421502	0,999787
ORM1	66,65503486	-0,352532366	0,8017676	-0,43969395	0,660159	0,999787
OSH3	181,4155945	0,480585189	0,7047482	0,681924662	0,495287	0,999787
OST1	78,57909776	0,202130726	0,8101704	0,249491613	0,802981	0,999787
OXR1	19,80216289	-0,131754411	0,971738	-0,13558635	0,892148	0,999787
OYE22	24,93847241	0,825171292	1,0178241	0,810720941	0,417526	0,999787
PAD1	23,11665869	-0,525316593	1,0050811	-0,52266092	0,60121	0,999787
PAM16	143,8302036	0,791714957	0,7075098	1,119016166	0,263133	0,999787
PAM17	71,25474292	0,654941014	0,8944539	0,732224487	0,464032	0,999787
PAM18	77,63066202	0,630946165	1,0711124	0,589056917	0,555823	0,999787
PAN3	85,35834544	0,193828438	0,8282848	0,234011819	0,814976	0,999787
PAN6	65,55277253	0,976256438	0,8687044	1,123807355	0,261095	0,999787
PAP1	131,1357966	-0,069331926	0,7044758	-0,09841634	0,921602	0,999787
PBP2	423,7943692	0,279567388	0,6881721	0,406246326	0,684562	0,999787
PBS2	274,7172876	0,501271599	0,7113109	0,704715237	0,480987	0,999787
PCD1	30,52479597	-0,721893599	0,8611	-0,83833886	0,40184	0,999787
PCL1	12,80344496	0,586004588	1,0991765	0,533130565	0,593943	0,999787
PCL2	21,44364311	-0,526320871	0,9407845	-0,5594489	0,575855	0,999787
PCL5	24,28286996	-0,793716363	0,9077233	-0,87440347	0,381899	0,999787
PCT1	140,1746401	-0,28684815	0,7023911	-0,40838809	0,682989	0,999787
PDA1	3611,790336	0,571290442	0,6533087	0,874457096	0,381869	0,999787
PDB1	1222,716496	0,817517364	0,8274294	0,988020638	0,323143	0,999787
PDC12	10,90628777	-0,229255193	1,2442115	-0,18425741	0,853812	0,999787
PDE2	119,1648988	-0,482042807	0,9078791	-0,53095485	0,59545	0,999787
PDI1	1592,858097	-0,096516926	0,7109484	-0,13575798	0,892013	0,999787
PDR6	79,7393084	-0,458854772	0,7381804	-0,62160251	0,534203	0,999787
PDS5	116,4152038	0,002980042	0,7443707	0,004003438	0,996806	0,999787
PDX1	646,0742356	0,63479434	0,6955253	0,912683353	0,361409	0,999787
PEA2	78,72284142	0,304440078	0,883086	0,344745683	0,730286	0,999787
PEP1	312,4496968	0,176820933	0,6736969	0,262463614	0,792964	0,999787

PEP12	43,58507609	-0,672196005	0,8215233	-0,81823126	0,413225	0,999787
PEP3	11,68553888	-0,964195115	1,1335273	-0,85061484	0,394983	0,999787
PEP7	43,07810091	-0,393305876	0,8060413	-0,48794756	0,625587	0,999787
PEP8	152,7132339	-0,496902967	0,7409041	-0,67067112	0,50243	0,999787
PET100	42,09963489	0,611300273	0,8319683	0,734763914	0,462483	0,999787
PET9	5790,539701	0,210423126	0,708425	0,297029501	0,766444	0,999787
PEX1	102,4289319	-0,653339319	0,7187464	-0,9089984	0,363351	0,999787
PEX11	10,91819657	-0,405122791	1,1933454	-0,33948495	0,734244	0,999787
PEX14	189,9608508	-0,5793128	0,7318055	-0,79162132	0,428582	0,999787
PEX3	41,99800136	-0,30970982	0,8123315	-0,38126039	0,70301	0,999787
PEX8	27,30278273	-0,457317161	0,881246	-0,51894383	0,6038	0,999787
PFK26	150,8470062	-0,19473768	0,723541	-0,26914533	0,787818	0,999787
PFY1	396,3042994	-0,57204611	0,7439482	-0,76893272	0,441933	0,999787
PGA1	61,3861531	0,117120183	0,8345612	0,140337438	0,888393	0,999787
PGA13	28,85690523	0,569960171	0,8989769	0,634009837	0,526074	0,999787
PGA14	1052,708989	-0,37520465	0,6847928	-0,54790977	0,583754	0,999787
PGA18	54,3842331	-0,445670185	0,8299981	-0,53695324	0,5913	0,999787
PGA25	58,66419829	-0,490842371	0,7751052	-0,63325905	0,526564	0,999787
PGA32	9,908114469	0,551187546	1,2395642	0,444662353	0,656564	0,999787
PGA33	84,89963487	-0,535973894	0,8715813	-0,6149442	0,538592	0,999787
PGA38	250,9399155	0,107476398	0,7819039	0,137454743	0,890671	0,999787
PGA4	607,9659611	-0,68658217	0,759791	-0,90364609	0,366183	0,999787
PGA41	6,314042032	-0,940777502	1,5305816	-0,6146536	0,538784	0,999787
PGA42	5,403690303	-1,542124177	1,6843638	-0,91555289	0,359901	0,999787
PGA43	9,84954098	-0,909387721	1,3386196	-0,67934738	0,496918	0,999787
PGA44	8,365893846	0,160353086	1,3080203	0,122592197	0,90243	0,999787
PGA45	238,9761686	0,449055333	0,6866895	0,653942354	0,513149	0,999787
PGA49	17,96922734	-0,237960841	0,9856534	-0,24142445	0,809226	0,999787
PGA52	220,3291457	0,575366557	0,7227285	0,796103311	0,425972	0,999787
PGA53	208,0181167	-0,419197098	0,7845688	-0,5343025	0,593132	0,999787
PGA54	169,6747703	0,315983754	0,8073853	0,391366764	0,695526	0,999787
PGA55	40,45029652	-0,115273362	0,8137203	-0,14166214	0,887347	0,999787
PGA57	14,04837265	0,550667805	1,1154034	0,493693857	0,621522	0,999787
PGA59	7251,573181	-0,287772397	0,6517902	-0,44151079	0,658843	0,999787
PGA6	169,1891284	-0,324728904	0,9646018	-0,33664555	0,736384	0,999787
PGA63	1253,8451	-0,472007253	0,6547865	-0,72085669	0,470998	0,999787
PGI1	1512,345966	-0,287568066	0,6546818	-0,4392486	0,660481	0,999787
PGM2	197,1767933	-0,66178466	0,7199199	-0,91924759	0,357966	0,999787
PHA2	28,15598266	0,448497323	1,0709365	0,418789843	0,67537	0,999787
PHB1	157,2306762	-0,101389427	0,7622259	-0,13301756	0,894179	0,999787
PHB2	127,1456938	0,333792433	0,7795629	0,428178937	0,668521	0,999787
PHM5	454,2626487	-0,506152049	0,7563947	-0,6691639	0,503391	0,999787
PHM7	359,9234261	-0,0870183	0,8222279	-0,10583233	0,915715	0,999787
PHO114	22,08662691	-0,528175416	0,9802981	-0,53879061	0,590031	0,999787
PHO13	32,66653168	0,515666003	0,9072874	0,568360174	0,56979	0,999787
PHO81	53,52234473	-0,727636315	0,8423248	-0,86384291	0,387674	0,999787
PHO85	182,8422958	-0,716396697	0,7177157	-0,99816219	0,318201	0,999787
PHO87	414,0705713	-0,061981487	0,7553832	-0,08205304	0,934605	0,999787
PHO88	193,5130331	0,086467627	0,6926994	0,124827065	0,90066	0,999787
PHO91	282,4967507	-0,44859336	0,6751923	-0,66439347	0,506439	0,999787

PHR2	731,5589458	0,299341761	0,7185293	0,416603402	0,676969	0,999787
PHR3	161,1258511	-0,739521807	0,8648053	-0,85513097	0,392479	0,999787
PIKA	74,01510702	0,127618614	0,8196796	0,15569329	0,876275	0,999787
PIM1	904,8263633	-0,14297541	0,6690073	-0,21371277	0,830771	0,999787
PIN4	211,3510997	-0,451760401	0,7112954	-0,63512345	0,525348	0,999787
PIR1	399,6814003	-0,128289523	0,670799	-0,19124882	0,848331	0,999787
PKC1	298,8520157	-0,233326959	0,7242448	-0,32216587	0,747327	0,999787
PKH2	144,9562446	-0,380300079	0,7421784	-0,51241057	0,608364	0,999787
PLB2	8,374828123	0,344839285	1,281726	0,269042914	0,787897	0,999787
PLB3	168,6492413	-0,151267586	0,7170995	-0,21094365	0,832931	0,999787
PLB5	115,4136521	-0,859056951	0,8039373	-1,06856214	0,285267	0,999787
PLC1	25,06395502	-0,445532547	1,0325443	-0,43149	0,666112	0,999787
PLC2	64,16637672	0,358910043	0,8243131	0,435405001	0,663268	0,999787
PLD1	795,6597658	-0,277402406	0,6598726	-0,42038784	0,674202	0,999787
PMA1	1920,6765	0,046842817	0,7885123	0,059406581	0,952628	0,999787
PMI1	293,2865529	-0,157054491	0,7124944	-0,2204291	0,825537	0,999787
PMM1	58,95925193	-0,669471617	0,8961698	-0,74703659	0,455041	0,999787
PMR1	387,9612647	0,576085948	0,6826072	0,84394946	0,398698	0,999787
PMS1	16,82473511	0,484639472	1,0747917	0,450914796	0,652051	0,999787
PMT1	699,7204242	-0,104470017	0,6608636	-0,15808106	0,874393	0,999787
PMT2	365,273852	0,287879186	0,808121	0,356232777	0,721666	0,999787
PMT4	394,8667918	-0,297634818	0,6728107	-0,4423753	0,658218	0,999787
PMT5	70,30077256	0,269316305	0,8321001	0,323658561	0,746197	0,999787
PMT6	294,0136742	-0,349621088	0,674657	-0,51822049	0,604304	0,999787
PNC1	14,6369508	-0,001090153	1,0528057	-0,00103547	0,999174	0,999787
PNP1	146,1685436	-0,681766132	0,7726906	-0,88232745	0,3776	0,999787
POB3	594,4559053	-0,52627522	0,6919303	-0,7605899	0,446902	0,999787
POL1	283,1071416	0,186416095	0,7556369	0,246700616	0,80514	0,999787
POL2	179,9991679	0,36745086	0,7946756	0,462391019	0,643801	0,999787
POL3	318,2456677	0,658644959	0,7049484	0,934316592	0,350141	0,999787
POL30	107,7397556	0,207287178	0,8392497	0,246991058	0,804915	0,999787
POL5	125,7457799	-0,158757686	0,740611	-0,21436042	0,830266	0,999787
POL93	54,48991762	-0,556317137	0,8834397	-0,62971716	0,52888	0,999787
POM152	153,555305	0,573757833	0,7502558	0,764749592	0,444421	0,999787
POP2	148,5855308	-0,487598703	0,6983402	-0,69822519	0,485036	0,999787
POR1	1293,447516	0,017537438	0,6568457	0,026699479	0,978699	0,999787
PPE1	35,54698694	-0,668193051	0,8546824	-0,78180272	0,434331	0,999787
PPG1	51,13340052	-0,17050883	0,7833157	-0,21767576	0,827682	0,999787
PPH21	205,3234446	-0,385181332	0,6892188	-0,55886654	0,576253	0,999787
PPH3	57,892947	-0,939382217	0,9115662	-1,0305145	0,302769	0,999787
PPR1	17,91706675	0,154664053	1,0340343	0,149573432	0,881101	0,999787
PPS1	20,45771953	-1,053654767	1,1111499	-0,94825619	0,342999	0,999787
PPT1	58,4771148	0,636143818	0,8852795	0,718579663	0,4724	0,999787
PPZ1	71,26065919	-0,664860796	0,7514375	-0,88478524	0,376272	0,999787
PR26	411,555717	-0,132067017	0,6826709	-0,19345634	0,846602	0,999787
PRC2	18,11708626	-1,120412452	1,051063	-1,06598028	0,286433	0,999787
PRC3	77,32471361	-0,526015971	0,7708045	-0,68242459	0,494971	0,999787
PRD1	361,5896474	-0,487490255	0,6704485	-0,72711072	0,467158	0,999787
PRE1	249,8268424	0,317888941	0,7463457	0,425927226	0,670161	0,999787
PRE10	338,9416077	-0,809234312	0,7562191	-1,07010564	0,284572	0,999787

PRE2	248,6047869	0,507681102	0,7293167	0,696105121	0,486363	0,999787
PRE3	189,482284	0,426602824	0,7235997	0,589556425	0,555488	0,999787
PRE5	845,5138933	-0,721836746	0,818054	-0,88238277	0,37757	0,999787
PRE6	316,8260629	-0,221183017	0,6879132	-0,3215275	0,747811	0,999787
PRE8	177,6862487	0,252444478	0,7348109	0,343550272	0,731185	0,999787
PRE9	194,7651246	0,605251488	0,701467	0,862836736	0,388227	0,999787
PRI2	11,13180543	-0,855055806	1,1618032	-0,73597305	0,461747	0,999787
PRK1	220,6238185	0,326957462	0,6840751	0,477955475	0,632682	0,999787
PRN3	28,62020707	1,001536101	0,928705	1,078422248	0,280845	0,999787
PRO1	263,1956437	0,046031228	0,7258139	0,063420148	0,949432	0,999787
PRO2	104,264075	0,246761063	0,8559068	0,28830366	0,773114	0,999787
PRO3	38,6351534	-0,308864742	0,8221809	-0,37566518	0,707166	0,999787
PRP13	33,90468926	0,111496921	0,8739263	0,127581607	0,89848	0,999787
PRP22	175,8024365	-0,399332035	0,7864594	-0,50775925	0,611622	0,999787
PRP3	59,23470554	0,794550174	0,7757895	1,024182635	0,305749	0,999787
PRP39	15,27566709	0,213578549	1,0637704	0,200775041	0,840874	0,999787
PRP42	28,71799345	0,060725169	0,9030973	0,067241005	0,94639	0,999787
PRP45	10,48736369	0,351405952	1,2301538	0,285660179	0,775138	0,999787
PRP8	567,6410972	0,638089675	0,6818203	0,935861961	0,349344	0,999787
PRR2	13,95437348	-0,280678977	1,0707719	-0,26212769	0,793223	0,999787
PRS	94,25219134	0,618261821	0,7288555	0,848263895	0,396291	0,999787
PRS1	168,9547544	0,608090147	0,8200906	0,741491444	0,458396	0,999787
PRS5	265,0948936	0,381363392	0,6774861	0,562909521	0,573496	0,999787
PRT1	718,1481549	0,569956301	0,8410356	0,677683881	0,497972	0,999787
PRX1	422,867616	0,308858405	0,7139601	0,432598972	0,665306	0,999787
PSA2	13,12225094	-0,518044843	1,1625518	-0,44561011	0,655879	0,999787
PSD1	299,2480677	-0,15283289	0,6740421	-0,22674086	0,820625	0,999787
PSD2	106,2090202	0,422098833	0,731075	0,577367381	0,563691	0,999787
PSF2	19,78011464	-0,178084789	0,9644424	-0,18465052	0,853503	0,999787
PSF3	13,14565645	0,920691203	1,1220662	0,820532014	0,411913	0,999787
PSO2	8,103433914	0,027138656	1,2650425	0,021452762	0,982884	0,999787
PST1	23,96133068	-0,224795579	0,9092533	-0,24723097	0,804729	0,999787
PSY2	28,96330736	0,262001695	0,9119448	0,287299959	0,773883	0,999787
PTC1	120,5367469	-0,33010821	0,7804796	-0,42295561	0,672328	0,999787
PTC5	219,3458376	-0,46449497	0,6903535	-0,67283637	0,501051	0,999787
PTC7	311,5988943	-0,073385436	0,7196044	-0,10198026	0,918772	0,999787
PTH1	37,90080898	0,156165696	0,9483117	0,164677602	0,869198	0,999787
PTH2	13,95219878	-0,285456385	1,051237	-0,27154332	0,785973	0,999787
PTK2	323,7055776	-0,523248755	0,7583482	-0,68998487	0,490204	0,999787
PTP1	160,9316203	0,071382206	0,7254394	0,098398577	0,921616	0,999787
PTP3	240,7035357	0,790509453	0,746096	1,059527847	0,289359	0,999787
PTR3	23,03962874	-0,203377622	0,9319831	-0,21822029	0,827257	0,999787
PUF3	87,18387024	0,023995166	0,8526055	0,028143338	0,977548	0,999787
PUP1	379,5479513	-0,224240063	0,6784699	-0,33050849	0,741016	0,999787
PUP2	189,4238028	-0,093207146	0,7106618	-0,13115542	0,895652	0,999787
PUP3	142,5576635	0,045347342	0,714712	0,06344841	0,949409	0,999787
PUS7	158,0142919	1,075746641	0,9648186	1,114973001	0,264862	0,999787
PUT1	47,57230967	-0,332937172	0,8123071	-0,40986615	0,681904	0,999787
PUT2	151,6386667	0,733955656	0,7208897	1,018124698	0,308619	0,999787
PUT3	116,4419705	0,471277164	0,729224	0,64627218	0,518103	0,999787

PUT4	7,824404179	-1,250960338	1,3592873	-0,92030607	0,357413	0,999787
PXA2	84,10066463	-0,701121979	0,7498814	-0,9349771	0,3498	0,999787
QCR2	683,9271277	0,017674011	0,7431234	0,023783412	0,981025	0,999787
QCR8	405,2517527	-0,29748107	0,6705073	-0,44366566	0,657284	0,999787
QCR9	552,800023	-0,419634084	0,6897888	-0,60835158	0,542954	0,999787
QDR2	58,26570419	0,365797533	0,7988014	0,457933023	0,647001	0,999787
QDR3	20,64080377	0,265087865	0,96209	0,27553334	0,782907	0,999787
RAC1	107,5693849	0,036700208	0,7174253	0,051155441	0,959202	0,999787
RAD1	107,8821608	-0,261342006	0,7232466	-0,36134565	0,717841	0,999787
RAD10	25,43821824	-0,643275207	0,896369	-0,71764549	0,472976	0,999787
RAD16	225,8231223	-0,717836164	0,7575656	-0,94755643	0,343355	0,999787
RAD18	42,46318377	0,291440721	0,8582622	0,33957072	0,73418	0,999787
RAD2	89,33754423	0,125127683	0,7327764	0,170758335	0,864414	0,999787
RAD23	475,0057064	0,498492888	0,6822791	0,730629016	0,465006	0,999787
RAD3	499,8141453	-0,53149834	0,7841689	-0,6777855	0,497908	0,999787
RAD32	424,2668185	0,013036699	0,6805836	0,019155176	0,984717	0,999787
RAD50	182,4047503	0,524488576	0,7225997	0,725835551	0,46794	0,999787
RAD51	120,8803496	0,447496622	0,7155231	0,625411832	0,531701	0,999787
RAD52	90,02650626	-0,371164228	0,7286906	-0,50935777	0,610501	0,999787
RAD53	91,33550218	-0,240984843	0,7295733	-0,3303093	0,741166	0,999787
RAD54	53,57205431	-0,241881252	0,8411699	-0,28755337	0,773689	0,999787
RAD6	101,7661043	-0,199676914	0,7582915	-0,26332474	0,7923	0,999787
RAD7	72,6251417	0,326023933	0,7891367	0,413139971	0,679504	0,999787
RAM1	36,43543098	0,159892281	0,8668648	0,18444893	0,853661	0,999787
RAM2	27,5382688	0,043624335	0,9357539	0,046619453	0,962817	0,999787
RAP1	89,35106915	0,110460771	0,7502677	0,147228482	0,882952	0,999787
RAS1	330,0725523	0,256536462	0,8264821	0,310395666	0,75626	0,999787
RAT1	312,127681	0,477170042	0,6810011	0,700689063	0,483497	0,999787
RAV2	32,73873669	-0,239892092	0,8493518	-0,28244136	0,777605	0,999787
RAX1	100,7525472	0,216371387	0,8331302	0,259708974	0,795088	0,999787
RAX2	65,80165104	0,021850356	0,9044339	0,024159151	0,980726	0,999787
RBD1	83,19481572	-0,75379504	0,7390222	-1,01998976	0,307733	0,999787
RBE1	104,3888459	-0,068845719	0,7191147	-0,09573678	0,92373	0,999787
RBF1	122,000088	-0,509321198	0,7415667	-0,68681777	0,492198	0,999787
RBK1	116,6142771	-0,394295249	0,7242596	-0,54441146	0,586158	0,999787
RBP1	182,8887097	-0,182936474	0,6961033	-0,26280075	0,792704	0,999787
RBR3	50,61913055	-0,385451967	0,8056128	-0,47845808	0,632324	0,999787
RBT1	15,51519806	-1,183171695	1,120806	-1,05564366	0,291131	0,999787
RBT4	67,89022844	-0,352278153	0,7568054	-0,46548052	0,641587	0,999787
RCA1	231,9494766	-0,697897814	0,7956271	-0,87716702	0,380396	0,999787
RCE1	55,77644366	-0,497575853	0,7841701	-0,63452543	0,525738	0,999787
RCK2	469,6975165	-0,80826705	0,7532443	-1,0730477	0,28325	0,999787
RCN1	21,991575	-0,209934234	0,9585931	-0,21900245	0,826648	0,999787
RCY1	23,99576904	0,275211622	1,0853151	0,253577621	0,799822	0,999787
RDH54	86,30734469	-0,017189825	0,7655615	-0,02245388	0,982086	0,999787
RDI1	160,4077588	-0,035984398	0,7071789	-0,05088443	0,959418	0,999787
REP1	54,04184498	-0,143272317	0,7828259	-0,18301938	0,854783	0,999787
RER2	134,3533893	-0,130361574	0,7821902	-0,16666224	0,867636	0,999787
RET2	410,628578	-0,685352178	0,6927565	-0,98931175	0,322511	0,999787
REV3	57,2938875	-0,158732409	0,8232203	-0,19281888	0,847101	0,999787

REX2	53,47272695	0,820123864	0,7878019	1,041028017	0,297863	0,999787
REX3	21,11589117	0,525442541	1,089856	0,482121089	0,62972	0,999787
RFA1	486,0213858	0,146056084	0,6694139	0,218185034	0,827285	0,999787
RFA2	146,5523722	0,377957289	0,7197372	0,525132359	0,599491	0,999787
RFC1	575,2994576	-0,195403224	0,6850349	-0,28524564	0,775456	0,999787
RFC2	39,27345858	-0,171131432	0,8789375	-0,19470263	0,845626	0,999787
RFC3	49,23621284	0,558681726	0,9614177	0,581101989	0,561172	0,999787
RFC5	67,67388497	0,055867197	0,9287355	0,060154047	0,952033	0,999787
RFX1	31,76809469	0,281138735	0,8619681	0,32615908	0,744304	0,999787
RFX2	36,64792381	-0,613592197	0,8492039	-0,72254994	0,469956	0,999787
RGA2	227,6782557	-0,036563037	0,6847953	-0,05339265	0,957419	0,999787
RGD1	51,20067974	-0,335088232	0,9588212	-0,34947938	0,726729	0,999787
RGS2	32,63123023	-1,066017262	0,9649592	-1,10472777	0,269278	0,999787
RHB1	25,34702273	-0,47006017	0,9138177	-0,51439163	0,606978	0,999787
RHD1	234,0416536	0,264288817	0,7168183	0,3686971	0,712354	0,999787
RHD2	68,34019238	0,244959662	0,8085368	0,302966627	0,761915	0,999787
RHO2	33,22928263	0,000482887	0,9032831	0,000534591	0,999573	0,999787
RHO3	142,9472108	-0,131022086	0,7122899	-0,18394489	0,854057	0,999787
RIA1	379,101899	0,811257372	0,7810543	1,038669683	0,298958	0,999787
RIB1	175,3467003	0,760390596	0,6946582	1,094625539	0,273681	0,999787
RIB5	95,87222524	-0,311687564	0,7569592	-0,41176268	0,680513	0,999787
RIC1	31,10322891	-0,813710021	0,9135488	-0,89071322	0,373083	0,999787
RIM1	154,3717547	-0,463276252	0,7140274	-0,64882141	0,516454	0,999787
RIM101	890,1567675	-0,307501318	0,6584086	-0,46703718	0,640473	0,999787
RIM13	56,68188146	-0,384589631	0,7756415	-0,49583424	0,620011	0,999787
RIM15	192,0618228	-0,106203513	0,6971193	-0,15234625	0,878914	0,999787
RIM2	65,58004729	0,750089077	0,8848153	0,847735167	0,396585	0,999787
RIM20	50,81976239	-0,604082031	0,9048074	-0,66763607	0,504366	0,999787
RIM21	58,65157171	-0,386839472	0,8759673	-0,44161404	0,658769	0,999787
RIM8	62,76768423	-0,559130338	0,870088	-0,64261353	0,520475	0,999787
RIO2	680,9555602	-0,180049176	0,7987399	-0,22541652	0,821655	0,999787
RIP1	2117,991919	-0,159103431	0,677273	-0,23491771	0,814273	0,999787
RIT1	40,93036704	0,810455814	0,8251716	0,982166343	0,326018	0,999787
RLI1	692,1048164	1,017083895	0,9020703	1,12749957	0,259531	0,999787
RLM1	55,0573459	0,84457981	0,7970036	1,059693834	0,289284	0,999787
RLP24	394,1389972	0,725116364	0,676786	1,071411591	0,283984	0,999787
RMP1	13,19129869	-0,033715961	1,1081912	-0,03042432	0,975729	0,999787
RMS1	47,66597846	0,463850838	0,9331657	0,497072301	0,619138	0,999787
RMT2	146,4869094	0,664407933	0,7156103	0,928449313	0,353175	0,999787
RNA1	354,9168283	0,51615419	0,7014878	0,735799218	0,461853	0,999787
RNH1	50,23431711	-0,344384427	0,8067575	-0,42687478	0,669471	0,999787
RNH35	55,65959065	-0,564825935	0,7979737	-0,70782529	0,479054	0,999787
RNR1	875,6446258	0,462900253	0,8116652	0,570309359	0,568468	0,999787
RNR21	1197,240335	0,42231555	0,8346861	0,505957355	0,612887	0,999787
ROD1	139,215409	-0,269733432	0,7620377	-0,35396336	0,723366	0,999787
ROM2	268,0777391	-0,013224031	0,8264223	-0,01600154	0,987233	0,999787
ROT1	24,16020945	-0,355786197	0,9851793	-0,36113851	0,717996	0,999787
ROT2	147,571608	0,249309677	0,7373789	0,338102547	0,735286	0,999787
RPA190	850,1751281	0,854962253	0,9824605	0,87022553	0,384177	0,999787
RPA34	424,6463196	0,379004607	0,75719	0,500540958	0,616694	0,999787

RPB11	75,68206578	0,987205316	0,9012376	1,095388571	0,273346	0,999787
RPB4	223,6595023	-0,501390693	0,713242	-0,70297413	0,482072	0,999787
RPB7	109,144422	-0,473972863	0,7629254	-0,62125718	0,53443	0,999787
RPB8	165,176073	-0,515456319	0,7376922	-0,69874174	0,484713	0,999787
RPC10	153,8888705	-0,2622018	0,842804	-0,3111065	0,75572	0,999787
RPC19	85,01220162	0,952060626	0,941557	1,011155631	0,311942	0,999787
RPC31	215,2685287	0,085212151	0,7432315	0,114650886	0,908722	0,999787
RPC40	161,8476305	0,653542265	0,9057575	0,721542193	0,470576	0,999787
RPD3	74,60627114	-0,006014549	0,7476469	-0,00804464	0,993581	0,999787
RPD31	297,7515925	-0,493061948	0,6965	-0,70791375	0,478999	0,999787
RPG1A	610,8698865	0,295001577	0,7695025	0,383366603	0,701448	0,999787
RPL10	3810,193449	0,665280596	0,6663674	0,998369103	0,3181	0,999787
RPL10A	2667,124245	0,461692881	0,761152	0,606571232	0,544135	0,999787
RPL11	1631,762304	0,123821344	0,6544581	0,189196755	0,849939	0,999787
RPL12	3881,242454	0,207594549	0,6526329	0,318087792	0,750418	0,999787
RPL13	8051,014271	0,311694865	0,6582278	0,47353646	0,63583	0,999787
RPL14	1488,230722	0,51908739	0,7341564	0,707052883	0,479534	0,999787
RPL15A	3707,222438	0,176035575	0,6686424	0,263273136	0,79234	0,999787
RPL16A	5440,220153	0,632982621	0,6659646	0,950474827	0,341871	0,999787
RPL17B	1768,883586	0,50927458	0,8274959	0,615440592	0,538264	0,999787
RPL18	3610,045887	0,349470016	0,6536846	0,534615666	0,592916	0,999787
RPL19A	6728,250253	0,622387141	0,6696492	0,929422712	0,35267	0,999787
RPL2	5519,647795	0,083761879	0,6684472	0,125308147	0,90028	0,999787
RPL20B	1336,378503	0,269103061	0,8055086	0,334078439	0,73832	0,999787
RPL21A	2836,272934	0,383959143	0,6600554	0,581707452	0,560764	0,999787
RPL23A	1568,876107	0,443829174	0,6668707	0,665540082	0,505705	0,999787
RPL24A	2977,45871	0,282281896	0,662621	0,426008065	0,670102	0,999787
RPL25	2418,764155	0,527419359	0,6746586	0,781757384	0,434357	0,999787
RPL27A	2644,538561	0,534743002	0,6774032	0,789401349	0,429877	0,999787
RPL28	4654,293837	0,170959842	0,6510047	0,262609233	0,792852	0,999787
RPL29	1556,861658	-0,213330058	0,6644334	-0,32107065	0,748157	0,999787
RPL3	10176,12307	0,73320442	0,6601918	1,110592986	0,266744	0,999787
RPL30	1487,326307	0,417785899	0,6614614	0,631610399	0,527641	0,999787
RPL32	2215,350456	0,490416358	0,6885885	0,712205284	0,476338	0,999787
RPL35	760,038159	0,302511733	1,0396284	0,290980639	0,771066	0,999787
RPL37B	1971,489556	-0,257337377	0,6535065	-0,39377935	0,693744	0,999787
RPL38	1603,58755	0,549559929	0,701868	0,782996173	0,433629	0,999787
RPL39	1090,811776	0,314738968	0,6617774	0,475596451	0,634362	0,999787
RPL40B	1961,05629	-0,351899127	0,6555788	-0,53677623	0,591422	0,999787
RPL42	2925,793806	0,168429224	0,6603901	0,255045056	0,798688	0,999787
RPL43A	1267,631905	0,247195689	0,6553216	0,377212806	0,706015	0,999787
RPL4B	5637,640994	0,463532934	0,6526159	0,710269104	0,477537	0,999787
RPL5	4705,201895	0,422375926	0,7535668	0,560502275	0,575137	0,999787
RPL6	2569,986335	0,393491406	0,7771073	0,506354043	0,612608	0,999787
RPL82	711,10752	0,749407635	0,6904785	1,085345304	0,277769	0,999787
RPL8B	2368,296954	0,265416832	0,7393885	0,358968035	0,719619	0,999787
RPL9B	1471,012296	0,812099564	0,8456259	0,960353197	0,336877	0,999787
RPM2	97,41659109	0,059046704	0,8161489	0,072347951	0,942325	0,999787
RPN10	549,4154364	-0,845710547	0,7862398	-1,07563951	0,282089	0,999787
RPN2	1063,44477	-0,147143938	0,6567435	-0,22405086	0,822718	0,999787

RPN3	459,8722086	0,018156943	0,6725215	0,026998309	0,978461	0,999787
RPN4	144,6791989	0,060441075	0,7241379	0,083466247	0,933481	0,999787
RPN5	344,2681745	0,309660134	0,6735204	0,459763585	0,645686	0,999787
RPN6	69,08363602	-0,14541825	0,8661186	-0,16789646	0,866665	0,999787
RPN7	556,5952899	0,156183322	0,6745084	0,231551325	0,816887	0,999787
RPN8	337,2658305	-0,21203389	0,6729598	-0,31507662	0,752703	0,999787
RPO21	2612,115619	-0,264962477	0,6637263	-0,39920444	0,689743	0,999787
RPO26	220,6775005	0,769147825	0,6973232	1,10300048	0,270027	0,999787
RPP0	4828,560464	-0,76903275	0,7945924	-0,96783306	0,333128	0,999787
RPP1A	2487,148209	0,00168506	0,6606016	0,002550796	0,997965	0,999787
RPP1B	1065,477192	-0,148448829	0,6581934	-0,22553983	0,821559	0,999787
RPP2A	2027,253312	-0,131188114	0,6624751	-0,19802724	0,843024	0,999787
RPP2B	2300,058858	-0,393918645	0,7140339	-0,55168063	0,581167	0,999787
RPR2	8,433172072	1,25012253	1,3039925	0,958688426	0,337716	0,999787
RPS1	4994,951156	0,598511194	0,7549446	0,792788282	0,427901	0,999787
RPS10	3601,657607	0,207237217	0,656384	0,315725552	0,752211	0,999787
RPS12	1738,242615	0,486631136	0,8139389	0,597871855	0,549925	0,999787
RPS13	1680,540445	0,712209688	0,7014152	1,015389551	0,30992	0,999787
RPS14B	4433,677767	-0,441317428	0,7167264	-0,61574043	0,538066	0,999787
RPS15	2807,047536	0,294058127	0,6548451	0,449049907	0,653396	0,999787
RPS16A	3100,317852	0,165404912	0,6526655	0,253429825	0,799936	0,999787
RPS17B	2784,87538	0,585089019	0,6862216	0,852623997	0,393868	0,999787
RPS18	2590,647071	0,237458534	0,6782668	0,350096048	0,726267	0,999787
RPS19A	2542,024386	0,030763265	0,6519022	0,047189998	0,962362	0,999787
RPS20	2952,916759	0,548955032	0,6733053	0,815313652	0,414893	0,999787
RPS21	4826,972271	0,484166312	0,653971	0,740348264	0,459089	0,999787
RPS21B	1377,071411	0,098638055	0,6560765	0,150345352	0,880492	0,999787
RPS22A	1649,145866	0,474748241	0,6668551	0,711921095	0,476514	0,999787
RPS23A	5216,371391	-0,111441027	0,6823258	-0,16332524	0,870262	0,999787
RPS24	6013,048821	-0,218905386	0,7228063	-0,30285484	0,762	0,999787
RPS25B	2782,463982	-0,059665644	0,6512905	-0,09161141	0,927007	0,999787
RPS26A	2355,845475	0,358229529	0,657136	0,545137592	0,585659	0,999787
RPS27	2776,614379	-0,129526862	0,6538096	-0,198111	0,842958	0,999787
RPS27A	19,95435952	-0,341256382	0,9593897	-0,35570153	0,722064	0,999787
RPS28B	1360,218583	-0,091846679	0,6632116	-0,13848775	0,889855	0,999787
RPS3	2136,17482	0,706168256	0,7876729	0,896524801	0,369973	0,999787
RPS30	877,9323473	0,202455089	0,753475	0,268695174	0,788164	0,999787
RPS4A	4859,943093	0,301754734	0,6511311	0,463431591	0,643055	0,999787
RPS5	4795,063875	0,232021382	0,667908	0,347385233	0,728302	0,999787
RPS6A	5331,084598	0,481217956	0,6693234	0,718961783	0,472164	0,999787
RPS7A	1925,470613	0,983050916	0,8785348	1,118966441	0,263154	0,999787
RPS8A	7290,076949	0,332991621	0,6569567	0,506869935	0,612246	0,999787
RPS9B	4182,780853	0,247170885	0,6739254	0,366762972	0,713796	0,999787
RPT1	293,7033967	-0,274042668	0,7191602	-0,38105926	0,703159	0,999787
RPT5	629,6739868	-0,414651507	0,6863409	-0,60414808	0,545745	0,999787
RPT6	541,7501619	0,42416221	0,671108	0,632032714	0,527366	0,999787
RRD1	50,64923252	-0,855623191	0,9046328	-0,94582378	0,344238	0,999787
RRP42	21,34357776	0,458704954	1,0981946	0,417690054	0,676174	0,999787
RRP6	287,2231283	0,721893945	0,7286387	0,990743395	0,321811	0,999787
RRP9	107,5662687	0,71233924	0,7846274	0,907869486	0,363947	0,999787

RSP5	234,9038954	0,14046586	0,7385969	0,19017933	0,849169	0,999787
RSR1	180,1624175	-0,486454776	0,7066278	-0,68841723	0,49119	0,999787
RTG1	93,64288464	-0,222937964	0,7366091	-0,30265439	0,762153	0,999787
RTG3	102,9929853	0,218578216	0,7241013	0,30186138	0,762758	0,999787
RTT101	16,13563945	-0,056809079	1,1493841	-0,04942567	0,96058	0,999787
RTT109	46,01391273	-0,340075131	0,8221606	-0,41363589	0,679141	0,999787
RUB1	72,04308781	0,413929055	0,7628028	0,542642294	0,587376	0,999787
RVB2	688,917281	-0,300183407	0,6698883	-0,44810967	0,654074	0,999787
RVS161	1257,422273	-0,976007094	0,8759305	-1,11425175	0,265171	0,999787
RVS167	489,9192553	-0,612008099	0,6872252	-0,89054955	0,373171	0,999787
RXT3	72,54869489	-0,124478834	0,7831081	-0,15895486	0,873704	0,999787
SAC1	47,55268312	-0,49752764	0,850867	-0,58473024	0,558729	0,999787
SAC3	129,9848095	0,436789619	0,8213376	0,531802804	0,594863	0,999787
SAC6	505,9076826	0,318506658	0,6753044	0,471649033	0,637177	0,999787
SAC7	257,0594207	-0,840402921	0,838074	-1,0027789	0,315968	0,999787
SAH1	1032,935452	-0,423365906	0,7139279	-0,59300933	0,553175	0,999787
SAL6	144,0878534	0,176040097	0,7763097	0,226765284	0,820606	0,999787
SAM2	881,6712081	0,551601448	0,7300446	0,755572244	0,449906	0,999787
SAM35	70,63982923	-0,991893347	0,8786555	-1,12887627	0,25895	0,999787
SAM37	25,62348428	-0,660737743	0,9086864	-0,72713507	0,467143	0,999787
SAM4	452,8181496	0,584615794	0,755307	0,774010807	0,438924	0,999787
SAM50	9,305376523	0,417857363	1,3730422	0,304329582	0,760877	0,999787
SAM51	69,33080152	-0,074851889	0,7969507	-0,09392286	0,92517	0,999787
SAP1	5,721678835	-0,629687889	1,435165	-0,43875645	0,660838	0,999787
SAP2	10,08003699	-0,03324639	1,2509446	-0,02657703	0,978797	0,999787
SAP8	6,026788864	0,323561483	1,4184164	0,228114593	0,819557	0,999787
SAP9	598,9989476	0,115918667	0,7577185	0,152983817	0,878411	0,999787
SAP98	17,97510503	-0,201594787	0,986105	-0,20443541	0,838013	0,999787
SAR1	152,3494788	-0,032545079	0,7230168	-0,0450129	0,964097	0,999787
SAS2	38,46260785	-0,725433483	0,8853418	-0,81938241	0,412568	0,999787
SAS3	64,33574375	-0,418244695	0,7578386	-0,5518915	0,581023	0,999787
SBA1	193,0421406	-0,357799451	0,6969615	-0,51337049	0,607692	0,999787
SCL1	233,2866508	-0,277685963	0,6986094	-0,39748389	0,691011	0,999787
SCO1	144,9764439	0,001746346	0,7093839	0,002461778	0,998036	0,999787
SCS7	265,9021006	-0,791426081	0,6968447	-1,13572803	0,25607	0,999787
SCT1	118,7659131	-0,323088116	0,7290582	-0,44315819	0,657651	0,999787
SCT2	89,55237111	-0,300561687	0,7621193	-0,39437614	0,693303	0,999787
SCW11	308,1648596	0,298675644	0,7572515	0,394420652	0,69327	0,999787
SCW4	6,658110859	-0,88435756	1,4710777	-0,60116306	0,547731	0,999787
SDC1	32,09237318	-0,267099087	0,8591525	-0,31088668	0,755887	0,999787
SDH1	197,7442342	0,09903642	0,7512348	0,131831507	0,895118	0,999787
SDH12	2677,58006	0,329174859	0,6567005	0,501255694	0,616191	0,999787
SDH2	1142,464894	-0,192929353	0,6673599	-0,2890934	0,77251	0,999787
SDH4	66,1726283	-0,200616899	0,7785666	-0,25767467	0,796658	0,999787
SDS22	137,3144461	0,139493159	0,7030345	0,198415819	0,84272	0,999787
SEC1	105,9107528	0,230461436	0,749224	0,307600171	0,758387	0,999787
SEC10	365,0966648	-0,155507713	0,6837889	-0,22742063	0,820097	0,999787
SEC12	199,2266113	-0,514498837	0,6885882	-0,74717926	0,454955	0,999787
SEC13	390,5481947	-0,669288968	0,7095823	-0,94321548	0,345571	0,999787
SEC14	257,8136377	-0,490095363	0,6884212	-0,71191211	0,476519	0,999787

SEC15	102,0759043	-0,252634371	0,72571	-0,34812027	0,72775	0,999787
SEC18	131,9626138	-0,386507248	0,8061319	-0,47945904	0,631612	0,999787
SEC2	103,8211962	-0,5709141	0,7318378	-0,7801102	0,435326	0,999787
SEC20	36,34925599	-0,904210913	0,8821635	-1,02499239	0,305367	0,999787
SEC21	436,2694866	0,181981116	0,7487243	0,243054908	0,807963	0,999787
SEC22	32,83998943	-0,378755527	1,0028945	-0,37766239	0,705681	0,999787
SEC23	569,5563723	-0,205106322	0,6791231	-0,30201639	0,76264	0,999787
SEC24	985,7228559	-0,444448621	0,6566645	-0,67682754	0,498515	0,999787
SEC26	509,1019654	-0,162987042	0,7473823	-0,21807721	0,827369	0,999787
SEC27	416,8497809	-0,226189323	0,6961071	-0,32493466	0,745231	0,999787
SEC3	195,852028	0,077385565	0,6883263	0,11242569	0,910486	0,999787
SEC34	193,1684713	0,069162596	0,6884931	0,100455026	0,919983	0,999787
SEC4	369,2278424	-0,227990981	0,7011577	-0,32516362	0,745057	0,999787
SEC5	38,85560488	-0,840264942	0,8364644	-1,00454357	0,315117	0,999787
SEC6	491,7976335	-0,928259088	0,8210929	-1,13051649	0,258259	0,999787
SEC61	845,0474503	0,022304772	0,6684698	0,033366912	0,973382	0,999787
SEC62	202,0865365	-0,305082004	0,7409729	-0,41173167	0,680536	0,999787
SEC65	151,6750127	0,08314487	0,6976869	0,11917218	0,905139	0,999787
SEC7	492,6172375	0,241055448	0,701863	0,343450867	0,731259	0,999787
SEC72	56,42206468	0,401405109	0,8578248	0,467933677	0,639832	0,999787
SEC8	119,6477109	-0,586709014	0,7992767	-0,73404991	0,462918	0,999787
SEC9	81,86720284	-0,237371702	0,7857376	-0,30210046	0,762575	0,999787
SEF1	85,05505191	0,474615534	0,812498	0,584143622	0,559124	0,999787
SEF2	14,33449794	-0,937048015	1,1212275	-0,83573405	0,403305	0,999787
SEH1	126,4846918	-0,875965614	0,8399696	-1,04285393	0,297016	0,999787
SEN1	272,7852587	0,417800308	0,702418	0,594802963	0,551975	0,999787
SEP7	194,349045	0,54592678	0,7224162	0,755695617	0,449832	0,999787
SER1	522,2700037	0,378396881	0,7076983	0,534686726	0,592867	0,999787
SER33	725,2625338	0,535834812	0,7808538	0,686216538	0,492577	0,999787
SES1	865,4306013	0,154278916	0,7223208	0,213587804	0,830869	0,999787
SET1	183,4850175	0,172050904	0,6950568	0,247535016	0,804494	0,999787
SET2	127,1335989	0,262873236	0,7452629	0,352725515	0,724294	0,999787
SET3	238,0159921	-0,025613241	0,687042	-0,03728046	0,970261	0,999787
SFH5	86,78844441	0,469138796	0,7677351	0,611068615	0,541154	0,999787
SFI1	90,26741227	-0,612338494	0,7640784	-0,80140794	0,422896	0,999787
SFL1	257,7722108	0,401693043	0,7152246	0,561632004	0,574367	0,999787
SFL2	116,4041554	-0,81414751	0,729205	-1,11648644	0,264214	0,999787
SFP1	493,4396019	-0,224477127	0,6860593	-0,32719785	0,743518	0,999787
SFT2	33,8222278	-0,668325257	0,9312212	-0,71768691	0,47295	0,999787
SGE1	42,05167145	0,380754413	0,9088169	0,418956112	0,675248	0,999787
SGO1	40,98969454	-0,280952687	0,8447889	-0,33257148	0,739458	0,999787
SGS1	375,311208	0,190205037	0,6708163	0,283542679	0,776761	0,999787
SGT1	435,5521676	0,412266019	0,6681427	0,61703289	0,537213	0,999787
SGT2	663,0550462	0,08662642	0,6776134	0,127840476	0,898275	0,999787
SHA3	1241,150872	-0,591002011	1,0871007	-0,54364976	0,586683	0,999787
SHE3	92,17306792	0,064941127	0,8307353	0,078173069	0,93769	0,999787
SHM1	569,8919526	0,675379764	0,7990083	0,845272575	0,397959	0,999787
SHM2	1642,016645	0,34546542	0,7313389	0,472373942	0,63666	0,999787
SHP1	294,3510809	0,009945681	0,677198	0,014686519	0,988282	0,999787
SHY1	75,30468171	0,757066269	0,7586639	0,997894214	0,318331	0,999787

SIK1	1852,998918	0,002510649	0,6632121	0,00378559	0,99698	0,999787
SIM1	986,6111154	0,536153822	0,7041893	0,761377456	0,446432	0,999787
SIN3	565,6452359	-0,208826573	0,7394022	-0,28242623	0,777617	0,999787
SIR2	9,763981931	0,030784309	1,2348075	0,024930451	0,98011	0,999787
SIS1	548,9050588	-0,445406263	0,6901433	-0,64538232	0,518679	0,999787
SIT1	24,76328238	-0,762021499	0,9425258	-0,80848873	0,418809	0,999787
SIT4	108,8016363	-0,71470969	0,7457705	-0,9583507	0,337886	0,999787
SIW14	28,72534669	-0,926454871	0,8779593	-1,05523664	0,291317	0,999787
SIZ1	262,7774368	-0,278843695	0,695639	-0,40084541	0,688534	0,999787
SKI2	493,0427999	0,436806252	0,6730763	0,648969898	0,516358	0,999787
SKI3	272,247418	0,368006058	0,7378512	0,49875377	0,617953	0,999787
SKI8	195,3498031	-0,367699472	0,7907783	-0,46498429	0,641943	0,999787
SKN2	55,76004372	-0,651679934	0,904694	-0,72033191	0,471321	0,999787
SKN7	69,74471232	0,001267314	0,7789061	0,001627043	0,998702	0,999787
SKO1	179,7575224	0,013623786	0,7626165	0,01786453	0,985747	0,999787
SKP1	145,2377011	-0,306069517	0,7024427	-0,43572169	0,663039	0,999787
SLA1	483,7231444	-0,754597539	0,6695714	-1,12698588	0,259748	0,999787
SLA2	209,3180382	-0,173970971	0,7355349	-0,23652307	0,813027	0,999787
SLC1	57,92921915	-0,35271077	0,7841387	-0,44980662	0,65285	0,999787
SLD1	52,66774438	-0,138941338	0,7815143	-0,17778476	0,858892	0,999787
SLD5	8,117270423	1,021192746	1,3050027	0,782521535	0,433908	0,999787
SLM2	322,7459166	-0,376599735	0,6828526	-0,55150957	0,581284	0,999787
SLN1	105,211006	0,446472485	0,8082334	0,552405389	0,580671	0,999787
SLP2	75,16537744	0,107290564	0,8132831	0,131922779	0,895045	0,999787
SLU7	24,65750328	0,426687845	0,9112386	0,468250405	0,639606	0,999787
SLY41	42,3087607	-0,815938336	0,8163188	-0,99953395	0,317536	0,999787
SMC1	109,3132105	0,332721663	0,8554918	0,388924444	0,697332	0,999787
SMC2	59,69510586	-0,32223607	0,9898003	-0,32555666	0,74476	0,999787
SMC3	33,49164289	0,36655421	1,0739851	0,341302867	0,732876	0,999787
SMC4	310,5696417	0,098382606	0,766848	0,128294799	0,897916	0,999787
SMC5	123,4442895	0,84994437	1,0063767	0,844558893	0,398357	0,999787
SMC6	196,4736883	-0,078837533	0,709502	-0,11111671	0,911524	0,999787
SMD2	25,80979498	0,475471762	0,9026376	0,526758233	0,598361	0,999787
SMD3	63,44173822	-0,501841382	0,7874876	-0,63726895	0,52395	0,999787
SMF12	124,482095	-0,001864651	0,710761	-0,00262346	0,997907	0,999787
SMF3	127,8689784	-0,495820324	0,7081522	-0,70016064	0,483827	0,999787
SMI1B	71,3045263	-0,816699052	0,8920212	-0,91556017	0,359898	0,999787
SMP2	421,8250476	0,036877242	0,7538794	0,048916635	0,960986	0,999787
SMX4	68,53072495	-0,222180017	0,7970502	-0,27875285	0,780434	0,999787
SNF1	379,6645417	-0,451811783	0,6714773	-0,67286232	0,501035	0,999787
SNF2	383,6822592	-0,188153031	0,7889128	-0,23849661	0,811496	0,999787
SNF4	228,0428809	-0,286819208	0,694318	-0,41309485	0,679537	0,999787
SNF5	143,7794045	-0,115567035	0,7350072	-0,15723251	0,875062	0,999787
SNF7	276,8986954	-0,431534601	0,7305069	-0,59073308	0,554699	0,999787
SNG3	29,11585066	-0,575873448	0,9031488	-0,63762855	0,523716	0,999787
SNG4	30,33356441	-0,713518961	0,8812931	-0,8096273	0,418154	0,999787
SNL1	49,6339834	-0,22944028	0,7929966	-0,28933324	0,772326	0,999787
SNM1	24,92704014	0,717657423	0,9142119	0,78500115	0,432453	0,999787
SNO1	128,0667238	0,813627463	0,7580176	1,073362217	0,283109	0,999787
SNP3	34,42294906	-0,218039817	0,8502722	-0,2564353	0,797615	0,999787

SNR52	7,044512423	-0,814392876	1,419595	-0,57367974	0,566185	0,999787
SNT1	544,8409478	-0,798185842	0,7583131	-1,05258084	0,292533	0,999787
SNU114	48,98157117	0,109967087	0,8202879	0,134059136	0,893356	0,999787
SNX4	41,20096358	-0,096063298	0,8316429	-0,11551027	0,908041	0,999787
SNZ1	821,4164597	0,729978661	0,7125859	1,024408014	0,305643	0,999787
SOD2	241,7598749	0,641330845	0,6923014	0,926375143	0,354251	0,999787
SOG2	210,5249278	-0,149302502	0,6858375	-0,21769369	0,827668	0,999787
SOK1	210,4267124	-0,389687239	0,7268751	-0,53611308	0,59188	0,999787
SOL1	49,49680967	0,751786023	0,8081769	0,930224604	0,352255	0,999787
SOL3	283,7408848	-0,796841672	0,7732753	-1,03047603	0,302787	0,999787
SPA2	785,5351808	-0,04315414	0,6593806	-0,06544648	0,947819	0,999787
SPC2	29,05999878	-0,679553971	0,89497	-0,75930362	0,447671	0,999787
SPC3	51,93348894	-0,219017427	0,7935936	-0,27598185	0,782562	0,999787
SPC34	22,89547282	0,432476986	0,9432957	0,458474456	0,646612	0,999787
SPC98	26,49213799	-0,200958834	0,9412319	-0,21350619	0,830932	0,999787
SPE1	187,3961457	-0,072754463	0,8023648	-0,09067504	0,927751	0,999787
SPE2	59,89179607	0,447824562	0,9751569	0,459233363	0,646067	0,999787
SPE3	284,0930983	0,516105828	0,7666733	0,673175667	0,500836	0,999787
SPF1	270,1565321	0,284104687	0,8498465	0,334301176	0,738152	0,999787
SPO7	52,4257095	0,381473124	0,8560402	0,445625256	0,655868	0,999787
SPO72	101,1896211	-0,051668673	0,7443058	-0,06941861	0,944656	0,999787
SPO75	46,67161359	0,308220229	0,8222629	0,374843902	0,707777	0,999787
SPP1	47,16425927	-0,164769153	0,8104868	-0,20329653	0,838903	0,999787
SPT10	34,19267666	-0,147578389	0,8441127	-0,17483256	0,861211	0,999787
SPT14	28,29571305	-0,419797799	0,8825901	-0,47564297	0,634329	0,999787
SPT20	97,75898414	0,017886412	0,7553189	0,02368061	0,981107	0,999787
SPT23	217,8070172	-0,418282608	0,8196365	-0,51032699	0,609822	0,999787
SPT3	52,06105051	0,102558127	0,7822547	0,1311058	0,895692	0,999787
SPT6	2351,222442	0,153719632	0,680638	0,22584638	0,821321	0,999787
SRB1	3018,487022	-0,647744964	0,7179099	-0,90226497	0,366916	0,999787
SRB8	54,43588784	-0,080047622	0,8171375	-0,09796102	0,921963	0,999787
SRB9	160,8124247	-0,687174404	0,7000377	-0,98162488	0,326285	0,999787
SRO77	305,5404459	-0,521690944	0,7285361	-0,71608111	0,473941	0,999787
SRP101	214,2043404	-0,134660695	0,7054156	-0,19089556	0,848607	0,999787
SRP54	451,4385693	-0,341062321	0,6716292	-0,50781342	0,611584	0,999787
SRR1	41,65849862	0,601862358	1,0508254	0,572752015	0,566813	0,999787
SRT1	12,97987624	-0,407104274	1,0871366	-0,3744739	0,708052	0,999787
SRV2	413,482221	0,028946117	0,6731589	0,043000424	0,965701	0,999787
SSA2	4928,609327	0,042653881	0,7491232	0,056938407	0,954594	0,999787
SSB1	11250,63134	0,242108758	0,6875472	0,352134026	0,724738	0,999787
SSC1	3005,618537	0,654186683	0,8033578	0,814315495	0,415464	0,999787
SSD1	881,7158004	-0,750612735	0,6755677	-1,11108447	0,266532	0,999787
SSF1	199,2619378	0,808578423	0,7585332	1,065976257	0,286434	0,999787
SSH1	129,4113244	0,352003666	0,7405993	0,47529572	0,634576	0,999787
SSK1	181,5424157	0,199846195	0,7006998	0,28520943	0,775484	0,999787
SSK2	404,6902637	0,335727603	0,6752496	0,497190347	0,619055	0,999787
SSN3	41,16998106	-0,144595191	0,8615125	-0,16783877	0,86671	0,999787
SSN8	25,70042525	-0,057824853	0,8955581	-0,06456851	0,948518	0,999787
SSO2	153,3499041	-0,166363847	0,7127112	-0,23342392	0,815432	0,999787
SSR1	191,8950658	-0,045904987	0,7288288	-0,06298459	0,949779	0,999787

SST2	12,8527902	-0,358729631	1,0891496	-0,32936671	0,741879	0,999787
SSU1	29,86561839	-0,049598751	0,8811839	-0,05628649	0,955114	0,999787
SSU72	52,71208671	0,117965233	0,7808985	0,15106346	0,879926	0,999787
SSY5	101,0219845	-0,370824296	0,7226655	-0,51313411	0,607858	0,999787
STB3	57,95427124	-0,875523973	0,7777734	-1,12568001	0,260301	0,999787
STB5	64,03616475	-0,153010187	0,8066389	-0,18968859	0,849553	0,999787
STE11	81,56749989	-0,35441335	0,7382035	-0,48010249	0,631155	0,999787
STE13	86,63931029	0,455085152	0,736658	0,617769899	0,536727	0,999787
STE23	93,1279154	0,200227939	0,8890188	0,225223527	0,821805	0,999787
STE24	203,1593655	-0,219181727	0,6895418	-0,31786576	0,750587	0,999787
STE3	13,35001366	0,08507324	1,0998224	0,077351801	0,938344	0,999787
STE4	8,403641711	-0,064765568	1,2668798	-0,05112211	0,959228	0,999787
STE50	69,74588104	-0,340441422	0,8273441	-0,4114871	0,680715	0,999787
STI1	312,7408921	0,21048425	1,0226521	0,205821953	0,83693	0,999787
STN1	8,113820356	-0,83575082	1,3083866	-0,63876445	0,522976	0,999787
STP1	113,6817223	-0,850928022	0,7907372	-1,0761199	0,281874	0,999787
STP2	329,5227696	-0,600808806	0,7136136	-0,84192456	0,39983	0,999787
STP4	148,163132	0,318358682	1,2186456	0,261239763	0,793908	0,999787
STR2	102,9984636	0,153204574	0,751264	0,203929073	0,838409	0,999787
STT4	206,0568251	-0,105983617	0,698972	-0,15162784	0,87948	0,999787
STV1	361,5448875	-0,716212801	0,7309327	-0,9798615	0,327154	0,999787
SUA71	384,3371585	0,469872189	0,7151643	0,657012896	0,511173	0,999787
SUB2	786,6182372	0,343566992	0,7029137	0,48877551	0,625001	0,999787
SUI1	224,5417086	-0,066933635	0,7580533	-0,08829674	0,929641	0,999787
SUI2	566,510639	0,218190641	0,6659871	0,327619919	0,743199	0,999787
SUI3	967,363829	-0,520485899	0,7071594	-0,73602344	0,461716	0,999787
SUL2	14,19999899	0,944614429	1,1172182	0,845505798	0,397828	0,999787
SUN41	547,4740229	0,165597048	0,6730283	0,246047658	0,805645	0,999787
SUP35	1107,267981	0,52740154	0,7625936	0,691589243	0,489195	0,999787
SUR2	147,2000273	-0,689120877	0,7003412	-0,98397872	0,325126	0,999787
SUR7	310,5192315	-0,58397072	0,7104135	-0,82201523	0,411068	0,999787
SUV3	69,12197084	0,876774636	1,1404074	0,768825791	0,441997	0,999787
SVF1	230,7835788	0,37769296	0,7418576	0,509117841	0,61067	0,999787
SWD1	93,69591309	0,259242434	0,7504682	0,345440918	0,729763	0,999787
SWD2	86,37584078	-0,250773115	0,743137	-0,33745206	0,735776	0,999787
SWD3	20,19749873	-0,04430296	0,9570359	-0,04629185	0,963078	0,999787
SWE1	185,2227787	-0,066737161	0,8069266	-0,08270537	0,934086	0,999787
SWI1	212,9069273	-0,417503872	0,7110352	-0,58717752	0,557084	0,999787
SWI4	216,8075116	0,119330137	0,7781331	0,153354405	0,878119	0,999787
SWI6	157,1174919	-0,015204448	0,6996643	-0,02173106	0,982662	0,999787
SWR1	416,0982227	0,35908887	0,6757291	0,531409546	0,595135	0,999787
SYG1	47,84968208	-0,914976483	0,8352725	-1,09542267	0,273332	0,999787
SYS3	243,5722902	-0,083854995	0,6800566	-0,1233059	0,901865	0,999787
TAC1	62,76561369	0,768491418	0,8593352	0,894285942	0,371169	0,999787
TAF14	201,9284629	0,044990967	0,7363424	0,061100603	0,951279	0,999787
TAF19	53,34205145	-0,404449549	0,8362984	-0,48361871	0,628656	0,999787
TAF4	171,8221861	-0,398057519	0,7554529	-0,52691242	0,598254	0,999787
TAF60	70,78029834	0,060855232	0,8737565	0,069647814	0,944474	0,999787
TAL1	1642,947562	0,290160439	0,6552722	0,44280901	0,657904	0,999787
TBP1	177,7051773	-0,00124281	0,6922486	-0,00179532	0,998568	0,999787

TCC1	395,6338324	-0,560794626	0,7412367	-0,75656614	0,44931	0,999787
TCP1	529,7219848	0,570421963	0,6980655	0,817146746	0,413845	0,999787
TEA1	50,27623758	0,115135673	0,8009875	0,143742157	0,885704	0,999787
TEF1	2313,594666	-0,368852528	0,6759928	-0,54564563	0,58531	0,999787
TEF2	1698,926254	0,052168876	0,6790842	0,076822399	0,938765	0,999787
TEL1	154,1903617	0,730467122	0,713443	1,023861971	0,3059	0,999787
TEM1	14,75075892	-0,501203217	1,1236323	-0,44605626	0,655557	0,999787
TERT	31,84176239	-0,432611565	0,9009818	-0,48015571	0,631117	0,999787
TES1	16,91963361	0,298143957	1,0022152	0,297484981	0,766096	0,999787
TFA1	239,5576675	-0,532077587	0,7983545	-0,6664678	0,505112	0,999787
TFB3	17,6905794	0,551974343	1,0115232	0,545686296	0,585282	0,999787
TFC4	105,5595964	-0,681092138	0,7367796	-0,92441777	0,355269	0,999787
TFG1	939,0042544	-0,70594905	0,7858424	-0,8983341	0,369007	0,999787
TFP1	451,1016116	-0,159663937	0,7524002	-0,21220612	0,831946	0,999787
TGL99	18,91603988	-0,444899459	0,9947534	-0,44724597	0,654697	0,999787
THI20	101,6851795	0,836895791	0,7875422	1,062667925	0,287933	0,999787
THI6	40,55998954	0,225702844	0,8401501	0,268645847	0,788202	0,999787
THR1	84,30401675	-0,369703785	0,9437632	-0,39173363	0,695255	0,999787
THR4	375,2074052	0,30769807	0,7259309	0,42386689	0,671663	0,999787
THS1	1886,717795	0,489661089	0,6592458	0,742759567	0,457627	0,999787
TIF	2363,123566	0,180958716	0,718319	0,25191971	0,801103	0,999787
TIF11	1850,517817	-0,427726624	0,795742	-0,53751924	0,590909	0,999787
TIF3	1047,106121	0,080433273	0,6581289	0,122215072	0,902729	0,999787
TIF34	605,5057419	0,154429554	0,7028466	0,219720133	0,826089	0,999787
TIF35	1058,883951	-0,764636303	0,7392825	-1,03429509	0,300998	0,999787
TIF4631	2633,70169	-0,513870122	0,6739992	-0,76241949	0,44581	0,999787
TIF5	220,9591435	0,380090777	0,925885	0,410516187	0,681427	0,999787
TIM10	79,67287448	0,377551043	0,9970176	0,378680408	0,704925	0,999787
TIM12	20,10429246	0,523664774	0,9793119	0,534727252	0,592838	0,999787
TIM13	52,87366977	0,795664508	0,802149	0,991916156	0,321238	0,999787
TIM17	99,84221889	-0,002162672	0,7881079	-0,00274413	0,997811	0,999787
TIM21	76,82953486	0,183217683	0,7731315	0,236981281	0,812671	0,999787
TIM22	154,1679034	-0,572735073	0,7107584	-0,80580842	0,420353	0,999787
TIM23	172,4064732	0,821737791	0,9492984	0,86562645	0,386695	0,999787
TIM44	828,0712302	0,463549151	0,671333	0,690490677	0,489886	0,999787
TIM50	642,3108084	0,806385623	0,8536331	0,94465128	0,344837	0,999787
TIM54	467,142125	0,342716884	0,7062185	0,485284497	0,627475	0,999787
TIM8	100,0563456	-0,508655226	0,7788775	-0,65306188	0,513716	0,999787
TIM9	46,03844118	0,044481397	0,9293771	0,047861518	0,961827	0,999787
TIP1	66,69545075	-0,387908996	0,8227829	-0,47145975	0,637312	0,999787
TIP120	139,3478787	-0,160170991	0,7175433	-0,22322136	0,823363	0,999787
TIP20	79,07392391	-0,129000607	0,7938768	-0,16249448	0,870916	0,999787
TIP41	36,63680774	0,067216033	0,8683018	0,077410907	0,938297	0,999787
TLO10	54,39648298	-0,863193494	0,8962703	-0,96309505	0,3355	0,999787
TLO13	216,9404645	-0,285661363	0,8145986	-0,35067746	0,72583	0,999787
TLO16	93,97338057	-0,377231482	0,8396632	-0,44926521	0,65324	0,999787
TLO34	37,51821125	-0,754067872	0,9317514	-0,80930158	0,418342	0,999787
TLO4	134,6976431	-0,702070053	0,8707932	-0,80624202	0,420103	0,999787
TLO8	165,5904417	-0,781355535	0,8797829	-0,88812317	0,374475	0,999787
TLO9	199,3408601	-0,758073265	0,7743556	-0,97897306	0,327593	0,999787

TMA19	1901,841502	0,182064697	0,6529651	0,278827597	0,780377	0,999787
TOA2	43,97272847	0,625960555	0,8126561	0,770265038	0,441143	0,999787
TOM20	272,7153889	0,799366051	0,7798594	1,025013055	0,305357	0,999787
TOM22	101,4551747	0,05503618	0,9087794	0,060560549	0,951709	0,999787
TOM40	1230,669254	0,119624962	0,6581093	0,181770653	0,855763	0,999787
TOM6	119,3354027	0,184497962	0,7119219	0,259154767	0,795516	0,999787
TOM7	96,73148547	0,500490634	0,9277613	0,539460575	0,589569	0,999787
TOM70	225,0043764	0,045871615	0,8804661	0,052099242	0,95845	0,999787
TOP1	568,0526016	-0,241106434	0,7602318	-0,31714856	0,751131	0,999787
TOP2	417,2600982	0,627395012	0,7733594	0,811259317	0,417217	0,999787
TOR1	482,5030587	-0,165018432	0,6730044	-0,24519665	0,806304	0,999787
TOS1	638,8171374	0,565345025	0,7341788	0,770037249	0,441278	0,999787
TOS4	176,7763826	-0,568608036	0,7259647	-0,78324473	0,433483	0,999787
TPD3	106,4488342	-0,303111587	0,8181254	-0,37049529	0,711013	0,999787
TPK1	69,09368172	-0,032717826	0,8629965	-0,03791189	0,969758	0,999787
TPM2	1039,781033	-0,681943312	0,7004492	-0,97357993	0,330265	0,999787
TPO2	27,87092891	-0,804863525	1,0405885	-0,77346957	0,439245	0,999787
TPO4	181,5202954	-0,230689359	0,9806139	-0,23524995	0,814015	0,999787
TPS1	95,21844029	-0,590774382	0,736264	-0,80239475	0,422325	0,999787
TPT1	66,43086176	0,464574255	0,7633494	0,608599785	0,54279	0,999787
TRA1	449,6384663	0,498877453	0,8490965	0,587539174	0,556842	0,999787
TRK1	166,7584742	0,595036871	0,732026	0,812862982	0,416297	0,999787
TRM12	16,77789955	0,710173919	1,0178948	0,69768894	0,485372	0,999787
TRM9	241,2399124	-0,063971024	0,7576786	-0,08443029	0,932714	0,999787
TRP1	161,392644	-0,753746162	0,8037294	-0,93781084	0,348342	0,999787
TRP2	353,536761	0,581779141	0,7067876	0,82313146	0,410433	0,999787
TRP3	92,37617797	0,569105399	1,1706631	0,486139346	0,626868	0,999787
TRP4	172,7496684	0,51326231	0,7850245	0,65381694	0,51323	0,999787
TRP5	318,5493335	0,568155114	0,7575533	0,749987002	0,453263	0,999787
TRP99	174,1264897	-0,880763115	0,8607757	-1,02322026	0,306204	0,999787
TRR1	256,7597772	0,530719604	0,7162643	0,740954995	0,458721	0,999787
TRS20	13,26461972	-0,252701513	1,1196903	-0,22568875	0,821444	0,999787
TRS33	50,94918146	-0,418486842	0,7946023	-0,52666204	0,598428	0,999787
TRX1	438,1891629	0,002396514	0,7299478	0,00328313	0,99738	0,999787
TRX2	6,88740187	0,416981158	1,4300524	0,291584538	0,770604	0,999787
TRY2	81,61432276	-0,018960062	0,7695256	-0,02463864	0,980343	0,999787
TRY3	109,3040175	0,477099363	0,7966747	0,598863462	0,549264	0,999787
TSC11	39,58639856	-0,850828374	0,818824	-1,03908581	0,298765	0,999787
TSC2	32,83844368	0,026468096	0,92115	0,028733752	0,977077	0,999787
TSM1	436,9350044	-0,363719087	0,7292583	-0,49875209	0,617954	0,999787
TTR1	271,7049667	0,535914902	0,7827095	0,684691972	0,493538	0,999787
TUB1	563,9092285	0,517716921	0,7258441	0,713261909	0,475684	0,999787
TUB2	446,0762281	-0,001977576	0,8494946	-0,00232794	0,998143	0,999787
TUB4	59,05165621	-0,151413766	0,7738122	-0,1956725	0,844867	0,999787
TUP1	3228,59755	-0,507382599	0,7738433	-0,65566583	0,512039	0,999787
TUS1	296,5642601	-0,514966294	0,6830217	-0,75395303	0,450877	0,999787
TVP18	68,75252099	0,207005059	0,7889756	0,262371933	0,793035	0,999787
TYE7	1838,70222	-0,468597438	0,9980388	-0,46951826	0,638699	0,999787
TYR1	106,6568562	0,419414595	0,7263494	0,57742814	0,56365	0,999787
TYS1	605,4469619	0,083817959	0,6631442	0,126394769	0,899419	0,999787

UAP1	54,88482934	0,14574578	0,906807	0,160724146	0,872311	0,999787
UBA1	687,7985203	0,04952894	0,7237197	0,068436638	0,945438	0,999787
UBA2	121,8588859	0,218508576	0,7413722	0,294735308	0,768196	0,999787
UBC15	74,58691647	-0,884350534	0,8445294	-1,04715187	0,29503	0,999787
UBC4	511,4463258	-0,405385134	0,6647637	-0,60981838	0,541982	0,999787
UBC8	109,3619001	-0,855072495	0,7703615	-1,1099626	0,267015	0,999787
UBI3	6537,205778	-0,109458393	0,710108	-0,1541433	0,877497	0,999787
UBI4	756,9371278	-0,68434337	0,6679597	-1,02452787	0,305586	0,999787
UBP1	181,3237246	0,087376892	0,7097263	0,123113511	0,902017	0,999787
UBP13	238,3558425	-0,70267394	0,691344	-1,01638831	0,309445	0,999787
UBP6	489,0301631	0,311408299	0,6651624	0,468168857	0,639664	0,999787
UBR1	442,1987183	0,259279694	0,6662351	0,389171495	0,697149	0,999787
UEC1	16,10546268	-0,718212334	1,0490988	-0,68459932	0,493597	0,999787
UGA1	331,8959033	0,213213182	0,684031	0,311701036	0,755268	0,999787
UGA2	92,15324856	-0,771551253	0,8358409	-0,92308386	0,355964	0,999787
UGA3	119,227985	-0,192855089	0,7137691	-0,27019256	0,787012	0,999787
UGA32	32,15490736	0,256598615	0,8965459	0,286207996	0,774719	0,999787
UGA33	25,90975489	0,257804921	0,9858343	0,261509384	0,7937	0,999787
UGA4	7,62866388	-0,060529169	1,3122778	-0,04612527	0,96321	0,999787
UGP1	390,2288197	-0,426462756	0,7314411	-0,58304456	0,559863	0,999787
UGT51C1	109,2003195	0,088974422	0,7182039	0,123884623	0,901407	0,999787
ULP1	36,0809874	0,079263612	0,8688131	0,091232065	0,927308	0,999787
ULP2	32,15218099	-0,608841955	0,8779551	-0,69347733	0,48801	0,999787
ULP3	84,44686615	-0,013665437	0,7461703	-0,0183141	0,985388	0,999787
UME1	172,524693	0,053370563	0,6935794	0,076949465	0,938664	0,999787
UPC2	234,8669323	-0,694180196	0,7149237	-0,97098505	0,331556	0,999787
URA1	483,4535447	-0,658560824	0,6897379	-0,95479872	0,339679	0,999787
URA2	1791,929535	0,022695167	0,9943565	0,022823975	0,981791	0,999787
URA4	108,8203424	0,231258709	0,7997235	0,289173316	0,772449	0,999787
URA5	85,08339948	0,599987284	0,7642804	0,785035592	0,432433	0,999787
URA6	247,9853893	0,621384623	0,6814314	0,911881452	0,361831	0,999787
URE2	73,3675502	-0,041861292	0,7500235	-0,05581331	0,955491	0,999787
URK1	49,71557944	0,384724828	0,8537097	0,450650665	0,652241	0,999787
USO1	232,6561929	0,190383683	0,9346311	0,203699276	0,838589	0,999787
USO5	21,3339384	-0,693442136	1,0111022	-0,68582795	0,492822	0,999787
USO6	341,3065576	0,206913522	0,6746265	0,306708242	0,759065	0,999787
UTP15	69,52495866	1,089143601	1,0383357	1,048932076	0,294209	0,999787
UTP20	227,7447963	0,646968849	0,7900583	0,818887447	0,412851	0,999787
UTP22	447,2313441	0,634866869	1,040936	0,609900005	0,541928	0,999787
UTP4	182,3848768	0,992882275	1,0717693	0,926395476	0,35424	0,999787
UTR2	137,1626712	0,809717613	0,735362	1,10111426	0,270847	0,999787
VAC7	53,55085762	0,598951033	0,8703754	0,688152554	0,491357	0,999787
VAC8	186,6403198	-0,659794392	0,7119259	-0,92677394	0,354044	0,999787
VAM3	29,26013545	0,22959268	0,887698	0,25863827	0,795914	0,999787
VAN1	189,5597029	0,366847565	0,6955766	0,527400643	0,597915	0,999787
VAS1	731,629911	0,049034051	0,7939462	0,061759916	0,950754	0,999787
VCX1	142,2255671	0,289192171	0,716282	0,403740658	0,686403	0,999787
VID21	368,607462	-0,332638304	0,7126653	-0,46675248	0,640677	0,999787
VID27	1159,672396	0,09140331	0,7289041	0,125398261	0,900208	0,999787
VMA10	322,8942438	-0,26730012	0,6722372	-0,39762768	0,690905	0,999787

VMA11	107,976619	-0,792673608	0,7430365	-1,06680304	0,286061	0,999787
VMA13	96,7347434	-0,172905244	0,7569754	-0,22841595	0,819323	0,999787
VMA2	556,0162713	-0,177717334	0,6794081	-0,2615767	0,793648	0,999787
VMA4	234,4213939	0,303620022	0,7247916	0,418906669	0,675284	0,999787
VMA5	37,85945287	-0,524533874	0,9095194	-0,57671545	0,564132	0,999787
VMA7	58,45775419	-0,308907436	0,7730698	-0,39958542	0,689462	0,999787
VMA8	223,1552487	-0,754318493	0,7230896	-1,04318812	0,296861	0,999787
VPH1	216,9405187	-0,251657707	0,7096338	-0,35463036	0,722867	0,999787
VPS1	454,4196812	0,312799869	0,7284578	0,429400148	0,667632	0,999787
VPS11	57,89043123	-0,549147679	0,8291539	-0,66229882	0,50778	0,999787
VPS13	295,2958889	-0,025340653	0,6954546	-0,03643754	0,970933	0,999787
VPS15	33,27234483	-0,506368411	0,8670475	-0,5840146	0,55921	0,999787
VPS16	80,05439953	-0,492161046	0,7870274	-0,62534168	0,531747	0,999787
VPS17	103,3804572	-0,105181499	0,7215295	-0,14577574	0,884098	0,999787
VPS2	181,6737729	-0,652836217	0,7729191	-0,84463716	0,398313	0,999787
VPS20	52,78880046	-0,529311069	0,814732	-0,64967507	0,515902	0,999787
VPS21	422,2968287	-0,903836344	0,8543809	-1,05788457	0,290108	0,999787
VPS22	8,168790364	-0,52019185	1,2560501	-0,41414896	0,678765	0,999787
VPS23	42,11139144	0,155198056	0,850586	0,182460161	0,855222	0,999787
VPS24	49,93814712	-0,50820303	0,7874295	-0,64539493	0,518671	0,999787
VPS27	188,2145737	-0,51504358	0,6896038	-0,7468688	0,455143	0,999787
VPS28	30,36407024	-0,509032515	0,8607249	-0,59139981	0,554253	0,999787
VPS33	44,76317178	0,0860476	0,8139897	0,105710915	0,915812	0,999787
VPS34	46,05246042	-0,423342101	0,7972993	-0,53097012	0,595439	0,999787
VPS35	135,9147978	-0,644760649	0,7052258	-0,91426127	0,36058	0,999787
VPS36	67,0602923	-0,563072153	0,7546535	-0,74613334	0,455587	0,999787
VPS4	174,177691	-0,350805871	0,7227506	-0,4853761	0,62741	0,999787
VPS41	65,97043525	-0,18194779	0,7610606	-0,23907135	0,81105	0,999787
VPS51	40,81907653	-0,398960084	0,8487782	-0,47004044	0,638326	0,999787
VPS52	66,13656097	0,095275709	0,7864571	0,12114547	0,903576	0,999787
VPS53	179,0358816	-0,39275759	0,6986008	-0,56220607	0,573976	0,999787
VPS8	42,87269094	0,276395626	0,8158721	0,338773235	0,734781	0,999787
VRG4	66,98901598	0,354076505	0,8532151	0,41499088	0,678149	0,999787
VRP1	261,6726323	-0,593897987	0,7290637	-0,8146037	0,415299	0,999787
VTC3	464,0680901	-0,404672194	0,7507836	-0,53899981	0,589887	0,999787
VTI1	54,59282159	0,196291454	0,8521879	0,230338222	0,817829	0,999787
WBP1	186,8229339	0,383172282	0,6950585	0,551280648	0,581441	0,999787
WHI3	99,41760101	-0,454340238	0,7318617	-0,6208007	0,534731	0,999787
WOR1	7,79340996	-0,226208169	1,3492132	-0,16765932	0,866851	0,999787
WOR2	46,6240448	-0,036393021	0,8905696	-0,04086488	0,967404	0,999787
WOR4	240,7985394	-0,39221007	0,7122854	-0,55063615	0,581883	0,999787
WRS1	303,8245482	0,742244524	0,8141949	0,911629983	0,361964	0,999787
WSC1	36,40066933	-0,158320915	0,8651206	-0,18300445	0,854795	0,999787
WSC2	72,05807875	-0,438922937	0,7612566	-0,57657688	0,564225	0,999787
WSC4	77,20712045	-0,476811331	0,757497	-0,62945642	0,52905	0,999787
XKS1	83,87064499	-0,800449152	0,7540853	-1,06148363	0,28847	0,999787
XOG1	24,81076435	-0,854436816	0,9090912	-0,93988017	0,347279	0,999787
XUT1	9,212670342	-0,741637685	1,3399786	-0,55346979	0,579942	0,999787
YAF9	25,63314109	-0,238359577	0,9510411	-0,25063015	0,8021	0,999787
YAH1	73,99817193	0,779228498	0,9386615	0,830148537	0,406455	0,999787

YBL053	111,826154	0,430513414	0,8182824	0,526118379	0,598806	0,999787
YBN5	879,9417906	0,604353542	0,7124905	0,848226812	0,396312	0,999787
YBP1	71,1721538	0,512648468	0,8833101	0,580372006	0,561664	0,999787
YCG1	185,8198758	-0,670369952	0,7197898	-0,93134121	0,351677	0,999787
YCK2	330,1706487	-0,508145584	0,7010411	-0,72484416	0,468548	0,999787
YCS4	207,5464988	0,237885744	0,6970243	0,341287593	0,732887	0,999787
YDC1	37,00833882	-0,697763188	0,9213103	-0,75735961	0,448834	0,999787
YDJ1	977,223332	0,717262447	0,6763319	1,060518481	0,288909	0,999787
YEA4	23,83650141	-0,017165042	0,9684145	-0,01772489	0,985858	0,999787
YFH1	41,32539245	0,076131715	0,813489	0,093586656	0,925438	0,999787
YHB4	88,13644378	-0,684771919	0,8945339	-0,76550693	0,44397	0,999787
YHB5	18,3759017	0,25252727	1,0692195	0,236179077	0,813294	0,999787
YHM1	171,5624497	0,541364899	0,9648948	0,561061078	0,574756	0,999787
YHM2	313,5582782	-0,249495479	0,6732104	-0,37060549	0,710931	0,999787
YKE2	47,5379532	-0,498991107	0,7934475	-0,62888986	0,529421	0,999787
YKT6	57,78160724	-0,221718978	0,9279992	-0,23892153	0,811166	0,999787
YKU80	47,65463417	0,392924174	0,7955117	0,493926299	0,621358	0,999787
YMC1	185,5301003	0,590180623	0,7147243	0,825745902	0,408948	0,999787
YMC2	33,53247088	-0,277239557	0,9455064	-0,29321805	0,769355	0,999787
YME1	649,4742591	-0,110088978	0,6622801	-0,16622721	0,867978	0,999787
YML6	112,2568745	0,751841332	0,9745037	0,771512068	0,440403	0,999787
YMX6	35,00748231	0,60158054	0,8812404	0,682652008	0,494827	0,999787
YNK1	755,4395459	0,26095537	0,735116	0,354985313	0,722601	0,999787
YOX1	42,15893208	0,661767591	0,9704944	0,681887077	0,49531	0,999787
YPD1	196,9097588	-0,625932841	0,836278	-0,74847459	0,454174	0,999787
YPS7	52,33751783	-0,07368814	0,7980679	-0,09233317	0,926433	0,999787
YPT31	362,2277403	-0,539042586	0,7590623	-0,71014273	0,477616	0,999787
YPT52	121,8845026	-0,090929883	0,7638425	-0,11904271	0,905242	0,999787
YPT53	31,17736638	-0,887828758	0,97812	-0,90768903	0,364043	0,999787
YPT7	29,56854906	0,126766792	0,8979866	0,141167792	0,887737	0,999787
YPT72	66,03525057	-0,485651909	0,7592242	-0,63966866	0,522388	0,999787
YRB1	602,2564284	0,116547496	0,6755288	0,17252781	0,863023	0,999787
YSA1	33,77560993	-0,069199042	0,9095357	-0,07608173	0,939354	0,999787
YST1	5009,305492	0,088505756	0,6519797	0,135749245	0,89202	0,999787
YTA6	167,9070849	0,013237024	0,6959536	0,019019981	0,984825	0,999787
YUH2	161,1416163	-0,751499456	0,8161311	-0,92080726	0,357151	0,999787
YWP1	974,2944841	-0,592015876	0,800257	-0,73978222	0,459432	0,999787
ZCF1	253,4138966	-1,000682712	0,8832593	-1,13294326	0,257238	0,999787
ZCF10	48,90675232	-0,862134005	0,8712353	-0,98955361	0,322392	0,999787
ZCF11	70,86142493	0,131833301	0,7523437	0,175230157	0,860899	0,999787
ZCF14	6,390166717	1,024780656	1,4668862	0,698609512	0,484796	0,999787
ZCF17	68,7973351	0,151454353	0,8050928	0,188120358	0,850782	0,999787
ZCF18	60,81080228	-0,863803302	0,8191555	-1,05450466	0,291652	0,999787
ZCF2	143,5449788	0,675234031	0,7033689	0,959999819	0,337055	0,999787
ZCF21	278,7850727	0,535972147	0,6927717	0,773663476	0,43913	0,999787
ZCF22	14,89723603	0,063565479	1,0663881	0,059608201	0,952468	0,999787
ZCF23	49,20347958	-0,16078178	0,7892191	-0,20372263	0,83857	0,999787
ZCF27	38,90726538	-0,407483868	0,9033606	-0,45107554	0,651935	0,999787
ZCF28	9,076085512	-0,40005671	1,2443726	-0,32149269	0,747837	0,999787
ZCF29	183,1173495	-0,220148858	0,6974929	-0,31562883	0,752284	0,999787

ZCF3	98,89801804	-0,676799297	0,7673274	-0,88202149	0,377765	0,999787
ZCF30	45,86508411	-0,003399849	0,7993144	-0,00425346	0,996606	0,999787
ZCF31	35,6059773	-0,423468735	0,8355618	-0,5068072	0,61229	0,999787
ZCF32	54,28025876	-0,589103973	0,7923801	-0,74346138	0,457202	0,999787
ZCF35	31,63827636	-0,347153254	1,0153021	-0,34192116	0,73241	0,999787
ZCF39	106,9485291	0,475967477	0,7217952	0,659421783	0,509625	0,999787
ZCF6	22,22301732	1,044068449	1,0174793	1,026132423	0,304829	0,999787
ZCF7	29,26087661	-0,699963517	0,8948724	-0,78219364	0,434101	0,999787
ZCF8	42,20645605	-0,678992278	1,0009908	-0,6783202	0,497569	0,999787
ZCF9	119,4716433	-0,732074567	0,8429421	-0,86847555	0,385134	0,999787
ZDS1	351,3018552	0,352721691	0,6857303	0,514373773	0,606991	0,999787
ZFU2	19,89882499	-0,250620319	1,004483	-0,2495018	0,802973	0,999787
ZNC1	110,1673287	0,067683976	0,719198	0,094110351	0,925021	0,999787
ZPR1	290,4911947	0,927181887	0,8976662	1,032880456	0,30166	0,999787
ZRT1	5,207873879	-1,780949555	1,7233061	-1,03344932	0,301394	0,999787
ZRT2	296,4230621	-0,464248914	0,6858815	-0,67686466	0,498492	0,999787
ZSF1	502,4616622	-0,648165891	0,6728664	-0,96329057	0,335402	0,999787
ZUO1	1068,098044	0,703143838	0,6828766	1,029679223	0,303161	0,999787
ZWF1	187,9094827	0,626136435	0,7844801	0,798154686	0,424781	0,999787
tR(CCG)1	20,05376677	-0,984545892	1,0988068	-0,89601364	0,370245	0,999787
_C402340WA	33,33890529	0,000201938	0,8831683	0,000228652	0,999818	0,999818
ADH3	2,30256212	0,124132495	2,1980028	0,05647513	0,954963	NA
ALK8	4,539374304	-2,243247215	1,733517	-1,29404394	0,19565	NA
ARC18	4,398845248	-0,39116897	1,7327601	-0,22574907	0,821397	NA
ARG3	5,014308283	0,097007199	1,614114	0,060099347	0,952077	NA
BET4	4,118275529	-0,367849372	1,8296821	-0,20104551	0,840663	NA
_C100370WA	4,962953449	-0,0170571	1,543799	-0,01104878	0,991185	NA
_C100820WA	4,906784202	-1,8257556	1,6052669	-1,1373533	0,255391	NA
_C101180CA	4,257035061	-1,058789512	1,7207508	-0,61530671	0,538352	NA
_C104010CA	4,555385515	-0,039499354	1,5719694	-0,0251273	0,979953	NA
_C104150CA	1,926623123	1,186775592	2,478066	0,478912013	0,632001	NA
_C104270CA	2,919794679	-0,233570925	1,9430174	-0,12021041	0,904316	NA
_C105150CA	3,558434841	-2,251856804	2,0481345	-1,09946722	0,271564	NA
_C105920WA	4,931083278	0,41394681	1,5377296	0,269193501	0,787781	NA
_C106120CA	2,644456244	0,269722496	2,2411632	0,120349333	0,904206	NA
_C106340WA	2,170244803	-0,025800941	2,3162896	-0,01113891	0,991113	NA
_C107450WA	2,379909429	-0,539619059	2,1752598	-0,24807108	0,804079	NA
_C107530WA	3,856467936	-2,420598732	2,0951898	-1,15531241	0,247963	NA
_C108150CA	2,415482592	-1,219690615	2,0576458	-0,59276023	0,553342	NA
_C108830CA	1,402672958	0,54644327	2,7448611	0,199078657	0,842201	NA
_C109500WA	1,984649713	4,016564066	2,6742652	1,501931821	0,133115	NA
_C109930WA	2,427473451	0,007161586	2,2895716	0,003127916	0,997504	NA
_C109940WA	2,854438229	1,542372284	2,0467987	0,75355347	0,451117	NA
_C111800CA	2,230128736	-0,572980166	2,1445231	-0,26718303	0,789328	NA
_C111910WA	2,431176444	0,157017091	2,0606346	0,076198414	0,939261	NA
_C112000CA	3,711918527	-1,870202784	1,8534652	-1,00903042	0,31296	NA
_C112110CA	2,646630946	0,093994442	2,0838698	0,045105718	0,964023	NA
_C112280CA	4,780015528	-0,545970945	1,7027842	-0,32063426	0,748488	NA
_C112480WA	2,410010075	0,134747331	2,1119601	0,063802026	0,949128	NA
_C112640WA	3,893645516	0,299211795	1,7178411	0,174178971	0,861725	NA

_C112690CA	4,684241073	-1,014256639	1,6009656	-0,63352808	0,526389	NA
_C112800WA	2,956896133	-0,591413292	1,9116488	-0,30937341	0,757037	NA
_C112980WA	3,763185542	-1,157330838	1,7373649	-0,66614147	0,505321	NA
_C113000WA	1,963724577	0,668210618	2,356719	0,283534275	0,776767	NA
_C113180WA	4,523439219	-1,88292079	1,7695094	-1,064092	0,287287	NA
_C113290WA	2,695723259	0,872242625	2,0207052	0,43165258	0,665994	NA
_C113430CA	2,889694033	-0,92050314	2,1766201	-0,42290482	0,672365	NA
_C113590WA	2,055478681	-0,886932016	2,211642	-0,40102875	0,688399	NA
_C114630CA	4,356183457	0,66841232	1,7474632	0,382504373	0,702087	NA
_C201050WA	3,81082569	1,505669665	1,8432892	0,816838529	0,414021	NA
_C201570WA	4,853494736	1,619691236	1,6928197	0,95680082	0,338668	NA
_C201730WA	4,156993092	-0,709284972	1,6590488	-0,42752508	0,668997	NA
_C202110CA	1,667219773	1,515482148	2,5742653	0,588704737	0,556059	NA
_C202230CA	4,313457234	-4,348504337	2,2791414	-1,90795727	0,056397	NA
_C202640CA	4,184601677	-1,738032271	2,00246	-0,86794855	0,385422	NA
_C202750CA	2,745144624	-0,453523619	1,9844344	-0,22854049	0,819226	NA
_C204090WA	3,059112804	-1,052098571	1,9808427	-0,53113686	0,595324	NA
_C204450WA	3,228607701	1,598949998	1,979958	0,807567619	0,41934	NA
_C204740CA	2,251295105	-0,545911588	2,1025439	-0,25964337	0,795139	NA
_C205870WA	1,995353514	1,825578119	2,3957701	0,762000548	0,44606	NA
_C206280CA	3,881654657	-0,446545838	1,7036739	-0,26210758	0,793239	NA
_C206930CA	4,641908335	-1,044989583	1,5703479	-0,66545099	0,505762	NA
_C207700CA	4,442465043	0,666476483	1,6117976	0,413498861	0,679241	NA
_C208170WA	4,308302076	-0,570842212	1,6351597	-0,34910487	0,727011	NA
_C208340CA	4,677164139	-3,464292007	1,8279651	-1,89516308	0,058071	NA
_C208690CA	4,585410036	-1,892086869	1,6419432	-1,15234611	0,249179	NA
_C208750WA	2,858065096	-1,946884518	2,0462814	-0,95142562	0,341388	NA
_C209110CA	3,553279683	0,409064337	1,7533045	0,233310497	0,81552	NA
_C209550CA	2,379909429	-0,539619059	2,1752598	-0,24807108	0,804079	NA
_C209630CA	4,72850728	-0,076451846	1,5698555	-0,04869993	0,961158	NA
_C209880CA	4,105397025	0,153561878	1,7188321	0,089340828	0,928811	NA
_C210060CA	4,077471081	-0,291058258	1,6791361	-0,1733381	0,862386	NA
_C210070WA	3,380728204	-3,043914327	1,96072	-1,55244719	0,120555	NA
_C210300CA	4,449794903	-1,079252648	1,6151335	-0,66821266	0,503998	NA
_C210320CA	4,931324512	-0,463720516	1,5522704	-0,29873694	0,765141	NA
_C210800WA	3,59408413	0,23390041	1,8639582	0,125485869	0,900139	NA
_C300410CA	1,916160555	0,128126735	2,4188626	0,052969828	0,957756	NA
_C301110CA	2,921640329	1,421931777	2,046688	0,694747698	0,487213	NA
_C301340WA	3,832233292	0,392220249	1,8833697	0,208254517	0,83503	NA
_C301440CA	2,447111529	-0,269668151	2,0276233	-0,13299717	0,894196	NA
_C301640CA	3,757954258	-1,520930238	1,7486426	-0,86977764	0,384422	NA
_C302010CA	4,846417802	0,371169232	1,5931183	0,232982841	0,815775	NA
_C302200WA	1,429070611	1,245153108	2,7047231	0,460362503	0,645256	NA
_C302360CA	2,11350527	0,849342913	2,2529543	0,37699075	0,70618	NA
_C302980CA	4,251879903	0,832477403	1,6492242	0,504769103	0,613721	NA
_C303570CA	3,964068142	3,684667344	2,0384718	1,807563549	0,070674	NA
_C303690WA	4,031194117	0,706283178	1,7225333	0,410025842	0,681787	NA
_C304100WA	2,118977788	-0,78780703	2,3643077	-0,33320834	0,738977	NA
_C304170WA	1,45023698	1,268406813	2,6823446	0,472872429	0,636304	NA
_C304210WA	3,620164424	-0,67129932	1,8010246	-0,3727319	0,709348	NA

_C304440CA	3,254929228	-0,768129915	1,9247553	-0,39907925	0,689835	NA
_C305080WA	3,428050992	-0,721544969	1,7825749	-0,40477681	0,685642	NA
_C305330CA	2,016519883	1,841617903	2,3896211	0,770673594	0,4409	NA
_C305820WA	1,731047932	-1,280734421	2,5194753	-0,50833378	0,611219	NA
_C306880WA	1,886059909	-0,65848208	2,2930941	-0,28715876	0,773991	NA
_C400850CA	2,724219488	-2,585382331	2,1920162	-1,17945404	0,238217	NA
_C400940WA	4,75304759	1,075466873	1,7015685	0,632044419	0,527358	NA
_C401310WA	4,823635324	0,394592538	1,565277	0,252091183	0,800971	NA
_C401600CA	2,879231465	-1,908925255	2,042335	-0,93467783	0,349954	NA
_C402460WA	4,384362327	-2,706348005	1,7456037	-1,55037939	0,12105	NA
_C402580WA	3,537268472	-2,273842402	1,9360813	-1,17445606	0,240212	NA
_C402660WA	4,835626182	1,141020524	1,6310705	0,699553168	0,484206	NA
_C403340CA	3,266279441	-1,532686441	2,0134054	-0,76124087	0,446513	NA
_C403460CA	4,97527336	-0,519363094	1,5476413	-0,33558363	0,737185	NA
_C403870CA	2,683491167	1,273861321	2,1076845	0,604389009	0,545585	NA
_C404630CA	4,156422807	1,796772925	1,7532817	1,024805593	0,305455	NA
_C405250WA	4,142827531	-1,439784058	1,6649362	-0,8647683	0,387166	NA
_C405830WA	4,442465043	0,666476483	1,6117976	0,413498861	0,679241	NA
_C406180CA	2,457574097	0,656651816	2,0733162	0,316715718	0,751459	NA
_C406200WA	4,419694257	-1,571775558	1,6252927	-0,9670723	0,333508	NA
_C406620CA	4,950327872	-4,604261475	2,2335969	-2,06136636	0,039268	NA
_C500620WA	2,950136558	-1,173395301	1,9013303	-0,61714436	0,53714	NA
_C500810CA	3,808903914	-3,160825673	2,1344277	-1,48087739	0,138639	NA
_C501020CA	1,95326201	-0,318630687	2,3578285	-0,13513734	0,892503	NA
_C501160CA	2,21419365	-0,114795926	2,3413685	-0,04902941	0,960896	NA
_C501990WA	2,457574097	0,656651816	2,0733162	0,316715718	0,751459	NA
_C502980CA	2,101514412	-0,556192381	2,2903328	-0,24284347	0,808127	NA
_C503210CA	4,065238989	0,052072598	1,7733586	0,029363829	0,976574	NA
_C503770CA	2,658863038	-0,420817296	2,0204344	-0,2082806	0,83501	NA
_C503890CA	2,879231465	-1,908925255	2,042335	-0,93467783	0,349954	NA
_C503970WA	3,013394432	0,322835341	1,8737088	0,172297503	0,863204	NA
_C504010CA	4,205768046	-1,705916201	1,9668998	-0,86731221	0,385771	NA
_C504850WA	2,632465385	-0,858941806	2,0199544	-0,42522831	0,67067	NA
_C504940WA	2,852833812	-2,643152131	2,1003618	-1,25842704	0,208237	NA
_C505010WA	4,893905698	-1,378601227	1,6792626	-0,82095631	0,411671	NA
_C505060CA	4,60665253	0,281767597	1,5818712	0,178122975	0,858626	NA
_C505240CA	4,354096574	0,603433246	1,6603715	0,363432669	0,716282	NA
_C600630WA	4,010027748	0,692346915	1,735373	0,39896145	0,689922	NA
_C601020WA	2,270933183	-0,739094231	2,226643	-0,33193208	0,739941	NA
_C601380CA	3,006634857	-0,229698877	1,9832353	-0,11582028	0,907795	NA
_C601530CA	4,632974058	-1,520167681	1,6784361	-0,90570485	0,365092	NA
_C601680CA	3,655496354	0,125350077	1,7464339	0,071774878	0,942781	NA
_C601810WA	2,535238765	1,659217832	2,186766	0,758754162	0,448	NA
_C602760WA	1,397441674	-0,121462567	2,7233094	-0,04460109	0,964425	NA
_C602950CA	3,073519598	-1,63159844	1,8801327	-0,86781025	0,385498	NA
_C603240WA	3,890271575	-1,315050941	1,7552352	-0,74921638	0,453727	NA
_C603280WA	3,808903914	-3,160825673	2,1344277	-1,48087739	0,138639	NA
_C603420WA	3,011866141	0,085897512	1,9785655	0,043414035	0,965371	NA
_C604070CA	3,126314904	-0,751982766	1,8585038	-0,40461728	0,685759	NA
_C604320CA	3,62757041	-0,382887008	1,7621585	-0,21728295	0,827988	NA

_C700460WA	2,791180355	-0,255407759	1,9188093	-0,13310742	0,894108	NA
_C700870WA	4,461709636	-4,446050826	2,2203665	-2,00239501	0,045242	NA
_C701010WA	3,796660129	0,872957419	1,750512	0,498686914	0,618	NA
_C701050WA	4,442388917	-1,448506756	1,671716	-0,86647897	0,386228	NA
_C701130CA	2,180707371	0,87828782	2,2750151	0,386058014	0,699454	NA
_C701170CA	3,699927668	-3,140932881	1,9125124	-1,64230723	0,100526	NA
_C701390WA	3,962704959	-0,762582193	1,6634951	-0,45842166	0,64665	NA
_C702130WA	4,318688518	-3,303841411	2,1279821	-1,55257015	0,120526	NA
_C702280WA	3,207365206	-1,222244708	1,9327812	-0,63237614	0,527141	NA
_C702290WA	2,282924042	0,467919833	2,1251403	0,220183034	0,825729	NA
_C703140WA	2,498378544	0,317961317	2,1283983	0,149389951	0,881246	NA
_C703460WA	1,886059909	-0,65848208	2,2930941	-0,28715876	0,773991	NA
_C703470WA	2,697580602	-0,966365106	2,1624802	-0,44687814	0,654963	NA
_CR00710CA	2,673028599	0,472340416	2,0742878	0,227712087	0,81987	NA
_CR00830WA	2,678259883	0,853780126	2,0848879	0,409508888	0,682166	NA
_CR01340WA	3,394247353	-1,650667402	2,1296836	-0,77507634	0,438295	NA
_CR01530CA	1,45023698	1,268406813	2,6823446	0,472872429	0,636304	NA
_CR01920WA	4,897279639	-0,029604523	1,8259352	-0,01621335	0,987064	NA
_CR02630CA	4,527700805	-1,367000897	1,6523559	-0,82730415	0,408065	NA
_CR02800CA	4,318764644	-0,030321818	1,6853599	-0,0179913	0,985646	NA
_CR03480WA	4,375186817	-1,773806286	1,8073437	-0,98144379	0,326374	NA
_CR04400WA	2,254998098	-0,327744601	2,2709932	-0,14431774	0,88525	NA
_CR04610CA	2,77524527	0,109923249	1,9533483	0,05627427	0,955123	NA
_CR04850CA	3,145952983	-0,903023326	1,8532766	-0,48725771	0,626076	NA
_CR06190CA	1,45023698	1,268406813	2,6823446	0,472872429	0,636304	NA
_CR06270WA	2,642927953	-0,037540778	1,9698123	-0,01905805	0,984795	NA
_CR06500CA	2,487674743	1,137418859	2,1737323	0,523256186	0,600796	NA
_CR06510WA	4,601180013	1,004819444	1,6126895	0,623070628	0,533238	NA
_CR06920WA	2,600595215	-0,077134702	1,9943226	-0,03867714	0,969148	NA
_CR08140WA	3,276171723	2,081984667	2,0131617	1,034186506	0,301049	NA
_CR10120CA	3,435127927	0,989130673	1,8957814	0,521753545	0,601842	NA
_CR10200WA	5,13052657	-1,218910272	1,5345084	-0,79433277	0,427002	NA
_CR10310WA	3,096214258	-1,464866455	2,1096948	-0,69434993	0,487463	NA
CDA2	2,565263285	-1,20632882	2,0295211	-0,59439087	0,552251	NA
DAL4	2,313265921	-0,680251352	2,2567231	-0,30143323	0,763084	NA
DAL5	4,472489563	-0,949796141	1,5970679	-0,59471245	0,552036	NA
DIE2	1,582554297	1,435879298	2,658911	0,540025342	0,58918	NA
DUR35	5,017288738	-1,756416326	1,9113183	-0,91895542	0,358119	NA
FAV2	2,39061323	-1,607901429	2,1287464	-0,75532784	0,450052	NA
FET99	2,618058591	-0,197071092	2,0754692	-0,09495255	0,924353	NA
FGR42	4,724486927	-1,29958544	1,7494776	-0,74284199	0,457577	NA
FGR46	3,202133922	-1,665548518	1,9180632	-0,86834915	0,385203	NA
FRP5	2,452342813	0,177821521	2,038827	0,087217562	0,930499	NA
FUS1	3,664354505	-2,459370012	1,999264	-1,23013767	0,218646	NA
GPI1	3,496781384	-0,336180881	1,7674537	-0,19020633	0,849147	NA
GPX1	3,24977407	1,607909521	1,9702029	0,816113652	0,414435	NA
HAK1	2,703053119	-2,634410856	2,0959813	-1,25688661	0,208795	NA
HSP31	4,691329699	-2,055039572	1,7713132	-1,16017857	0,245976	NA
HWP2	2,528403064	-3,613115122	2,4235118	-1,49085933	0,135998	NA
IFA4	3,865719572	-0,196779242	1,6863777	-0,11668753	0,907108	NA

IMG2	1,9547903	0,021230349	2,2992353	0,009233656	0,992633	NA
KAR5	1,418608043	-0,090619455	2,6835909	-0,03376798	0,973062	NA
KTI11	2,371051277	2,903872025	2,3256511	1,248627569	0,211801	NA
LDG3	1,476634633	2,155616136	2,7103946	0,795314491	0,426431	NA
01-lip	3,3678497	-2,173310882	2,0135484	-1,07934372	0,280435	NA
09-lip	1,651284688	2,336198813	2,6066625	0,896241403	0,370124	NA
LYS5	2,586429654	-1,177654264	2,0511067	-0,57415552	0,565863	NA
MED22	1,418608043	-0,090619455	2,6835909	-0,03376798	0,973062	NA
PGA15	2,986996779	-0,054071659	1,867488	-0,02895422	0,976901	NA
PGA19	4,200536762	-2,086904501	1,8537541	-1,12577199	0,260262	NA
PGA30	4,7809091	-4,561762416	2,3740131	-1,92154052	0,054664	NA
PGA61	1,593258098	0,203822615	2,5595105	0,079633437	0,936529	NA
PHO112	2,758023127	-1,199575782	2,1212356	-0,56550804	0,571728	NA
PHO8	4,714341719	-0,619027035	1,5535801	-0,39845195	0,690297	NA
PSF1	4,107571727	0,076086398	1,7710929	0,04296014	0,965733	NA
RBT7	1,886059909	-0,65848208	2,2930941	-0,28715876	0,773991	NA
RTA4	3,879885133	0,419367734	1,8143875	0,231134602	0,81721	NA
SAP6	1,995353514	1,825578119	2,3957701	0,762000548	0,44606	NA
SAP7	4,65968907	-0,121022323	1,6502956	-0,07333372	0,941541	NA
SEN15	4,504435859	0,516476042	1,7149216	0,301165977	0,763288	NA
SFT1	3,458151639	-0,247041479	1,7932458	-0,1377622	0,890428	NA
SNR6	1,577323013	0,794700855	2,6384568	0,301199118	0,763263	NA
SOD4	1,630118319	2,317650522	2,6214696	0,884103536	0,37664	NA
SPC19	4,927697645	1,14325163	1,6749133	0,682573606	0,494876	NA
SPO1	4,985735927	-0,066501919	1,5798765	-0,04209311	0,966424	NA
SPO11	4,034820984	-1,684186805	1,7843612	-0,94385979	0,345241	NA
SPO22	2,07664505	-0,854494592	2,1801791	-0,39193779	0,695104	NA
SPR28	3,533641604	0,705464737	1,8568424	0,379927094	0,704	NA
STE18	3,742019173	-1,180072135	1,7499732	-0,67433726	0,500097	NA
TEP1	3,779120627	-1,499517735	1,7830122	-0,84100253	0,400347	NA
UGA6	3,521333386	-1,804138947	1,9556035	-0,92254842	0,356243	NA
UME6	2,877703174	-2,066684907	1,9760051	-1,04589045	0,295612	NA
UME7	4,518207935	-2,275077899	1,7566746	-1,29510488	0,195284	NA
VMA22	2,673028599	0,472340416	2,0742878	0,227712087	0,81987	NA
ZCF19	3,250015304	0,270949179	1,9468842	0,139170672	0,889315	NA
ZCF25	3,27979859	-0,557560978	1,8050289	-0,3088931	0,757403	NA
tG(CCC)1	2,087107617	0,226717588	2,2041587	0,102859012	0,918075	NA
tG(GCC)6	1,836638544	1,080488419	2,4718065	0,437124997	0,662021	NA
tH(GUG)3	2,734682056	-1,363833913	1,9709011	-0,69198497	0,488947	NA
tI(UAU)1	3,027483867	-2,072089194	1,9838289	-1,04448986	0,296259	NA
tL(AAG)1	4,632974058	-1,520167681	1,6784361	-0,90570485	0,365092	NA
tL(AAG)2	3,556665316	-0,741383958	1,8680088	-0,39688463	0,691453	NA
tL(CAA)6	4,297522149	-3,369014795	2,2316981	-1,50961942	0,131141	NA
tP(UGG)2	2,11350527	0,849342913	2,2529543	0,37699075	0,70618	NA
tP(UGG)3	2,554559484	-0,275882298	2,1262371	-0,12975142	0,896763	NA
